# Regioselective activation of benzocyclobutenones and dienamides lead to anti-Bredt bridged-ring systems by a [4+4] cycloaddition

**DOI:** 10.1038/s41467-021-23344-0

**Published:** 2021-05-21

**Authors:** Jianyu Zhang, Xi Wang, Tao Xu

**Affiliations:** 1 Molecular Synthesis Center and Key Laboratory of Marine Drugs, Ministry of Education, School of Medicine and Pharmacy, Ocean University of China, Qingdao, China; 2 Laboratory for Marine Drugs and Bioproducts and Open Studio for Druggability Research of Marine Natural Products, Pilot National Laboratory for Marine Science and Technology, Qingdao, China

**Keywords:** Catalyst synthesis, Synthetic chemistry methodology

## Abstract

To the best of our knowledge, bridgehead carbon benzofused-bridged ring systems have previously not been accessible to the synthetic community. Here, we describe a formal type-II [4 + 4] cycloaddition approach that provides fully sp^2^-carbon embedded anti-Bredt bicyclo[5.3.1] skeletons through the Rh-catalyzed C_1_–C_8_ activation of benzocyclobutenones (BCBs) and their coupling with pedant dienamides. Variously substituted dienamides have been coupled with BCBs to provide a range of complex bicyclo[5.3.1] scaffolds (>20 examples, up to 89% yield). The bridged rings were further converted to polyfused hydroquinoline-containing tetracycles via a serendipitously discovered transannular 1,5-hydride shift/Prins-like cyclization/Schmidt rearrangement cascade.

## Abstract (web summary)

Bridgehead carbon benzofused-bridged ring systems have previously not been accessible via synthetic approaches. Here, the authors report a formal type-II [4 + 4] annulation approach that provides fully sp_2_-carbon embedded anti-Bredt bicyclo[5.3.1] skeletons through the Rh-catalyzed C1–C8 activation of benzocyclobutenones and their coupling with pedant dienamides.

## Introduction

**Fig. 1:**
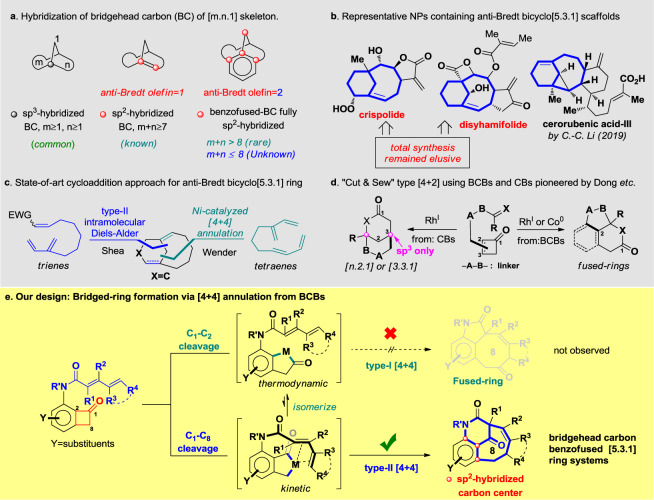
Representative natural products and design of a [4 + 4] annulation approach to access anti-Bredt bicyclo[5.3.1] skeleton. **a** Hybridization of bridgehead carbon of [m.n.1] skeleton. **b** Representative NPs containing anti-Bredt bicyclo[5.3.1] scaffolds. **c** State-of-art cycloaddition approaches for anti-Bredt bicyclo[5.3.1] rings. **d** “Cut and sew” type [4 + 2] using BCBs and CBs pioneered by Dong, etc. **e** Our design: bridged ring formation via [4 + 4] annulation from BCBs.

Bridged ring systems are key structural motifs in many important functional molecules^1–3^. The skeleton were characterized by its size (e.g., bicyclo[m.n.1]) and ring strain (hybridization form of the bridgehead carbon (BC)). While the sp^3^-hybridized BC are commonly seen in many natural products, the sp^2^-hybridized BC underpins certain limitation on both the ring size (m + n ≥ 7) as well as ring strain^3^. However, consecutive sp^2^-hybridized carbon centers and/or bridgehead benzofused [m.n.1] systems are unusual (m + n > 8) and, to the best of our knowledge, unknown to chemist when it was confined in a small ring (m + n ≤ 8) framework (Fig. 1a)^4,5^. We are herein set off to challenge these limitations. Bicyclo[5.3.1] bridged ring systems with anti-Bredt olefins at the bridgehead position are widely found in bioactive natural products (Fig. 1b)^6–8^, whose total synthesis was highly dictated by the efficiency of assembling such scaffolds^9,10^. The state-of-art cycloaddition methods in constructing anti-Bredt bicyclo[5.3.1] scaffolds^11–14^ are two: (1) type-II intramolecular Diels–Alder reactions developed by Shea et al.^15,16^, which requires activating substituent on dienophile, otherwise the reactions are mostly in gas phase; (2) a seminal report by Wender et al.^17,18^ using Ni-catalyzed type-II [4 + 4] intramolecular cycloaddition under diluted conditions between 1,3-dienes (Fig. 1c)^19^. A general, catalytic and diversifiable cycloaddition strategy remained elusive, especially for the highly strained anti-Bredt^20,21^ bridged skeletons. The transition metal-catalyzed intramolecular [4 + 2] annulation via C_1_–C_2_ bond activation^22–33^ of benzocyclobutenones (BCBs) has emerged as an attractive approach for preparing fused- and bridged ring systems pioneered by Liebeskind^34^ and Dong^35,36^ (Fig. 1d). However, no catalytic [4 + 4] annulation via oxidative C–C activation has been known, not to mention forming the highly strained anti-Bredt bridged ring systems^37^. Accessing anti-Bredt bridged bicycles remained elusive since only a few examples had been known starting from cyclobutanones^38–43^. To the best of our knowledge, no report had been known to construct bridged ring systems that bears highly strained consecutive double bond surrounding the BC.

Herein, we disclose our discovery of a type-II [4 + 4] annulation between BCBs and dienamides enabled by Rh-catalyzed regioselective C_1_–C_8_ bond activation (Fig. 1e).

## Results and discussion

**Fig. 2:**
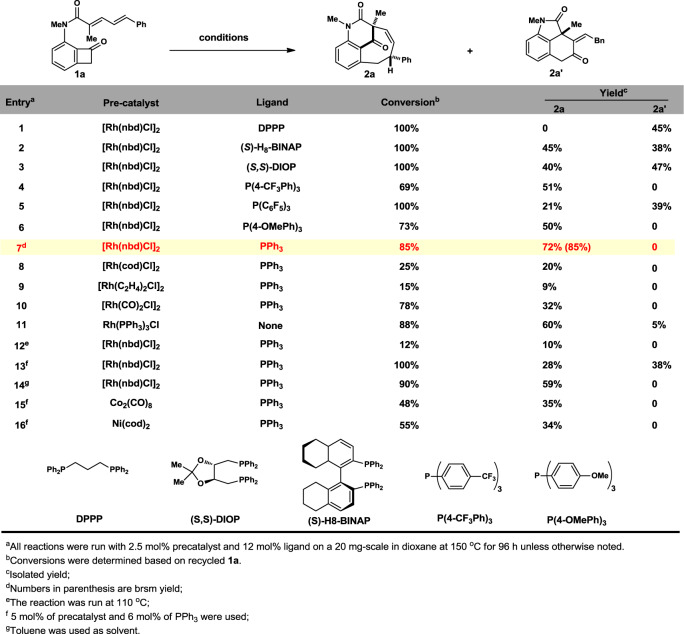
Condition optimizations. Precatalysts, ligands, temperatures and their ratios have been screened.

The challenges are three-fold: (1) there is no precedent report of benzo-fused BC in bicyclo[5.3.1] systems; (2) the kinetically C_1_–C_8_ rhodaindanone would be readily isomerize to its thermodynamically stable C_1_–C_2_ cleaved counterpart^44^; (3) competitive fused ring formation through normal cut and sew pathway. Our investigation commenced with preparation of **1a** through amide formation in 72% yield between our previously reported 3-NHMe BCB and the known dienoic acid (see supporting information (SI) for detail)^45^. We tested our hypothesis by using 2.5 mol% of [Rh(nbd)Cl]_2_ and 12 mol% of DPPP, unfortunately, only the fused tricycle **2a′** was isolated in 45% yield, presumably from the “cut and sew” process followed by a double bond migration (entry 1, Fig. 2). It is noted that the type-I [4 + 4] benzofused ring^46,47^ product was not observed. When switching to other bidetate ligand with varied bite-angles, namely (*S*)-H_8_-BINAP and (*S*,*S*)-DIOP. Surprisingly, the desired anti-Bredt benzofused aza-bicyclo[5.3.1] bridged product **2a** was successfully isolated (45% and 40% yield, respectively) together with the side product **2a′** (38% and 47% yield, respectively, entry 2 and 3). In both cases, product **2a**’s ee values were 0. These results led us to speculate that only one phosphine-center served as dative ligand based on the 18d-electron rule. Mono-dentate P(4-CF_3_Ph)_3_ greatly improved the selectivity yielding only **2a** in 51% yield (entry 4). However, the analogous P(C_6_F_5_)_3_ only lead to significantly decreased yield of **2a** (21%) and selectivity (39% yield of **2a′**), presumably due to the loose coordinating ability owing to its π-acidity as well as the large cone angle (entry 5)^48^. The electron-rich P(4-OMePh)_3_ provided elevated yield (50%) of **2a** albeit with 73% conversion (entry 6). Surprisingly, it was found that the feedstock PPh_3_ provide the highest 72% isolated yield based on 85% conversion while preserving the complete selectivity for the bridged products (entry 7, with light yellow background). Encouraged by this result, a series of pre-catalysts were screened, but the yields of **2a** ranged between 9 and 32% (entry 8–10). The Wilkinson catalyst was also used but proved less chemoselective as both **2a** and **2a′** were obtained (entry 11). An attempt to lower the reaction temperature from 150 to 110 °C proved unfruitful, as **2a** was only obtained in 10% yield with most of **1a** recovered (entry 12). The Rh/ligand ratio was also proved crucial to the success of this [4 + 4] annulation pathway, since decreased efficiency (28% yield of **2a**) and loss of selectivity (38% yield of **3a**) was observed when lowering the loading of PPh_3_ (entry 13). Toluene is an inferior solvent (entry 14). First row late transition metal are known to cleave C_1_–C_8_ bond of BCB, but Co^0^ and Ni^0^ complex were tested and proved less efficient (entry 15–16).

**Fig. 3:**
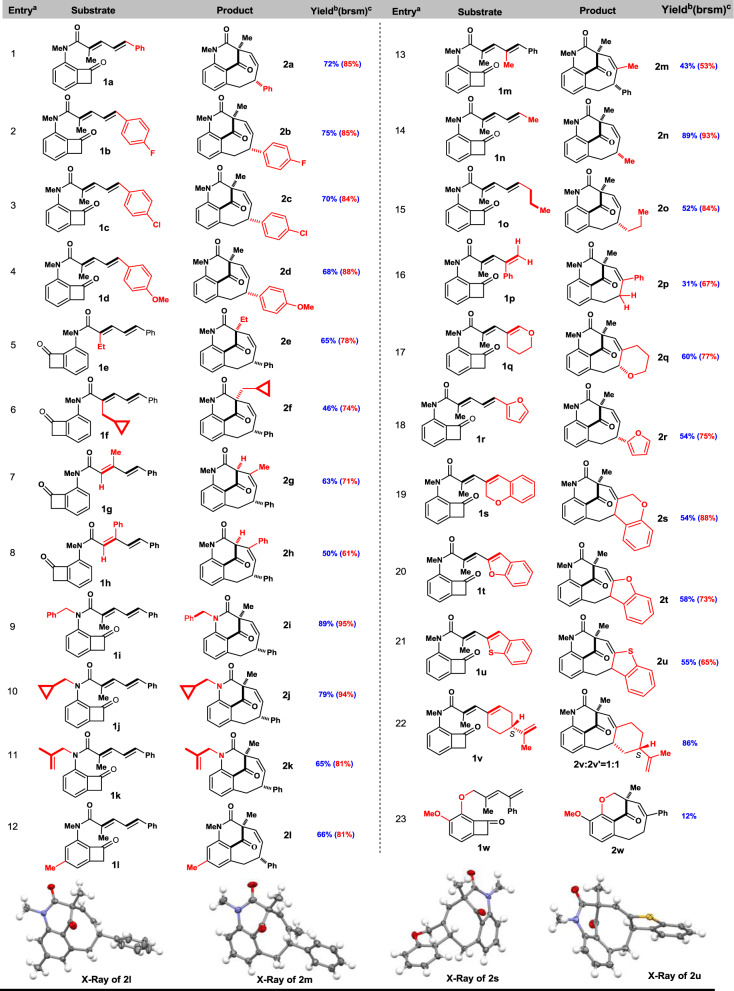
Substrate scope. ^a^Conditions: all reactions were run with 2.5 mol% rhodium complex and 12 mol% ligand in dioxane at 150 °C for 96 h unless otherwise noted; ^b^isolated yield; ^c^numbers in parenthesis are brsm yields. X-ray structures were shown for compounds **2l**, **2m**, **2s**, and **2u**.

With optimal condition in hand, we set off to explore the reaction scope (Fig. 3). A broad series of dienamide coupled BCB substrates with different steric and electronic properties have been examined. Delightedly, good to high yields of bicyclo[5.3.1] bridged rings were obtained. It was found that changing the electronegativity of aryl substituents on δ-position of dienamides (**1a–1d**) does not affect the efficacy, and 68–75% yields were obtained, with a slight trend for *para*-substituted effect favoring electron-withdrawing groups (entry 1–4, Fig. 3). The alkyl substituents at α and β-position of dienamide were also investigated (entry 5–8). It was gratifyingly to find that ethyl (**1e**), cyclopropylmethyl (**1f**) as well as hydrogen (**1g–h**) at α-position were all well tolerated yielding desired bridged products **2e–h** in useful yields (46–65%). It is noteworthy that no cleavage of cyclopropyl moiety was detected, revealing good chemoselectivity of this catalyst system. Variation of R group on the nitrogen promised potential use in complex settings, as 89% yield of **2i** was isolated (95% brsm), when Bn (**1i**) was used (entry 9). Increasing the steric bulkiness to cyclopropylmethyl (**1j**) resulted in no less efficiency (79% yield of **2j**, entry 10). The reaction demonstrated excellent chemoselectivity when **1k** was employed as substrate, only bridged [4 + 4] product **2k** (79% yield, 94% brsm) was observed but no traditional [4 + 2] product (entry 11). The robustness toward electronic variation on BCB was enlightened by obtaining **2l** in 66% yield (81% brsm, entry 12). A grand challenge in “migratory insertion” is the unviability of sterically demanding multi-substituted olefins as coupling partners, presumably due to their low binding affinity. We postulated that dienamides are potential chelating ligands, which increased their binding ability to the rhodaindanone complex. The tetrasubstituted dienamide **1m** was synthesized and the resulting [4 + 4] annulation product **2m** (84% brsm yield) was undoubtedly verified through X-ray crystallography together with the aforementioned **2l** (entry 12 and 13). A major competitive side reaction after migratory insertion is the tendency to undergo β-H elimination when a β-H is available. Gratifyingly, in our trials to effect the alkyl substituted dienamides **1n** and **1o** to the benzo-fused bridged skeletons, high to moderate yields were obtained, respectively, for **2n** (89% yield) and **2o** (52% yield) as the only isolated product in each case (entry 14 and 15). The terminal dienamide **1p** underwent a certain decomposition which cause isolation of desired **2p** in 31% yield albeit with moderate 67% brsm yield (entry 16). When the electronic property of dienamide was altered (**1q**), the desired [4 + 4] annulation took place without loss of efficacy, obtaining **2q** in 60% yield (entry 17). It was very encouraging to find that extended conjugated tetraenamide **1r** react smoothly yielding **2r** in 54% yield and the electron-rich furan moiety was well preserved (entry 18). When using even challenging (possessing *cis*-terminal olefins) cyclic dienamides **1s**–**u**, the desired [4 + 4] annulation reactions proceeded without any difficulties, affording **2s**~**u** in synthetically useful yields (54–58%, entry 19–21). The product **2t** and **2u** suggested that aromaticity can be deprived, since the X-ray crystallographic experiment undisputedly showed an *exo*-olefin for product **2u** even with the possibility for re-aromatization (Fig. 3, bottom). This might be caused by the highly strained anti-Bredt aza-bicyclo[5.3.1] skeleton. An effort to generate enantioselective bridged rings through chiral substrate induction was carried out using **1v**, delightedly, the resulting product **2v** and **2v′** was obtained in a 1:1 ratio as inseparable isomers in a combined 86% yield (entry 22). These provide valuable clues in our future endeavor toward enantioselective [4 + 4] annulation study. Additionally, we also found the ether substrate **1w** yielded desired [4 + 4] product **2w**, albeit in 12% yield. This indicated that our [4 + 4] conditions can be extrapolated to O linked diene substrate albeit in low yield (entry 23).

**Fig. 4:**
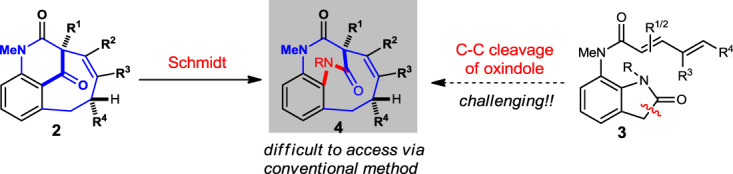
Derivatization hypothesis. We postulated that a Schmidt reaction of **2** maybe able to afford [5.3.2]bridged compound like **4**, which is very challenging using conventional C–C activation method.

With the highly diastereomerically pure benzo-fused bridged cyclic compounds **2** in hand, we hypothesized that if a Schmidt rearrangement reaction can be performed (Fig. 4), which would further extended the structural diversity^49–53^ based on this [4 + 4] annulation strategy. In addition, it may be considered as a reaction surrogate for using the oxindole **3** as the annulation substrate, which has been an elusive substrate in C–C bond activation due to the more reactive amide bonds.

**Fig. 5:**
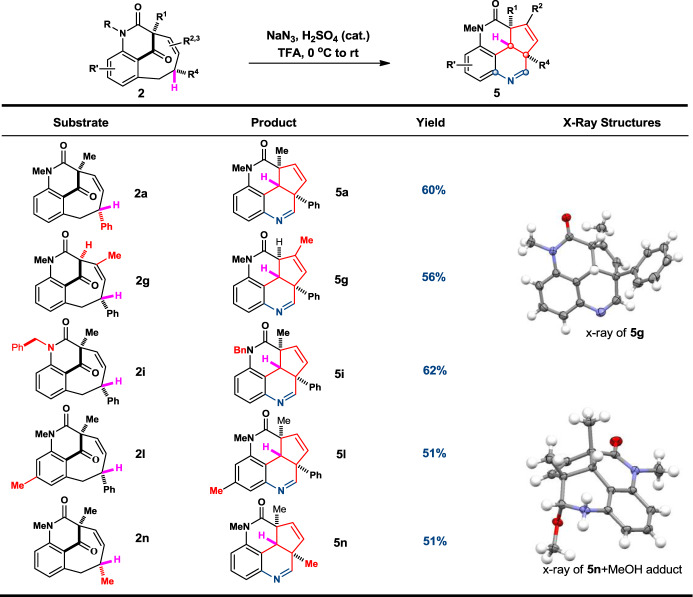
Cascade rearrangement for fuse-ring system. Conditions: **2** (1.0 equiv.) and H_2_SO_4_ (5 drops) in TFA at 0 °C, then NaN_3_ (3.0 equiv) at rt. X-ray structures was obtained for **5g** and **5n** + MeOH.

**Fig. 6:**
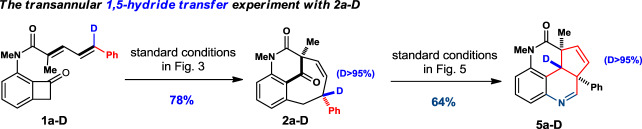
Mechanism probing of the cascade rearrangement. Standard conditions of [4 + 4] converted **1a-D** to product **2a-D** diastereoslelctively and with >95% Deuterium preserved. Standard conditions of cascade rearrangement afforded **5a-D** with >95% Deuterium.

After extensive investigations, we were delighted to discover that the bicyclo[5.3.1] bridged tricycles can readily undergo a clean rearrangement reaction with isolated yields ranging from 51 to 62% yield (Fig. 5). A careful cultivation of a single crystal of the product **5g/5n** and X-ray crystallographic analysis revealed that a densely functionalized and polyfused ring systems were obtained. Product **5** consists of a highly congested tetracyclic fused ring cycle rather than the designed simple Schmidt reaction in scheme 1. The mechanism of this transformation was probed using deuterated product **2a-D** as substrate, which was synthesized from correspond **1a-D** in 78% isolated yield using our standard [4 + 4] conditions (see SI for detail). The fused tetracyclic product **5a-D** was successfully obtained in 64% yield indicating an unambiguous transannular 1,5-deuteryde transfer involved in the cascade skeleton rearrangement (Fig. 6). The dihydroquinoline moiety is a strong evidence of a cation-induced Schmidt rearrangement with NaN_3_ presumably after the hydride shift (detailed mechanism is provided vide infra).

**Fig. 7:**
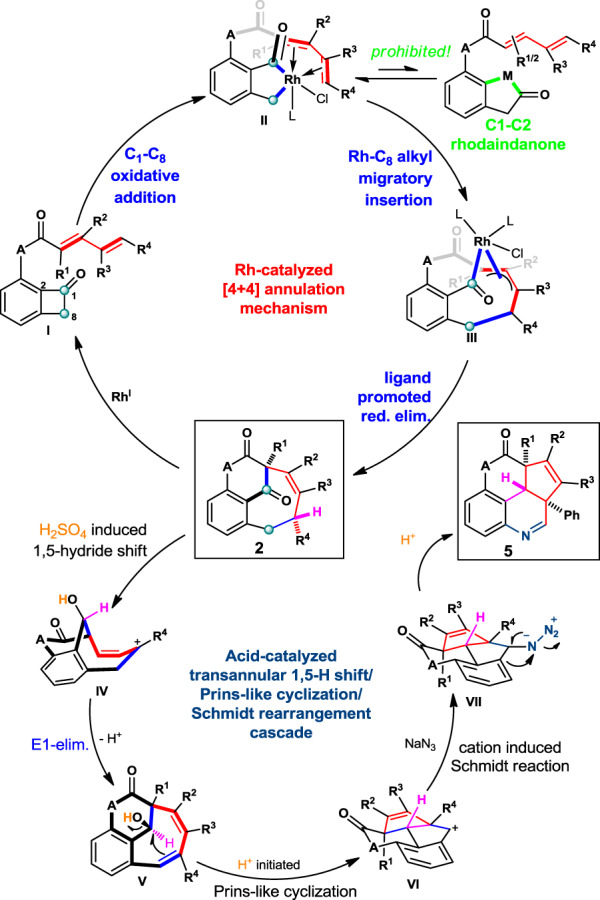
Proposed mechanism. The upper catalytic cycle is the proposed mechanism for Rh-catalyzed type-II [4 + 4] cycloaddition, while the lower is the proposed mechanism for acid-catalyzed cascade rearrangement.

Based on the previous report and the deuterium experiment, we tentatively proposed the catalytic cycle of the Rh-catalyzed [4 + 4] annulation between BCBs and pedant dienamides, together with the mechanism of the cascade skeleton rearrangement to form fused tetracycles from bridged bicyclo[5.3.1]undecane (Fig. 7, upper cycle). The C_1_–C_8_ oxidative addition with Rh^I^ complex took place first regioselectively, and the equilibration from **II** to the C_1_–C_2_ cleaved rhodaindanone (green) was competitively overrode by the regioselective migratory insertion into γ,δ-olefin due presumably to readily coordination with Rh-center. The resulting penta/hexa coordinated intermediate **II** was proposed to undergo a migratory insertion with the dienamide, yielding intermediate **III**. The Rh^III^ complex **III** will undergo reductive elimination thanks to the excess PPh_3_ ligand, providing the benzofused aza-bicyclo[5.3.1]undecandienone **2**.

**Fig. 8:**
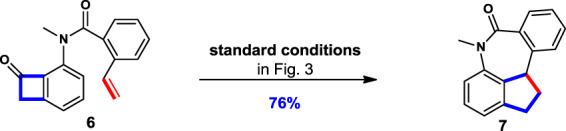
Control experiments. Substrate 6 was used to probe the mechanism of the migratory insertion step.

To gain insight for our hypothesis of alkyl C_8_-Rh undergoing migratory insertion rather than acyl C_1_-Rh into the distal olefin, compound **6** was designed, prepared, and subjected to the standard [4 + 4] conditions. A formal [4 + 2 − 1] annulated product **7** was obtained in 76% yield (Fig. 8). The formation of product **7** indicated that C_8_-Rh migratory inserted into the terminal olefins first, followed by decarbonylation and reductive elimination. Otherwise, the decarbonylation could not be realized if acyl C_1_-Rh migrated first.

In the cascade reaction (Fig. 7, lower cycle), the bridged carbonyl group activated by catalytic H_2_SO_4_ will initiated a through space 1,5-hydride transfer followed by a quick E1-type elimination from **IV**, affording hydride (pink) shifted intermediate **V**. The benzyl alcohol **V** will be protonated by the acid generating benzylic cation, which will induce a Prins-type cyclization obtaining **VI**. The intermediate **VI** will be trapped by NaN_3_ and trigger a Schmidt rearrangement yielding the final polyfused hydroquinoline-containing tetracycle **5**.

In summary, we have discovered a Rh-catalyzed type-II [4 + 4] annulation between BCBs and pedant dienamide constituting the first synthesis of the anti-Bredt bridgehead benzofused aza-bicyclo[5.3.1] ring system. The methodology featured low catalyst loading (2.5 mol%) and neutral reaction condition with broad substrate scope (23 examples) and high efficiency (up to 89% yield). The mechanism probing experiment supported that an unusual alkyl C_8_-Rh bond undergoes migratory insertion into distal olefin first. To further diversify the anti-Bredt bridged ring systems, a cascade transformation consisting of transannular 1,5-hydride shift, E1 elimination, Prins-like cyclization, and Schmidt rearrangement was discovered, generating polyfused tetracyclic ring systems. A deuterated experiment supported the 1,5-hydride transfer mechanism.

## Methods

### Procedure for rh-catalyzed type-II [4 + 4] annulation to make 2a

**Fig. 9:**
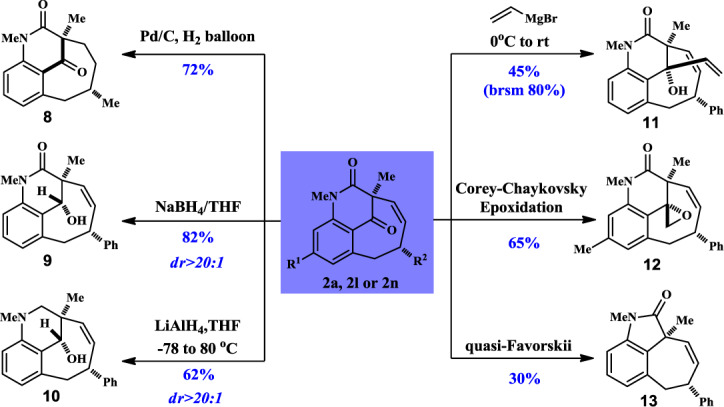
Applications of compound 2. Hydrogentation with Pd/C afforded **8**. Selective reduction can be achieved chemoselectively obtaining **9** and **10**. A 1,2-addition product **11** can be diastereoselectively obtained with vinylMgBr. Corey-Chaykovsky expoxidation provide **12** and quasi-Favorskii led to unexpected fused product **13**.

A 4 ml oven-dried vial was transferred in a N_2_-filled glove box and was charged with **1a** (40 mg, 0.126 mmol), [Rh(nbd)_2_Cl]_2_ (1.5 mg, 3.15 μmol), PPh_3_ (4.0 mg, 0.015 mmol), and 1,4-dioxane (3.7 ml) and the vial was capped. The vial was stirred at 150 °C for 96 h. Upon completion, it was cooled to room temperature. The solvent was removed by rotavap under reduced pressure and the residue was purified by flash chromatography to afford desired product **2a** (28.8 mg) in 72% yield and partial **1a** (4.9 mg) was recovered. The benzofused bicyclo[5.3.1] products can be further applied in a number of transformations (Fig. 9), Chemoselective reduction can be achieved by using different reagents, e.g., olefin could be hydrogenated obtaining saturated backbone **8** in 72% yield. Ketone and amide functional groups could be reduced to alcohol (**9** in 82% yield as a single isomer) and methylene (**10** in 60% yield), respectively, with different hydride reagents. In addition, product **2a** could underwent nucleophilic attack with vinyl Grignard reagent yielding **11** as a single diastereoisomer (45% yield). When a methylene sulfur ylide was used, an expoxidation product will be isolated in 65% from **2l**. A quasi-Favorskii reaction can be performed using aqueous NaOH, the fused tricycle product **13** was afforded in 30% yield.

## Supplementary information

Supplementary Information

## Data Availability

The data that support the findings of this study are available within the paper and its supplementary information files. Raw data are available from the corresponding author on reasonable request. Materials and methods, experimental procedures, characterization data, 1H, 13C, 19F NMR spectra, and mass spectrometry data are available in the Supplementary Information. The X-ray crystallographic coordinates for structures reported in this study have been deposited at the Cambridge Crystallographic Data Centre (CCDC), under deposition numbers CCDC 1873018 (**2a**), 1873019 (**2b**), 1873021 (**2j**), and 1873022 (**2n**). These data can be obtained free of charge from The Cambridge Crystallographic Data Centre via www.ccdc.cam.ac.uk/data_request/cif.
