# Supplementary material for: Regioselective activation of benzocyclobutenones and dienamides lead to anti-Bredt bridged-ring systems by a [4+4] cycloaddition
Source: Nat Commun. 2021 May 21;12:3022. doi: 10.1038/s41467-021-23344-0 (PMC8140143; doi:10.1038/s41467-021-23344-0)

Supplementary Information

Regioselective Activation of Benzocyclobutenones and Dienamides Lead to anti-Bredt Bridged-ring Systems by a [4+4] Cycloaddition.

Jianyu Zhang,[1] Xi Wang[1] and Tao Xu*[,1,2]

[1]Key Laboratory of Marine Drugs, Ministry of Education; School of Medicine and Pharmacy, Ocean University of China, 5 Yushan Road, Qingdao 266003.

[2]Laboratory for Marine Drugs and Bioproducts & Open Studio for Druggability Research of Marine Natural Products, Pilot National Laboratory for Marine Science and Technology, 1 Wenhai Road, Qingdao 266237, China.

Table of Contents

1. General information and Starting Materials **S2**

2. Experimental procedure and characterization data **S3**

3. References **S47**

4. X-Ray data for compounds **S48**

5. NMR Spectra **S54**

General information and Starting Materials.

General information. Unless otherwise mentioned, all manipulations were carried out under an inert atmosphere in a nitrogen-filled glovebox or by standard Schlenk techniques. The vials (1 dram, 15×45 mm with PTFE lined cap attached) were purchased from Devan Scientific and dried in an oven overnight and cooled under a steam of nitrogen prior to use. All reagents were purchased from commercial suppliers (like Energy Chemical, Bidepharm) without further purification. Solvent purification was conducted according to Purification of Laboratory Chemicals (Peerrin, D. D.; Armarego, W. L. and Perrins, D. R., Pergamon Press: Oxford, 1980). Yields refer to chromatographically and spectroscopically (^{1}H NMR) homogeneous materials. Reactions were monitored by Thin Layer Chromatography on plates (GF254) supplied by Yantai xinnuo Chemicals (China) visualized by UV or chemical stains. An oil bath was used as the heating source for reactions requiring heat. If not specially mentioned, flash column chromatography was performed using Yantai xinnuo Chemicals (China) (particle size 0.040 - 0.063 mm). The displacement ellipsoids are scaled to the 30% probability level for x-ray structure. NMR spectra were recorded on JEOL 400, Bruker AV500 instruments and calibrated by using residual undeuterated chloroform-*d* (δ H = 7.260 ppm, δ C = 77.160 ppm), dimethyl sulfoxide-d_6 (δ H = 2.500 ppm, δ C = 39.520 ppm), as internal references. The following abbreviations were used to explain the multiplicities: s = singlet, d = doublet, t = triplet, q = quartet, b = broad, td = triple doublet, dt = double triplet, dq = double quartet, m = multiplet. Infrared (IR) spectra were recorded on a BFRL WQF-530 FT-IR spectrometer. High-resolution mass spectra (HRMS) were recorded on a Thermo Fisher Scientific Exactive Quadruplole mass spectrometer using ESI (electrosprayionization) as ionization method.

Starting Materials. The compounds **S11** (Molmall sarl), **S23** (Zerenex), **S33**, **S37**, **S39** (Rarechem) was purchased from indicated commercial suppliers. The compounds **S1**,[3,5] **S2**,[3,5] **S4**,[3,5] **S13**,[7,8] **S15**,[7] **S17**,[7] **S19**,[9,14] **S21**,[9,14] **S25**,[11] **S27**,[10] **S29**,[13] **S30**,[13] **S31**,[12] **S35**,[10] **S38**,[11] **S39**,[11] **S40**,[10] **S42**[10] and **S46**[7] were prepared following literature reported procedure.

Experimental procedure and characterization data

Ⅰ. *General information about substrate synthesis*

The substrates were synthesized following the route shown below

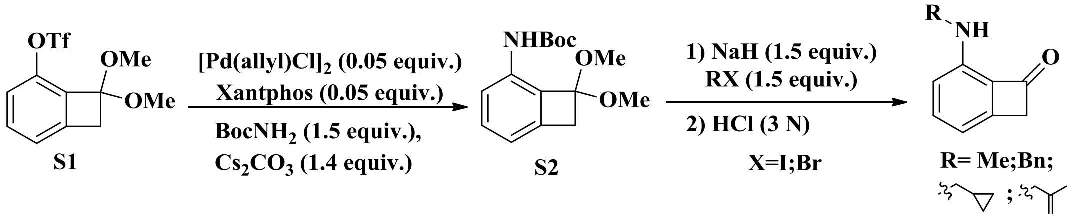

The procedure was modified from previous report. [1~6]

II. *Synthesis of intermediates and substrates*

a) *Synthesis of known compounds* **S1, S3** *and* **S4**:

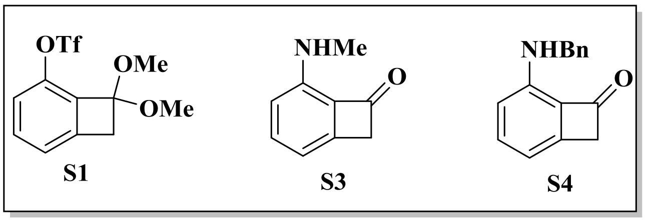

Compounds **S1, S3** and **S4** were synthesized according to the reported procedure, Their spectroscopic data match those reported in literature.[3,5]

b) *Synthesis of compounds* **S2, S5, S6, S7, S8, S9** *and* **S10**:

***tert*-butyl (8,8-dimethoxybicyclo[4.2.0]octa-1,3,5-trien-2-yl)carbamate (S2)**

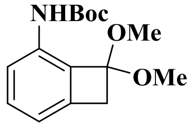

S2

To a 20 mL flamed-dried vial equipped with a stir bar was added **S1** (1.40 g, 1.0 equiv, 4.5 mmol), $BocNH_2$ (0.79 g, 1.5 equiv, 6.72 mmol), $[Pd(allyl)Cl]_2$ (0.08 g, 0.05 equiv, 0.224 mmol), Xantphos (0.12 g, 0.05 equiv, 0.224 mmol) and Cs_2CO_3 (2.04 g, 1.4 equiv, 6.27 mmol). Then the vial was loosely capped and transferred into a nitrogen-filled glovebox and 1,4-dioxane (10 mL) was added to the mixture before the vial was tightly capped and transferred out. The system was then heated to 100 ℃ overnight. Upon completion, the reaction was cooled to room temperature and filtered through a pad of celite. The combined organic phases were dried over $MgSO_4$ and the

solvent was removed under reduced pressure. The crude product was purified by using silica gel flash chromatography gave compound **S2** (1.11 g, 89%) as a yellow oil.

TLC (Hex : EA = 5:1): R_f = 0.6;

^{1}H NMR (400 MHz, Chloroform-*d*) δ 7.84 (d, J = 7.9 Hz, 1H), 7.29 - 7.24 (m, 1H), 6.86 (d, J = 7.2 Hz, 1H), 6.56 (s, 1H), 3.42 (s, 6H), 3.27 (s, 2H), 1.49 (s, 9H). **^{13}C NMR** (100 MHz, Chloroform-*d*) δ 187.3, 152.0, 150.4, 137.6, 133.9, 132.1, 117.2, 116.4, 81.7, 51.9, 50.9, 28.3. **IR**: ***v*** 2974, 2827, 1735, 1608, 1509, 1413, 1240, 1224, 1155, 1062, 974 cm^{-1}; **HRMS** (m/z): $[M-H]^-$ calcd. for $C_{15}H_{20}O_4N^-$, 278.1397; found, 278.1398.

((cyclopropylmethyl)amino)bicyclo[4.2.0]octa-1,3,5-trien-7-one (S5)

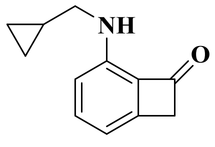

S5

A flamed-dried flask was charged with **S2** (1.20 g, 1.0 equiv, 4.3 mmol) and NaH (0.26 g, 1.5 equiv, 6.4 mmol). The flask was put under vacuum and backfilled with N_2 for three times. The commercial available bromomethyl cyclopropane (0.62 mL, 1.5 equiv, 6.4 mmol) was added to the mixture after the reaction was stirred at 0 ^oC for 15 min. The reaction was stirred at room temperature for 12 h. The reaction was quenched with MeOH and extracted with DCM (3 × 20 mL). The combined organic phases were dried over $MgSO_4$, and the solvent was removed under reduced pressure. The crude product was dissolved with MeOH and HCl (3 M). The mixture was stirred at room temperature for 12 h. The reaction was extracted with DCM (3 × 20 mL). The combined organic phrase was washed with brine (20.0 mL), dried over $MgSO_4$ and the solvent was removed under reduced pressure. The crude product was purified by using silica gel flash chromatography gave compound **S5** (0.52 g, 65%) as a white solid.

TLC (Hex : EA = 5:1): R_f = 0.5;

^{1}H NMR (400 MHz, Chloroform-*d*) δ 7.27 - 7.20 (m, 1H), 6.65 (d, J = 6.9 Hz, 1H),

6.39 (d, J = 8.4 Hz, 1H), 3.76 (s, 2H), 3.28 (d, J = 7.1 Hz, 2H), 1.05 (ddd, J = 12.5, 7.6, 4.9 Hz, 1H), 0.59 - 0.47 (m, 2H), 0.25 (q, J = 4.9 Hz, 2H). ^{13}**C NMR** (100 MHz, Chloroform-*d*) δ 186.7, 150.9, 142.8, 137.3, 129.2, 111.4, 110.2, 50.7, 49.7, 3.5. **IR**: ***v*** 3079, 3002, 1731, 1612, 1569, 1527, 1463, 1421, 1162, 1114, 1020 cm^{-1}; **HRMS** (m/z): $[M+H]^+$ calcd. for $C_{12}H_{14}ON^+$, 188.1069; found, 188.1065. **mp**: 85-86 ^{o}C.

5-((2-methylallyl)amino)bicyclo[4.2.0]octa-1,3,5-trien-7-one (S6)

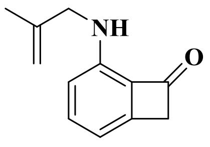

S6

S6 was synthesized following the same procedure for **S5** from **S2** (1.20 g, 1.0 equiv, 4.3 mmol) and 3-Bromo-2-methylpropene (0.65 ml, 1.5 equiv, 6.4 mmol). **S6** was obtained (0.49 g, 61%) as a white solid.

TLC (Hex : EA = 5:1): R_f = 0.5;

^{1}**H NMR** (400 MHz, Chloroform-*d*) δ 7.32 - 7.22 (m, 1H), 6.66 (d, J = 6.9 Hz, 1H), 6.42 (d, J = 8.4 Hz, 1H), 4.85 (d, J = 12.7 Hz, 2H), 4.00 (s, 2H), 3.76 (s, 2H), 1.77 (s, 3H). ^{13}**C NMR** (100 MHz, Chloroform-*d*) δ 186.6, 150.9, 143.0, 142.5, 137.3, 129.2, 111.6, 110.8, 110.6, 50.7, 50.5, 20.2. **IR**: ***v*** 3032, 2941, 1749, 1647, 1522, 1467, 1422, 1210, 1112, 1021, 991 cm^{-1}; **HRMS** (m/z): $[M+H]^+$ calcd. for $C_{12}H_{14}ON^+$, 188.1070; found, 188.1070. **mp**: 84-85 ^{o}C.

4-methyl-8-oxobicyclo[4.2.0]octa-1,3,5-trien-2-yl trifluoromethanesulfonate (S7)

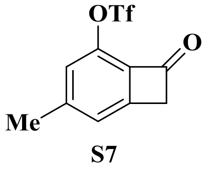

S7

Compound **S7** was synthesized according to the reported procedure[6]. To a solution of 5-hydroxy-3-methylbicyclo[4.2.0]octa-1,3,5-trien-7-one (1.90 g, 1.0 equiv, 12.8 mmol) in DCM (20 mL) at -78 ^{o}C was added Et_3N (3.55 mL, 2.0 equiv, 25.6 mmol), the reaction was stirred at this temperature for 30 min, Tf_2O (2.71 mL, 1.5 equiv, 19.2 mmol) was added, then the mixture was stirred at -78 ^{o}C for 1 h. Upon completion, the reaction was quenched with aqueous NH_4Cl (10 mL) and extracted with EtOAc (3

× 20 mL), washed with brine (30 mL), dried over anhydrous $MgSO_4$, concentrated and purificated with silica gel flash chromatography to afford **S7** (1.79 g, 50%) as a yellow oil.

TLC (Hex : EA = 5:1): R_f = 0.8;

^{1}H NMR (400 MHz, Chloroform-*d*) δ 7.36 (s, 1H), 7.04 (s, 1H), 4.00 (s, 2H), 2.49 (s, 3H). **^{13}C NMR** (100 MHz, Chloroform-*d*) δ 181.4, 152.4, 150.1, 137.5, 136.5, 124.5, 123.5, 122.3, 120.3, 117.2, 114.0, 52.9, 22.6. **^{19}F NMR** (376 MHz, Chloroform-*d*) δ -72.88. **IR**: ***v*** 3084, 2929, 1898, 1778, 1602, 1467, 1434, 1211, 1162, 1141, 1012 cm^{-1}; **HRMS** (m/z): [M-H]$^-$ calcd. for $C_{10}H_6O_4F_3S^-$, 278.9948; found, 278.9944.

8,8-dimethoxy-4-methylbicyclo[4.2.0]octa-1,3,5-trien-2-yl trifluoromethanesulfonate (S8)

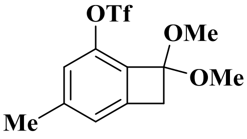

S8

S8 was synthesized following the same procedure for **S1** from **S7** (3.40 g, 1.0 equiv, 12.1 mmol), *p*-TsOH (3.12 g, 1.5 equiv, 18.2 mmol) and $H(OMe)_3$ (11.91 mL, 9.0 equiv, 108.9 mmol). **S8** was obtained (470 mg, 80%) as a yellow oil.

TLC (Hex : EA = 5:1): R_f = 0.7;

^{1}H NMR (400 MHz, Chloroform-*d*) δ 7.06 (s, 1H), 6.95 (s, 1H), 3.44 (s, 6H), 3.35 (s, 2H), 2.40 (s, 3H). **^{13}C NMR** (100 MHz, Chloroform-*d*) δ 144.7, 143.3, 140.5, 133.4, 124.9, 123.4, 120.2, 120.1, 117.0, 113.9, 105.0, 51.9, 43.3, 22.0. **^{19}F NMR** (376 MHz, Chloroform-*d*) δ -73.65. **IR**: ***v*** 2940, 2833, 1615, 1584, 1423, 1243, 1214, 1141, 1124, 1058, 1008 cm^{-1}; **HRMS** (m/z): [M-H]$^-$ calcd. for $C_{12}H_{12}O_5F_3S^-$, 325.0366; found, 325.0363.

***tert*-butyl (8,8-dimethoxy-4-methylbicyclo[4.2.0]octa-1,3,5-trien-2-yl)carbamate (S9)**

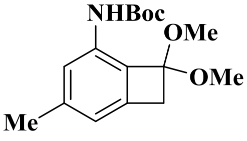

S9

S9 was synthesized following the same procedure for **S2** from **S8** (1.40 g, 1.0 equiv, 4.29 mmol). **S9** was obtained (1.07 g, 85%) as a yellow oil.

TLC (Hex : EA = 5:1): R_f = 0.6;

^{1}H NMR (400 MHz, Chloroform-*d*) δ 7.86 (s, 1H), 6.93 (s, 1H), 3.84 (s, 2H), 3.49 (s, 6H), 2.42 (s, 3H), 1.51 (s, 9H). **^{13}C NMR** (100 MHz, Chloroform-*d*) δ 186.7, 152.1, 150.5, 149.8, 132.0, 131.5, 118.1, 117.2, 81.6, 51.2, 50.9, 28.3, 23.2. **IR**: ***v*** 2933, 1720, 1654, 1496, 1438, 1412, 1390, 1255, 1159, 1101, 1062 cm^{-1}; **HRMS** (m/z): [M+Na]$^+$ calcd. for $C_{16}H_{23}NO_4Na^+$, 316.1511; found, 316.1519.

3-methyl-5-(methylamino)bicyclo[4.2.0]octa-1,3,5-trien-7-one (S10)

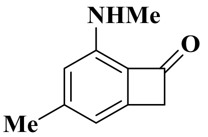

S10

S10 was synthesized following the same procedure for **S5** from **S9** (0.50 g, 1.0 equiv, 1.71 mmol). **S10** was obtained (0.19 g, 70%) as a faint yellow solid.

TLC (Hex : EA = 5:1): R_f = 0.5;

^{1}H NMR (400 MHz, Chloroform-*d*) δ 6.50 (s, 1H), 6.20 (s, 1H), 4.48 (s, 1H), 3.71 (s, 2H), 3.04 (s, 3H), 2.30 (s, 3H). **^{13}C NMR** (100 MHz, Chloroform-*d*) δ 186.0, 150.9, 149.0, 143.8, 127.3, 111.7, 111.4, 50.1, 31.5, 22.9. **IR**: ***v*** 3022, 2943, 1741, 1638, 1610, 1574, 1422, 1401, 1254, 1210, 1103 cm^{-1}; **HRMS** (m/z): [M+H]$^+$ calcd. for $C_{10}H_{12}NO^+$, 162.0912; found, 162.0913. **mp**: 116-117 ^{o}C.

<u>General procedure Ⅰ for compounds (1a~1m, 1p~1r, 1v~1a-D, 6)</u>

Oxalyl chloride (3.6 equiv) was added dropwise to a solution of acid (3.0 equiv) in dried DCM, then DMF (one drop) was added to the mixture. The solution was stirred for 1 h at room temperature, solvents were removed in vacuo to obtain acyl chloride. To a 50-mL flamed-dried flasks equipped with a stir bar and a nitrogen-filled balloon was added amine (1.0 equiv) and dissolved by dried DCM. NaHMDS (1.5 equiv, 2.0 mol/L in THF) was added dropwise to the mixture and stirred for 20 min at -78 ^{o}C. Followed by addition of acyl chloride. The reaction was warmed to rt and stirred overnight. The reaction was quenched by saturated NH_4Cl aqueous solution then

extracted with EtOAc, the combined organic extracts were washed with a saturated solution of brine, dried over $MgSO_4$, and evaporated *in vacuo*. The residue was purified by flash chromatography to afford the desired compound.

General procedure Ⅱ for the synthesis of compounds (1n~1o)

DIPEA (4.0 equiv) was added to a solution of acid (2.0 equiv), amine (1.0 equiv) in MeCN at 0 °C, the solution was stirred for 5 min. BOPCl (1.5 equiv) was added to the mixture at 0 °C. Then the mixture was heated to 80 °C and stirred overnight. After the starting material was consumed, the reaction mixture was cooled to rt. The reaction was quenched by saturated NH_4Cl aqueous solution and extracted with EtOAc, the combined organic layer was dried over $MgSO_4$, and evaporated in vacuo. The residue was purified by flash chromatography to afford the desired compound.

General procedure Ⅲ for the synthesis of compounds (1s~1u)

Methylimidazole (2.1 equiv, NMI) was added to a solution of acid (1.0 equiv), amine (1.2 equiv) in MeCN at rt, then Chloro-N,N,N',N'-tetramethylformamidinium Hexafluorophosphate (1.1 equiv, TCFH) was added to mixture and stirred overnight. After the starting material was consumed, the reaction was quenched by saturated NH_4Cl aqueous solution and extracted with DCM, the combined organic layer was dried over $MgSO_4$, and evaporated in vacuo. The residue was purified by flash chromatography to afford the desired compound.[7]

(2*E*,4*E*)-*N*,2-dimethyl-*N*-(8-oxobicyclo[4.2.0]octa-1(6),2,4-trien-2-yl)-5-phenylpenta-2,4-dienamide (1a)

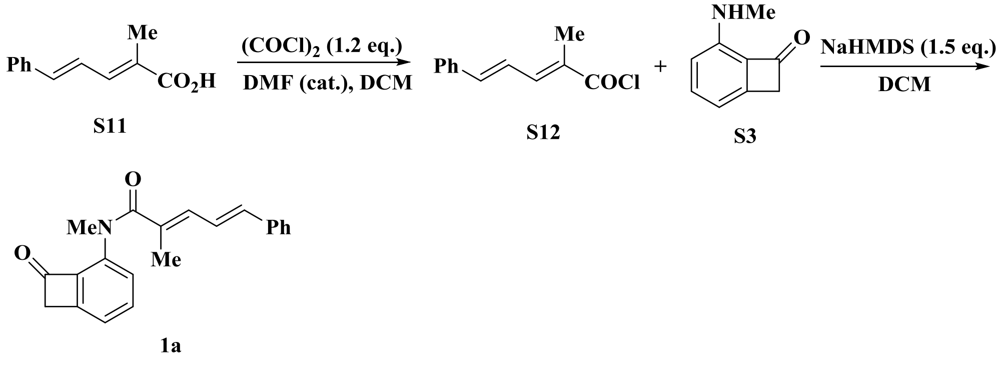

1a

1a was obtained (78 mg, 72% yield, *E*/*Z* = 96:4) as a light yellow oil following the **general procedure** Ⅰ from **S11** (191 mg, 1.01 mmol) and **S3** (50 mg, 0.34 mmol).

TLC (Hex : EA = 5:1): R_f = 0.4;

^{1}H NMR (400 MHz, Chloroform-*d*) δ 7.42 (d, J = 8.0 Hz, 1H), 7.38 (d, J = 8.3 Hz, 2H), 7.30 (t, J = 7.4 Hz, 2H), 7.24 (dd, J = 7.1, 1.8 Hz, 2H), 7.10 (d, J = 8.1 Hz, 1H), 6.87 (dd, J = 15.5, 11.2 Hz, 1H), 6.55 (d, J = 15.5 Hz, 1H), 6.50 - 6.43 (m, 1H), 3.97 (s, 2H), 3.52 (s, 3H), 1.91 (s, 3H). **^{13}C NMR** (100 MHz, Chloroform-*d*) δ 185.3, 172.9, 151.6, 139.4, 137.3, 137.0, 136.7, 136.2, 135.5, 131.9, 128.8, 128.5, 126.9, 124.0, 123.5, 119.9, 51.9, 37.3, 14.8. **IR**: ***v*** 3031, 2934, 1770, 1644, 1598, 1522, 1474, 1429, 1350, 1231, 1141 cm^{-1}; **HRMS** (m/z): $[M+H]^+$ calcd. for $C_{21}H_{20}NO_2^+$, 318.1490; found, 318.1489.

(2*E*,4*E*)-5-(4-fluorophenyl)-*N*,2-dimethyl-*N*-(8-oxobicyclo[4.2.0]octa-1(6),2,4-trien-2-yl)penta-2,4-dienamide (1b)

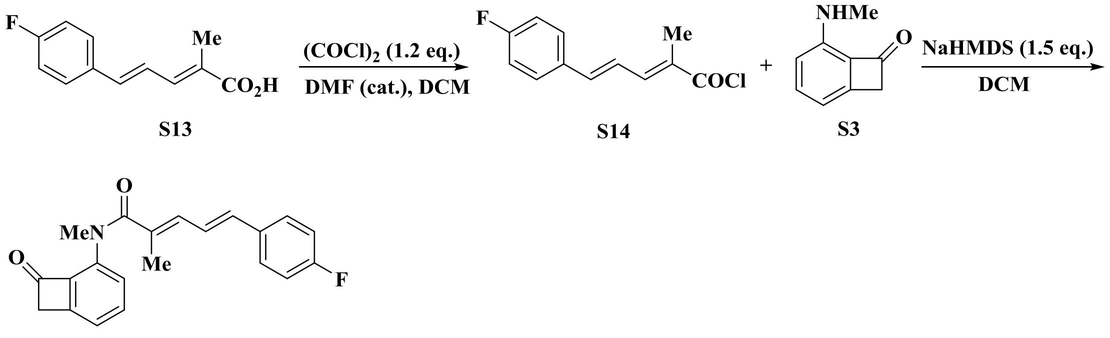

1b was obtained (72 mg, 63% yield, *E*/*Z* = 96:4) as a yellow solid following the **general procedure** Ⅰ from **S13** (210 mg, 1.02 mmol) and **S3** (50 mg, 0.34 mmol).

TLC (Hex : EA = 5:1): R_f = 0.4;

^{1}H NMR (400 MHz, Chloroform-*d*) δ 7.44 - 7.39 (m, 1H), 7.35 (dd, J = 8.6, 5.5 Hz, 2H), 7.25 (d, J = 7.2 Hz, 1H), 7.09 (d, J = 8.1 Hz, 1H), 6.99 (t, J = 8.6 Hz, 2H), 6.78 (dd, J = 15.4, 11.1 Hz, 1H), 6.51 (d, J = 15.5 Hz, 1H), 6.47 - 6.42 (m, 1H), 3.97 (s, 2H), 3.52 (s, 3H), 1.90 (s, 3H). **^{13}C NMR** (100 MHz, Chloroform-*d*) δ 185.4, 172.9, 151.6, 139.4, 137.3, 136.2, 135.6, 135.3, 133.0, 132.0, 128.5, 128.4, 124.0, 123.3, 119.9, 115.9, 51.9, 37.3, 14.8. **^{19}F NMR** (376 MHz, Chloroform-*d*) δ -113.29, -112.58. **IR**: ***v*** 3039, 2920, 1762, 1648, 1596, 1508, 1477, 1429, 1346, 1230, 1159 cm^{-1}; **HRMS** (m/z): $[M+H]^+$ calcd. for $C_{21}H_{19}O_2NF^+$, 336.1391; found, 336.1394. **mp**: 53-54 ^{o}C.

(2*E*,4*E*)-5-(4-chlorophenyl)-*N*,2-dimethyl-*N*-(8-oxobicyclo[4.2.0]octa-1(6),2,4-trien-2-yl)penta-2,4-dienamide (1c)

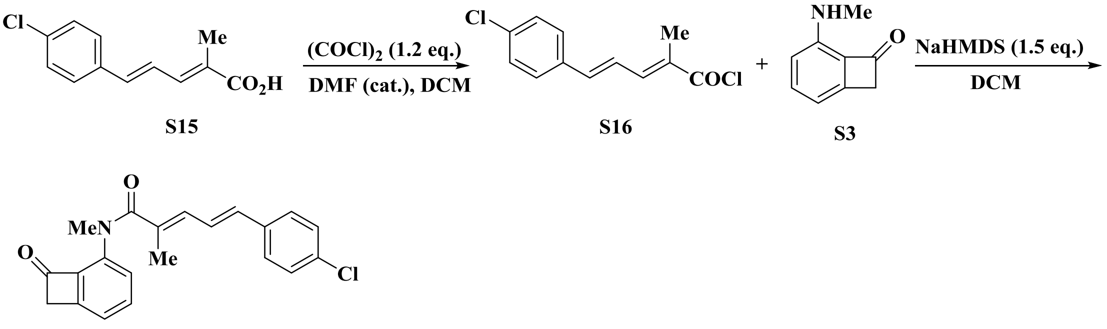

1c was obtained (72 mg, 60% yield, *E*/*Z* = 97:3) as a yellow solid following the **general procedure** Ⅰ from **S15** (226 mg, 1.01 mmol) and **S3** (50 mg, 0.34 mmol). TLC (Hex : EA = 5:1): R_f = 0.4;

^{1}H NMR (400 MHz, Chloroform-*d*) δ 7.41 (t, J = 7.7 Hz, 1H), 7.30 (d, J = 8.6 Hz, 2H), 7.25 (d, J = 8.6 Hz, 3H), 7.09 (d, J = 8.1 Hz, 1H), 6.83 (dd, J = 15.4, 11.2 Hz, 1H), 6.56 - 6.39 (m, 2H), 3.96 (s, 2H), 3.51 (s, 3H), 1.90 (s, 3H). **^{13}C NMR** (100 MHz, Chloroform-*d*) δ 185.3, 172.8, 151.6, 139.5, 137.2, 136.2, 135.5, 135.3, 135.0, 134.1, 132.6, 129.0, 128.0, 124.1, 124.0, 120.0, 51.9, 37.3, 14.8. **IR**: ***v*** 3434, 3038, 2920, 1754, 1639, 1540, 1477, 1223, 1008, 1000, 740 cm^{-1}; **HRMS** (m/z): $[M+H]^+$ calcd. for $C_{21}H_{19}ClNO_2^+$, 352.1100; found, 352.1100. **mp**: 64-65 ^oC.

(2*E*,4*E*)-5-(4-methoxyphenyl)-*N*,2-dimethyl-*N*-(8-oxobicyclo[4.2.0]octa-1(6),2,4-trien-2-yl)penta-2,4-dienamide (1d)

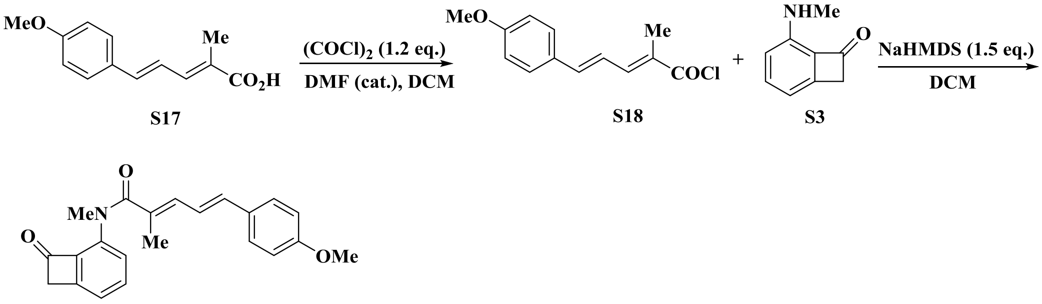

1d was obtained (77 mg, 65% yield, *E*/*Z* = 99:1) as a colorless oil following the **general procedure** Ⅰ from **S17** (226 mg, 1.04 mmol) and **S3** (50 mg, 0.34 mmol). TLC (Hex : EA = 5:1): R_f = 0.35;

^{1}H NMR (400 MHz, Chloroform-*d*) δ 7.40 (t, J = 7.7 Hz, 1H), 7.32 (d, J = 8.7 Hz,

2H), 7.25 - 7.21 (m, 1H), 7.09 (d, J = 8.2 Hz, 1H), 6.83 (d, J = 8.7 Hz, 2H), 6.73 (dd, J = 15.3, 11.2 Hz, 1H), 6.54 - 6.40 (m, 2H), 3.96 (s, 2H), 3.79 (s, 3H), 3.51 (s, 3H), 1.88 (s, 3H). **^{13}C NMR** (100 MHz, Chloroform-*d*) δ 185.4, 173.2, 160.0, 151.5, 139.3, 137.5, 136.7, 136.2, 136.1, 130.6, 129.6, 128.3, 123.9, 121.6, 119.7, 114.3, 55.4, 51.8, 37.3, 14.7. **IR**: ***v*** 3036, 3002, 2926, 2838, 1766, 1644, 1598, 1509, 1479, 1346, 1245 cm^{-1}; **HRMS** (m/z): $[M+H]^+$ calcd. for $C_{22}H_{22}NO_3^+$, 348.1591; found, 348.1594.

(2*E*,4*E*)-2-ethyl-*N*-methyl-*N*-(8-oxobicyclo[4.2.0]octa-1(6),2,4-trien-2-yl)-5-phenyl penta-2,4-dienamide (1e)

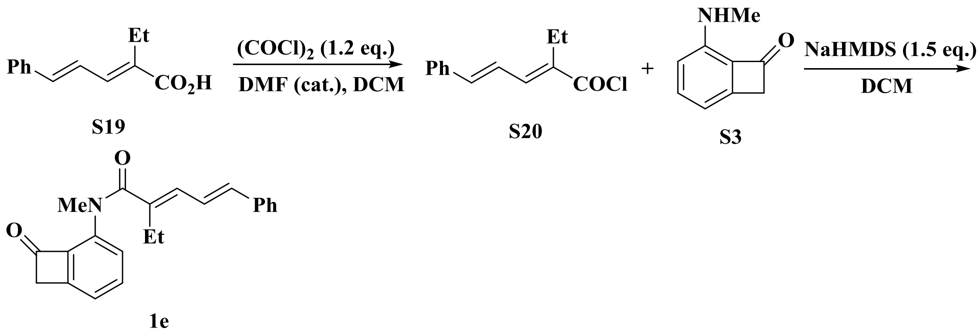

1e was obtained (72 mg, 64% yield, *E*/*Z* = 96:4) as a colorless oil following the **general procedure** Ⅰ from **S19** (206 mg, 1.02 mmol) and **S3** (50 mg, 0.34 mmol). TLC (Hex : EA = 5:1): R_f = 0.4;

^{1}H NMR (400 MHz, Chloroform-*d*) δ 7.43 - 7.37 (m, 3H), 7.31 (t, J = 7.4 Hz, 2H), 7.25 (d, J = 6.2 Hz, 2H), 7.21 (d, J = 8.3 Hz, 1H), 6.91 (dd, J = 15.4, 11.2 Hz, 1H), 6.55 (d, J = 15.5 Hz, 1H), 6.37 (d, J = 11.2 Hz, 1H), 3.98 (s, 2H), 3.54 (s, 3H), 2.39 (q, J = 7.5 Hz, 2H), 1.12 (t, J = 7.6 Hz, 3H). **^{13}C NMR** (100 MHz, Chloroform-*d*) δ 185.4, 172.6, 151.5, 139.3, 138.2, 137.2, 136.9, 136.8, 136.1, 134.3, 128.8, 128.5, 126.9, 124.3, 123.3, 119.8, 51.8, 37.5, 22.3, 13.9. **IR**: ***v*** 3036, 2963, 2929, 2875, 1762, 1648, 1596, 1477, 1448, 1346, 1230 cm^{-1}; **HRMS** (m/z): $[M+H]^+$ calcd. for $C_{22}H_{22}NO_2^+$, 332.1640; found, 332.1645.

(2*E*,4*E*)-2-(cyclopropylmethyl)-*N*-methyl-*N*-(8-oxobicyclo[4.2.0]octa-1(6),2,4-trie n-2-yl)-5-phenylpenta-2,4-dienamide (1f)

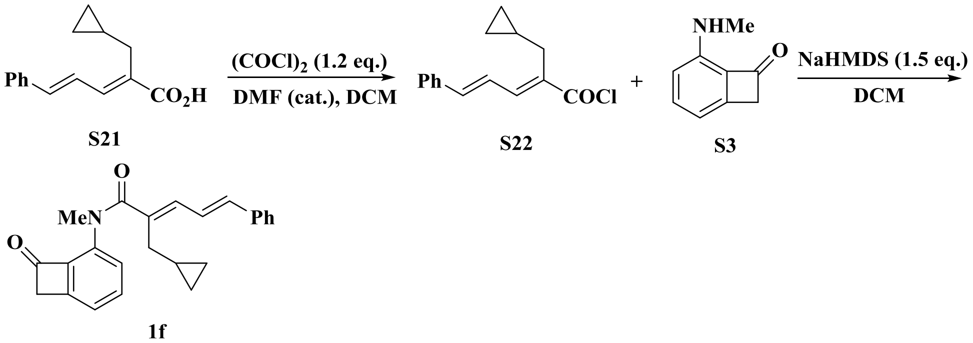

1f was obtained (52 mg, 43% yield, *E*/*Z* = 96:4) as a colorless oil following the **general procedure Ⅰ** from **S21** (232 mg, 1.02 mmol) and **S3** (50 mg, 0.34 mmol).

TLC (Hex : EA = 5:1): R_f = 0.5;

^{1}H NMR (400 MHz, Chloroform-*d*) δ 7.40 - 7.36 (m, 3H), 7.30 (d, J = 7.7 Hz, 2H), 7.28 - 7.19 (m, 3H), 6.92 (dd, J = 15.4, 11.2 Hz, 1H), 6.55 (d, J = 15.4 Hz, 1H), 6.36 (d, J = 11.1 Hz, 1H), 3.98 (s, 2H), 3.57 (s, 3H), 2.35 (d, J = 6.9 Hz, 2H), 0.89 - 0.83 (m, 1H), 0.52 (d, J = 7.8 Hz, 2H), 0.19 (d, J = 4.9 Hz, 2H). **^{13}C NMR** (100 MHz, Chloroform-*d*) δ 185.5, 172.9, 151.4, 139.1, 137.0, 136.5, 136.0, 134.4, 128.8, 128.5, 126.9, 124.5, 123.3, 119.7, 51.8, 37.7, 33.4, 10.5, 5.0. **IR**: ***ν*** 3073, 2999, 2917, 2847, 1762, 1646, 1594, 1477, 1340, 1225, 1130 cm^{-1}; **HRMS** (m/z): $[M+H]^+$ calcd. for $C_{24}H_{24}NO_2^+$, 358.1800; found, 358.1801.

(2*E*,4*E*)-*N*,3-dimethyl-*N*-(8-oxobicyclo[4.2.0]octa-1(6),2,4-trien-2-yl)-5-phenylpenta-2,4-dienamide (1g)

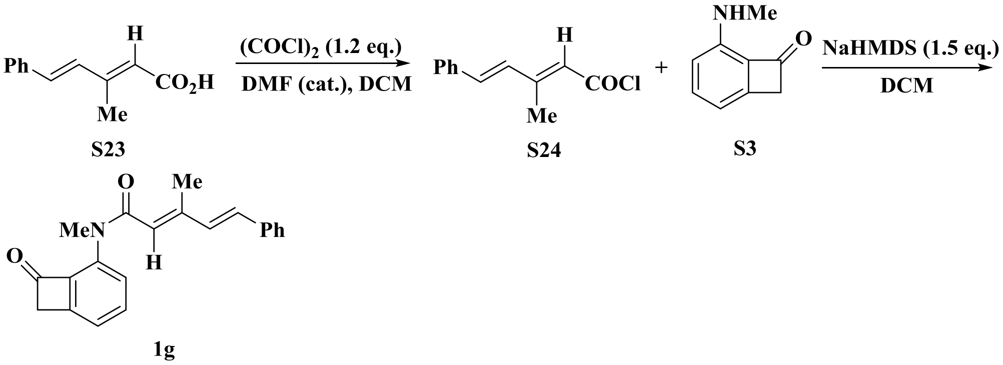

1g was obtained (72 mg, 67% yield, *E*/*Z* = 96:4) as a colorless oil following the **general procedure Ⅰ** from **S23** (191 mg, 1.01 mmol) and **S3** (50 mg, 0.34 mmol).

TLC (Hex : EA = 5:1): R_f = 0.4;

^{1}H NMR (400 MHz, Chloroform-*d*) δ 8.11 (d, J = 16.3 Hz, 1H), 7.51 (d, J = 7.3 Hz,

2H), 7.46 (t, J = 7.7 Hz, 1H), 7.34 - 7.28 (m, 3H), 7.25 - 7.20 (m, 2H), 6.83 (d, J = 16.3 Hz, 1H), 5.80 (s, 1H), 3.94 (s, 2H), 3.52 (s, 3H), 2.01 (s, 3H). **^{13}C NMR** (100 MHz, Chloroform-*d*) δ 185.2, 166.8, 151.6, 147.0, 140.1, 136.9, 136.5, 136.0, 134.4, 128.7, 128.5, 127.4, 126.6, 125.1, 120.5, 120.3, 51.9, 36.4, 20.9. **IR**: ***v*** 3059, 3022, 2917, 1760, 1644, 1592, 1475, 1332, 1294, 1139, 1012 cm^{-1}; **HRMS** (m/z): [M+Na]$^+$ calcd. for $C_{21}H_{19}NO_2Na^+$, 340.1308; found, 340.1308.

(2*Z*,4*E*)-*N*-methyl-*N*-(8-oxobicyclo[4.2.0]octa-1(6),2,4-trien-2-yl)-3,5-diphenylpenta-2,4-dienamide (1h)

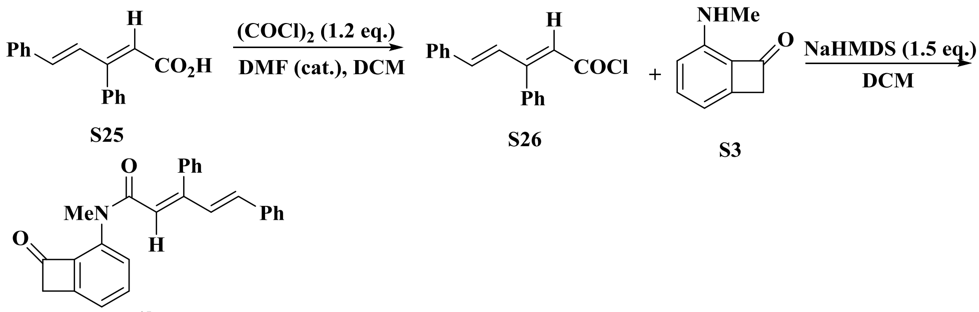

1h was obtained (55 mg, 43% yield, *E*/*Z* = 95:5) as a colorless oil following the **general procedure Ⅰ** from **S25** (255 mg, 1.02 mmol) and **S3** (50 mg, 0.34 mmol). TLC (Hex : EA = 5:1): R_f = 0.45;

^{1}H NMR (400 MHz, Chloroform-*d*) δ 8.22 (d, J = 17.0 Hz, 1H), 7.51 - 7.42 (m, 4H), 7.35 (dd, J = 6.6, 3.3 Hz, 4H), 7.31 (d, J = 7.1 Hz, 2H), 7.27 - 7.24 (m, 3H), 6.51 (d, J = 16.3 Hz, 1H), 5.85 (s, 1H), 3.92 (s, 2H), 3.56 (s, 3H). **^{13}C NMR** (100 MHz, Chloroform-*d*) δ 185.2, 166.6, 152.1, 151.6, 140.3, 138.5, 136.8, 136.3, 136.0, 129.1, 128.9, 128.7, 128.6, 128.4, 127.9, 127.5, 126.4, 125.1, 120.7, 120.5, 51.9, 36.5. **IR**: ***v*** 3436, 3023, 2947, 2440, 2387, 1772, 1637, 1488, 1297, 1139 ,1110 cm^{-1}; **HRMS** (m/z): [M+H]$^+$ calcd. for $C_{26}H_{22}NO_2^+$, 380.1646; found, 380.1645.

(2*E*,4*E*)-*N*-benzyl-2-methyl-*N*-(8-oxobicyclo[4.2.0]octa-1(6),2,4-trien-2-yl)-5-phenylpenta-2,4-dienamide (1i)

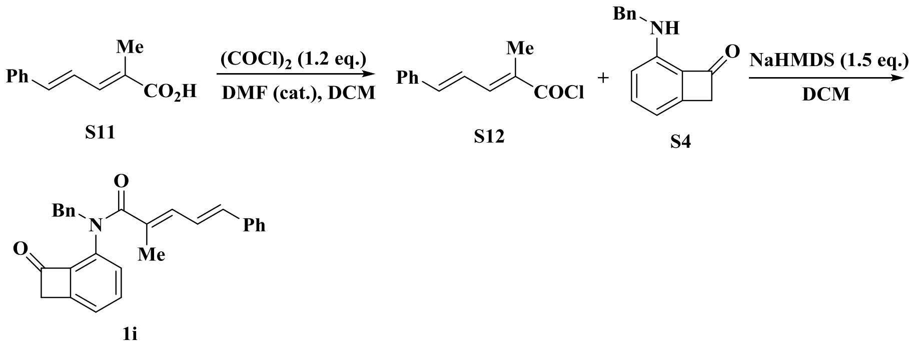

1i was obtained (55 mg, 62% yield, *E*/*Z* = 98:2) as a colorless oil following the **general procedure** Ⅰ from **S11** (126 mg, 0.67 mmol) and **S4** (50 mg, 0.224 mmol). TLC (Hex : EA = 5:1): R_f = 0.45;

^{1}H NMR (400 MHz, Chloroform-*d*) δ 7.40 - 7.36 (m, 2H), 7.34 - 7.28 (m, 3H), 7.23 - 7.19 (m, 5H), 7.19 - 7.13 (m, 2H), 6.98 (d, J = 8.1 Hz, 1H), 6.86 (dd, J = 15.5, 11.1 Hz, 1H), 6.62 - 6.44 (m, 2H), 5.30 (s, 2H), 3.93 (s, 2H), 1.91 (s, 3H). **^{13}C NMR** (100 MHz, Chloroform-*d*) δ 185.5, 173.0, 151.5, 140.0, 137.4, 137.1, 136.7, 136.3, 136.1, 131.8, 128.8, 128.5, 127.8, 127.3, 126.9, 125.2, 123.6, 120.2, 52.2, 51.9, 14.9. **IR**: ***v*** 3033, 2956, 2923, 2852, 1762, 1646, 1594, 1475, 1454, 1369, 1294 cm^{-1}; **HRMS** (m/z): $[M+H]^+$ calcd. for $C_{27}H_{24}NO_2^+$, 394.1802; found, 394.1802.

(2*E*,4*E*)-*N*-(cyclopropylmethyl)-2-methyl-*N*-(8-oxobicyclo[4.2.0]octa-1(6),2,4-trien-2-yl)-5-phenylpenta-2,4-dienamide (1j)

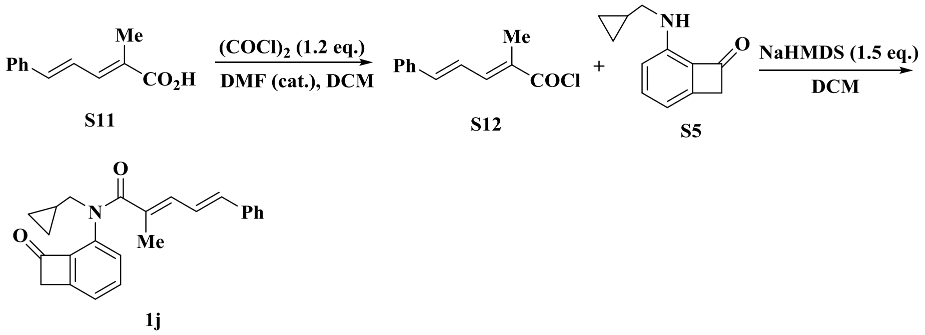

1j was obtained (72 mg, 76% yield, *E*/*Z* = 98:2) as a colorless oil following the **general procedure** Ⅰ from **S11** (150 mg, 0.8 mmol) and **S5** (50 mg, 0.267 mmol). TLC (Hex : EA = 5:1): R_f = 0.5;

^{1}H NMR (400 MHz, Chloroform-*d*) δ 7.44 (t, J = 7.7 Hz, 1H), 7.38 (d, J = 7.5 Hz, 2H), 7.33 - 7.27 (m, 3H), 7.23 (d, J = 7.0 Hz, 1H), 7.08 (d, J = 8.1 Hz, 1H), 6.84 (dd, J

= 15.4, 11.2 Hz, 1H), 6.50 (dd, J = 21.8, 13.3 Hz, 2H), 3.98 (s, 2H), 3.90 (d, J = 7.1 Hz, 2H), 1.86 (s, 3H), 1.02 (tt, J = 7.7, 4.9 Hz, 1H), 0.37 (q, J = 5.3 Hz, 2H), 0.16 (q, J = 4.9 Hz, 2H). **^{13}C NMR** (100 MHz, Chloroform-*d*) δ 185.5, 172.8, 151.7, 140.5, 136.8, 136.3, 135.5, 132.3, 128.8, 128.4, 126.8, 125.7, 123.6, 120.3, 53.6, 52.0, 14.8, 10.3, 3.7. **IR**: ***v*** 3467, 3400, 2400, 2387, 2350, 1759, 1639, 1575, 1474, 1369, 1204 cm^{-1}; **HRMS** (m/z): $[M+H]^+$ calcd. for $C_{24}H_{24}NO_2^+$, 358.1802; found, 358.1802.

(2*E*,4*E*)-2-methyl-*N*-(2-methylallyl)-*N*-(8-oxobicyclo[4.2.0]octa-1(6),2,4-trien-2-yl)-5-phenylpenta-2,4-dienamide (1k)

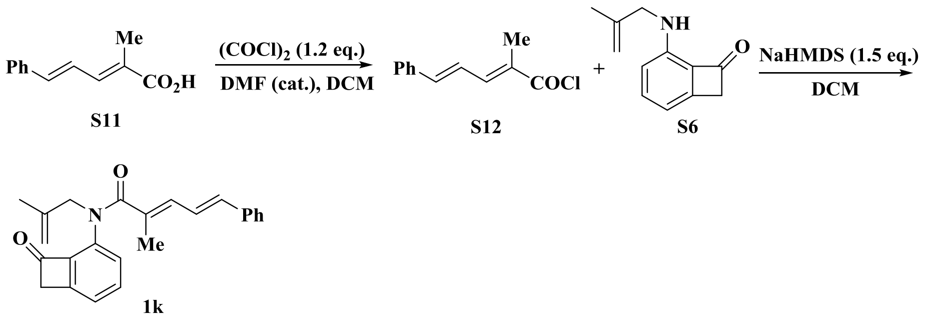

1k was obtained (56 mg, 59% yield, *E*/*Z* = 96:4) as a colorless oil following the **general procedure** Ⅰ from **S11** (150 mg, 0.8 mmol) and **S6** (50 mg, 0.27 mmol).
TLC (Hex : EA = 5:1): R_f = 0.55;
^{1}H NMR (400 MHz, Chloroform-*d*) δ 7.46 - 7.37 (m, 3H), 7.31 (dd, J = 8.1, 6.6 Hz, 3H), 7.25 - 7.22 (m, 1H), 7.12 (d, J = 7.8 Hz, 1H), 6.88 (dd, J = 15.4, 11.2 Hz, 1H), 6.53 (t, J = 12.7 Hz, 2H), 4.76 (d, J = 10.8 Hz, 2H), 4.67 (s, 2H), 3.95 (s, 2H), 1.92 (s, 3H), 1.69 (s, 3H). **^{13}C NMR** (100 MHz, Chloroform-*d*) δ 185.5, 172.8, 151.4, 141.0, 139.9, 137.0, 136.7, 136.3, 136.0, 135.6, 131.9, 128.8, 128.5, 126.9, 124.7, 123.6, 120.0, 112.4, 54.0, 51.8, 20.3, 14.8. **IR**: ***v*** 2923, 2847, 1759, 1646, 1590, 1477, 1367, 1225, 1163, 1135, 966 cm^{-1}; **HRMS** (m/z): $[M+H]^+$ calcd. for $C_{24}H_{24}NO_2^+$, 358.1799; found, 358.1802.

(2*E*,4*E*)-*N*,2-dimethyl-*N*-(4-methyl-8-oxobicyclo[4.2.0]octa-1(6),2,4-trien-2-yl)-5-phenylpenta-2,4-dienamide (1l)

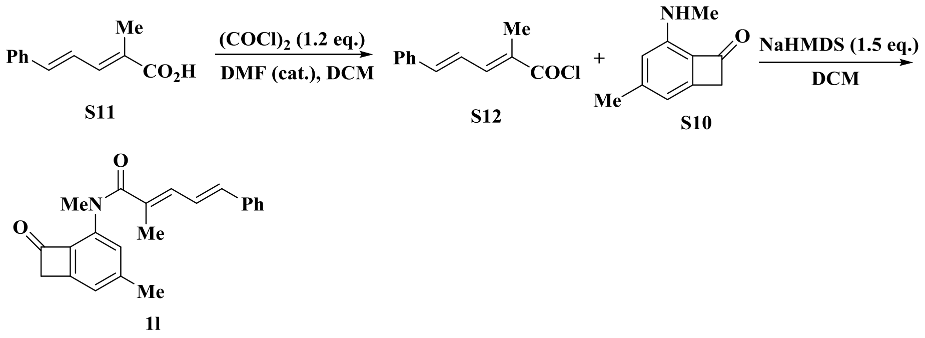

1l was obtained (77 mg, 75% yield, *E*/*Z* = 96:4) as a faint white oil following the **general procedure Ⅰ** from **S11** (175 mg, 0.93 mmol) and **S10** (50 mg, 0.31 mmol). TLC (Hex : EA = 5:1): R_f = 0.4;

^{1}H NMR (400 MHz, Chloroform-*d*) δ 7.39 (d, J = 7.3 Hz, 2H), 7.30 (t, J = 7.5 Hz, 3H), 7.24 (d, J = 6.0 Hz, 1H), 6.94 (s, 1H), 6.88 (dd, J = 15.4, 11.1 Hz, 1H), 6.56 (d, J = 15.5 Hz, 1H), 6.47 (d, J = 11.2 Hz, 1H), 3.89 (s, 2H), 3.50 (s, 3H), 2.36 (s, 3H), 1.92 (s, 3H). **^{13}C NMR** (100 MHz, Chloroform-*d*) δ 184.8, 173.0, 151.6, 148.1, 137.0, 136.9, 136.8, 135.2, 132.0, 128.8, 128.7, 128.4, 126.9, 125.0, 123.5, 120.7, 51.3, 37.4, 22.8, 14.8. **IR**: ***v*** 3056, 3028, 2920, 1754, 1646, 1596, 1473, 1361, 1311, 1194, 1106 cm^{-1}; **HRMS** (m/z): $[M+H]^+$ calcd. for $C_{22}H_{22}NO_2^+$, 332.1645; found, 332.1645.

(2*E*,4*E*)-*N*,2,4-trimethyl-*N*-(8-oxobicyclo[4.2.0]octa-1(6),2,4-trien-2-yl)-5-phenylpenta-2,4-dienamide (1m)

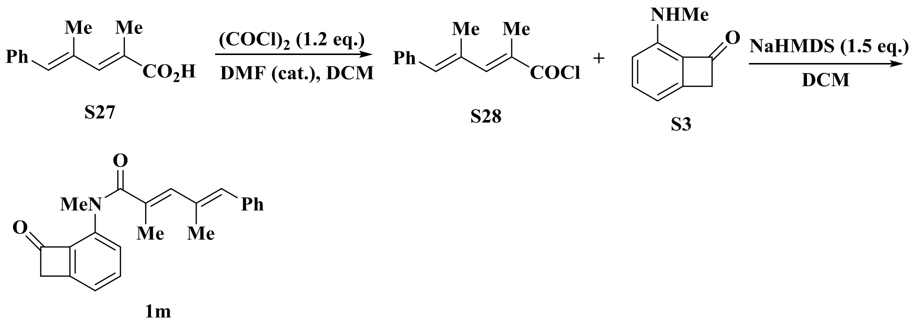

1m was obtained (62 mg, 55% yield, *E*/*Z* = 96:4) as a faint white oil following the **general procedure Ⅰ** from **S27** (206 mg, 1.02 mmol) and **S3** (50 mg, 0.34 mmol). TLC (Hex : EA = 5:1): R_f = 0.4;

^{1}H NMR (400 MHz, Chloroform-*d*) δ 7.48 - 7.43 (m, 1H), 7.34 - 7.26 (m, 3H), 7.23 (t, J = 6.8 Hz, 3H), 7.17 (d, J = 8.2 Hz, 1H), 6.30 (s, 1H), 6.22 (s, 1H), 3.97 (s, 2H), 3.53

(s, 3H), 1.98 (s, 3H), 1.90 (s, 3H). **^{13}C NMR** (100 MHz, Chloroform-*d*) δ 185.4, 173.6, 151.5, 139.6, 139.2, 137.3, 137.1, 136.1, 133.9, 132.4, 131.3, 129.2, 128.3, 127.1, 124.3, 120.0, 51.9, 37.2, 18.2, 16.2. **IR**: ***v*** 3022, 2920, 2858, 1764, 1650, 1596, 1477, 1351, 1231, 1172, 1124 cm^{-1}; **HRMS** (m/z): $[M+H]^+$ calcd. for $C_{22}H_{22}NO_2^+$, 332.1646; found, 332.1645.

(2*E*,4*E*)-*N*,2-dimethyl-*N*-(8-oxobicyclo[4.2.0]octa-1(6),2,4-trien-2-yl)hexa-2,4-dien amide (1n)

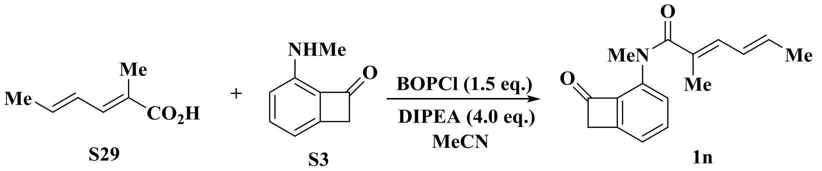

1n was obtained (31 mg, 36% yield, *E*/*Z* = 98:2) as a colorless oil following the **general procedure** Ⅱ from **S29** (86 mg, 0.68 mmol) and **S3** (50 mg, 0.34 mmol). TLC (Hex : EA = 5:1): R_f = 0.4;

^{1}H NMR (400 MHz, Chloroform-*d*) δ 7.45 - 7.32 (m, 1H), 7.22 (d, J = 7.2 Hz, 1H), 7.05 (d, J = 8.2 Hz, 1H), 6.25 (d, J = 11.1 Hz, 1H), 6.21 - 6.10 (m, 1H), 5.74 (dd, J = 14.6, 6.7 Hz, 1H), 3.94 (s, 2H), 3.47 (s, 3H), 1.76 (s, 3H). **^{13}C NMR** (100 MHz, Chloroform-*d*) δ 185.4, 173.3, 151.4, 139.3, 137.5, 136.1, 135.7, 135.3, 129.0, 126.8, 124.0, 119.6, 51.8, 37.2, 18.8, 14.4. **IR**: ***v*** 3010, 2980, 2700, 1754, 1622, 1543, 1412, 1351, 1232, 1092, 1004 cm^{-1}; **HRMS** (m/z): $[M+H]^+$ calcd. for $C_{16}H_{18}NO_2^+$, 256.1334; found, 256.1332.

(2*E*,4*E*)-*N*,2-dimethyl-*N*-(8-oxobicyclo[4.2.0]octa-1(6),2,4-trien-2-yl)octa-2,4-dien amide (1o)

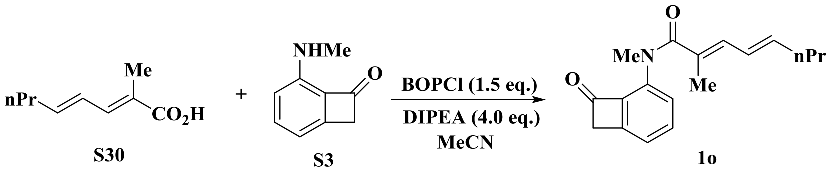

1o was obtained (35 mg, 36% yield, *E*/*Z* = 98:2) as a colorless oil following the **general procedure** Ⅱ from **S30** (104 mg, 0.67 mmol) and **S3** (50 mg, 0.34 mmol).

TLC (Hex : EA = 5:1): R_f = 0.5;

^{1}H NMR (400 MHz, Chloroform-*d*) δ 7.44 - 7.37 (m, 1H), 7.23 (d, J = 7.1 Hz, 1H), 7.08 (d, J = 8.1 Hz, 1H), 6.28 (d, J = 11.0 Hz, 1H), 6.13 (dd, J = 14.3, 11.7 Hz, 1H), 5.74 (dt, J = 14.6, 7.1 Hz, 1H), 3.95 (s, 2H), 3.49 (s, 3H), 2.06 (q, J = 7.1 Hz, 2H), 1.77 (s, 3H), 1.38 (q, J = 7.4 Hz, 2H), 0.86 (t, J = 7.4 Hz, 3H). **^{13}C NMR** (100 MHz, Chloroform-*d*) δ 185.4, 173.3, 151.4, 140.7, 139.3, 137.5, 136.1, 135.9, 129.2, 125.5, 124.0, 119.6, 51.8, 37.3, 35.2, 22.3, 14.4, 13.7. **IR**: ***v*** 3434, 2980, 1841, 1762, 1685, 1643, 1556, 1477, 1324, 1119, 1020 cm^{-1}; **HRMS** (m/z): $[M+H]^+$ calcd. for $C_{18}H_{22}NO_2^+$, 284.1637; found, 284.1645.

(2*E*,4*E*)-5-(furan-2-yl)-*N*,2-dimethyl-*N*-(8-oxobicyclo[4.2.0]octa-1(6),2,4-trien-2-yl)penta-2,4-dienamide (1p)

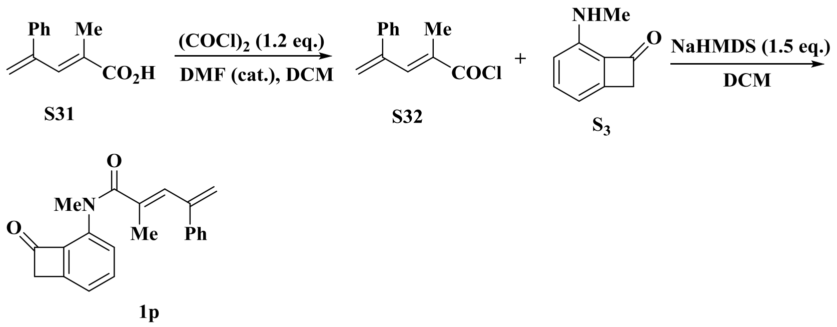

1p was obtained (60 mg, 56% yield, *E*/*Z* = 99:1) as a colorless oil following the **general procedure Ⅰ** from **S31** (191 mg, 1.02 mmol) and **S3** (50 mg, 0.34 mmol).

TLC (Hex : EA = 5:1): R_f = 0.4;

^{1}H NMR (400 MHz, Chloroform-*d*) δ 7.54 - 7.48 (m, 1H), 7.35 (d, J = 7.3 Hz, 1H), 7.24 - 7.18 (m, 3H), 7.12 (d, J = 8.1 Hz, 1H), 6.99 (dd, J = 7.5, 2.0 Hz, 2H), 6.24 (s, 1H), 5.59 (s, 1H), 5.06 (s, 1H), 3.97 (s, 2H), 3.53 (s, 3H), 1.88 (s, 3H). **^{13}C NMR** (100 MHz, Chloroform-*d*) δ 185.3, 173.0, 151.8, 143.1, 140.1, 139.6, 137.4, 136.3, 134.8, 134.3, 128.4, 128.0, 126.3, 124.6, 120.3, 116.6, 52.1, 36.9, 16.1. **IR**: ***v*** 3013, 2956, 2870, 1766, 1687, 1611, 1542, 1436, 1322, 1192, 1123 cm^{-1}; **HRMS** (m/z): $[M+H]^+$ calcd. for $C_{21}H_{20}NO_2^+$, 318.1485; found, 318.1489.

(*E*)-3-(3,4-dihydro-2*H*-pyran-5-yl)-*N*,2-dimethyl-*N*-(8-oxobicyclo[4.2.0]octa-1(6),2,4-trien-2-yl)acrylamide (1q)

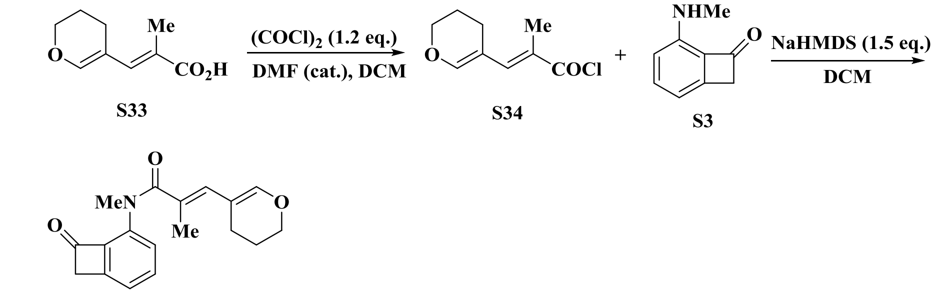

1q

1q was obtained (60 mg, 59% yield, *E*/*Z* = 99:1) as a yellow oil following the **general procedure Ⅰ** from **S33** (171 mg, 1.02 mmol) and **S3** (50 mg, 0.34 mmol).

TLC (Hex : EA = 5:1): R_f = 0.4;

^{1}H NMR (400 MHz, Chloroform-*d*) δ 7.45 - 7.36 (m, 1H), 7.27 - 7.18 (m, 1H), 7.10 (d, J = 8.2 Hz, 1H), 6.45 (s, 1H), 6.06 (s, 1H), 3.97 - 3.91 (m, 4H), 3.47 (s, 3H), 2.23 (t, J = 6.3 Hz, 2H), 1.84 (s, 3H), 1.83 - 1.77 (m, 2H). **^{13}C NMR** (100 MHz, Chloroform-*d*) δ 185.4, 174.5, 151.4, 149.3, 139.1, 137.8, 136.1, 125.9, 123.9, 119.4, 112.1, 66.0, 51.7, 37.3, 23.4, 22.0, 16.0. **IR**: ***v*** 2957, 2920, 2852, 1762, 1646, 1594, 1480, 1454, 1378, 1121, 754 cm^{-1}; **HRMS** (m/z): [M+H]$^+$ calcd. for $C_{18}H_{20}NO_3^+$, 298.1435; found, 298.1437.

(2*E*,4*E*)-5-(furan-2-yl)-*N*,2-dimethyl-*N*-(8-oxobicyclo[4.2.0]octa-1(6),2,4-trien-2-yl)penta-2,4-dienamide (1r)

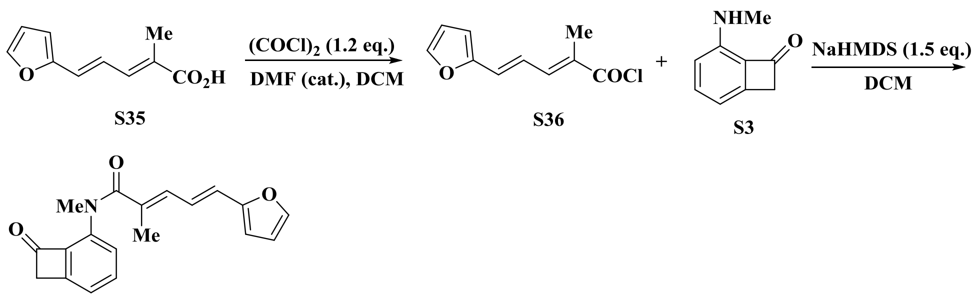

1r

1r was obtained **(**76 mg, 73% yield, *E*/*Z* = 99:1) as a yellow oil following the **general procedure Ⅰ** from **S35** (182 mg, 1.02 mmol) and **S3** (50 mg, 0.34 mmol).

TLC (Hex : EA = 5:1): R_f = 0.3;

^{1}H NMR (400 MHz, Chloroform-*d*) δ 7.43 - 7.34 (m, 2H), 7.24 (d, J = 7.2 Hz, 1H), 7.06 (d, J = 7.8 Hz, 1H), 6.75 (dd, J = 15.4, 11.5 Hz, 1H), 6.43 - 6.38 (m, 1H), 6.37 (dd, J = 3.4, 1.8 Hz, 1H), 6.35 - 6.29 (m, 2H), 3.96 (s, 2H), 3.50 (s, 3H), 1.87 (s, 3H). **^{13}C NMR** (100 MHz, Chloroform-*d*) δ 185.3, 172.9, 152.8, 151.5, 143.0, 139.4, 137.3, 136.2, 135.2, 132.0, 123.8, 122.1, 119.8, 112.1, 110.5, 51.9, 37.2, 14.7. **IR**: ***v*** 3124, 3100, 2923, 1762, 1646, 1593, 1447, 1428, 1361, 1228, 1174 cm^{-1}; **HRMS** (m/z): $[M+H]^+$ calcd. for $C_{19}H_{18}NO_3^+$, 308.1286; found, 308.1281.

(*E*)-3-(2*H*-chromen-3-yl)-*N*,2-dimethyl-*N*-(8-oxobicyclo[4.2.0]octa-1(6),2,4-trien-2-yl)acrylamide (1s)

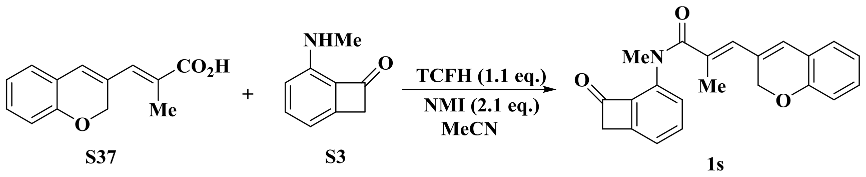

1s was obtained (58 mg, 59% yield, *E*/*Z* = 99:1) as a colorless oil following the **general procedure** Ⅲ from **S37** (61 mg, 0.283 mmol) and **S3** (50 mg, 0.34 mmol). TLC (Hex : EA = 5:1): R_f = 0.4;

^{1}H NMR (400 MHz, Chloroform-*d*) δ 7.50 - 7.43 (m, 1H), 7.30 (d, J = 7.2 Hz, 1H), 7.11 (t, J = 8.5 Hz, 2H), 6.97 (d, J = 8.8 Hz, 1H), 6.87 (t, J = 7.4 Hz, 1H), 6.76 (d, J = 8.1 Hz, 1H), 6.34 (s, 1H), 6.12 (s, 1H), 4.76 (s, 2H), 3.99 (s, 2H), 3.52 (s, 3H), 1.95 (s, 3H). **^{13}C NMR** (100 MHz, Chloroform-*d*) δ 185.3, 172.9, 153.4, 151.7, 139.8, 137.0, 136.2, 133.6, 131.8, 129.9, 129.6, 127.3, 126.2, 124.2, 122.6, 121.9, 120.4, 115.7, 67.5, 52.0, 37.2, 17.1. **IR**: ***v*** 3200, 3121, 2923, 1762, 1652, 1596, 1477, 1450, 1346, 1228, 1127 cm^{-1}; **HRMS** (m/z): $[M+H]^+$ calcd. for $C_{22}H_{20}NO_3^+$, 346.1433; found, 346.1438.

(*E*)-3-(benzofuran-2-yl)-*N*,2-dimethyl-*N*-(8-oxobicyclo[4.2.0]octa-1(6),2,4-trien-2-yl)acrylamide (1t)

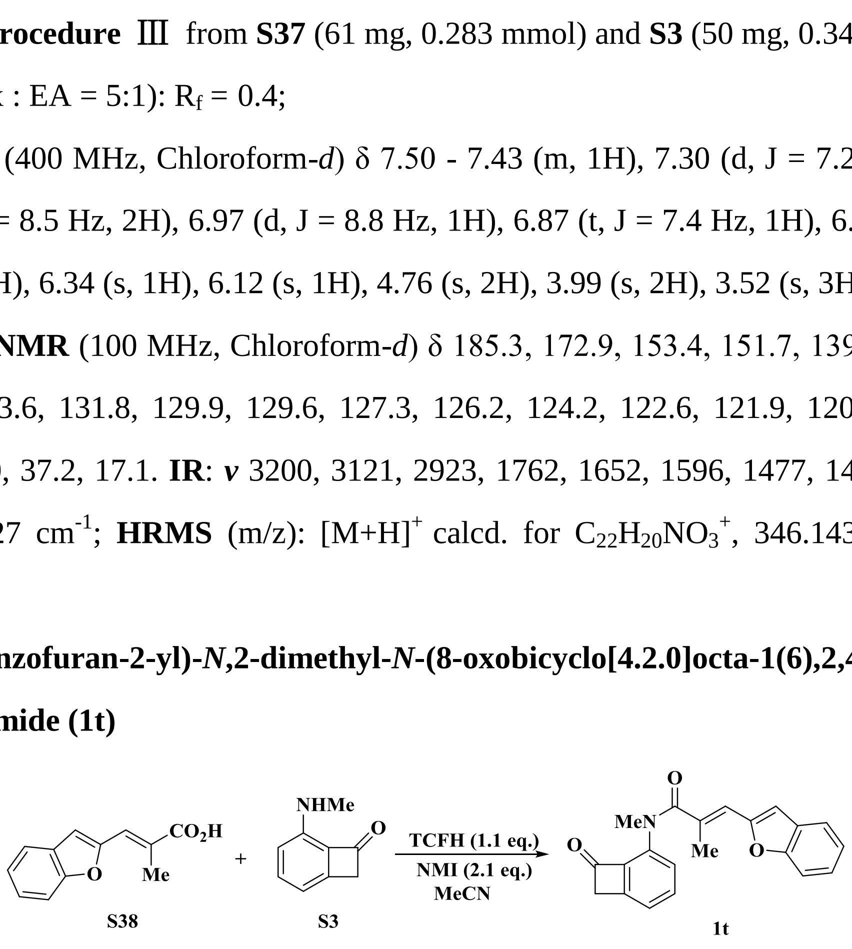

1t was obtained (66 mg, 70% yield, *E*/*Z* = 99:1) as a colorless oil following the **general procedure Ⅲ** from **S38** (57 mg, 0.283 mmol) and **S3** (50 mg, 0.34 mmol). TLC (Hex : EA = 5:1): R_f = 0.4;

^{1}H NMR (400 MHz, Chloroform-*d*) δ 7.52 (d, J = 7.7 Hz, 1H), 7.41 (dt, J = 8.2, 3.6 Hz, 2H), 7.26 (d, J = 7.9 Hz, 2H), 7.20 (d, J = 7.4 Hz, 1H), 7.13 (d, J = 8.2 Hz, 1H), 6.68 (d, J = 3.4 Hz, 2H), 3.97 (s, 2H), 3.55 (s, 3H), 2.16 (s, 3H). **^{13}C NMR** (100 MHz, Chloroform-*d*) δ 185.3, 172.6, 155.0, 153.5, 151.7, 139.6, 136.9, 136.4, 133.9, 128.4, 125.4, 124.1, 123.3, 121.3, 120.3, 111.3, 109.1, 52.0, 37.3, 17.0. **IR**: ***v*** 3056, 2948, 2921, 1762, 1648, 1596, 1477, 1430, 1342, 1294, 1232 cm^{-1}; **HRMS** (m/z): $[M+H]^+$ calcd. for $C_{21}H_{18}NO_3^+$, 332.1285; found, 332.1281.

(*E*)-3-(benzo[b]thiophen-2-yl)-*N*,2-dimethyl-*N*-(8-oxobicyclo[4.2.0]octa-1(6),2,4-trien-2-yl)acrylamide (1u)

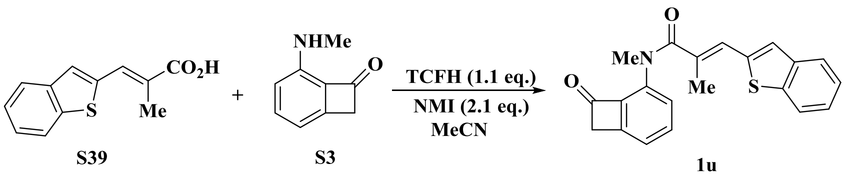

1u was obtained (67 mg, 68% yield, *E*/*Z* = 99:1) as a faint yellow solid following the **general procedure Ⅲ** from **S39** (62 mg, 0.283 mmol) and **S3** (50 mg, 0.34 mmol). TLC (Hex : EA = 5:1): R_f = 0.4;

^{1}H NMR (400 MHz, Chloroform-*d*) δ 7.84 - 7.70 (m, 2H), 7.42 (dd, J = 8.1, 7.3 Hz, 1H), 7.35 - 7.30 (m, 2H), 7.28 (s, 1H), 7.23 (s, 1H), 7.14 (d, J = 8.4 Hz, 1H), 7.02 (s, 1H), 3.98 (s, 2H), 3.57 (s, 3H), 2.09 (s, 3H). **^{13}C NMR** (100 MHz, Chloroform-*d*) δ 185.3, 172.8, 151.7, 140.9, 139.7, 139.0, 138.9, 136.9, 136.3, 132.6, 129.2, 126.7, 125.1, 124.8, 124.1, 124.0, 122.2, 120.3, 52.0, 37.3, 16.7. **IR**: ***v*** 3032, 2966, 2921, 1762, 1653, 1561, 1434, 1466, 1321, 1294, 1259 cm^{-1}; **HRMS** (m/z): $[M+H]^+$ calcd. for $C_{21}H_{18}NO_2S^+$, 348.1054; found, 348.1053. **mp:** 165-166 ^{o}C.

(*R*,*E*)-*N*,2-dimethyl-*N*-(8-oxobicyclo[4.2.0]octa-1(6),2,4-trien-2-yl)-3-(4-(prop-1-en-2-yl)cyclohex-1-en-1-yl)acrylamide (1v)

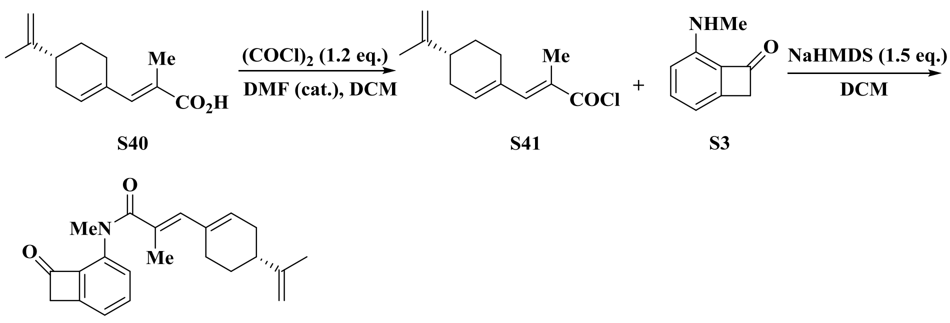

1v was obtained (59 mg, 52% yield, *E*/*Z* = 96:4) as a colorless oil following the **general procedure Ⅰ** from **S40** (210 mg, 1.02 mmol) and **S3** (50 mg, 0.34 mmol). TLC (Hex : EA = 5:1): R_f = 0.55;

^{1}H NMR (400 MHz, Chloroform-*d*) δ 7.48 - 7.41 (m, 1H), 7.27 (s, 1H), 7.14 (q, J = 9.9, 8.4 Hz, 1H), 6.05 (s, 1H), 5.62 (s, 1H), 4.70 (d, J = 12.7 Hz, 2H), 3.97 (s, 2H), 3.51 (s, 3H), 2.26 - 2.16 (m, 1H), 2.09 (dd, J = 7.4, 4.8 Hz, 3H), 1.89 (s, 3H), 1.83 - 1.76 (m, 1H), 1.72 (s, 3H), 1.47 - 1.36 (m, 1H), 1.29 - 1.21 (m, 1H). **^{13}C NMR** (100 MHz, Chloroform-*d*) δ 185.3, 174.0, 151.4, 149.5, 139.5, 137.5, 136.0, 134.1, 130.4, 129.7, 124.2, 119.7, 108.9, 51.8, 40.4, 37.2, 31.2, 28.8, 27.6, 20.8, 16.1. **IR**: ***v*** 2963, 2926, 2858, 1762, 1717, 1635, 1594, 1479, 1343, 1163, 1076 cm^{-1}; **HRMS** (m/z): $[M+H]^+$ calcd. for $C_{22}H_{26}NO_2^+$, 336.1953; found, 336.1958.

(2*E*,4*E*)-*N*,2-dimethyl-*N*-(8-oxobicyclo[4.2.0]octa-1(6),2,4-trien-2-yl)-5-phenylpenta-2,4-dienamide-5-d (1a-D)

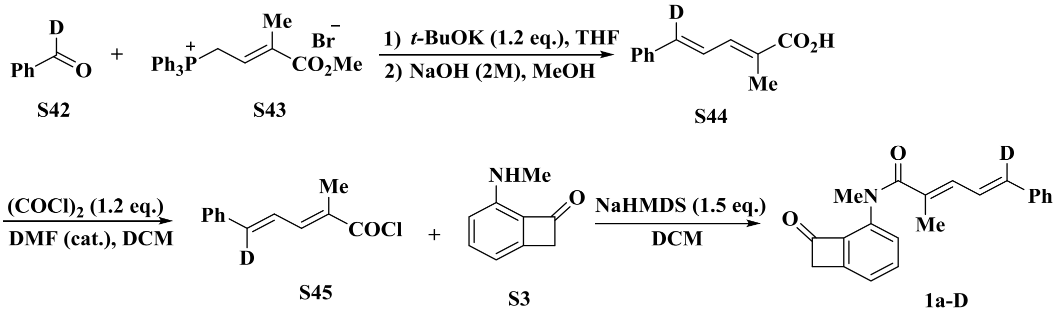

To a 10 mL flamed-dried Schlenk flask equipped with a stir bar and a nitrogen-filled balloon was added **S43** (25 g, 1.0 equiv, 6.07 mmol) in dry THF (30 mL). The system was cooled to 0 ℃ before *t*-BuOK (7.28 mL, 1.2 equiv. 7.28 mmol, 1.0 mol/L in THF) was added dropwisely. The system was kept at 0 ℃ and stirred for 0.5 h under

nitrogen atmosphere. Then **S42** (650 mg, 1.0 equiv, 6.07 mmol) dissolved in THF (5 mL) was added to up system, the flask was removed to rt stirred upon completion of the addition. Then the reaction was quenched with saturated aqueous NH_4Cl solution (20 mL), the mixture was extracted with EtOAc (3×10 mL), washed with brine, and dried over $MgSO_4$. The combined organic extract was concentrated under reduced pressure, the residue was purified by flash chromatography to obtained ester. After that to a solution of ester in MeOH (10 mL) was added NaOH (9 mL, 3 equiv, 2.0 M), the solution was stirred at 60 ℃ for 10 h. Then the pH of the system is neutralized to 7.0 with HCl (2.0 M), the mixture was extracted with DCM (3×10 mL), washed with brine, and dried over $MgSO_4$, and evaporated in vacuo. The residue was purified by flash chromatography (*n*hexane/EtOAc = 5:1) to afford **S44** (711 mg, 62%, *E*/*Z* = 98:2) as a white solid.

TLC (Hex : EA = 5:1): R_f = 0.2;

^{1}H NMR (400 MHz, Chloroform-d) δ 7.52 - 7.47 (m, 3H), 7.37 (t, J = 7.3 Hz, 2H), 7.32 (d, J = 7.2 Hz, 1H), 7.08 (d, J = 11.5 Hz, 1H), 2.07 (s, 3H). **^{13}C NMR** (400 MHz, Chloroform-d) δ 173.5, 140.7, 136.4, 129.1, 128.9, 127.3, 126.4, 123.7, 122.8, 12.6. **IR**: ***v*** 2921, 1658, 1415, 1243, 1110, 970, 804 cm^{-1}; **HRMS** (m/z): $[M-H]^-$ calcd. for $C_{12}H_{10}DO_2^-$, 188.0833; found, 188.0827. **mp**: 148-149 ^oC.

1a-D was obtained (58.4 mg, 54% yield, *E*/*Z* = 96:4) as a colorless oil following the **general procedure** Ⅰ from **S44** (193 mg, 1.02 mmol) and **S3** (50 mg, 0.34 mmol).

TLC (Hex : EA = 5:1): R_f = 0.4;

^{1}H NMR (400 MHz, Chloroform-d) δ 7.43 - 7.36 (m, 3H), 7.30 (t, J = 7.5 Hz, 2H), 7.24 (d, J = 4.7 Hz, 2H), 7.09 (s, 1H), 6.86 (d, J = 11.2 Hz, 1H), 6.47 (d, J = 12.3 Hz, 1H), 3.97 (s, 2H), 3.52 (s, 3H), 1.91 (s, 3H). **^{13}C NMR** (100 MHz, Chloroform-*d*) δ 185.4, 173.0, 151.6, 139.4, 137.3, 136.7, 136.2, 135.5, 131.9, 128.8, 128.5, 126.9, 124.0, 123.4, 119.9, 51.9, 37.3, 14.8. **IR**: ***v*** 2964, 2934, 2900, 1731, 1709, 1663, 1577, 1421, 1343, 1137, 1040 cm^{-1}; **HRMS** (m/z): $[M+H]^+$ calcd. for $C_{21}H_{19}DNO_2^+$, 319.1549; found, 319.1551.

N-methyl-N-(8-oxobicyclo[4.2.0]octa-1(6),2,4-trien-2-yl)-2-vinylbenzamide (6)

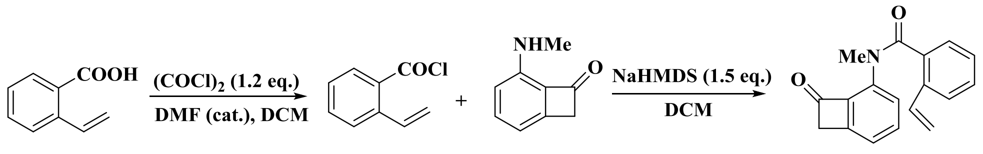

6 was obtained (40 mg, 42% yield) as a colorless oil following the **general procedure** Ⅰ from **S46** (151 mg, 1.02 mmol) and **S3** (50 mg, 0.34 mmol).

TLC (Hex : EA = 5:1): R_f = 0.7;

^{1}**H NMR** (400 MHz, Chloroform-*d*) δ 7.43 (d, J = 7.8 Hz, 1H), 7.32 - 7.15 (m, 5H), 6.98 (dd, J = 18.5, 8.3 Hz, 1H), 6.77 (dd, J = 17.4, 11.0 Hz, 1H), 5.60 (d, J = 17.4 Hz, 1H), 5.25 (d, J = 11.0 Hz, 1H), 3.92 (s, 2H), 3.50 (s, 3H). ^{13}**C NMR** (100 MHz, Chloroform-*d*) δ 185.2, 170.5, 151.1, 140.3, 135.8, 135.1, 133.7, 129.7, 127.7, 125.7, 125.2, 120.4, 116.9, 51.8, 37.3. **IR**: ***v*** 3074, 2963, 1759, 1731, 1700, 1652, 1477, 1347, 1311, 1047, 998 cm^{-1}; **HRMS** (m/z): $[M+H]^+$ calcd. for $C_{18}H_{16}NO_2^+$, 278.1171; found, 278.1176.

Ⅲ. *Procedure for a [4+4] Coupling and characterization of compounds (2a~2v)*

General procedure Ⅳ

To a 4-mL over-dried vial in the glove box was added substrate (1.0 equiv), $[Rh(nbd)_2Cl]_2$ (0.025 equiv), PPh_3 (0.12 equiv). Followed add 1,4-dioxane (3.7 mL) and the vial was capped. The solution was stirred at 150 ℃ under N_2 for 96 h. Upon completion, it was cooled to rt, and the solvent was removed in vacuo. The crude product was purified by flash chromatography to afford desired product.

(*Z*)-9,12-dimethyl-6-phenyl-6,9-dihydro-1,9-(epiminomethano)benzo[8]annulene-10,11(5*H*)-dione (2a)

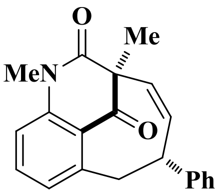

2a

2a was obtained (28.8 mg, 72% yield) as colorless oil together with recycled **1a** (4.9 mg) following **the general procedure IV** from **1a** (40 mg, 0.126 mmol).

TLC (Hex : EA = 5:1): R_f = 0.6;

^{1}H NMR (400 MHz, Chloroform-*d*) δ 7.32 (dt, J = 7.9, 4.1 Hz, 3H), 7.26 - 7.21 (m, 3H), 6.88 (dd, J = 7.8, 3.6 Hz, 2H), 5.51 (d, J = 8.6 Hz, 2H), 4.27 - 4.04 (m, 1H), 3.87 (dd, J = 16.2, 8.6 Hz, 1H), 3.32 (s, 3H), 2.81 (dd, J = 16.3, 10.9 Hz, 1H), 1.59 (s, 3H). **^{13}C NMR** (100 MHz, Chloroform-*d*) δ 207.4, 169.8, 143.5, 139.1, 137.5, 137.2, 131.4, 130.8, 129.0, 127.6, 127.0, 124.7, 112.1, 63.4, 42.7, 41.8, 30.7, 15.7. **IR**: ***v*** 2920, 2850, 1759, 1725, 1635, 1567, 1468, 1336, 1053, 901, 745 cm^{-1}; **HRMS** (m/z): $[M+H]^+$ calcd. for $C_{21}H_{20}NO_2^+$, 318.1486; found, 318.1489.

(6*R*,9*R*,*Z*)-6-(4-fluorophenyl)-9,12-dimethyl-6,9-dihydro-1,9-(epiminomethano)benzo[8]annulene-10,11(5*H*)-dione (2b)

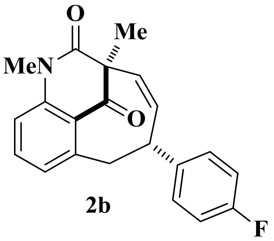

2b

2b was obtained (33.8 mg, 75% yield) as a faint yellow solid together with recycled **1b** (5.2 mg) following **the general procedure Ⅳ** from **1b** (45 mg, 0.134 mmol).

TLC (Hex : EA = 5:1): R_f = 0.6;

^{1}H NMR (400 MHz, Chloroform-*d*) δ 7.33 (t, J = 7.9 Hz, 1H), 7.25 - 7.12 (m, 2H), 7.02 (t, J = 8.6 Hz, 2H), 6.89 (d, J = 8.0 Hz, 2H), 5.54 (d, J = 10.7 Hz, 1H), 5.47 - 5.36 (m, 1H), 4.28 - 4.07 (m, 1H), 3.94 - 3.79 (m, 1H), 3.33 (s, 3H), 2.77 (dd, J = 16.3, 10.9 Hz, 1H), 1.60 (s, 3H). **^{13}C NMR** (100 MHz, Chloroform-*d*) δ 207.4, 169.7, 139.1, 137.3, 136.9, 131.6, 131.2, 130.9, 129.1, 129.1, 124.7, 116.0, 115.7, 112.2, 63.3, 41.0, 30.7, 15.8. **^{19}F NMR** (376 MHz, Chloroform-*d*) δ -115.71. **IR**: ***v*** 3064, 2929, 2847,

1731, 1681, 1587, 1509, 1463, 1334, 1222, 1047 cm^{-1}; **HRMS** (m/z): $[M-H]^-$ calcd. for $C_{21}H_{17}FNO_2^-$, 334.1247; found, 334.1249. **mp**: 140-141 ^oC.

(6*R*,9*R*,*Z*)-6-(4-chlorophenyl)-9,12-dimethyl-6,9-dihydro-1,9-(epiminomethano)benzo[8]annulene-10,11(5*H*)-dione (2c)

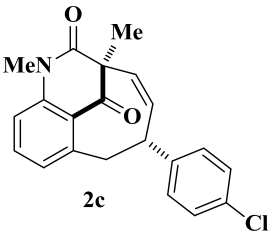

2c

2c was obtained (24.5 mg, 70% yield) as a faint yellow oil together with recycled **1c** (5.8 mg) following **the general procedure IV** from **1c** (35 mg, 0.099 mmol).

TLC (Hex : EA = 5:1): R_f = 0.6;

^{1}H NMR (400 MHz, Chloroform-*d*) δ 7.31 (dd, J = 15.2, 8.2 Hz, 3H), 7.14 (d, J = 8.4 Hz, 2H), 6.88 (d, J = 7.6 Hz, 2H), 5.53 (d, J = 12.0 Hz, 1H), 5.47 - 5.38 (m, 1H), 4.14 (q, J = 10.3 Hz, 1H), 3.85 (dd, J = 16.2, 8.6 Hz, 1H), 3.31 (s, 3H), 2.75 (dd, J = 16.3, 10.9 Hz, 1H), 1.58 (s, 3H). **^{13}C NMR** (100 MHz, Chloroform-*d*) δ 207.3, 169.6, 142.0, 139.1, 137.1, 136.6, 132.8, 131.8, 131.1, 130.9, 129.2, 129.0, 112.3, 63.3, 42.6, 41.1, 30.7, 15.8. **IR**: ***v*** 3019, 2982, 2929, 2850, 1731, 1681, 1587, 1490, 1463, 1336, 1276 cm^{-1}; **HRMS** (m/z): $[M+H]^+$ calcd. for $C_{21}H_{19}ClNO_2^+$, 352.1101; found, 352.1099.

(6*R*,9*R*,*Z*)-6-(4-methoxyphenyl)-9,12-dimethyl-6,9-dihydro-1,9-(epiminomethano)benzo[8]annulene-10,11(5*H*)-dione (2d)

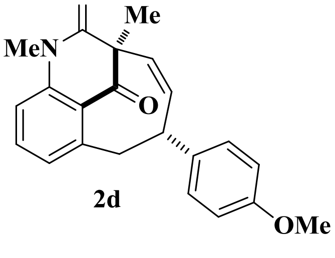

2d

2d was obtained (27.2 mg, 68% yield) as a faint yellow oil together with recycled **1d** (9.1 mg) following **the general procedure IV** from **1d** (40 mg, 0.115 mmol).

TLC (Hex : EA = 5:1): R_f = 0.5;

^{1}H NMR (400 MHz, Chloroform-*d*) δ 7.32 (t, *J* = 7.9 Hz, 1H), 7.14 (d, *J* = 8.7 Hz, 2H), 6.92 - 6.81 (m, 4H), 5.56 - 5.40 (m, 2H), 4.19 - 4.07 (m, 1H), 3.85 (d, *J* = 7.6 Hz, 1H), 3.79 (s, 3H), 3.32 (s, 3H), 2.85 - 2.70 (m, 1H), 1.60 (s, 3H). **^{13}C NMR** (100 MHz, Chloroform-*d*) δ 207.5, 169.8, 158.6, 137.6, 137.5, 135.4, 131.3, 131.1, 130.7, 128.6, 124.7, 114.4, 112.1, 63.3, 55.4, 42.8, 40.9, 30.7, 15.8. **IR**: ***v*** 3012, 2982, 2926, 2850, 1731, 1683, 1652, 1585, 1508, 1248, 1047 cm^{-1}; **HRMS** (m/z): [M+H]$^+$ calcd. for $C_{22}H_{22}NO_3^+$, 348.1599; found, 348.1594.

(6*R*,9*R*,*Z*)-9-ethyl-12-methyl-6-phenyl-6,9-dihydro-1,9-(epiminomethano)benzo[8]annulene-10,11(5*H*)-dione (2e)

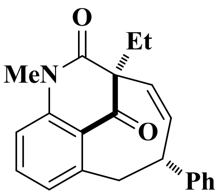

2e

2e was obtained (23.4 mg, 65% yield) as a colorless oil together with recycled **1e** (6.0 mg) following **the general procedure IV** from **1e** (36 mg, 0.109 mmol).

TLC (Hex : EA = 5:1): R_f = 0.6;

^{1}H NMR (400 MHz, Chloroform-*d*) δ 7.35 - 7.29 (m, 3H), 7.25 - 7.21 (m, 3H), 6.95 - 6.82 (m, 2H), 5.63 (t, J = 10.2 Hz, 1H), 5.38 (d, J = 10.8 Hz, 1H), 4.20 (q, J = 9.6 Hz, 1H), 3.84 (dd, J = 16.3, 8.5 Hz, 1H), 3.30 (s, 3H), 2.82 (dd, J = 16.3, 11.1 Hz, 1H), 2.29 - 2.05 (m, 2H), 1.17 (t, J = 7.2 Hz, 3H). **^{13}C NMR** (100 MHz, Chloroform-*d*) δ 206.7, 169.8, 143.6, 139.0, 138.9, 137.4, 131.3, 130.6, 129.1, 127.7, 124.5, 111.8, 67.1, 42.9, 42.2, 30.7, 23.1, 9.0. **IR**: ***v*** 3030, 2923, 2852, 1725, 1679, 1585, 1463, 1333, 1278, 1141, 1062 cm^{-1}; **HRMS** (m/z): [M+H]$^+$ calcd. for $C_{22}H_{22}NO_2^+$, 332.1684; found, 332.1683.

(6*R*,9*R*,*Z*)-9-(cyclopropylmethyl)-12-methyl-6-phenyl-6,9-dihydro-1,9-(epiminomethano)benzo[8]annulene-10,11(5*H*)-dione (2f)

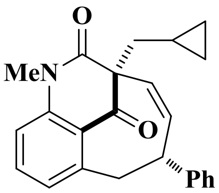

2f

2f was obtained (9.2 mg, 46% yield) as a colorless oil together with recycled **1f** (7.6 mg) following **the general procedure IV** from **1f** (20 mg, 0.056 mmol).

TLC (Hex : EA = 5:1): R_f = 0.7;

^{1}H NMR (400 MHz, Chloroform-*d*) δ 7.31 (td, J = 7.5, 4.1 Hz, 3H), 7.24 - 7.18 (m, 3H), 6.96 - 6.78 (m, 2H), 5.79 - 5.58 (m, 1H), 5.46 (dd, J = 10.8, 1.3 Hz, 1H), 4.32 (q, J = 9.9 Hz, 1H), 3.87 (dd, J = 16.3, 8.7 Hz, 1H), 3.30 (s, 3H), 2.79 (dd, J = 16.4, 11.0 Hz, 1H), 2.13 (dd, J = 14.4, 6.6 Hz, 1H), 2.03 (dd, J = 14.5, 7.3 Hz, 1H), 1.07 (dt, J = 11.9, 5.0 Hz, 1H), 0.48 (tdd, J = 9.3, 5.8, 3.9 Hz, 1H), 0.38 (dq, J = 9.0, 4.9, 4.4 Hz, 1H), 0.31 (td, J = 9.3, 5.3 Hz, 1H), 0.12 (dq, J = 9.0, 4.9 Hz, 1H). **^{13}C NMR** (100 MHz, Chloroform-*d*) δ 206.5, 169.8, 138.7, 131.4, 130.5, 129.3, 129.0, 126.9, 124.5, 111.8, 67.3, 43.3, 42.3, 34.7, 30.8, 6.5, 5.9, 5.6. **IR**: ***v*** 3002, 2920, 2855, 1727, 1677, 1587, 1463, 1333, 1276, 1141, 1076 cm^{-1}; **HRMS** (m/z): $[M+H]^+$ calcd. for $C_{24}H_{24}NO_2^+$, 358.1804; found, 358.1802.

(6*R*,9*R*,*Z*)-8,12-dimethyl-6-phenyl-6,9-dihydro-1,9-(epiminomethano)benzo[8]annulene-10,11(5*H*)-dione (2g)

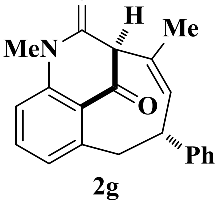

2g

2g was obtained (26.5 mg, 63% yield) as a colorless oil together with recycled **1g** (4.7 mg) following **the general procedure IV** from **1g** (42 mg, 0.132 mmol).

TLC (Hex : EA = 5:1): R_f = 0.6;

^{1}H NMR (400 MHz, Chloroform-*d*) δ 7.32 (q, J = 7.0 Hz, 3H), 7.25 (s, 3H), 7.23 (d, J = 1.8 Hz, 1H), 6.89 (d, J = 7.9 Hz, 2H), 5.38 (d, J = 9.4 Hz, 1H), 4.29 (s, 1H), 4.27 - 4.19 (m, 1H), 3.78 (dd, J = 16.4, 8.4 Hz, 1H), 3.34 (s, 3H), 2.75 (dd, J = 16.4, 11.1 Hz,

1H), 1.62 (s, 3H). **^{13}C NMR** (100 MHz, Chloroform-*d*) δ 205.3, 166.9, 144.3, 139.0, 138.2, 133.8, 131.5, 130.9, 129.0, 127.8, 126.9, 124.8, 112.3, 66.5, 42.9, 42.2, 30.1, 23.3. **IR**: ***v*** 3434, 3000, 2855, 1869, 1731, 1646, 1553, 1453, 1333, 1276, 1066 cm^{-1}; **HRMS** (m/z): [M+H]$^+$ calcd. for $C_{21}H_{20}NO_2^+$, 318.1491; found, 318.1489.

(6*R*,9*R*,*E*)-12-methyl-6,8-diphenyl-6,9-dihydro-1,9-(epiminomethano)benzo[8]annulene-10,11(5*H*)-dione (2h)

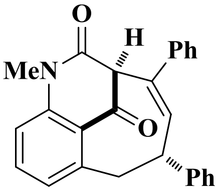

2h

2h was obtained (20 mg, 50% yield) as a colorless oil together with recycled **1h** (7.2 mg) following **the general procedure IV** from **1h** (40 mg, 0.105 mmol).

TLC (Hex : EA = 5:1): R_f = 0.7;

^{1}H NMR (400 MHz, Chloroform-*d*) δ 7.38 (t, J = 7.9 Hz, 1H), 7.35 - 7.26 (m, 4H), 7.25 - 7.20 (m, 1H), 7.19 - 7.14 (m, 3H), 6.96 (d, J = 7.6 Hz, 1H), 6.87 (d, J = 8.2 Hz, 1H), 6.81 (dd, J = 6.7, 2.9 Hz, 2H), 5.62 (d, J = 12.5 Hz, 1H), 4.80 (s, 1H), 4.40 (q, J = 10.1 Hz, 1H), 3.95 (dd, J = 16.1, 8.8 Hz, 1H), 3.09 (s, 3H), 2.87 (dd, J = 16.2, 10.7 Hz, 1H). **^{13}C NMR** (100 MHz, Chloroform-*d*) δ 204.3, 165.8, 143.7, 140.3, 139.5, 138.4, 138.1, 138.1, 131.8, 131.1, 129.0, 128.0, 127.8, 127.8, 127.5, 127.0, 124.7, 112.8, 65.9, 43.3, 42.6, 30.0. **IR**: ***v*** 3064, 3030, 2923, 2855, 1731, 1681, 1585, 1461, 1336, 1143, 1070 cm^{-1}; **HRMS** (m/z): [M+H]$^+$ calcd. for $C_{26}H_{22}NO_2^+$, 380.1648; found, 380.1645.

(6*R*,9*R*,*Z*)-12-benzyl-9-methyl-6-phenyl-6,9-dihydro-1,9-(epiminomethano)benzo[8]annulene-10,11(5*H*)-dione (2i)

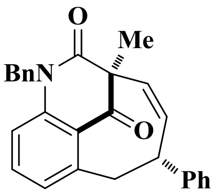

2i

2i was obtained (30.3 mg, 89% yield) as a faint yellow solid together with recycled **1i** (2.1 mg) following **the general procedure IV** from **1i** (34 mg, 0.086 mmol).

TLC (Hex : EA = 5:1): R_f = 0.65;

^{1}**H NMR** (400 MHz, Chloroform-*d*) δ 7.37 - 7.28 (m, 4H), 7.26 (s, 1H), 7.25 - 7.21 (m, 3H), 7.20 - 7.14 (m, 3H), 6.84 (d, J = 7.5 Hz, 1H), 6.75 (d, J = 8.1 Hz, 1H), 5.67 (d, J = 10.7 Hz, 1H), 5.62 - 5.52 (m, 1H), 5.18 (d, J = 16.2 Hz, 1H), 5.05 (d, J = 16.2 Hz, 1H), 4.18 (q, J = 9.6 Hz, 1H), 3.88 (dd, J = 16.2, 8.7 Hz, 1H), 2.82 (dd, J = 16.3, 10.8 Hz, 1H), 1.65 (s, 3H). ^{13}**C NMR** (100 MHz, Chloroform-*d*) δ 207.2, 169.9, 143.5, 138.2, 137.7, 137.5, 136.1, 131.6, 131.3, 130.7, 129.1, 129.0, 127.7, 127.5, 127.1, 126.4, 124.7, 113.4, 63.6, 46.8, 42.8, 41.9, 15.8. **IR**: ***v*** 2926, 2847, 1728, 1683, 1584, 1505, 1451, 1361, 1126, 1043, 728 cm^{-1}; **HRMS** (m/z): $[M+H]^+$ calcd. for $C_{27}H_{24}NO_2^+$, 394.1805; found, 394.1802. **mp**: 190-191 ^{o}C.

(6*R*,9*R*,*Z*)-12-(cyclopropylmethyl)-9-methyl-6-phenyl-6,9-dihydro-1,9-(epiminomethano)benzo[8]annulene-10,11(5*H*)-dione (2j)

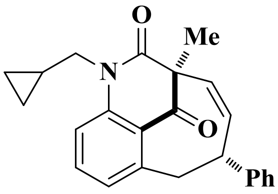

2j

2j was obtained (33.2 mg, 79% yield) as a white solid together with recycled **1j** (6.6 mg) following **the general procedure** Ⅳ from **1j** (42 mg, 0.117 mmol).

TLC (Hex : EA = 5:1): R_f = 0.7;

^{1}**H NMR** (400 MHz, Chloroform-*d*) δ 7.35 - 7.30 (m, 3H), 7.23 (t, J = 7.1 Hz, 3H), 7.00 (d, J = 8.2 Hz, 1H), 6.87 (d, J = 7.5 Hz, 1H), 5.65 - 5.46 (m, 2H), 4.15 (q, J = 9.1 Hz, 1H), 3.89 (dt, J = 15.2, 7.5 Hz, 2H), 3.69 (dd, J = 14.6, 6.2 Hz, 1H), 2.88 - 2.75 (m, 1H), 1.59 (s, 3H), 1.15 (dt, J = 11.1, 6.7 Hz, 1H), 0.55 (ddt, J = 11.9, 7.4, 4.5 Hz,

1H), 0.46 (ddt, J = 16.8, 9.2, 4.6 Hz, 2H), 0.30 (dt, J = 8.8, 4.4 Hz, 1H). **^{13}C NMR** (100 MHz, Chloroform-*d*) δ 207.4, 169.6, 143.6, 138.4, 137.6, 137.4, 131.7, 130.7, 129.0, 127.7, 127.0, 112.9, 63.6, 46.8, 42.9, 41.8, 15.7, 9.7, 4.6. **IR**: ***v*** 2952, 2921, 2850, 1731, 1675, 1583, 1488, 1457, 1334, 1234, 1041 cm^{-1}; **HRMS** (m/z): $[M+H]^+$ calcd. for $C_{24}H_{24}NO_2^+$, 358.1728; found, 358.1726. **mp:** 178-179 °C.

(6*R*,9*R*,*Z*)-9-methyl-12-(2-methylallyl)-6-phenyl-6,9-dihydro-1,9-(epiminomethano)benzo[8]annulene-10,11(5*H*)-dione (2k)

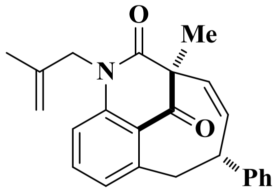

2k

2k was obtained (24.7 mg, 65% yield) as a colorless solid together with recycled **1k** (7.5 mg) following **the general procedure IV** from **1k** (38 mg, 0.106 mmol).

TLC (Hex : EA = 5:1): R_f = 0.7;

^{1}H NMR (400 MHz, Chloroform-*d*) δ 7.34 (t, J = 7.3 Hz, 2H), 7.26 - 7.22 (m, 3H), 6.85 (dd, J = 15.4, 7.8 Hz, 2H), 5.56 (dt, J = 19.5, 10.6 Hz, 2H), 4.91 (s, 1H), 4.67 (s, 1H), 4.42 (s, 2H), 4.17 (q, J = 9.2 Hz, 1H), 3.89 (dd, J = 16.2, 8.8 Hz, 1H), 2.90 - 2.75 (m, 1H), 1.75 (s, 3H), 1.61 (s, 3H). **^{13}C NMR** (100 MHz, Chloroform-*d*) δ 207.3, 169.5, 143.5, 139.1, 138.3, 137.6, 137.4, 131.5, 131.3, 130.6, 129.1, 127.7, 127.0, 124.6, 113.3, 111.4, 63.5, 48.7, 42.8, 41.9, 20.1, 15.7. **IR**: ***v*** 3030, 2957, 2929, 2852, 1765, 1734, 1683, 1587, 1474, 1330, 1160 cm^{-1}; **HRMS** (m/z): $[M+H]^+$ calcd. for $C_{24}H_{24}NO_2^+$, 358.1805; found, 358.1802. **mp**: 200-201 °C.

(6*R*,9*R*,*Z*)-3,9,12-trimethyl-6-phenyl-6,9-dihydro-1,9-(epiminomethano)benzo[8]annulene-10,11(5*H*)-dione (2l)

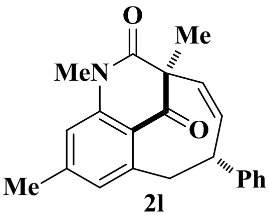

2l

2l was obtained (26.4 mg, 66% yield) as a white solid together with recycled **1l** (7.4 mg) following **the general procedure IV** from **1l** (40 mg, 0.121 mmol).

TLC (Hex : EA = 5:1): R_f = 0.6;

^{1}H NMR (400 MHz, Chloroform-*d*) δ 7.35 - 7.29 (m, 2H), 7.22 (tt, J = 4.4, 2.5 Hz, 3H), 6.77 - 6.66 (m, 2H), 5.50 (d, J = 7.3 Hz, 2H), 4.20 - 4.09 (m, 1H), 3.83 (dd, J = 16.1, 8.7 Hz, 1H), 3.30 (s, 3H), 2.75 (dd, J = 16.2, 10.8 Hz, 1H), 2.36 (s, 3H), 1.57 (s, 3H). **^{13}C NMR** (100 MHz, Chloroform-*d*) δ 207.7, 169.9, 143.7, 141.4, 139.3, 137.4, 137.1, 131.6, 129.0, 127.7, 127.0, 125.4, 113.0, 63.3, 42.8, 41.7, 30.7, 22.0, 15.7. **IR**: ***v*** 3248, 2963, 2929, 1728, 1677, 1627, 1505, 1415, 1333, 1256, 1011 cm^{-1}; **HRMS** (m/z): $[M+H]^+$ calcd. for $C_{22}H_{22}NO_2^+$, 331.1640; found, 331.1645. **mp**: 203-204 ^oC.

(6*R*,9*R*,*Z*)-7,9,12-trimethyl-6-phenyl-6,9-dihydro-1,9-(epiminomethano)benzo[8]annulene-10,11(5*H*)-dione (2m)

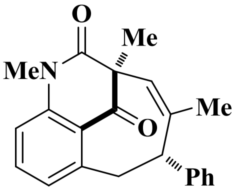

2m

2m was obtained (19.4 mg, 43% yield) as a white solid together with recycled **1m** (8.4 mg) following **the general procedure IV** from **1m** (45 mg, 0.136 mmol).

TLC (Hex : EA = 5:1): R_f = 0.6;

^{1}H NMR (400 MHz, Chloroform-*d*) δ 7.31 (q, J = 7.5 Hz, 3H), 7.22 (d, J = 7.4 Hz, 1H), 7.19 (d, J = 7.4 Hz, 2H), 6.97 (d, J = 7.6 Hz, 1H), 6.86 (d, J = 8.1 Hz, 1H), 5.27 (s, 1H), 4.40 (dd, J = 12.3, 7.4 Hz, 1H), 3.68 (dd, J = 15.9, 7.3 Hz, 1H), 3.31 (s, 3H), 3.24 - 3.10 (m, 1H), 1.67 (s, 3H), 1.04 (s, 3H). **^{13}C NMR** (100 MHz, Chloroform-*d*) δ 207.4, 170.1, 143.2, 140.3, 138.6, 136.7, 132.3, 130.1, 128.6, 128.2, 127.0, 126.7, 124.8, 112.4, 63.4, 44.0, 36.1, 30.7, 20.7, 16.4. **IR**: ***v*** 3032, 2901, 2872, 1751, 1717, 1680, 1556, 1505, 1451, 1156, 773 cm^{-1}; **HRMS** (m/z): $[M+Na]^+$ calcd. for $C_{22}H_{21}NO_2Na^+$, 354.1459; found, 354.1465. **mp:** 150-151 ^oC.

(6*R*,9*R*,*Z*)-6,9,12-trimethyl-6,9-dihydro-1,9-(epiminomethano)benzo[8]annulene-

10,11(5*H*)-dione (2n)

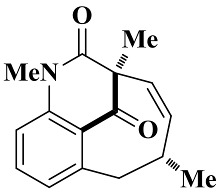

2n

2n was obtained (34.7 mg, 89% yield) as a colorless oil together with recycled **1n** (1.7 mg) following **the general procedure IV** from **1n** (39 mg, 0.153 mmol).

TLC (Hex : EA = 5:1): R_f = 0.65;

^{1}H NMR (400 MHz, Chloroform-*d*) δ 7.27 (s, 1H), 6.80 (dd, J = 7.9, 5.0 Hz, 2H), 5.43 (d, J = 12.1 Hz, 1H), 5.19 - 5.03 (m, 1H), 3.63 (dd, J = 16.2, 8.4 Hz, 1H), 3.28 (s, 3H), 3.08 - 2.95 (m, 1H), 2.28 (dd, J = 16.3, 10.7 Hz, 1H), 1.51 (s, 3H), 1.09 (d, J = 6.4 Hz, 3H). **^{13}C NMR** (100 MHz, Chloroform-*d*) δ 207.4, 170.0, 139.2, 139.0, 138.3, 131.2, 130.9, 130.5, 124.5, 111.7, 63.4, 42.1, 30.6, 30.0, 21.9, 15.7. **IR**: ***v*** 3142, 2961, 2911, 1771, 1720, 1660, 1601, 1533, 1481, 1440, 1062 cm^{-1}; **HRMS** (m/z): $[M+H]^+$ calcd. for $C_{16}H_{18}NO_2^+$, 256.1333; found, 256.1332.

(6*R*,9*R*,*Z*)-9,12-dimethyl-6-propyl-6,9-dihydro-1,9-(epiminomethano)benzo[8]annulene-10,11(5*H*)-dione (2o)

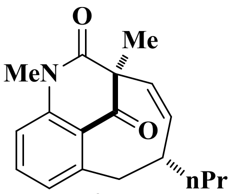

2o

2o was obtained (21.3 mg, 52% yield) as a white solid together with recycled **1o** (15.6 mg) following **the general procedure** Ⅳ from **1o** (41 mg, 0.145 mmol).

TLC (Hex : EA = 5:1): R_f = 0.7;

^{1}H NMR (400 MHz, Chloroform-*d*) δ 7.23 (s, 1H), 6.80 (t, J = 7.4 Hz, 2H), 5.49 (d, J = 10.6 Hz, 1H), 5.17 - 4.99 (m, 1H), 3.61 (dd, J = 16.2, 8.3 Hz, 1H), 3.28 (s, 3H), 3.02 - 2.76 (m, 1H), 2.29 (dd, J = 16.2, 10.7 Hz, 1H), 1.51 (s, 3H), 1.42 (ddd, J = 12.2, 7.5, 2.4 Hz, 1H), 1.38 - 1.28 (m, 3H), 0.87 (t, J = 7.1 Hz, 3H). **^{13}C NMR** (100 MHz, Chloroform-*d*) δ 207.5, 170.0, 138.9, 138.6, 138.2, 131.9, 131.4, 130.5, 124.5, 111.7,

63.3, 40.5, 39.1, 35.3, 30.6, 21.2, 15.7, 14.0. **IR**: ***v*** 2960, 2920, 2847, 1726, 1669, 1635, 1454, 1338, 1141, 1050, 751 cm^{-1}; **HRMS** (m/z): $[M+H]^+$ calcd. for $C_{18}H_{22}NO_2^+$, 284.1644; found, 284.1645. **mp**: 185-186 °C.

(*R,E*)-9,12-dimethyl-7-phenyl-6,9-dihydro-1,9-(epiminomethano)benzo[8]annulene-10,11(5*H*)-dione (2p)

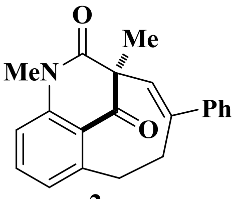

2p

2p was obtained (13 mg, 31% yield) as a colorless oil together with recycled **1p** (22.6 mg) following **the general procedure IV** from **1p** (42 mg, 0.132 mmol).

TLC (Hex : EA = 5:1): R_f = 0.6;

^{1}H NMR (400 MHz, Chloroform-*d*) δ 7.24 (d, J = 7.9 Hz, 1H), 7.21 - 7.16 (m, 3H), 6.89 (dd, J = 6.5, 3.0 Hz, 2H), 6.85 (d, J = 8.2 Hz, 1H), 6.81 (d, J = 7.6 Hz, 1H), 5.64 (s, 1H), 3.64 (dd, J = 15.7, 8.8 Hz, 1H), 3.33 (s, 3H), 3.11 - 2.95 (m, 1H), 2.88 - 2.75 (m, 1H), 2.69 (dd, J = 14.8, 7.5 Hz, 1H), 1.61 (s, 3H). **^{13}C NMR** (100 MHz, Chloroform-*d*) δ 206.7, 169.8, 144.3, 143.2, 138.9, 137.0, 131.4, 130.7, 129.5, 128.4, 127.7, 126.1, 124.5, 112.1, 63.7, 32.8, 30.7, 28.0, 15.4. **IR**: ***v*** 3011, 2934, 2900, 1726, 1643, 1521, 1464, 1330, 1247, 1131, 1022 cm^{-1}; **HRMS** (m/z): $[M+H]^+$ calcd. for $C_{21}H_{20}NO_2^+$, 318.1485; found, 318.1489.

(6*R*,12a*R,Z*)-6,13-dimethyl-2,3,4,6,12,12a-hexahydro-7*H*-8,6-(epiminomethano)benzo[6,7]cycloocta[1,2-b]pyran-7,14-dione (2q)

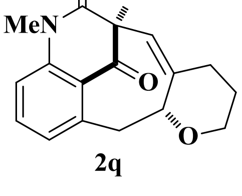

2q

2q was obtained (24 mg, 60% yield) as a faint yellow oil together with recycled **1q** (8.8 mg) following **the general procedure IV** from **1q** (40 mg, 0.135 mmol).

TLC (Hex : EA = 5:1): R_f = 0.6;

^{1}H NMR (400 MHz, Chloroform-*d*) δ 7.27 (d, J = 7.9 Hz, 1H), 6.90 (d, J = 7.6 Hz, 1H), 6.82 (d, J = 8.2 Hz, 1H), 5.20 (s, 1H), 5.02 (t, J = 9.4 Hz, 1H), 3.79 - 3.63 (m, 2H), 3.60 - 3.53 (m, 1H), 3.27 (s, 3H), 2.91 (dd, J = 15.8, 9.6 Hz, 1H), 1.97 - 1.87 (m, 1H), 1.67 - 1.59 (m, 3H), 1.53 (s, 3H). **^{13}C NMR** (100 MHz, Chloroform-*d*) δ 206.2, 169.6, 141.9, 138.8, 135.4, 131.5, 130.6, 124.8, 124.6, 112.4, 72.3, 62.9, 61.3, 36.4, 30.6, 30.4, 26.0, 15.4. **IR**: ***v*** 2923, 2857, 1731, 1667, 1585, 1488, 1460, 1333, 1228, 1039, 1008 cm^{-1}; **HRMS** (m/z): $[M+H]^+$ calcd. for $C_{18}H_{20}NO_3^+$, 298.1364; found, 298.1364.

(6*R*,9*R*,*Z*)-6-(furan-2-yl)-9,12-dimethyl-6,9-dihydro-1,9-(epiminomethano)benzo[8]annulene-10,11(5*H*)-dione (2r)

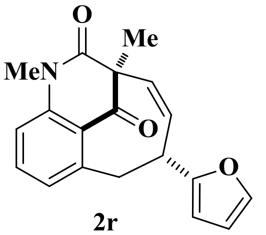

2r

2r was obtained (18.9 mg, 54% yield) as a colorless oil together with recycled **1r** (9.8 mg) following **the general procedure IV** from **1r** (35 mg, 0.114 mmol).

TLC (Hex : EA = 5:1): R_f = 0.55;

^{1}H NMR (400 MHz, Chloroform-*d*) δ 7.36 - 7.27 (m, 2H), 6.87 (dd, J = 7.8, 3.5 Hz, 2H), 6.38 - 6.21 (m, 1H), 6.16 - 6.02 (m, 1H), 5.57 (d, J = 11.6 Hz, 1H), 5.53 - 5.43 (m, 1H), 4.27 (q, J = 9.4 Hz, 1H), 3.86 (dd, J = 16.1, 8.6 Hz, 1H), 3.31 (s, 3H), 2.88 (dd, J = 16.2, 10.9 Hz, 1H), 1.57 (s, 3H). **^{13}C NMR** (100 MHz, Chloroform-*d*) δ 207.2, 169.6, 155.5, 142.0, 139.1, 136.7, 133.6, 132.1, 130.9, 124.8, 112.2, 110.3, 105.4, 63.3, 39.0, 35.1, 30.7, 15.6. **IR**: ***v*** 3002, 2929, 2847, 1728, 1677, 1635, 1587, 1553, 1451, 1338, 1045 cm^{-1}; **HRMS** (m/z): $[M+H]^+$ calcd. for $C_{19}H_{18}NO_3^+$, 308.1278; found, 308.1281.

(8*R*,14a*R*,*E*)-8,15-dimethyl-6,8,14,14a-tetrahydro-9*H*-10,8-(epiminomethano)ben

zo[4,5]cycloocta[1,2-c]chromene-9,16-dione (2s)

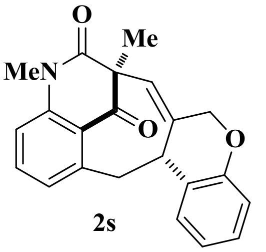

2s

2s was obtained (20.5 mg, 54% yield) as a faint yellow solid together with recycled **1s** (14.7 mg) following **the general procedure IV** from **1s** (38 mg, 0.110 mmol).

TLC (Hex : EA = 5:1): R_f = 0.65;

^{1}H NMR (400 MHz, Chloroform-*d*) δ 7.28 (d, J = 7.9 Hz, 1H), 7.13 (t, J = 7.5 Hz, 2H), 6.95 (t, J = 7.4 Hz, 1H), 6.86 (d, J = 8.2 Hz, 1H), 6.80 (dd, J = 7.5, 5.5 Hz, 2H), 5.62 (s, 1H), 4.21 - 4.12 (m, 1H), 4.08 (d, J = 12.1 Hz, 2H), 3.80 (d, J = 13.6 Hz, 1H), 3.32 (s, 3H), 2.79 (dd, J = 15.3, 9.5 Hz, 1H), 1.55 (s, 3H). **^{13}C NMR** (100 MHz, Chloroform-*d*) δ 206.0, 169.2, 153.0, 139.0, 138.0, 136.0, 130.8, 129.2, 128.5, 128.0, 124.6, 124.3, 121.6, 117.2, 112.6, 68.5, 63.0, 42.7, 34.5, 30.7, 15.4. **IR**: ***v*** 2926, 2820, 1727, 1675, 1590, 1467, 1344, 1234, 1066, 1039, 998 cm^{-1}; **HRMS** (m/z): [M+Na]$^+$ calcd. for $C_{22}H_{19}NO_3Na^+$, 368.1252; found, 368.1257. **mp**: 200-201 ^{o}C.

(6*R*,12b*S*,*E*)-6,15-dimethyl-12b,13-dihydro-4,6-(epiminomethano)benzo[4,5]cyclo octa[1,2-b]benzofuran-5,14(6*H*)-dione (2t)

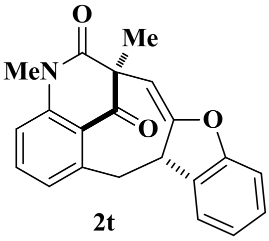

2t

2t was obtained (20.9 mg, 58% yield) as a faint yellow oil together with recycled **1t** (7.4 mg) following **the general procedure IV** from **1t** (36 mg, 0.109 mmol).

TLC (Hex : EA = 5:1): R_f = 0.65;

^{1}H NMR (400 MHz, Chloroform-*d*) δ 7.33 - 7.26 (m, 1H), 7.22 (d, J = 8.6 Hz, 1H), 7.16 (t, J = 7.8 Hz, 1H), 7.02 - 6.97 (m, 1H), 6.89 (d, J = 8.2 Hz, 1H), 6.78 (d, J = 8.0 Hz, 1H), 6.73 (d, J = 7.6 Hz, 1H), 5.30 (d, J = 1.7 Hz, 1H), 4.40 (d, J = 9.7 Hz, 2H),

3.35 (s, 3H), 2.65 - 2.56 (m, 1H), 1.54 (s, 3H). **^{13}C NMR** (100 MHz, Chloroform-*d*) δ 204.5, 169.0, 160.1, 155.4, 139.4, 135.2, 131.7, 131.5, 128.8, 128.6, 124.3, 123.9, 122.7, 112.4, 109.5, 106.2, 60.5, 42.3, 41.2, 30.7, 16.2. **IR**: ***v*** 2926, 2847, 1727, 1681, 1668, 1635, 1558, 1454, 1336, 1053, 701 cm^{-1}; **HRMS** (m/z): $[M+H]^+$ calcd. for $C_{21}H_{18}NO_3^+$, 332.1283; found, 332.1281.

(6*R*,12b*S*,*E*)-6,15-dimethyl-12b,13-dihydro-4,6-(epiminomethano)benzo[b]benzo[6,7]cycloocta[1,2-d]thiophene-5,14(6*H*)-dione (2u)

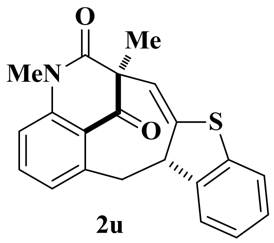

2u

2u was obtained (22.6 mg, 55% yield) as a faint yellow solid together with recycled **1u** (6.2 mg) following **the general procedure IV** from **1u** (41 mg, 0.118 mmol).

TLC (Hex : EA = 5:1): R_f = 0.65;

^{1}H NMR (400 MHz, Chloroform-*d*) δ 7.31 (t, J = 7.9 Hz, 1H), 7.17 (t, J = 6.4 Hz, 2H), 7.10 (d, J = 7.8 Hz, 2H), 6.89 (d, J = 8.2 Hz, 1H), 6.81 (d, J = 7.6 Hz, 1H), 5.59 - 5.51 (m, 1H), 4.65 - 4.45 (m, 1H), 4.08 (dd, J = 15.9, 9.8 Hz, 1H), 3.33 (s, 3H), 2.56 (dd, J = 15.9, 9.1 Hz, 1H), 1.56 (s, 3H). **^{13}C NMR** (100 MHz, Chloroform-*d*) δ 205.5, 168.7, 146.0, 142.9, 138.7, 137.4, 135.5, 131.4, 129.6, 128.2, 125.3, 124.7, 124.1, 122.0, 121.7, 112.4, 64.0, 47.6, 42.9, 30.7, 15.8. **IR**: ***v*** 2973, 2931, 1729, 1677, 1587, 1463, 1338, 1280, 1049, 889, 769 cm^{-1}; **HRMS** (m/z): $[M+H]^+$ calcd. for $C_{21}H_{18}NO_2S^+$, 348.1053; found, 348.1053. **mp**: 190-191 ^{o}C.

(6*R*,10*S*,11a*R*,*Z*)-6,14-dimethyl-10-(prop-1-en-2-yl)-8,9,10,11,11a,12-hexahydro-4,6-(epiminomethano)dibenzo[a,d][8]annulene-5,13(6*H*)-dione (2v)

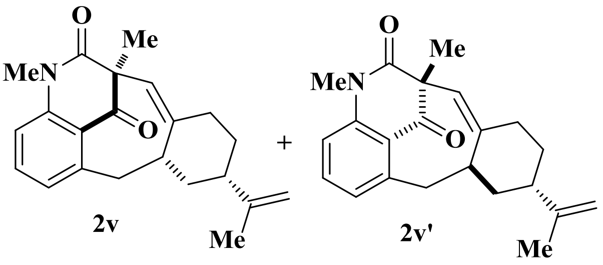

2v and 2v' was obtained (38.7 mg, 86% yield) as inseparable isomers (1:1 dr) following **the general procedure IV** from **1v** (45 mg, 0.134 mmol).

TLC (Hex : EA = 5:1): R_f = 0.75;

^{1}H NMR (400 MHz, Chloroform-*d*) δ 7.22 (dt, J = 7.9, 3.9 Hz, 2H), 6.86 (d, J = 7.6 Hz, 1H), 6.83 - 6.76 (m, 3H), 5.24 (s, 1H), 5.13 (s, 1H), 4.73 - 4.60 (m, 4H), 3.65 (dd, J = 16.0, 8.5 Hz, 1H), 3.38 (dd, J = 15.7, 8.3 Hz, 1H), 3.27 (s, 6H), 3.25 - 3.18 (m, 1H), 3.06 (dd, J = 17.0, 8.4 Hz, 1H), 2.73 (dd, J = 15.7, 11.5 Hz, 1H), 2.48 (dd, J = 16.1, 10.5 Hz, 1H), 2.10 (t, J = 12.4 Hz, 1H), 1.88 (dt, J = 12.8, 7.1 Hz, 2H), 1.82 - 1.72 (m, 1H), 1.69 (d, J = 4.7 Hz, 6H), 1.67 - 1.59 (m, 3H), 1.52 (d, J = 5.5 Hz, 6H), 1.49 - 1.37 (m, 4H), 1.33 - 1.22 (m, 3H), 1.17 (dd, J = 12.9, 4.2 Hz, 1H). **^{13}C NMR** (100 MHz, Chloroform-*d*) δ 207.2, 170.0, 149.3, 145.9, 145.7, 137.4, 137.3, 130.4, 129.9, 125.5, 124.4, 124.2, 124.0, 112.1, 111.7, 109.2, 108.9, 63.2, 62.8, 40.3, 39.7, 39.4, 37.9, 35.8, 35.5, 35.4, 34.2, 34.1, 31.7, 30.6, 30.5, 30.3, 29.2, 21.0, 21.0, 16.0, 15.9. **IR**: ***v*** 2929, 2850, 1728, 1670, 1646, 1457, 1333, 1276, 1090, 1045, 748 cm^{-1}; **HRMS** (m/z): $[M+H]^+$ calcd. for $C_{22}H_{26}NO_2^+$, 336.1959; found, 336.1958.

(6*R*,9*R*,*Z*)-9,12-dimethyl-6-phenyl-6,9-dihydro-1,9-(epiminomethano)benzo[8]annulene-10,11(5*H*)-dione-6-d (2a-D)

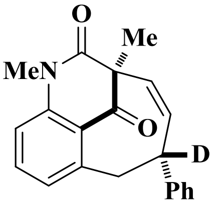

2a-D

2a-D was obtained (35.1 mg, 78% yield) as a faint yellow oil following **the general procedure IV** from **1a-D** (45 mg, 0.141 mmol).

TLC (Hex : EA = 5:1): R_f = 0.6;

^{1}H NMR (400 MHz, Chloroform-*d*) δ 7.32 (dt, J = 7.9, 4.1 Hz, 3H), 7.23 (dd, J = 9.8, 6.9 Hz, 3H), 6.88 (dd, J = 7.8, 3.7 Hz, 2H), 5.52 (t, J = 8.2 Hz, 2H), 3.86 (d, J = 16.2 Hz, 1H), 3.32 (s, 3H), 2.81 (d, J = 16.3 Hz, 1H), 1.59 (s, 3H). **^{13}C NMR** (100 MHz, Chloroform-*d*) δ 207.4, 169.8, 143.4, 139.1, 137.5, 137.1, 131.4, 130.8, 129.0, 127.6, 127.0, 124.7, 112.1, 63.4, 42.6, 30.7, 15.8. **IR**: ***v*** 2923, 2850, 1734, 1711, 1646, 1557, 1451, 1376, 1045, 1011, 746 cm^{-1}; **HRMS** (m/z): $[M+H]^+$ calcd. for $C_{21}H_{19}DNO_2^+$, 319.1520; found, 319.1521.

4-methyl-4,9b,10,11-tetrahydro-5*H*-benzo[e]indeno[7,1-bc]azepin-5-one (7)

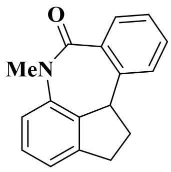

7

7 was obtained (54.7 mg, 76% yield) as a white solid following **the general procedure IV** from **6** (80 mg, 0.29 mmol).

TLC (Hex : EA = 5:1): R_f = 0.7;

^{1}H NMR (400 MHz, Chloroform-*d*) δ 7.82 - 7.74 (m, 1H), 7.36 (td, J = 7.6, 1.2 Hz, 1H), 7.27 (d, J = 7.7 Hz, 1H), 7.22 (t, J = 7.5 Hz, 1H), 7.14 (t, J = 7.7 Hz, 1H), 7.03 - 6.97 (m, 2H), 4.48 - 4.36 (m, 1H), 3.65 (s, 3H), 3.10 (d, J = 4.3 Hz, 1H), 3.07 (d, J = 4.4 Hz, 1H), 2.78 (dt, J = 13.9, 4.4 Hz, 1H), 2.50 - 2.34 (m, 1H). **^{13}C NMR** (100 MHz, Chloroform-*d*) δ 169.6, 143.2, 141.8, 141.0, 138.3, 134.0, 132.0, 131.1, 127.9, 126.6, 121.4, 120.7, 118.3, 44.9, 36.2, 31.8, 25.4. **IR**: ***v*** 2926, 1737, 1631, 1587, 1540, 1447, 1382, 1353, 1321, 1122, 1033 cm^{-1}; **HRMS** (m/z): $[M+H]^+$ calcd. for $C_{17}H_{16}NO^+$, 250.1226; found, 250.1226. **mp**: 160-161 ^{o}C.

Ⅳ. *Procedure for cascade rearrangement and characterization of compounds (**5a**,**5g**, **5i**, **5l**, **5n** and **5a-D**)*

General procedure V

To a solution of substrates (1.0 equiv) in trifluoroacetic acid was slowly added H_2SO_4 (5 drops) at 0 °C with stirring. The reaction was stirred for several minutes at 0 °C. NaN_3 (3.0 equiv) was added to the mixture then cooling bath was removed and the reaction was stirred at room temperature for an 1.0 hours. After the starting material was consumed, the reaction was quenched by saturated NH_4Cl aqueous solution and extracted with DCM (3 × 10 mL) and washed by aqueous solution of $NaHCO_3$ to removed residual trifluoroacetic acid. Then the combined organic layers were dried over $MgSO_4$ and concentrated to give a yellow oil. The crude product was purified by flash chromatography to give desired product.

(2a*R*,2a1*S*,9a*S*)-2a,4-dimethyl-9a-phenyl-2a,2a1,4,9a-tetrahydro-3*H*-benzo[ij]cyclopenta[de][2,7]naphthyridin-3-one (5a)

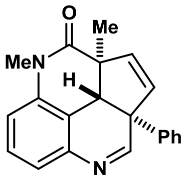

5a

5a was obtained (14 mg, 60% yield) as a faint yellow oil following **the general procedure V** from **2a** (24 mg, 0.076 mmol).

TLC (Hex : EA = 5:1): R_f = 0.2;

^{1}H NMR (400 MHz, Chloroform-*d*) δ 7.64 (s, 1H), 7.44 - 7.38 (m, 2H), 7.35 - 7.28 (m, 4H), 7.22 (d, J = 7.7 Hz, 1H), 6.89 (d, J = 8.2 Hz, 1H), 6.12 (d, J = 5.5 Hz, 1H), 5.90 (d, J = 5.6 Hz, 1H), 3.71 (s, 1H), 3.32 (s, 3H), 1.07 (s, 3H). **^{13}C NMR** (100 MHz, Chloroform-*d*) δ 171.9, 162.1, 145.7, 138.9, 138.4, 129.3, 128.7, 127.7, 127.7, 122.7, 113.7, 110.1, 58.5, 56.1, 48.4, 30.6, 25.0. **IR**: ***v*** 2926, 2850, 1655, 1629, 1533, 1480, 1299, 1150, 1090, 1045, 762 cm^{-1}; **HRMS** (m/z): $[M+H]^+$ calcd. for $C_{21}H_{19}N_2O^+$, 315.1488; found, 315.1492.

(2a*S*,2a1*S*,9a*S*)-2,4-dimethyl-9a-phenyl-2a,2a1,4,9a-tetrahydro-3*H*-benzo[ij]cyclopenta[de][2,7]naphthyridin-3-one (5g)

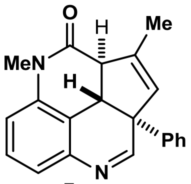

5g

5g was obtained (11 mg, 56% yield) as a colorless solid following **the general procedure V** from **2g** (20 mg, 0.063 mmol).

TLC (Hex : EA = 5:1): R_f = 0.15;

^{1}H NMR (400 MHz, Chloroform-*d*) δ 8.03 (s, 1H), 7.44 - 7.38 (m, 2H), 7.35 (t, J = 5.5 Hz, 3H), 7.32 - 7.29 (m, 2H), 6.95 (d, J = 9.2 Hz, 1H), 5.44 (s, 1H), 3.70 (d, J = 5.6 Hz, 1H), 3.63 (s, 1H), 3.36 (s, 3H), 1.89 (s, 3H). **^{13}C NMR** (100 MHz, Chloroform-*d*) δ 167.7, 164.1, 142.8, 141.8, 138.6, 129.1, 128.7, 127.9, 127.4, 123.1, 122.5, 114.2, 111.1, 56.9, 52.8, 43.8, 29.9, 15.3. **IR**: ***v*** 2960, 2920, 2844, 1658, 1595, 1471, 1361, 1256, 1146, 1041, 802 cm^{-1}; **HRMS** (m/z): $[M+H]^+$ calcd. for $C_{21}H_{19}N_2O^+$, 315.1448; found, 315.1452. **mp**: 220-221 ^{o}C.

(2a*R*,2a1*S*,9a*S*)-4-benzyl-2a-methyl-9a-phenyl-2a,2a1,4,9a-tetrahydro-3*H*-benzo[ij]cyclopenta[de][2,7]naphthyridin-3-one (5i)

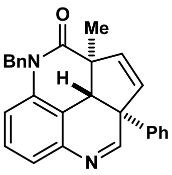

5i

5i was obtained (18.4 mg, 62% yield) as a colorless oil following **the general procedure V** from **2i** (30 mg, 0.08 mmol).

TLC (Hex : EA = 5:1): R_f = 0.25;

^{1}H NMR (400 MHz, Chloroform-*d*) δ 7.63 (s, 1H), 7.41 (d, J = 7.3 Hz, 2H), 7.37 - 7.27 (m, 5H), 7.23 (d, J = 7.3 Hz, 1H), 7.19 - 7.11 (m, 2H), 6.74 (d, J = 9.4 Hz, 1H), 6.22 (d, J = 5.6 Hz, 1H), 5.98 (d, J = 5.6 Hz, 1H), 5.18 (q, J = 16.5, 14.3 Hz, 2H), 3.79 (s, 1H), 1.13 (s, 3H). **^{13}C NMR** (100 MHz, Chloroform-*d*) δ 171.7, 161.7, 145.8, 141.2, 138.2, 137.8, 136.4, 129.3, 128.9, 128.6, 128.2, 127.6, 127.2, 126.4, 123.0, 115.0, 110.2, 58.7, 56.0, 48.6, 46.5, 24.9. **IR**: ***v*** 2954, 2925, 2872, 1664, 1601, 1454,

1375, 1229, 1149, 980, 762 cm^{-1}; **HRMS** (m/z): $[M+H]^+$ calcd. for $C_{27}H_{23}N_2O^+$, 391.1800; found, 391.1805.

(2a*R*,2a1*S*,9a*S*)-2a,4,6-trimethyl-9a-phenyl-2a,2a1,4,9a-tetrahydro-3*H*-benzo[ij]cyclopenta[de][2,7]naphthyridin-3-one (5l)

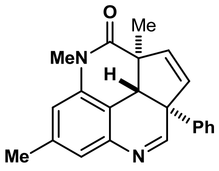

5l

5l was obtained (12.6 mg, 51% yield) as a colorless oil following t**he general procedure V** from **2l** (25 mg, 0.08 mmol).

TLC (Hex : EA = 5:1): R_f = 0.2;

^{1}H NMR (400 MHz, Chloroform-*d*) δ 7.64 (s, 1H), 7.43 - 7.38 (m, 2H), 7.34 - 7.29 (m, 3H), 7.06 (s, 1H), 6.71 (s, 1H), 6.11 (d, J = 5.6 Hz, 1H), 5.89 (d, J = 5.6 Hz, 1H), 3.66 (s, 1H), 3.30 (s, 3H), 2.38 (s, 3H), 1.06 (s, 3H). **^{13}C NMR** (100 MHz, Chloroform-*d*) δ 172.0, 162.4, 145.6, 138.9, 138.7, 138.5, 129.3, 127.7, 127.6, 123.1, 114.7, 107.2, 58.6, 56.1, 48.2, 30.6, 24.8, 21.6. **IR**: ***v*** 3111, 2945, 2902, 1729, 1635, 1589, 1451, 1323, 1095, 1012, 792 cm^{-1}; **HRMS** (m/z): $[M+H]^+$ calcd. for $C_{22}H_{21}N_2O^+$, 329.1646; found, 329.1648.

(2a*R*,2a1*S*,9a*S*)-2a,4,9a-trimethyl-2a,2a1,4,9a-tetrahydro-3*H*-benzo[ij]cyclopenta[de][2,7]naphthyridin-3-one (5n)

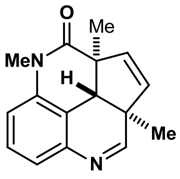

5n

5n was obtained (12.6 mg, 51% yield) as a faint yellow oil following **the general procedure V** from **2n** (25 mg, 0.1 mmol).

TLC (Hex : EA = 5:1): R_f = 0.2;

^{1}H NMR (400 MHz, Chloroform-*d*) δ 7.67 (s, 1H), 7.26 (td, J = 8.1, 0.9 Hz, 1H), 7.16

(d, J = 7.8 Hz, 1H), 6.83 (d, J = 8.9 Hz, 1H), 5.87 (d, J = 5.5 Hz, 1H), 5.49 (d, J = 5.5 Hz, 1H), 3.29 (s, 3H), 3.16 (s, 1H), 1.70 (s, 3H), 1.59 (s, 3H). **^{13}C NMR** (100 MHz, Chloroform-*d*) δ 172.5, 165.5, 139.1, 135.8, 130.8, 128.8, 122.3, 113.6, 109.9, 56.7, 50.3, 46.9, 30.7, 28.5, 27.2. **IR**: ***v*** 3001, 2955, 1732, 1656, 1579, 1523, 1473, 1366, 1200, 1112, 967 cm^{-1}; **HRMS** (m/z): [M+H]$^+$ calcd. for $C_{16}H_{17}N_2O^+$, 253.1332; found, 253.1335.

(2a*R*,2a1*S*,9a*S*)-2a,4-dimethyl-9a-phenyl-2a,2a1,4,9a-tetrahydro-3*H*-benzo[ij]cyclopenta[de][2,7]naphthyridin-3-one-2a1-d (5a-D)

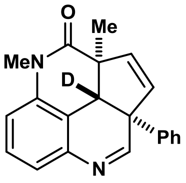

5a-D

5a-D was obtained (12.7 mg, 64% yield) as a faint yellow oil following **the general procedure V** from **2a-D** (20 mg, 0.063 mmol).

TLC (Hex : EA = 5:1): R_f = 0.2;

^{1}H NMR (400 MHz, Chloroform-*d*) δ 7.63 (s, 1H), 7.40 (t, J = 7.2 Hz, 2H), 7.31 (t, J = 8.4 Hz, 4H), 7.20 (d, J = 7.5 Hz, 1H), 6.88 (d, J = 8.1 Hz, 1H), 6.12 (s, 1H), 5.90 (s, 1H), 3.31 (s, 3H), 1.07 (s, 3H). **^{13}C NMR** (100 MHz, Chloroform-*d*) δ 172.0, 162.1, 145.7, 140.9, 138.9, 138.3, 129.3, 128.7, 127.8, 127.6, 122.8, 113.7, 58.5, 56.0, 30.6, 24.9. **IR**: ***v*** 3012, 2924, 1756, 1612, 1556, 1521, 1464, 1366, 1232, 1102, 964 cm^{-1}; **HRMS** (m/z): [M+H]$^+$ calcd. for $C_{21}H_{18}DN_2O^+$, 316.1551; found, 316.1551.

<u>Applications</u>

(6*S*,9*S*)-6,9,12-trimethyl-6,7,8,9-tetrahydro-1,9-(epiminomethano)benzo[8]annulene-10,11(5*H*)-dione (8)

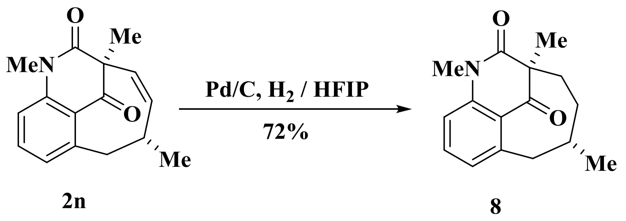

To a 10 ml flame-dried flask equipped with a stirer and a N_2 balloon was added HFIP (5 mL), Pd/C (12.7 mg, 0.2 equiv, 0.012 mmol, 10 % on carbon) and **2n** (20 mg, 1.0 equiv, 0.06 mmol). The system was ventilated with hydrogen gas three times and the mixture was stirred at rt for overnight. Upon completion, the reaction mixture was filtered through a glass frit (elution with dichloromethane and acetone) and the solvent was removed under reduced pressure. Then the crude product was purified by flash column chromatography to afford the product **8** (5.2 mg, 30% yield) as a colorless oil.

TLC (Hex : EA = 5:1): R_f = 0.7;

^{1}H NMR (400 MHz, Chloroform-d) δ 7.28 (d, J = 7.9 Hz, 1H), 6.84 (dd, J = 14.2, 7.9 Hz, 2H), 3.56 (dd, J = 13.4, 2.7 Hz, 1H), 3.29 (s, 3H), 2.30 - 2.12 (m, 2H), 1.78 - 1.62 (m, 3H), 1.50 (ddd, J = 13.8, 8.4, 2.0 Hz, 1H), 1.38 (s, 3H), 0.40 (d, J = 7.1 Hz, 3H). **^{13}C NMR** (100 MHz, Chloroform-d) δ 203.7, 171.9, 140.9, 138.5, 131.1, 127.8, 125.6, 111.6, 59.9, 37.3, 33.7, 30.6, 30.1, 30.0, 16.7, 14.3. **IR**: ***v*** 2923, 2850, 1734, 1711, 1646, 1557, 1451, 1376, 1045, 1011, 746 cm^{-1}; **HRMS** (m/z): $[M+H]^+$ calcd. for $C_{16}H_{20}O_2N^+$, 258.1488; found, 258.1488.

(6*R*,9*S*,10*R*,*Z*)-10-hydroxy-9,12-dimethyl-6-phenyl-5,6,9,10-tetrahydro-1,9-(epiminomethano)benzo[8]annulen-11-one (9)

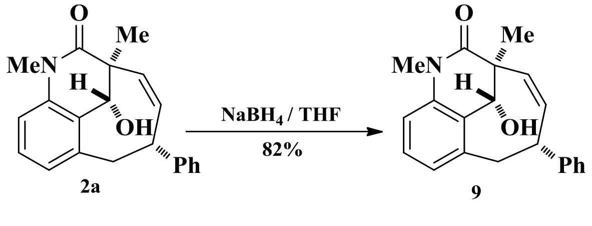

To a 10 ml flame-dried flask equipped with a stirer and a N_2 balloon was added THF (5 ml) and **2a** (40 mg, 1.0 equiv, 0.13 mmol). The system was cooled to 0 ℃ and $NaBH_4$ (7.2 mg, 1.5 equiv, 0.2 mmol) was added. Stirring was continued stirred for 5

mins at the same temperature. Then the reaction was warmed up to rt and stirred for 1.5 h. The reaction was monitored by TLC. Upon completion, the reaction was quenched by slow addition of HCl (1 mL, 2M) and extracted with EtOAc (20 mL×3). The organic layer was dried over Na_2SO_4, filtered, was purified by flash column chromatography to afford the product **9** (34 mg, 82% yield) as a colorless oil.

TLC (Hex : EA = 5:1): R_f = 0.3;

^{1}H NMR (400 MHz, Chloroform-d) δ 7.31 (dd, J = 8.0, 7.1 Hz, 2H), 7.24 - 7.16 (m, 3H), 7.14 (t, J = 7.7 Hz, 1H), 6.86 - 6.78 (m, 2H), 5.38 (dd, J = 11.8, 7.5 Hz, 1H), 5.06 (s, 1H), 5.03 - 4.97 (m, 1H), 4.93 - 4.80 (m, 1H), 4.22 (dd, J = 14.9, 10.7 Hz, 1H), 3.32 (s, 3H), 2.69 (dd, J = 14.8, 7.9 Hz, 1H), 1.45 (s, 3H). **^{13}C NMR** (100 MHz, Chloroform-d) δ 176.1, 148.0, 138.6, 138.5, 136.4, 130.7, 130.5, 128.8, 128.0, 126.7, 126.2, 125.4, 111.8, 72.7, 53.7, 45.9, 45.8, 30.6, 17.8. **IR**: ***v*** 2931, 2885, 1660, 1590, 1467, 1342, 1300, 1090, 1052, 765, 730 cm^{-1}; **HRMS** (m/z): $[M-H]^-$ calcd. for $C_{21}H_{20}O_2N^-$, 318.1497; found, 318.1500.

(6*R*,9*R*,10*S*,*Z*)-9,12-dimethyl-6-phenyl-5,6,9,10-tetrahydro-1,9-(epiminomethano) benzo[8]annulen-10-ol (10)

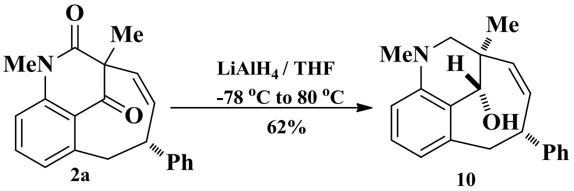

To a 10 ml flame-dried flask equipped with a stirer and a N_2 balloon was added THF (5 ml) and **2a** (45 mg, 1.0 equiv, 0.14 mmol). The system was cooled to -78 ℃ and $LiAlH_4$ (42.5 mg, 8.0 equiv, 1.12 mmol) was added. Stirring was continued stirred for 10 mins at the same temperature. Then the reaction was warmed up to 80 ^{o}C and stirred overnight. Upon completion, the reaction was quenched by slow addition of MeOH (5 mL) and aqueous HCl (1N, 5 ml), and extracted with EtOAc (20 mL×3). The organic layer was dried over Na_2SO_4, filtered, was purified by flash column chromatography to afford the product **10** (26.8 mg, 62% yield) as a colorless oil.[15]

TLC (Hex : EA = 5:1): R_f = 0.6;

^{1}H NMR (400 MHz, Chloroform-d) δ 7.34 - 7.26 (m, 4H), 7.20 (d, J = 6.8 Hz, 1H), 7.06 (t, J = 7.7 Hz, 1H), 6.62 - 6.54 (m, 2H), 5.35 - 5.13 (m, 1H), 4.99 - 4.79 (m, 3H), 4.09 (dd, J = 14.7, 10.7 Hz, 1H), 3.23 (d, J = 10.1 Hz, 1H), 2.80 (s, 3H), 2.66 (dd, J = 10.1, 1.0 Hz, 1H), 2.58 (dd, J = 14.8, 8.0 Hz, 1H), 2.39 (d, J = 4.2 Hz, 1H), 1.33 (s, 3H). **^{13}C NMR** (100 MHz, Chloroform-d) δ 148.9, 147.0, 140.4, 137.7, 132.5, 128.7, 128.7, 128.1, 126.3, 125.9, 121.2, 109.7, 74.7, 69.5, 47.9, 45.4, 45.0, 37.4, 22.4. **IR**: ***v*** 3431, 2934, 2867, 1600, 1587, 1470, 1360, 1301, 1223, 1082, 1001, 760 cm^{-1}; **HRMS** (m/z): $[M+H]^+$ calcd. for $C_{21}H_{24}ON^+$, 306.1850; found, 306.1852.

(6*R*,9*S*,10*R*,*Z*)-10-hydroxy-9,12-dimethyl-6-phenyl-10-vinyl-5,6,9,10-tetrahydro-1,9-(epiminomethano)benzo[8]annulen-11-one (11)

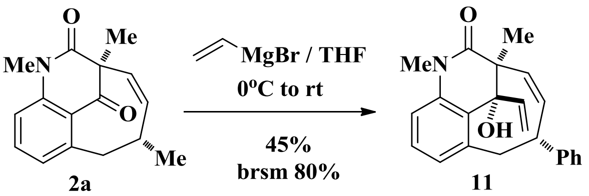

To a 10 ml flame-dried flask equipped with a stirer and a N_2 balloon was added THF (5 ml) and **2a** (20 mg, 1.0 equiv, 0.06 mmol). The system was cooled to 0 ℃ and Vinylmagnesium bromide (0.076 mL, 1.2 equiv, 0.076 mmol, 1M in THF) was added. Stirring was continued stirred for 15 mins at the same temperature. Then the reaction was warmed up to rt and stirred for 1.5 h. The reaction was monitored by TLC. Upon completion, the reaction was quenched by slow addition of H_2O (1 mL) and extracted with EtOAc (20 mL×3). The organic layer was dried over Na_2SO_4, filtered, was purified by flash column chromatography to afford the product **11** (9.3 mg, 45% yield) as a colorless oil.

TLC (Hex : EA = 5:1): R_f = 0.4;

^{1}H NMR (400 MHz, Chloroform-d) δ 7.29 (t, J = 7.5 Hz, 2H), 7.22 - 7.09 (m, 4H), 6.86 (d, J = 7.9 Hz, 1H), 6.78 (d, J = 7.5 Hz, 1H), 6.47 (dd, J = 17.2, 10.5 Hz, 1H), 5.46 - 5.23 (m, 2H), 5.17 - 5.03 (m, 2H), 4.72 (dtd, J = 11.5, 5.8, 2.5 Hz, 1H), 4.46 (dd, J = 14.5, 11.6 Hz, 1H), 3.26 (s, 3H), 2.69 (dd, J = 14.6, 5.7 Hz, 1H), 1.43 (s, 3H). **^{13}C NMR** (100 MHz, Chloroform-d) δ 173.8, 149.7, 142.7, 139.0, 136.4, 132.5, 131.6,

128.8, 128.0, 127.1, 126.0, 125.8, 116.0, 113.0, 56.2, 47.2, 47.1, 30.2, 14.8. **IR**: ***ν*** 2931, 2852, 1587, 1567, 1420, 1367, 1311, 1254, 1032, 1011, 730 cm^{-1}; **HRMS** (m/z): $[M-H]^-$ calcd. for $C_{23}H_{22}O_2N^-$, 344.1651; found, 344.1656.

(2*R*,6'*R*,9'*S*,*Z*)-3',9',12'-trimethyl-6'-phenyl-6',9'-dihydro-5'*H*-spiro[oxirane-2,10'-[1,9](epiminomethano)benzo[8]annulen]-11'-one (12)

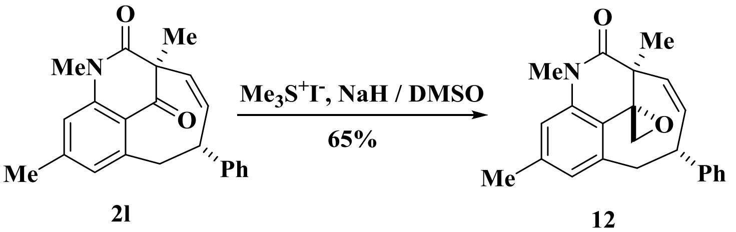

To a suspension of NaH (2.7 mg, 1.0 equiv, 0.07 mmol, 60%) in dry DMSO (5 mL) at the room temperature was added $Me_3S^+I^-$ (15 mg, 1.05 equiv, 0.074 mmol) over a period of 15 mins. The mixture was allowed to stir at rt over 45 mins, a solution of **2l** (20 mg, 0.9 equiv, 0.06 mmol) in DMSO (5 mL) was added via a dropping funnel to the mixture. After 15 mins stirring at rt, the mixture was heated to 65 ℃ for 2 h. The reaction was monitored by TLC. Upon completion, H_2O (5 mL) was added to the mixture and extracted with EtOAc (20 mL×3). The organic layer was dried over Na_2SO_4, filtered, was purified by flash column chromatography to afford the product **12** (13.5 mg, 65% yield) as a colorless oil.

TLC (Hex : EA = 5:1): R_f = 0.5;

^{1}H NMR (400 MHz, Chloroform-d) δ 7.36 - 7.31 (m, 4H), 7.25 - 7.21 (m, 1H), 6.65 (d, J = 18.9 Hz, 2H), 5.48 - 5.39 (m, 1H), 5.21 (d, J = 12.5 Hz, 1H), 4.73 (d, J = 9.1 Hz, 1H), 3.99 (dd, J = 15.2, 9.7 Hz, 1H), 3.35 (s, 3H), 3.23 (d, J = 5.4 Hz, 1H), 2.81 (d, J = 5.4 Hz, 1H), 2.69 (dd, J = 15.2, 9.6 Hz, 1H), 2.35 (s, 3H), 1.21 (s, 3H). **^{13}C NMR** (100 MHz, Chloroform-d) δ 174.8, 146.2, 140.4, 139.1, 137.8, 135.9, 131.2, 128.9, 128.0, 126.5, 125.3, 112.7, 59.9, 51.3, 50.6, 44.6, 43.7, 30.6, 21.7, 14.3. **IR**: ***ν*** 2944, 2890, 1657, 1546, 1453, 1342, 1304, 1192, 1076, 765, 729 cm^{-1}; **HRMS** (m/z): $[M+H]^+$ calcd. for $C_{23}H_{24}O_2N^+$, 346.1800; found, 346.1802.

(7*R*,9*aS*)-2,9a-dimethyl-7-phenyl-2,6,7,9a-tetrahydro-1*H*-cyclohepta[cd]indol-1-one (13)

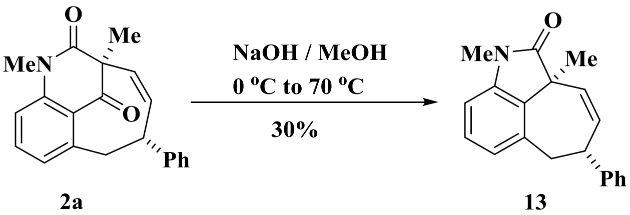

To a 10 ml flame-dried flask equipped with a stirer and a N_2 balloon was added MeOH (5 mL) and **2a** (20 mg, 1.0 equiv, 0.06 mmol). The system was cooled to 0 ℃ and NaOH (7.6 mg, 3.0 equiv, 0.18 mmol) was added. Stirring was continued stirred for 5 mins at the same temperature. Then the reaction was warmed up to 70 ℃ and stirred overnight. Upon completion, the reaction was quenched by slow addition of HCl (1 mL, 2M) and extracted with EtOAc (20 mL×3). The organic layer was dried over Na_2SO_4, filtered, was purified by flash column chromatography to afford the product **13** (5.2 mg, 30% yield) as a colorless oil.

TLC (Hex : EA = 5:1): R_f = 0.7;

^{1}H NMR (400 MHz, Chloroform-d) δ 7.07 (dt, J = 6.4, 3.4 Hz, 3H), 6.98 - 6.88 (m, 1H), 6.75 - 6.60 (m, 3H), 6.25 (dd, J = 7.5, 4.6 Hz, 1H), 6.11 - 6.02 (m, 1H), 5.72 - 5.61 (m, 1H), 3.94 (dd, J = 12.3, 4.2 Hz, 2H), 3.24 (s, 3H), 2.77 - 2.65 (m, 1H), 1.64 (s, 3H). **^{13}C NMR** (100 MHz, Chloroform-d) δ 180.0, 141.9, 141.6, 135.2, 132.6, 131.8, 128.8, 128.2, 127.9, 127.6, 126.4, 123.9, 106.2, 49.4, 44.8, 38.5, 26.6, 25.3. **IR**: ***v*** 2930, 2885, 1658, 1567, 1459, 1337, 1297, 1086, 1041, 763, 729 cm^{-1}; **HRMS** (m/z): $[M+H]^+$ calcd. for $C_{20}H_{20}O_2N^+$, 290.1541; found, 290.1539.

(*E*)-4-methoxy-5-((2-methyl-4-phenylpenta-2,4-dien-1-yl)oxy)bicyclo[4.2.0]octa-1,3,5-trien-7-one (6)

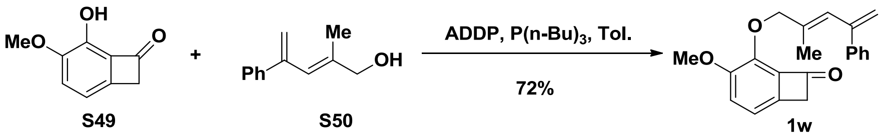

To a solution of **S49** (0.1 g, 1.5 equiv, 0.75 mmol), **S50** (0.07 g, 1.0 equiv, 0.41 mmol) in toluene (20 mL) at r.t was added $P(n\text{-}Bu)_3$ (0.2 mL, 1.5 equiv, 0.75 mmol). Then

the reaction was cooled to 0 ℃, ADDP (0.19 g, 1.5 equiv, 0.75 mmol) was added. After that the mixture was warmed to rt and stirred for 12 h. Upon completion, the reaction was filtered through a pad of celite and washed with n-hexane (3 × 20 mL). The solvent was removed under reduced pressure. Then the crude product was purified by using silica gel flash chromatography gave compound **1w** (94.5 mg, 72%) as a colorless oil..

R_f = 0.6 (Hex : EA= 5:1)

^{1}H NMR (400 MHz, Chloroform-*d*) δ 7.29 (ddd, *J* = 16.2, 10.8, 4.7 Hz, 5H), 7.06 (d, *J* = 7.6 Hz, 1H), 6.95 (d, *J* = 7.7 Hz, 1H), 6.28 (s, 1H), 5.59 (s, 1H), 5.14 (s, 1H), 5.04 (s, 2H), 3.86 (s, 5H), 1.81 (s, 3H). **^{13}C NMR** (100 MHz, Chloroform-*d*) δ 184.9, 148.7, 144.2, 142.4, 141.0, 140.7, 135.0, 133.4, 128.3, 127.6, 126.9, 126.6, 120.3, 115.6, 114.9, 57.2, 50.2, 15.5. **IR**: ***v*** 2921, 2850, 1759, 1598, 1488, 1437, 1256, 1194, 1177, 1045, 982 cm^{-1}. **HRMS** (m/z): calcd. for $C_{21}H_{19}O_3^-$ [M-H]$^-$: 319.1333; found: 319.1340.

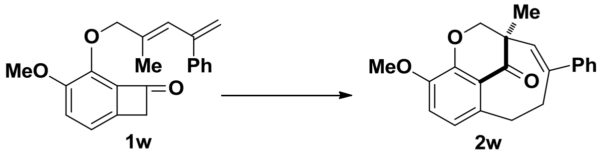

2w was obtained (3.8 mg, 12% yield) as colorless oil following **the general procedure II** from **1w** (32 mg, 0.1 mmol).

R_f = 0.35 (Hex : EA= 5:1)

^{1}H NMR (400 MHz, Chloroform-*d*) δ 7.19 (dd, *J* = 5.1, 1.8 Hz, 3H), 6.95 - 6.90 (m, 2H), 6.70 (d, *J* = 8.1 Hz, 1H), 6.56 (d, *J* = 8.2 Hz, 1H), 5.69 (s, 1H), 4.62 (d, *J* = 10.5 Hz, 1H), 4.30 (d, *J* = 10.5 Hz, 1H), 3.82 (s, 3H), 3.59 - 3.44 (m, 1H), 3.00 - 2.88 (m, 1H), 2.72 - 2.64 (m, 1H), 2.65 - 2.53 (m, 1H), 1.37 (s, 3H). **^{13}C NMR** (100 MHz, Chloroform-*d*) δ 209.2, 143.5, 140.4, 132.7, 130.6, 128.4, 127.6, 126.6, 126.0, 122.8, 120.4, 113.7, 85.8, 56.6, 51.8, 47.1, 41.4, 17.1. **IR**: ***v*** 3031, 2922, 1762, 1598, 1498, 1440, 1374, 1200, 983, 703, 700 cm^{-1}; **HRMS** (m/z): calcd. for $C_{21}H_{21}O_3^+$ [M+H]$^+$: 321.1489; found: 321.1485.

Supplementary References

[1] Deng, L.; Xu, T.; Li, H.; Dong, G. *J. Am. Chem. Soc.* **2016**, *138*, 369.

[2] Xu, Z.; Hu, W.; Liu, Q.; Zhang, L.; Jia, Y. *J. Org. Chem.* **2010**, *75*, 7626.

[3] Chen, P. H.; Savage, N. A.; Dong, G. *Tetrahedron* **2014**, 70, 4135.

[4] Qiu, Bo.; Li, X.T.; Xu, T. *Org. Lett.* **2018**, 20, 7689.

[5] Xu, T.; Ko, H.M.; Savage, N. A.; Dong, G. *J. Am. Chem. Soc.* **2012**, *134*, 20005.

[6] Deng, L.; Chen, M.Q.; Dong, G. *J. Am. Chem. Soc.* **2018**, 140, 30, 9652.

[7] Beutner, Gregory L.; Young. Ian S. *Org. Lett.* **2018**, 20, 14, 4218.

[8] Sahner, J. Henning.; Müller, Rolf.; Hartmann, Rolf W. *ChemBioChem* **2015**, 16, 946.

[9] Liu, X.; Jiang, R.; Lan, Y.; Chen, W. P. *Org. Lett.* **2020**, 22, 8, 3149.

[10] Bankar, Siddheshwar K.; Ramasastry, S. S. V. *Angew. Chem.* **2018**, 130, 1694.

[11] Jin, J.H. *et al* . *Chinese Journal of Chemistry*, **2017**, 35(4), 397.

[12] Fu, C.L.; Ma, S.M. *Org. Lett.* **2015**, 7, 9, 1707.

[13]Yutaka. Mori.; Tsuyoshi. Chiba.; Akikazu. Matsumoto. *Crystal Growth & Design,* **2007**, 7, 1356.

[14]Dieter Scholz.; Sabine Weber-roth.; Elena Macoratti.; Eric Francotte. *Synthetic Communications.* **1999**, 29, 7, 1143.

[15]Y.N. Zhang, S.N. Shen, F. Hua, T. Xu, *Org. Lett.* **2020**, 22, 4, 1244.

X-Ray data for compounds

Crystals of **2l**, **2m**, **2s**, **2u**, **5g** and **5n** were obtained by recrystallization from MeOH.

The X-ray crystallography was measured on Bruker D8 Venture Photon instrument.

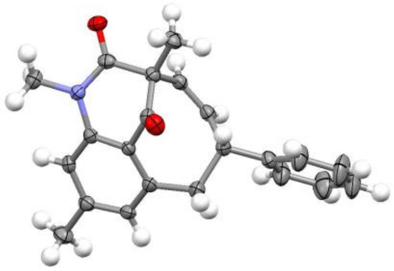

2l (The displacement ellipsoids are scaled to the 30% probability level.)

Supplementary table 1: Crystal data and structure refinement for 2l.

Identification code	190925b
Empirical formula	$C_{22}H_{21}NO_2$
Formula weight	331.40
Temperature/K	293(2)
Crystal system	orthorhombic
Space group	$P2_12_12_1$
a/Å	6.9756(6)
b/Å	8.9522(6)
c/Å	29.065(3)
α/°	90.00
β/°	90.00
γ/°	90.00
Volume/Å^3	1815.0(3)
Z	4
ρ_{calc}g/cm^3	1.213
μ/mm^{-1}	0.077
F(000)	704.0
Crystal size/mm^3	0.37 × 0.18 × 0.12
Radiation	MoKα (λ = 0.71073)
2Θ range for data collection/°	4.76 to 50.04
Index ranges	-8 ≤ h ≤ 8, -10 ≤ k ≤ 9, -34 ≤ l ≤ 32
Reflections collected	9281
Independent reflections	3209 [R_{int} = 0.0865, R_{sigma} = 0.0772]
Data/restraints/parameters	3209/0/229
Goodness-of-fit on F^2	1.032
Final R indexes [I>=2σ (I)]	R_1 = 0.0626, wR_2 = 0.1225
Final R indexes [all data]	R_1 = 0.0869, wR_2 = 0.1314

Largest diff. peak/hole / e Å^{-3}	0.17/-0.12
Flack parameter	1(2)

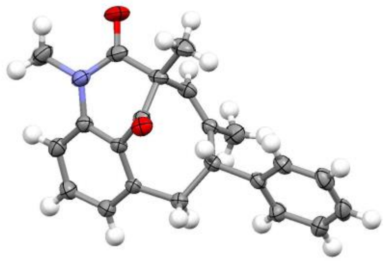

2m (The displacement ellipsoids are scaled to the 30% probability level.)

Supplementary table 2: Crystal data and structure refinement for 2m.

Identification code	190719b
Empirical formula	$C_{22}H_{21}NO_2$
Formula weight	331.40
Temperature/K	298(2)
Crystal system	N/A
Space group	Pbca
a/Å	19.7961(17)
b/Å	8.2070(8)
c/Å	21.6659(19)
α/°	90.00
β/°	90.00
γ/°	90.00
Volume/Å^3	3520.0(6)
Z	8
ρ_{calc}g/cm^3	1.251
μ/mm^{-1}	0.080
F(000)	1408.0
Crystal size/mm^3	0.40 × 0.16 × 0.10
Radiation	MoKα (λ = 0.71073)
2Θ range for data collection/°	5.58 to 50.04
Index ranges	-21 ≤ h ≤ 23, -9 ≤ k ≤ 9, -21 ≤ l ≤ 25
Reflections collected	16097
Independent reflections	3102 [R_{int} = 0.0909, R_{sigma} = N/A]
Data/restraints/parameters	3102/0/229
Goodness-of-fit on F^2	1.034
Final R indexes [I>=2σ (I)]	R_1 = 0.0499, wR_2 = 0.0973
Final R indexes [all data]	R_1 = 0.1064, wR_2 = 0.1076
Largest diff. peak/hole / e Å^{-3}	0.14/-0.14

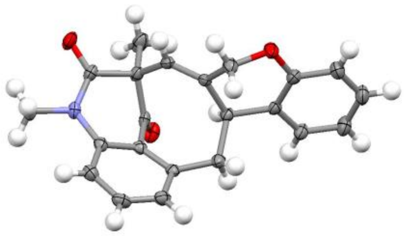

2s (The displacement ellipsoids are scaled to the 30% probability level.)

Supplementary table 3: Crystal data and structure refinement for 2s.

Identification code	200829c
Empirical formula	$C_{22}H_{19}NO_3$
Formula weight	345.38
Temperature/K	298(2)
Crystal system	triclinic
Space group	P-1
a/Å	8.5891(6)
b/Å	10.0496(8)
c/Å	10.8868(9)
α/°	99.979(2)
β/°	94.1160(10)
γ/°	113.287(5)
Volume/Å^3	839.82(11)
Z	2
ρ_{calc}g/cm^3	1.366
μ/mm^{-1}	0.091
F(000)	364.0
Crystal size/mm^3	0.3 × 0.2 × 0.11
Radiation	MoKα (λ = 0.71073)
2Θ range for data collection/°	4.52 to 50.04
Index ranges	-10 ≤ h ≤ 6, -10 ≤ k ≤ 11, -12 ≤ l ≤ 12
Reflections collected	4221
Independent reflections	2887 [R_{int} = 0.0249, R_{sigma} = 0.0638]
Data/restraints/parameters	2887/0/237
Goodness-of-fit on F^2	1.059
Final R indexes [I>=2σ (I)]	R_1 = 0.0588, wR_2 = 0.1346
Final R indexes [all data]	R_1 = 0.0945, wR_2 = 0.1476
Largest diff. peak/hole / e Å^{-3}	0.18/-0.27

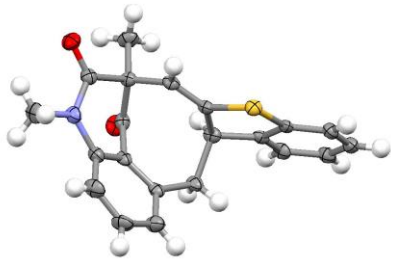

2u (The displacement ellipsoids are scaled to the 30% probability level.)

Supplementary table 4: Crystal data and structure refinement for 2u.

Identification code	190720g
Empirical formula	$C_{21}H_{17}NO_2S$
Formula weight	347.42
Temperature/K	298(2)
Crystal system	N/A
Space group	$P2_1$
a/Å	8.6332(8)
b/Å	8.5534(9)
c/Å	12.2603(11)
α/°	90.00
β/°	107.009(3)
γ/°	90.00
Volume/Å^3	865.74(14)
Z	2
ρ_{calc}g/cm^3	1.333
μ/mm^{-1}	0.201
F(000)	364.0
Crystal size/mm^3	0.32 × 0.12 × 0.10
Radiation	MoKα (λ = 0.71073)
2Θ range for data collection/°	4.94 to 50.04
Index ranges	-6 ≤ h ≤ 10, -10 ≤ k ≤ 9, -14 ≤ l ≤ 12
Reflections collected	4166
Independent reflections	2915 [R_{int} = 0.0366, R_{sigma} = N/A]
Data/restraints/parameters	2915/1/228
Goodness-of-fit on F^2	1.062
Final R indexes [I>=2σ (I)]	R_1 = 0.0550, wR_2 = 0.1131
Final R indexes [all data]	R_1 = 0.0753, wR_2 = 0.1226
Largest diff. peak/hole / e Å^{-3}	0.35/-0.21
Flack parameter	0.17(12)

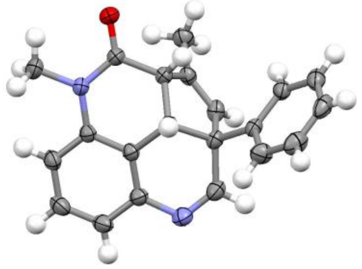

5g (The displacement ellipsoids are scaled to the 30% probability level.)

Supplementary table 5: Crystal data and structure refinement for 5g.

Identification code	200605b
Empirical formula	$C_{21}H_{18}N_2O$
Formula weight	314.37
Temperature/K	298(2)
Crystal system	orthorhombic
Space group	$P2_12_12_1$
a/Å	8.8268(8)
b/Å	12.9340(11)
c/Å	13.5268(11)
α/°	90.00
β/°	90.00
γ/°	90.00
Volume/Å^3	1544.3(2)
Z	4
ρ_{calc}g/cm^3	1.352
μ/mm^{-1}	0.084
F(000)	664.0
Crystal size/mm^3	0.4 × 0.16 × 0.14
Radiation	MoKα (λ = 0.71073)
2Θ range for data collection/°	4.36 to 50.04
Index ranges	-10 ≤ h ≤ 10, -15 ≤ k ≤ 14, -12 ≤ l ≤ 16
Reflections collected	7572
Independent reflections	2711 [R_{int} = 0.0778, R_{sigma} = 0.0650]
Data/restraints/parameters	2711/0/220
Goodness-of-fit on F^2	1.013
Final R indexes [I>=2σ (I)]	R_1 = 0.0521, wR_2 = 0.1307
Final R indexes [all data]	R_1 = 0.0714, wR_2 = 0.1419
Largest diff. peak/hole / e Å^{-3}	0.16/-0.17
Flack parameter	4(3)

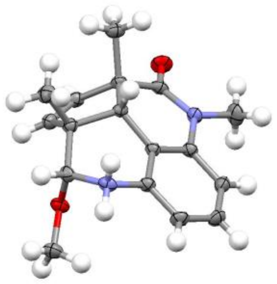

5n Methanol adduct (The displacement ellipsoids are scaled to the 30% probability level.)

Supplementary table 6: Crystal data and structure refinement for 5n Methanol adduct.

Identification code	200628d
Empirical formula	$C_{17}H_{21}N_2O_2$
Formula weight	285.36
Temperature/K	298(2)
Crystal system	triclinic
Space group	P-1
a/Å	9.113(15)
b/Å	9.349(15)
c/Å	10.247(17)
α/°	78.64(2)
β/°	72.48(2)
γ/°	62.402(19)
Volume/Å^3	736(2)
Z	2
ρ_{calc}g/cm^3	1.287
μ/mm^{-1}	0.085
F(000)	306.0
Crystal size/mm^3	17 × 0.33 × 0.21
Radiation	MoKα (λ = 0.71073)
2Θ range for data collection/°	4.92 to 50.04
Index ranges	-10 ≤ h ≤ 10, -11 ≤ k ≤ 11, -10 ≤ l ≤ 12
Reflections collected	3714
Independent reflections	2555 [R_{int} = 0.0496, R_{sigma} = 0.0455]
Data/restraints/parameters	2555/0/194
Goodness-of-fit on F^2	1.029
Final R indexes [I>=2σ (I)]	R_1 = 0.0638, wR_2 = 0.1713
Final R indexes [all data]	R_1 = 0.0801, wR_2 = 0.1844
Largest diff. peak/hole / e Å^{-3}	0.31/-0.66

NMR Data

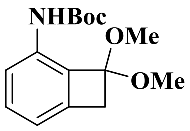

S2

^{1}H NMR of compound **S2**

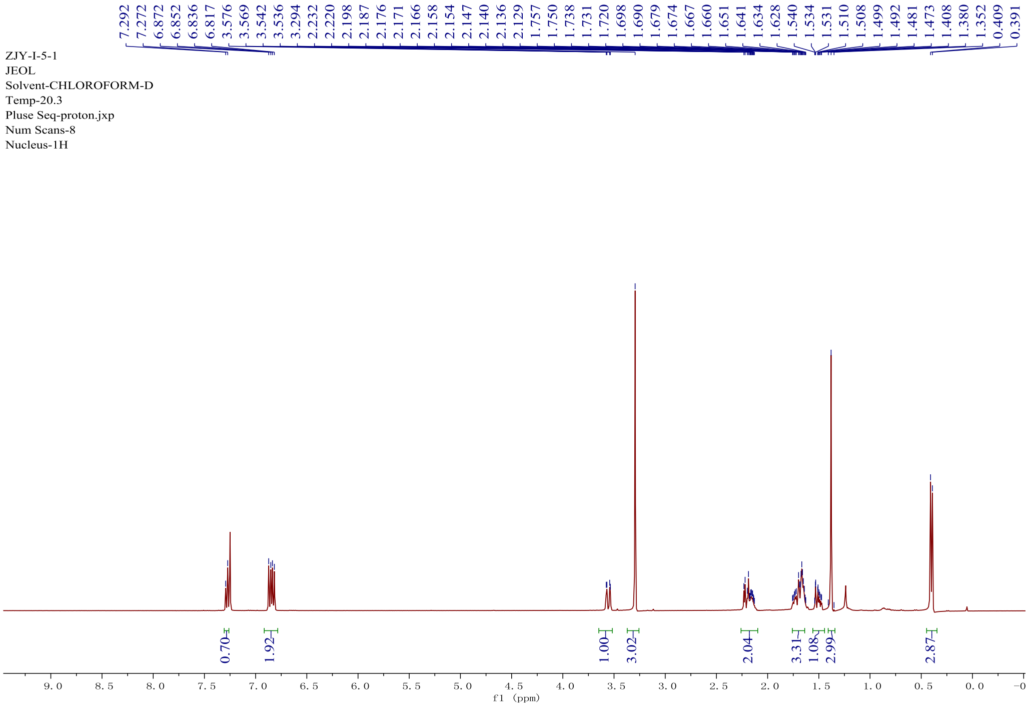

^{13}C NMR of compound **S2**

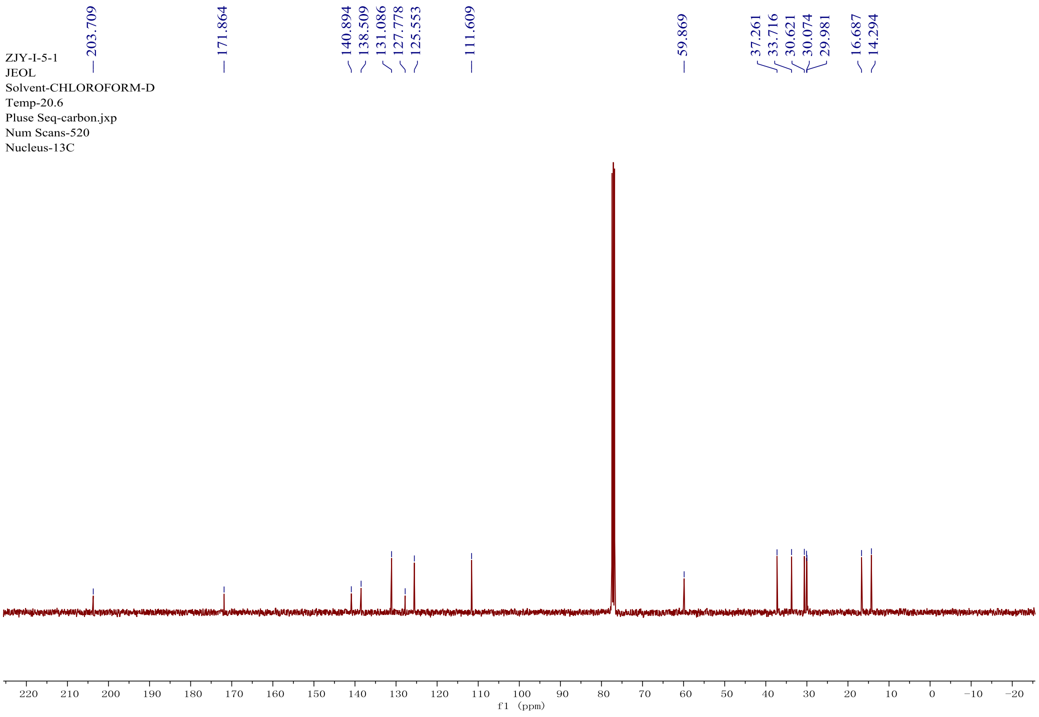

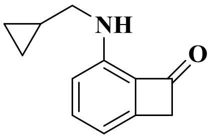

S5

^{1}H NMR of compound **S5**

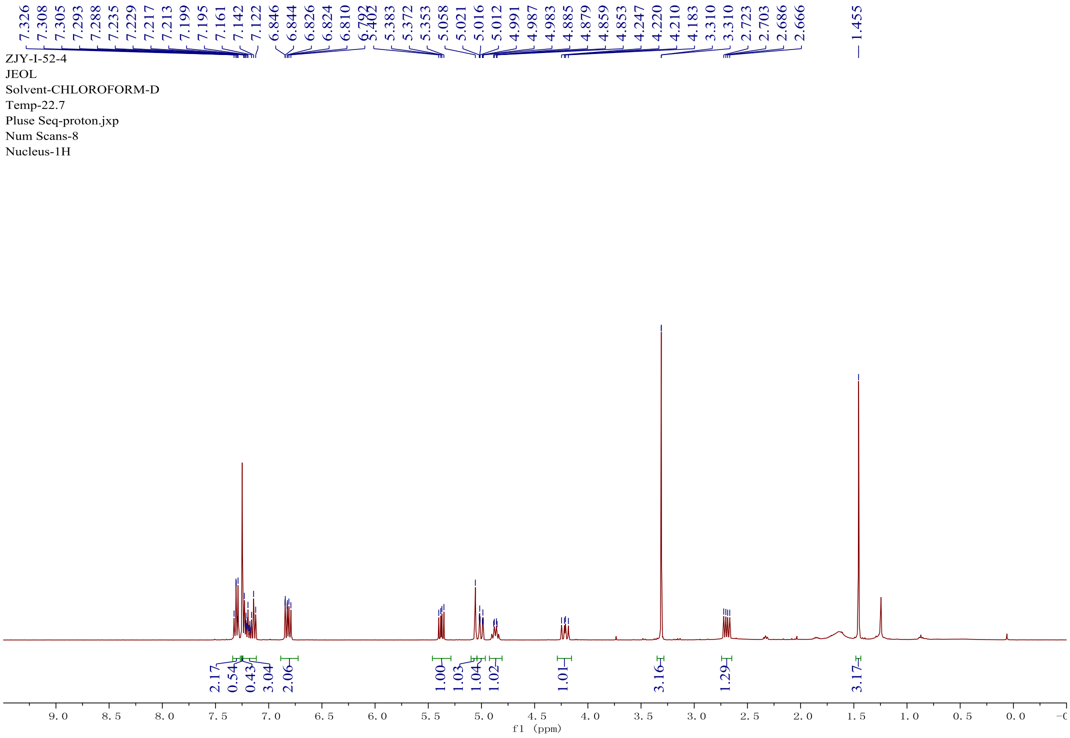

^{13}C NMR of compound **S5**

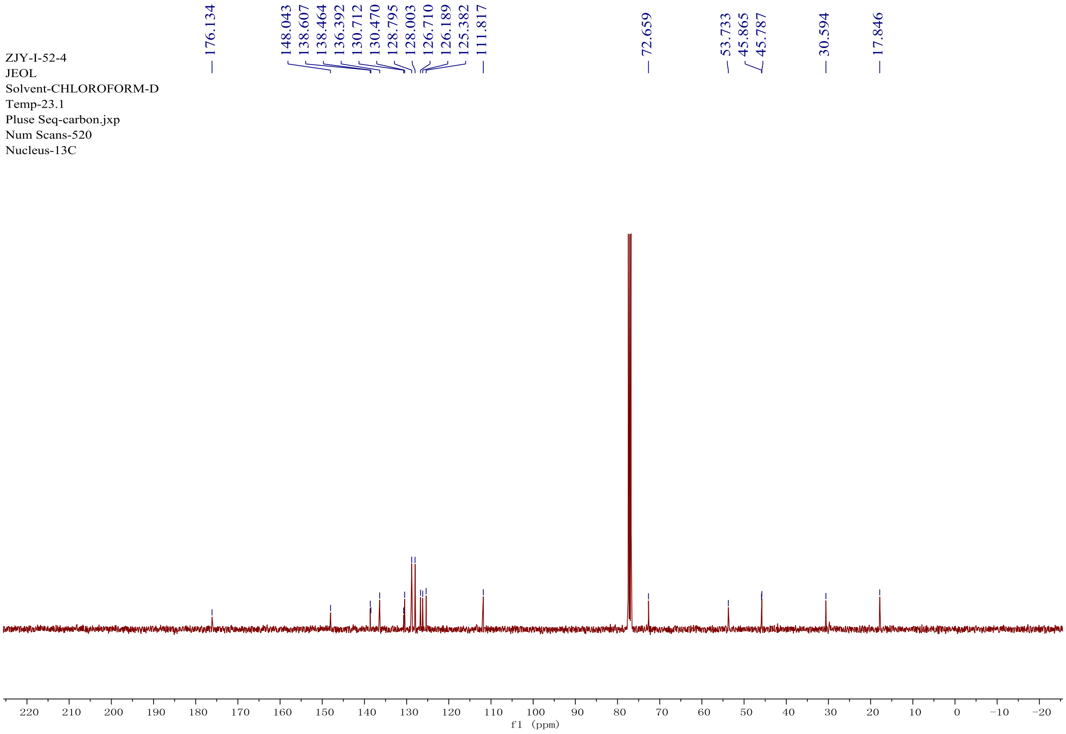

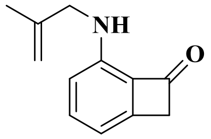

S6

^{1}H NMR of compound **S6**

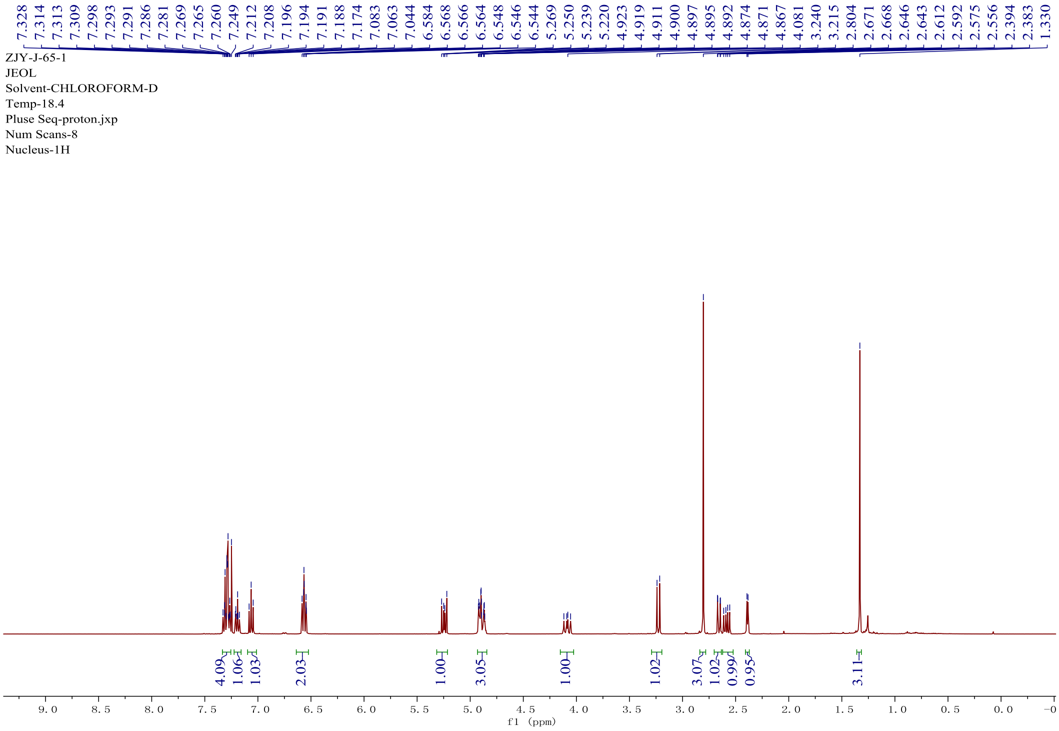

^{13}C NMR of compound **S6**

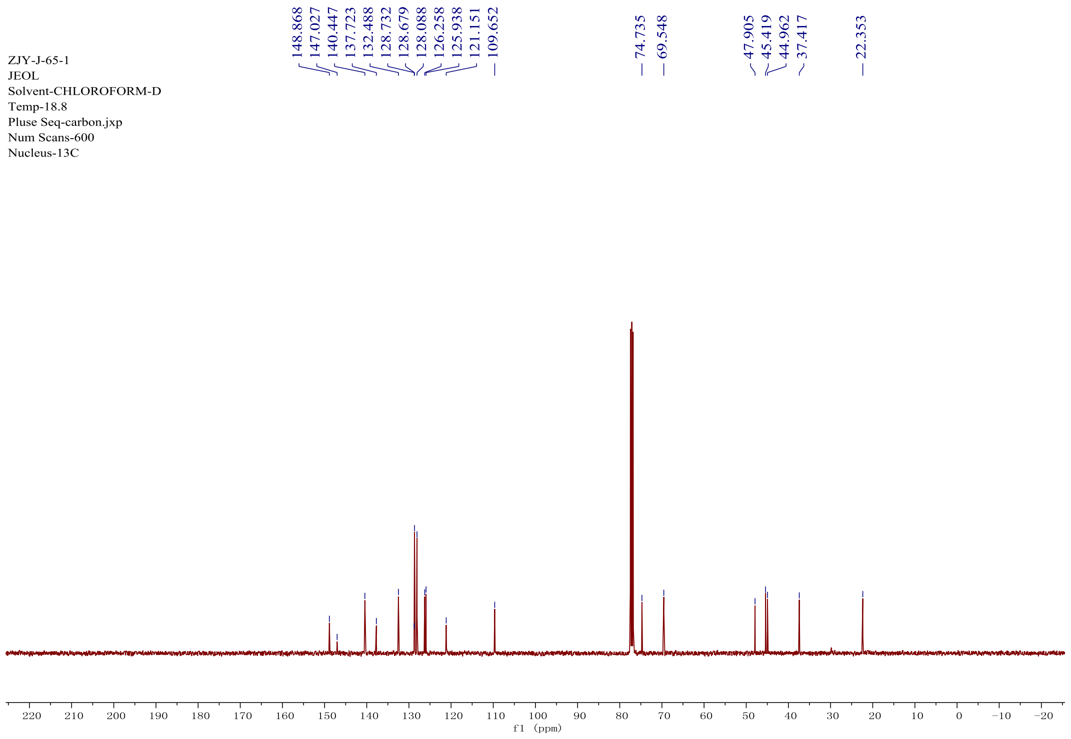

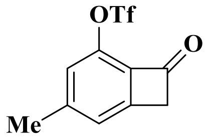

S7

^{1}H NMR of compound **S7**

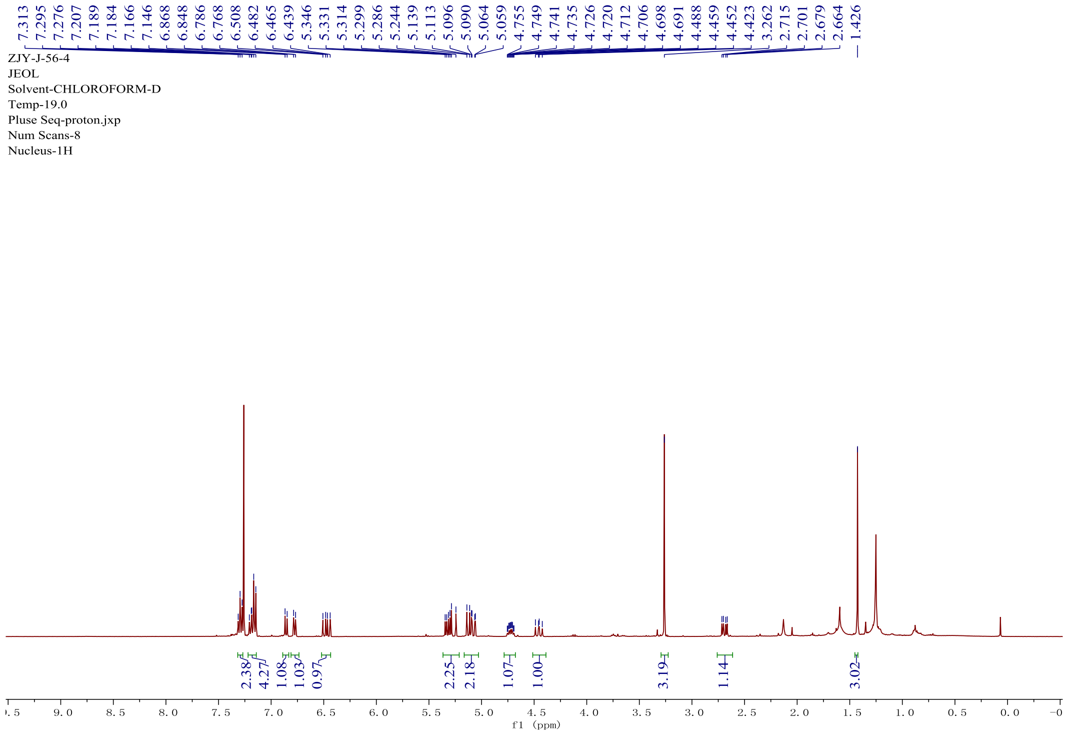

^{13}C NMR of compound **S7**

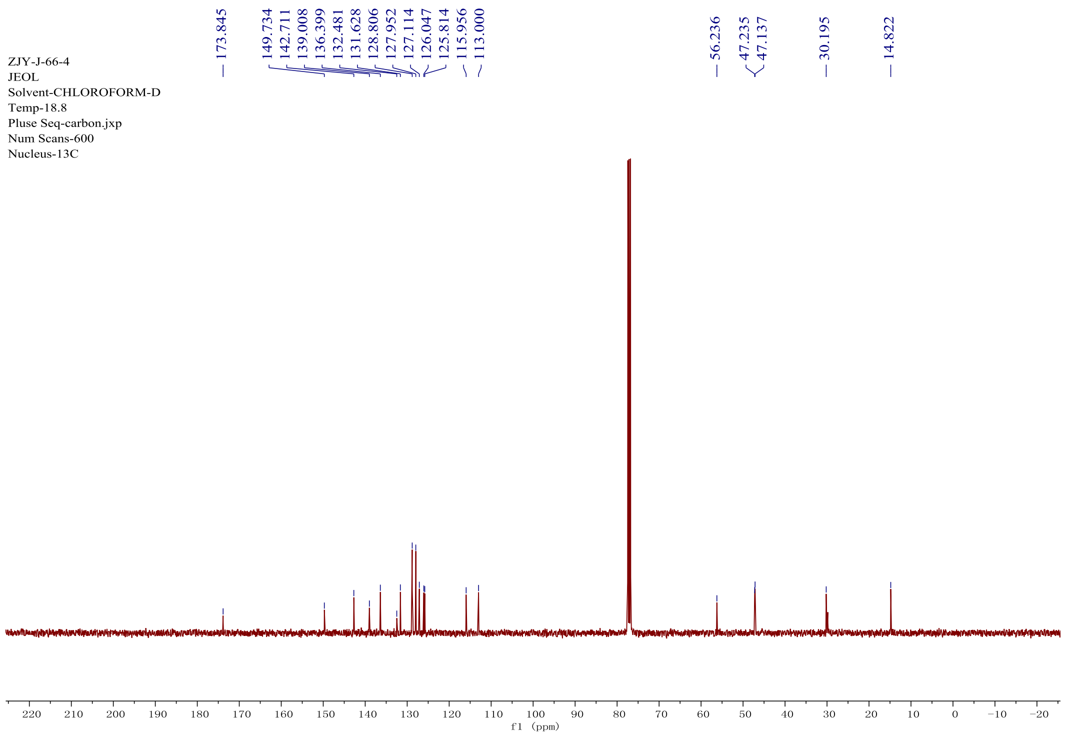

^{19}F NMR of compound **S7**

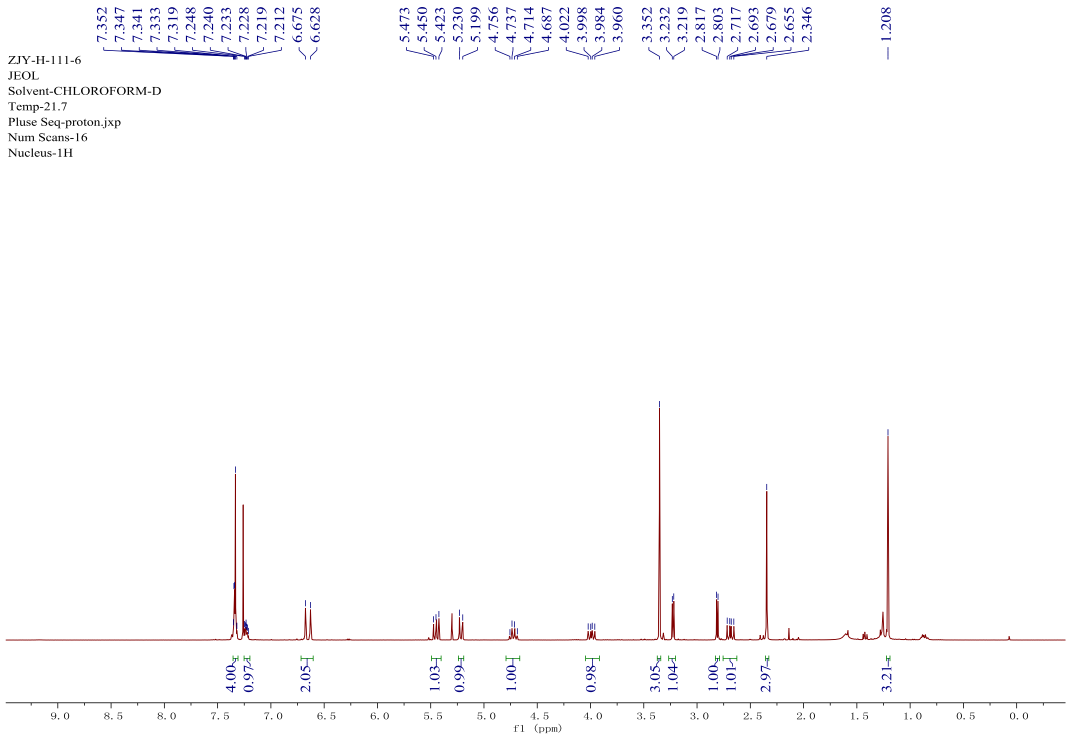

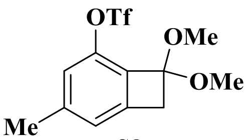

S8

^{1}H NMR of compound **S8**

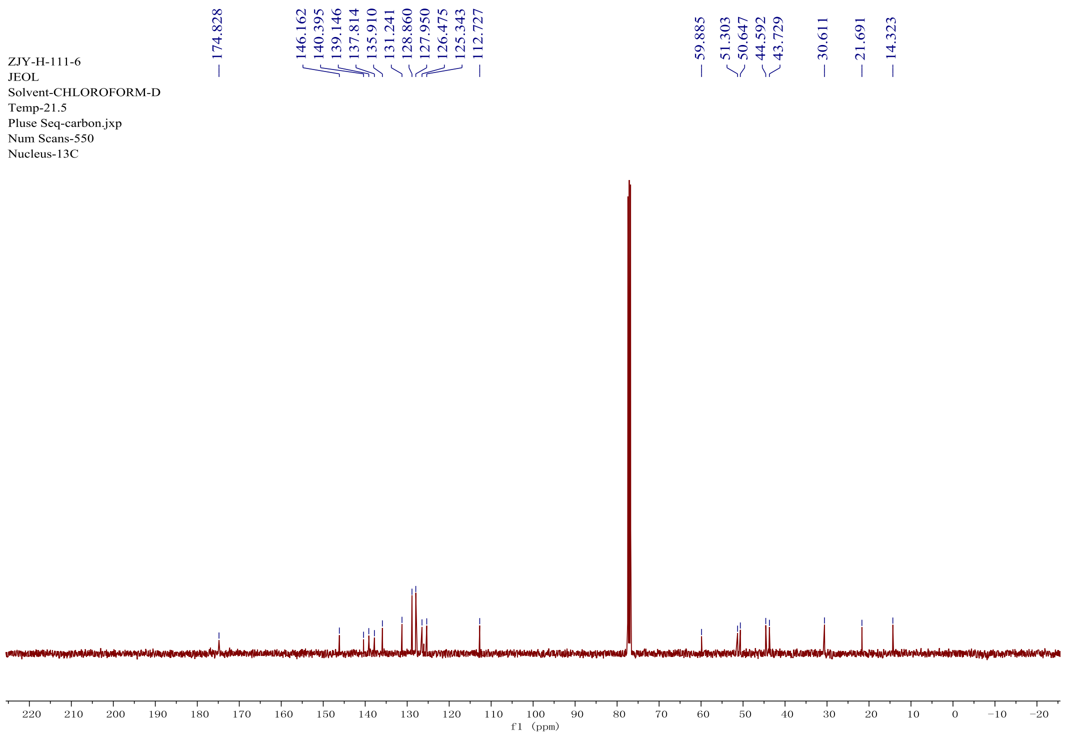

^{13}C NMR of compound **S8**

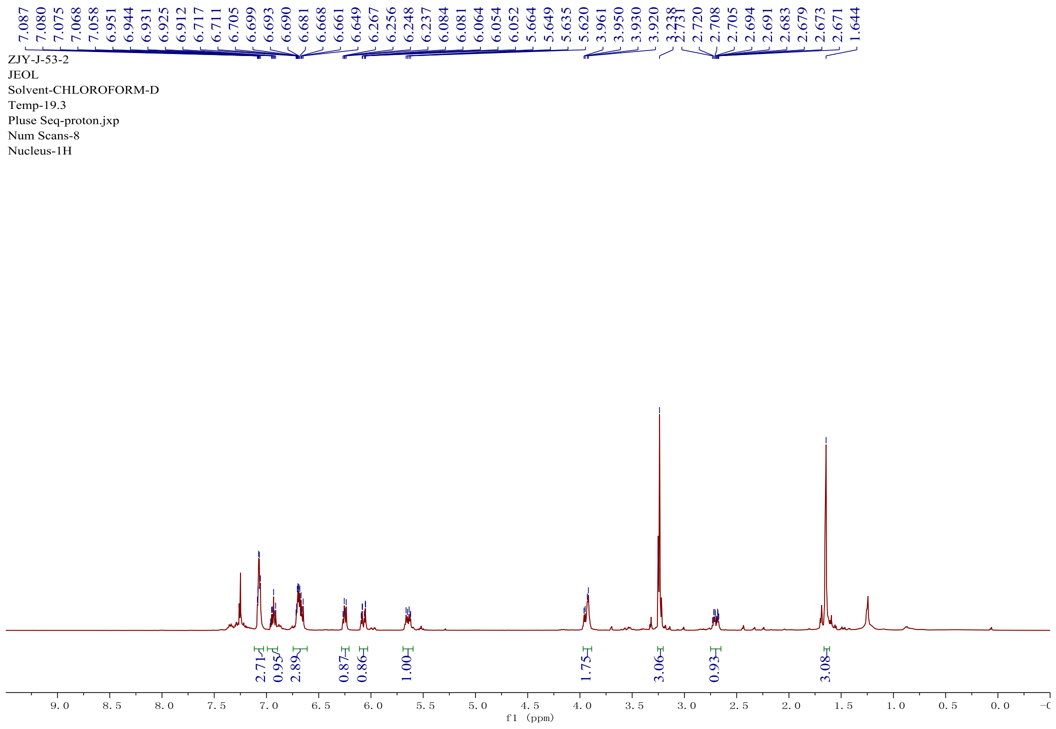

^{19}F NMR of compound **S8**

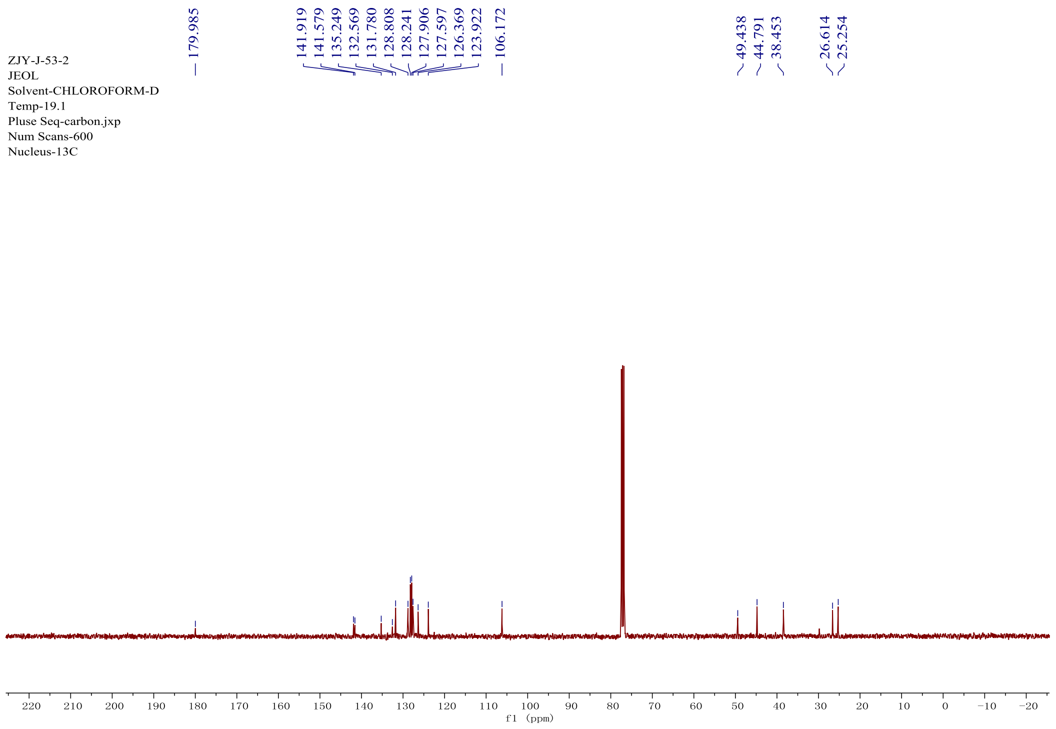

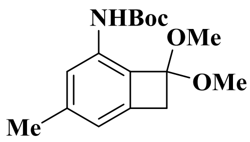

S9

^{1}H NMR of compound **S9**

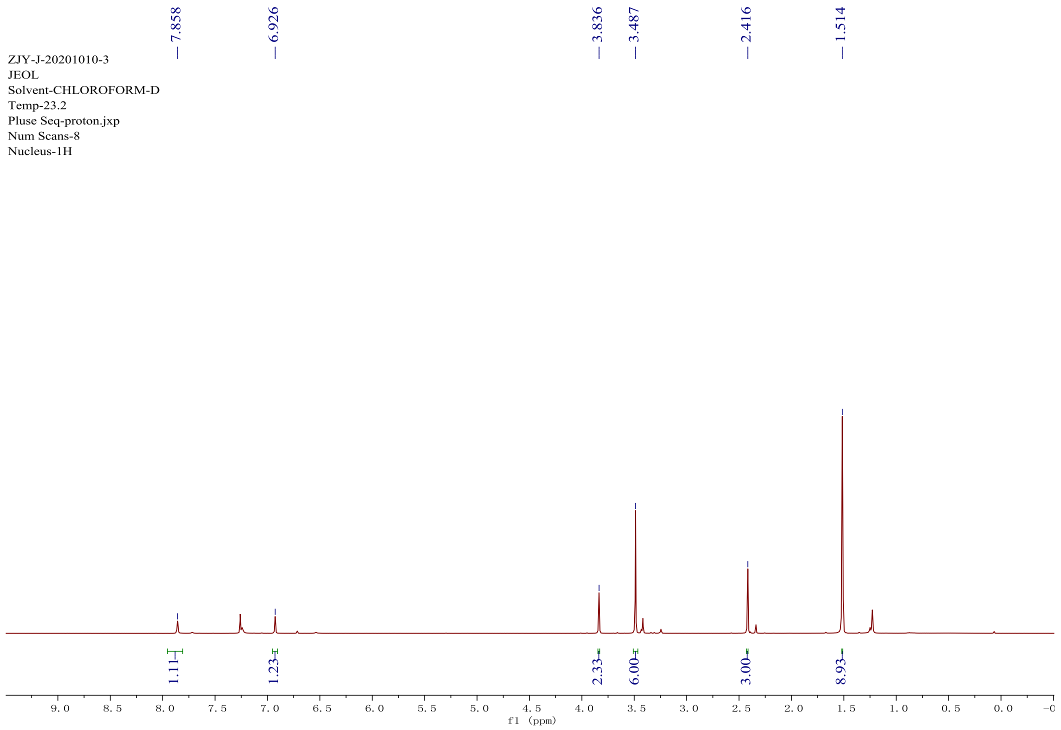

^{13}C NMR of compound **S9**

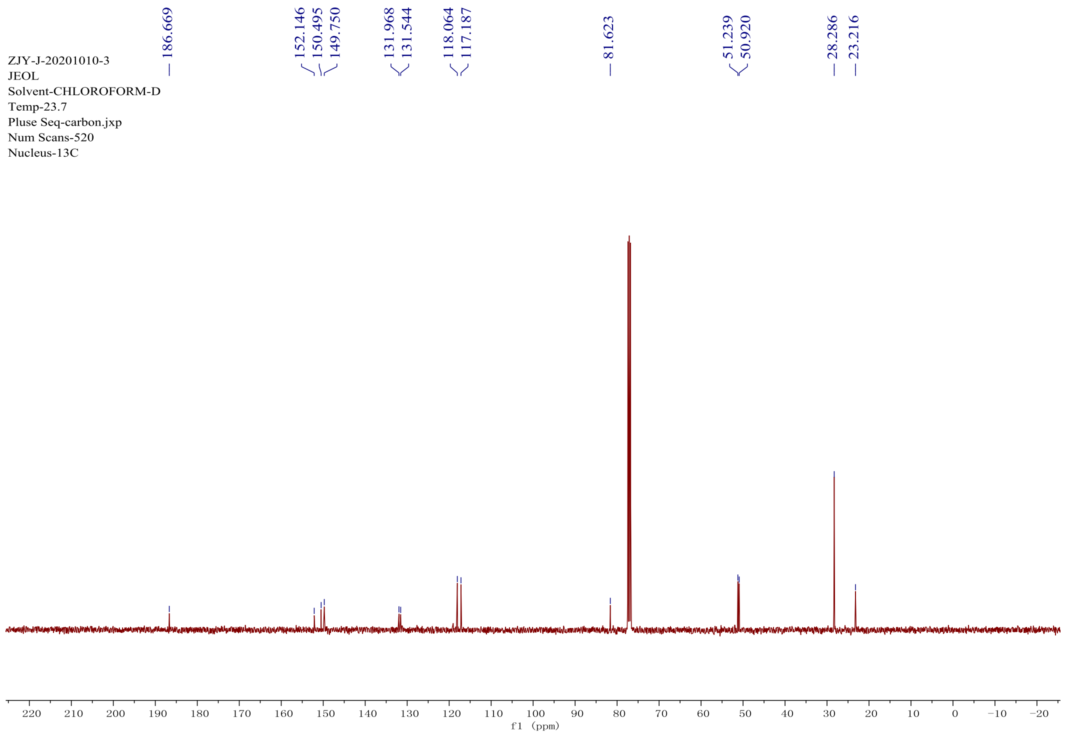

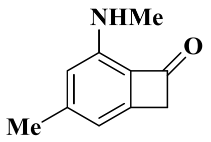

S10

^{1}H NMR of compound **S10**

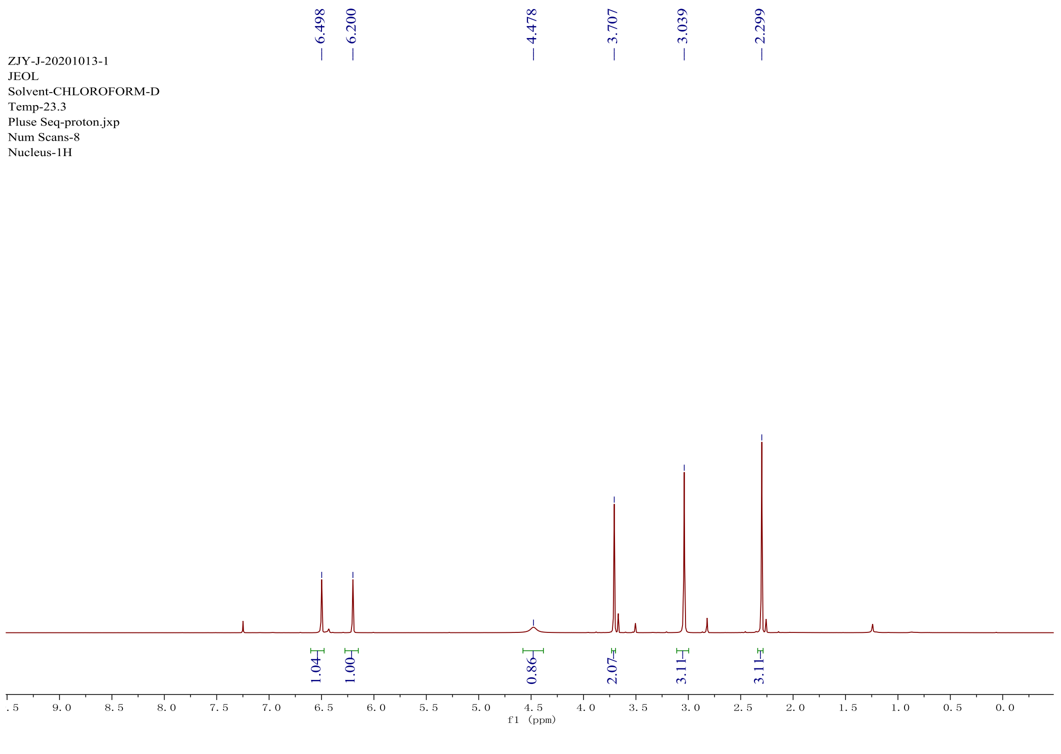

^{13}C NMR of compound **S10**

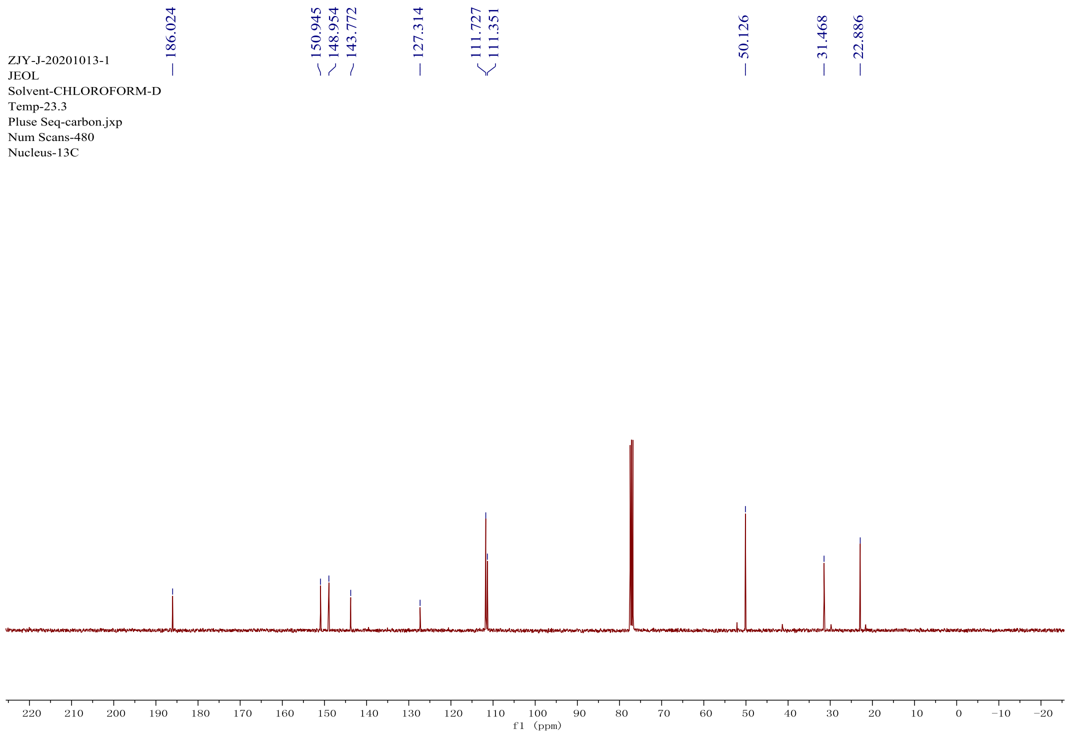

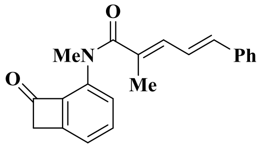

1a

^{1}H NMR of compound **1a**

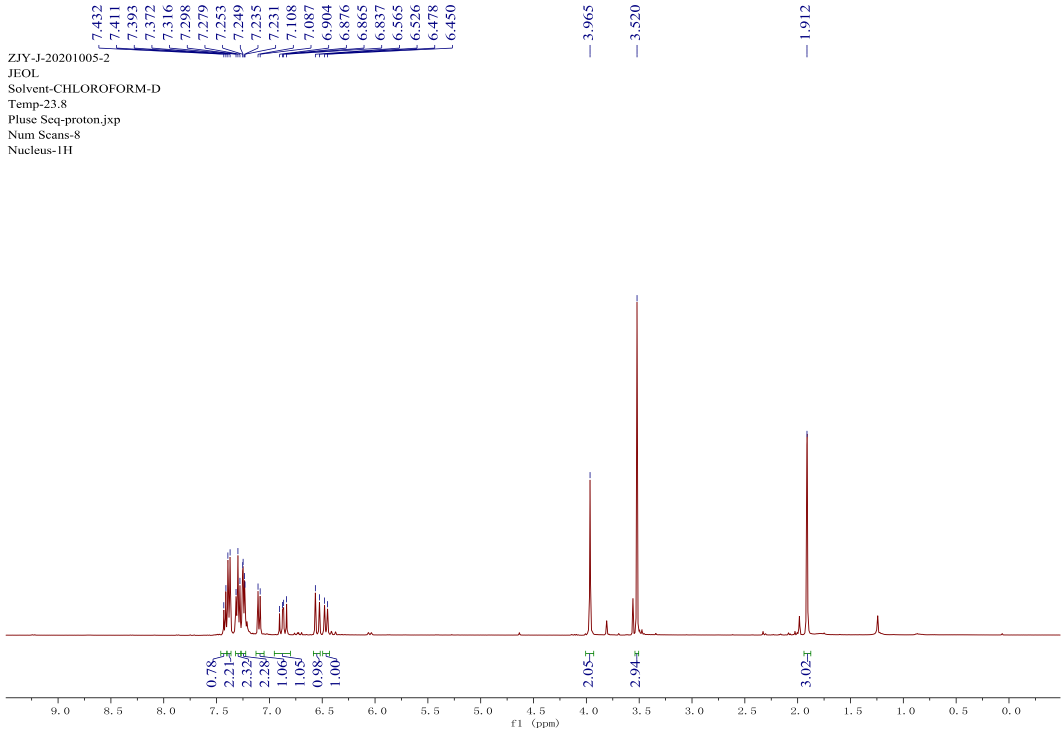

^{13}C NMR of compound **1a**

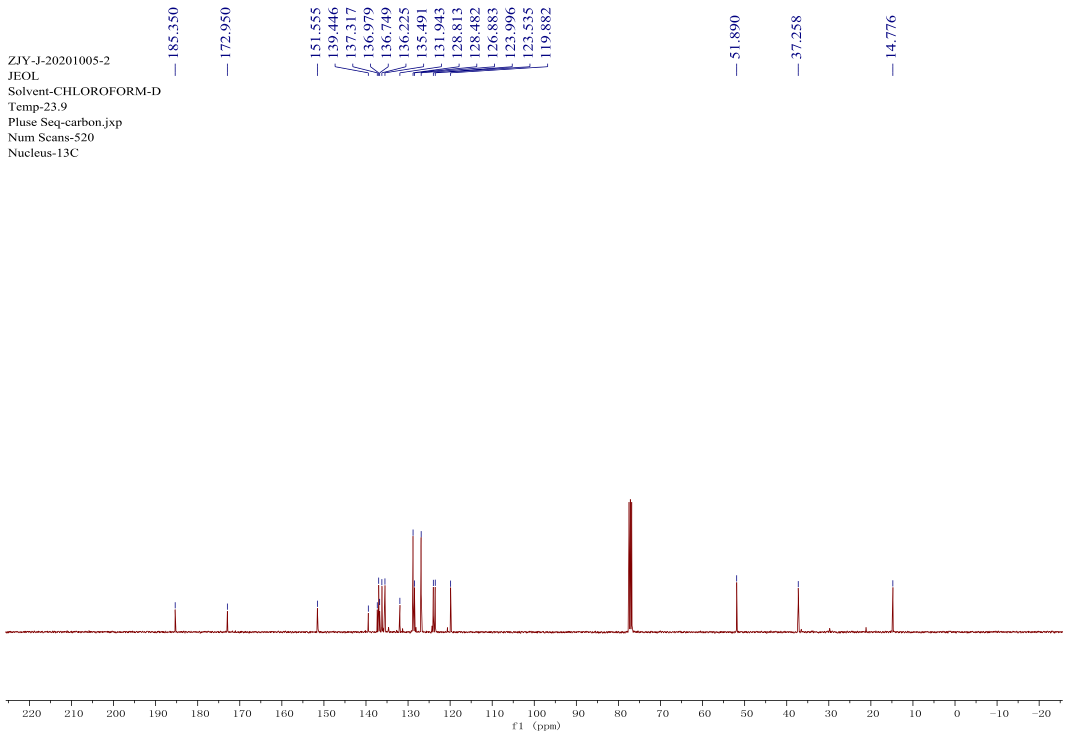

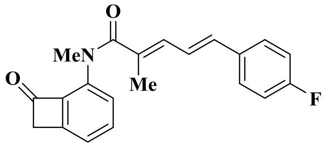

1b

^{1}H NMR of compound **1b**

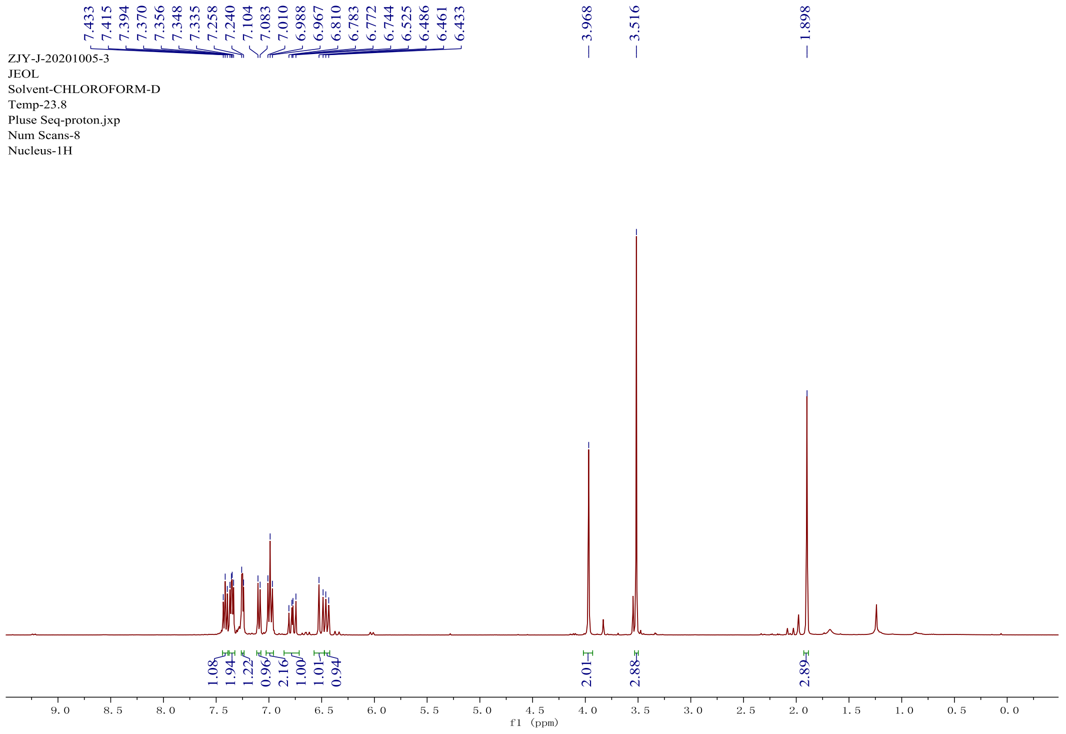

^{13}C NMR of compound **1b**

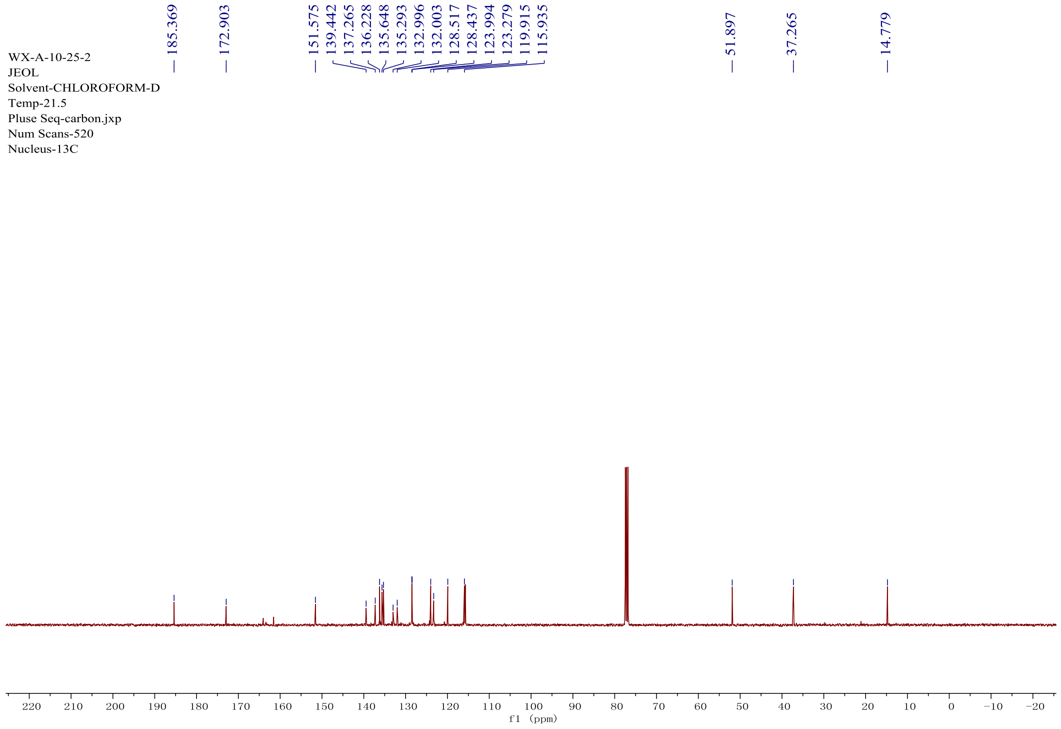

^{19}F NMR of compound **1b**

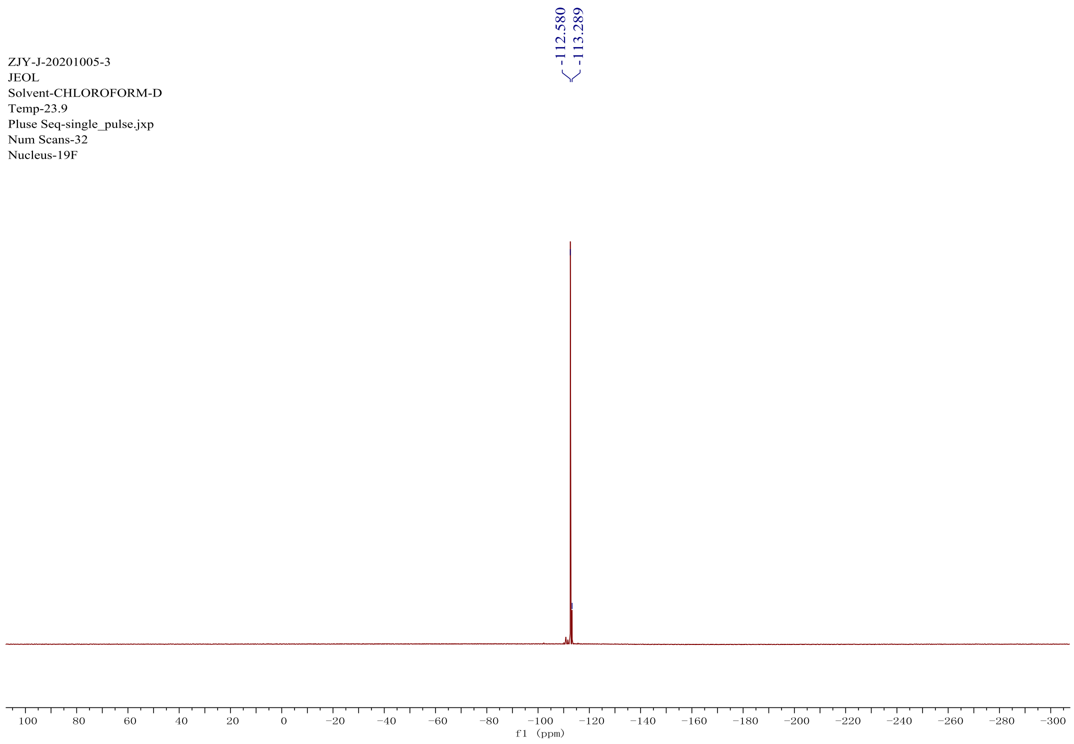

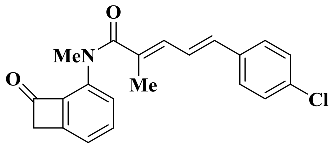

1c

^{1}H NMR of compound **1c**

ZJY-H-47-1-1
JEOL
Solvent-CHLOROFORM-D
Temp-22.8
Pluse Seq-proton.jxp
Num Scans-32
Nucleus-1H

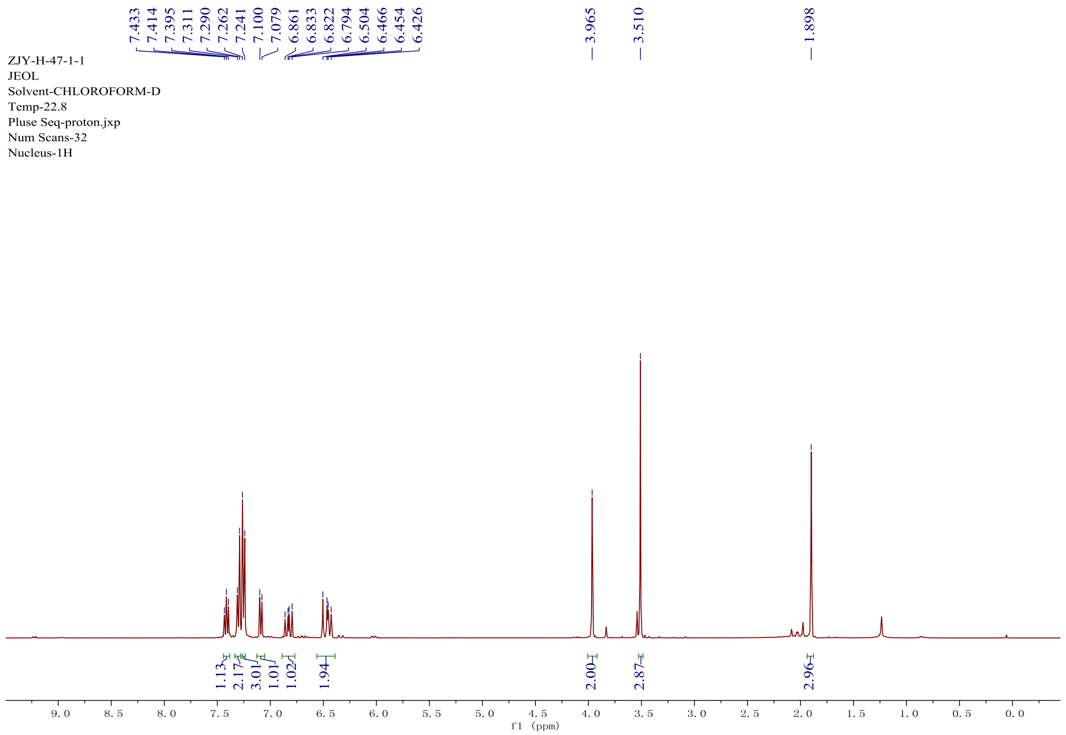

^{13}C NMR of compound **1c**

ZJY-H-47-1
JEOL
Solvent-CHLOROFORM-D
Temp-22.9
Pluse Seq-carbon.jxp
Num Scans-500
Nucleus-13C

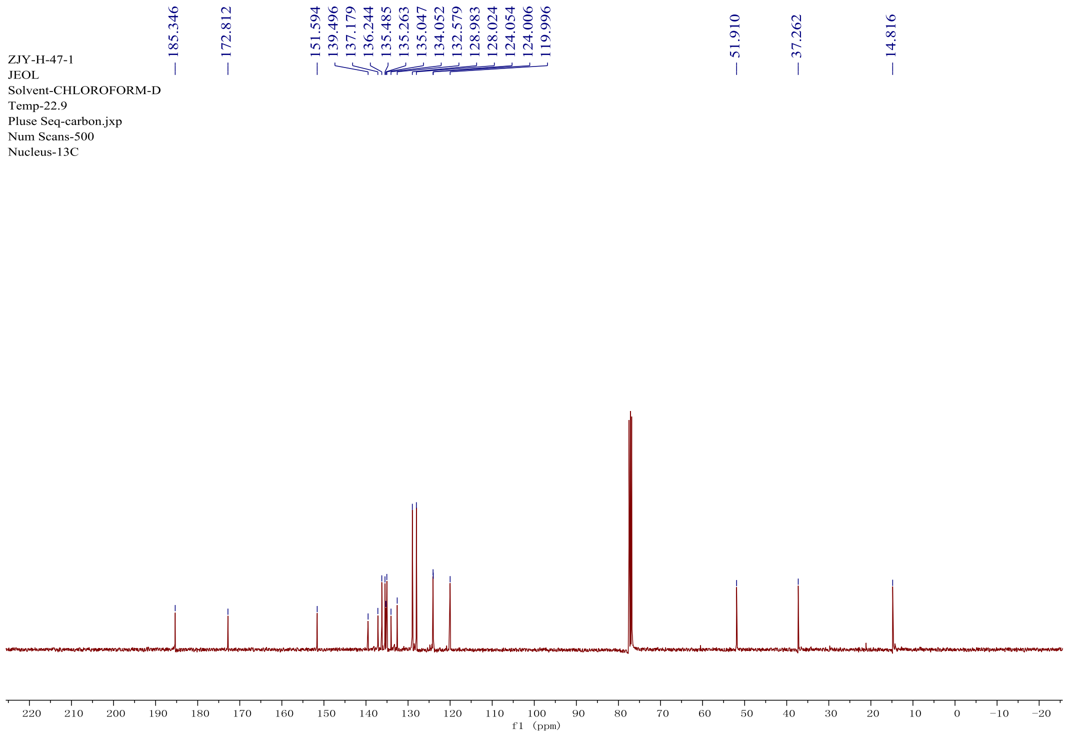

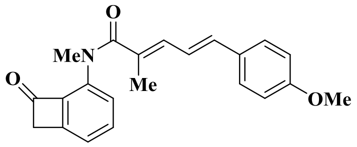

1d

^{1}H NMR of compound **1d**

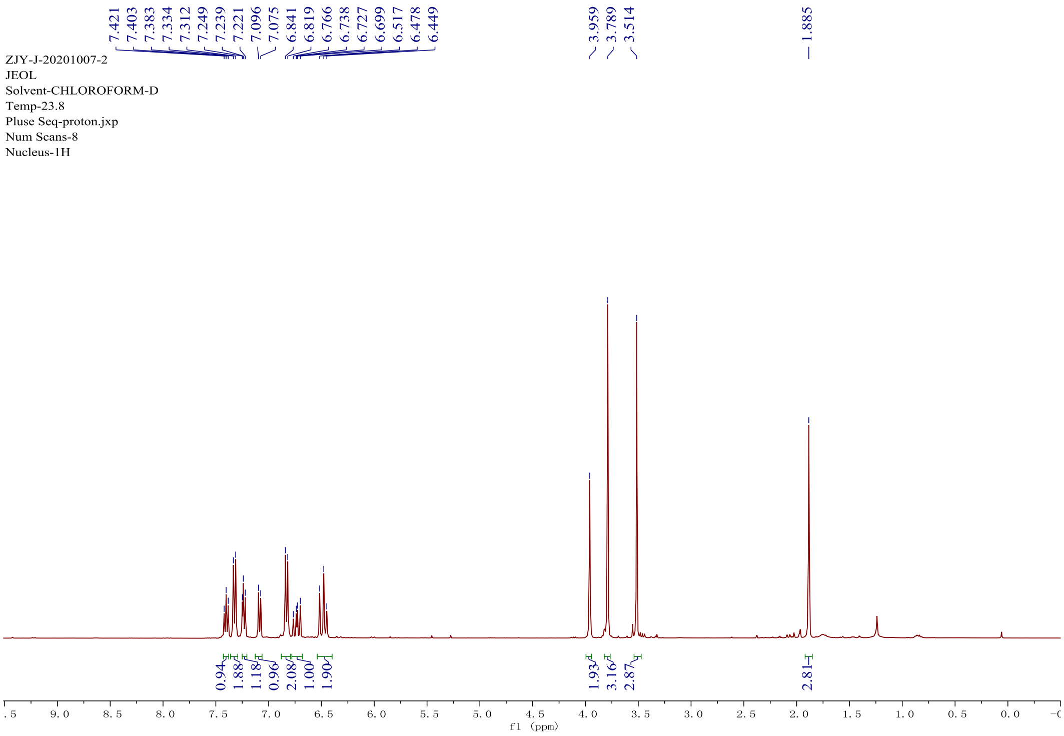

^{13}C NMR of compound **1d**

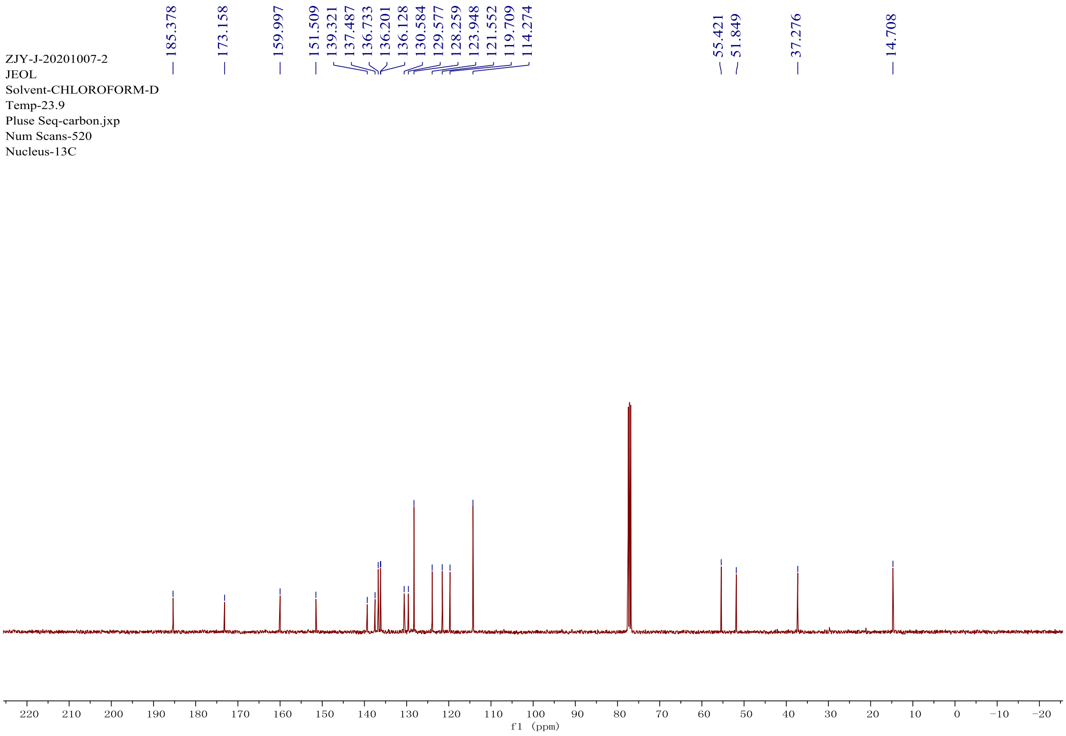

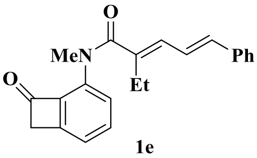

1e

^{1}H NMR of compound**1e**

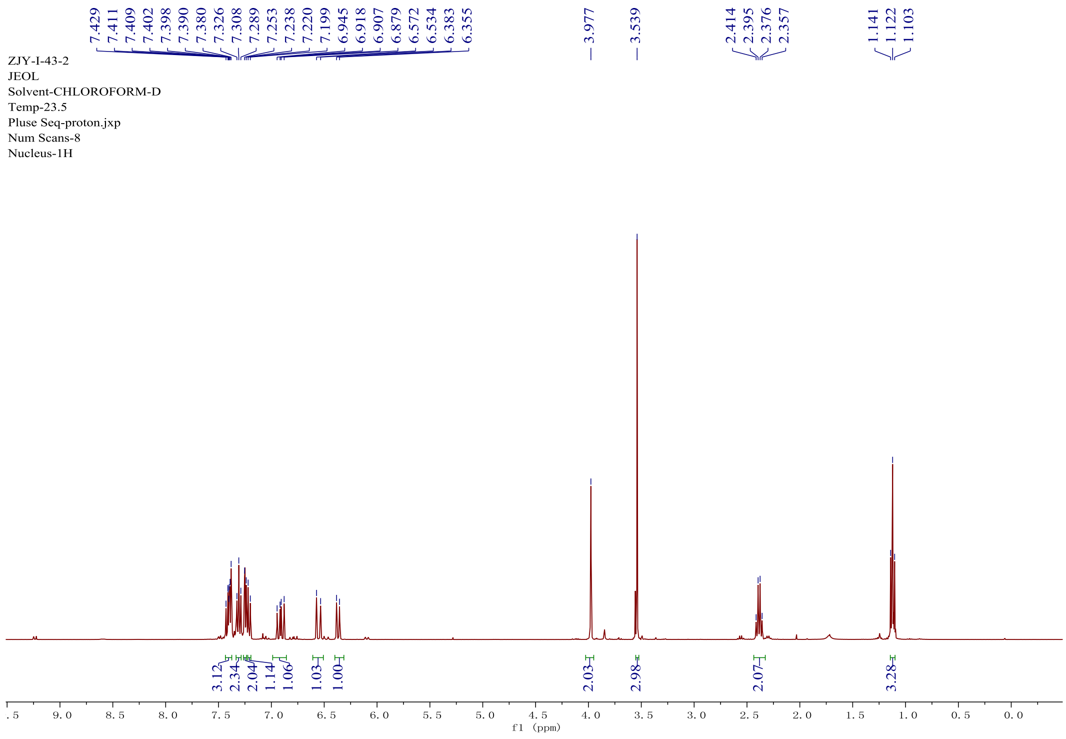

^{13}C NMR of compound **1e**

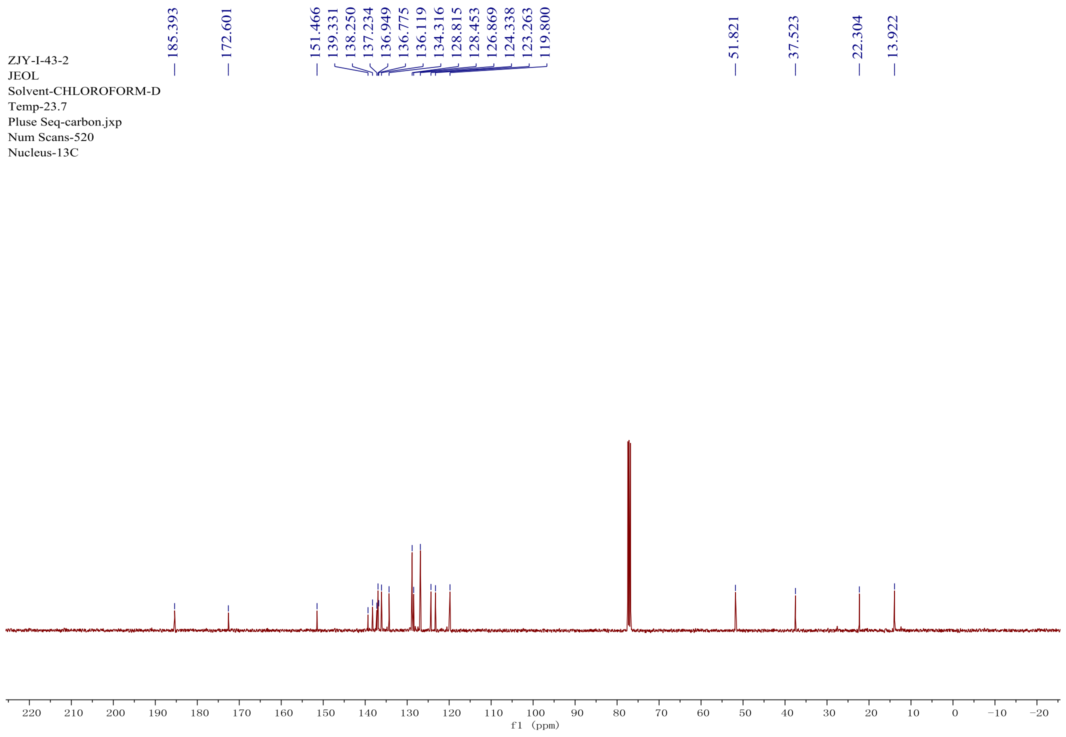

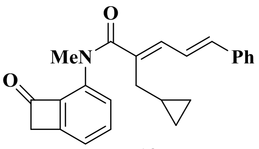

1f

^{1}H NMR of compound **1f**

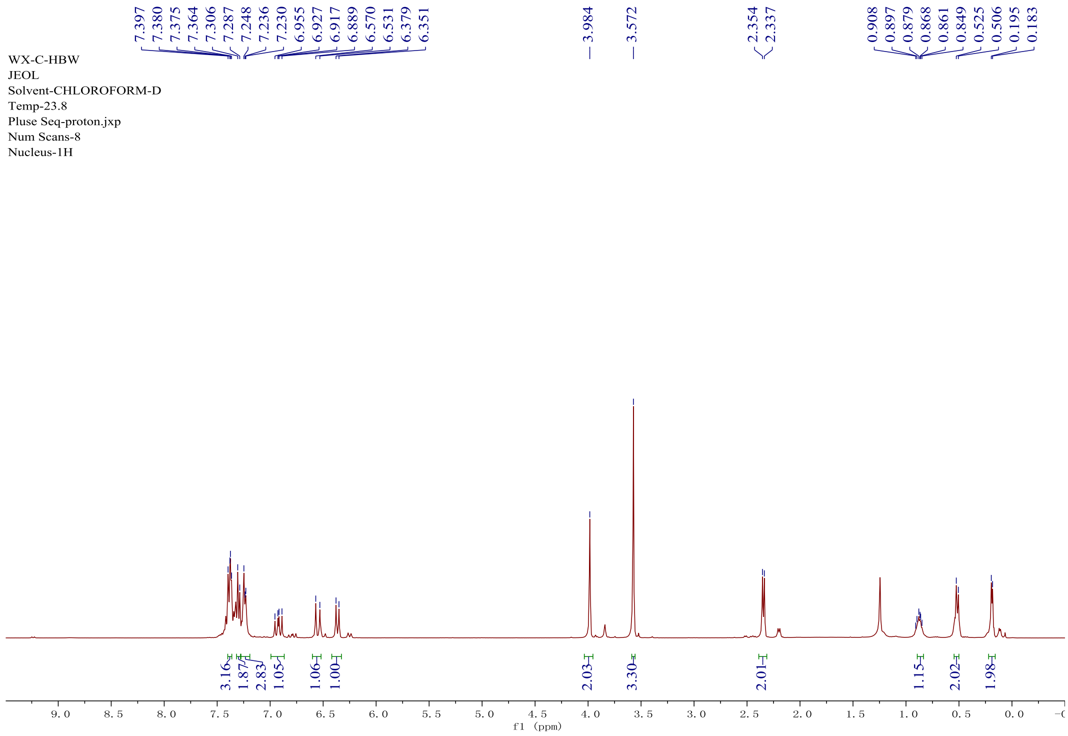

^{13}C NMR of compound **1f**

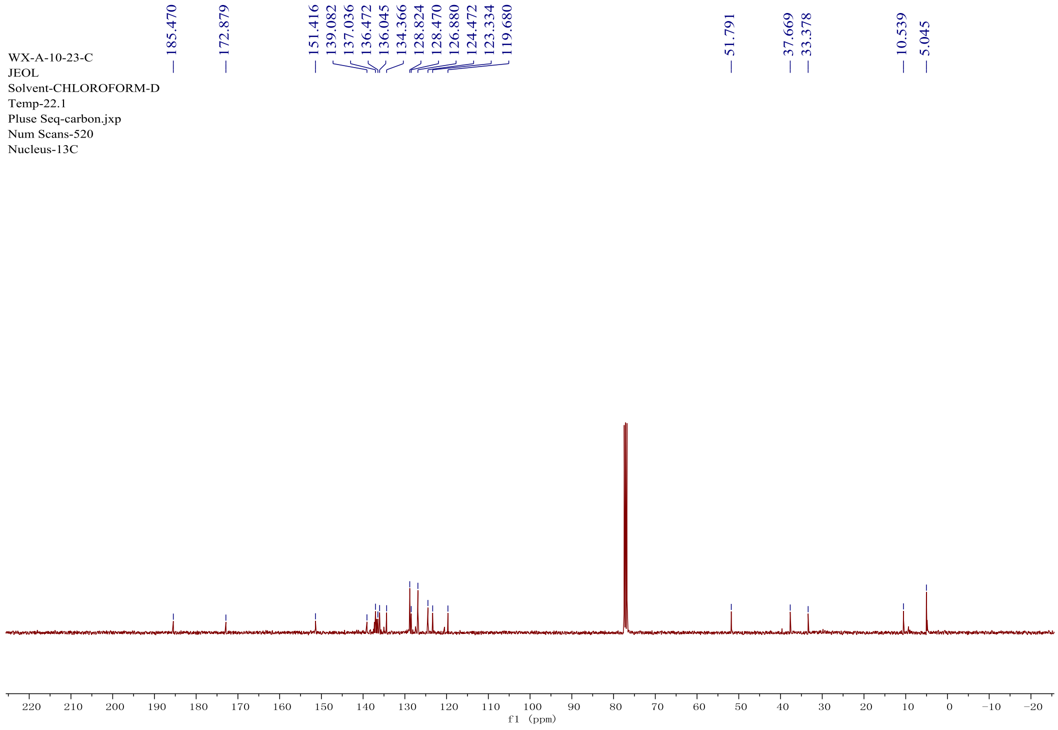

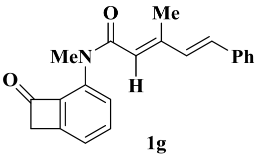

1g

^{1}H NMR of compound **1g**

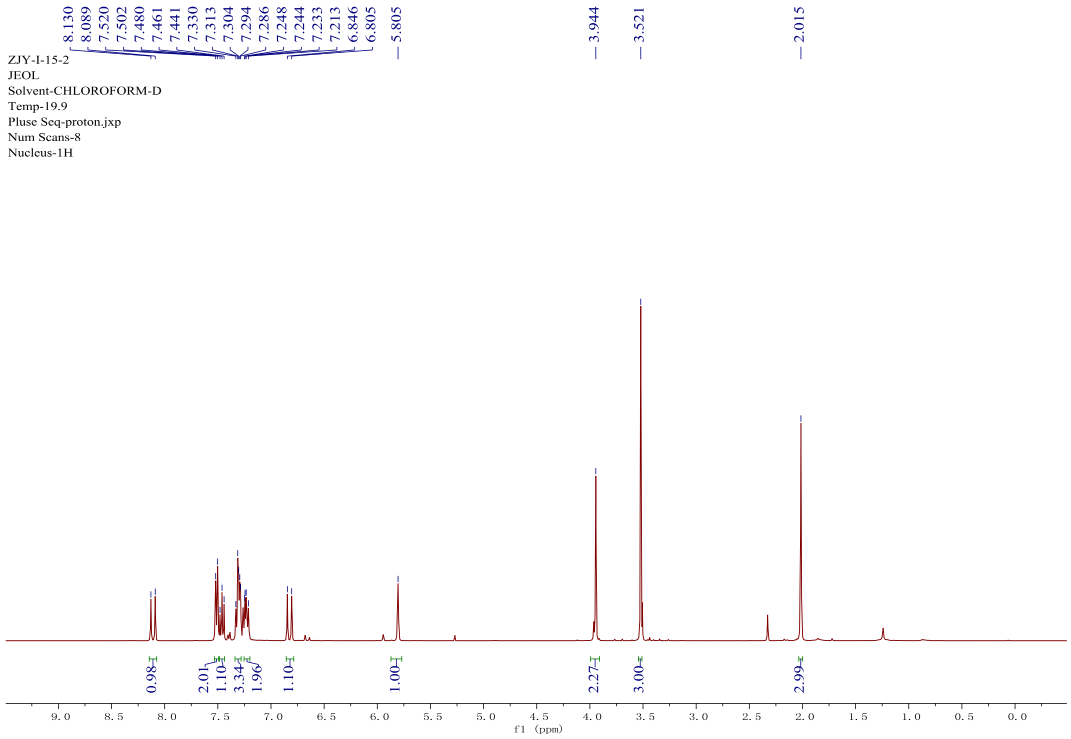

^{13}C NMR of compound **1g**

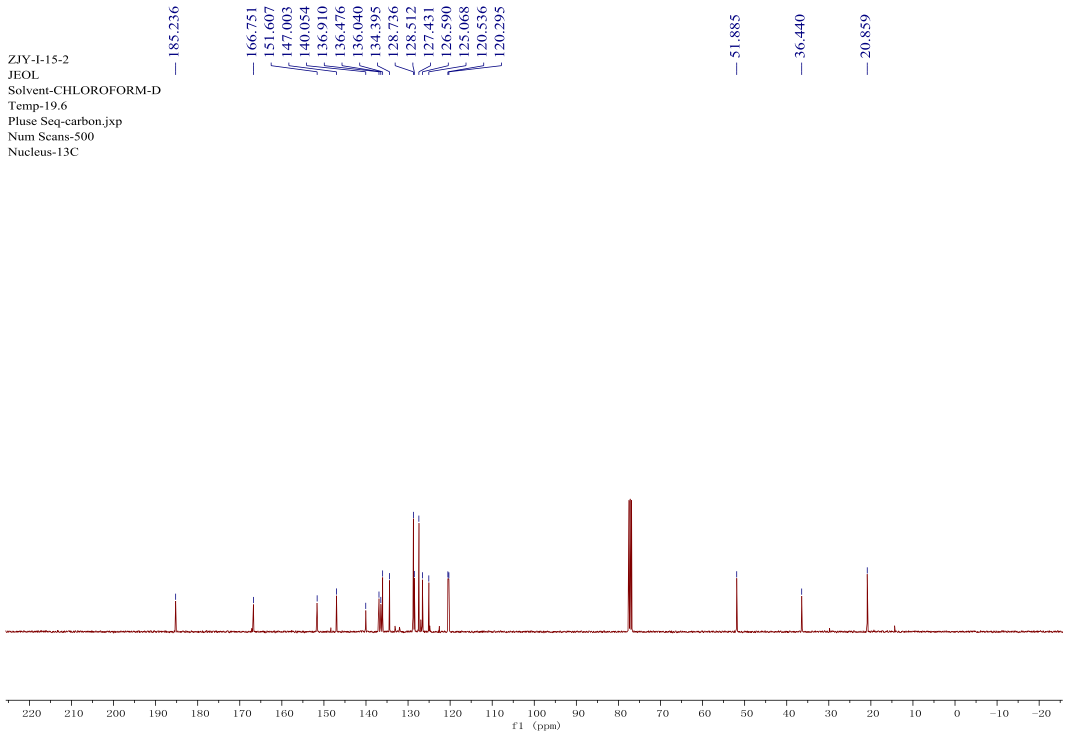

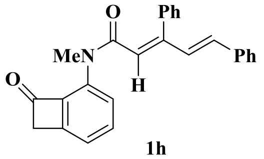

1h

^{1}H NMR of compound **1h**

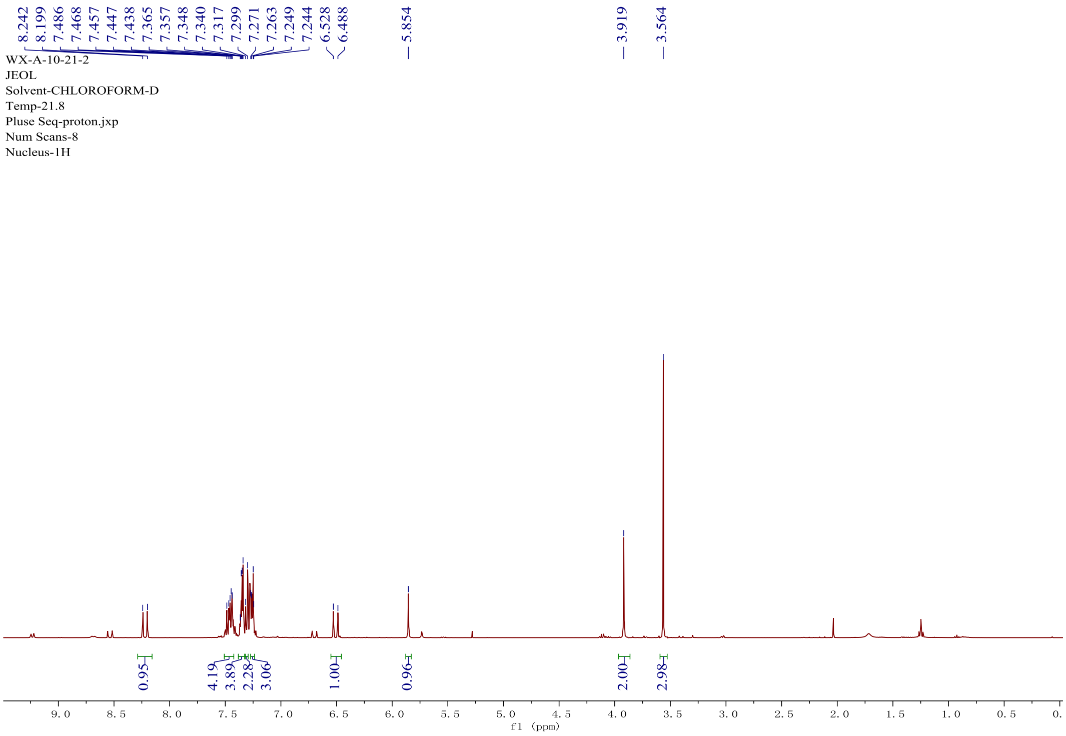

^{13}C NMR of compound **1h**

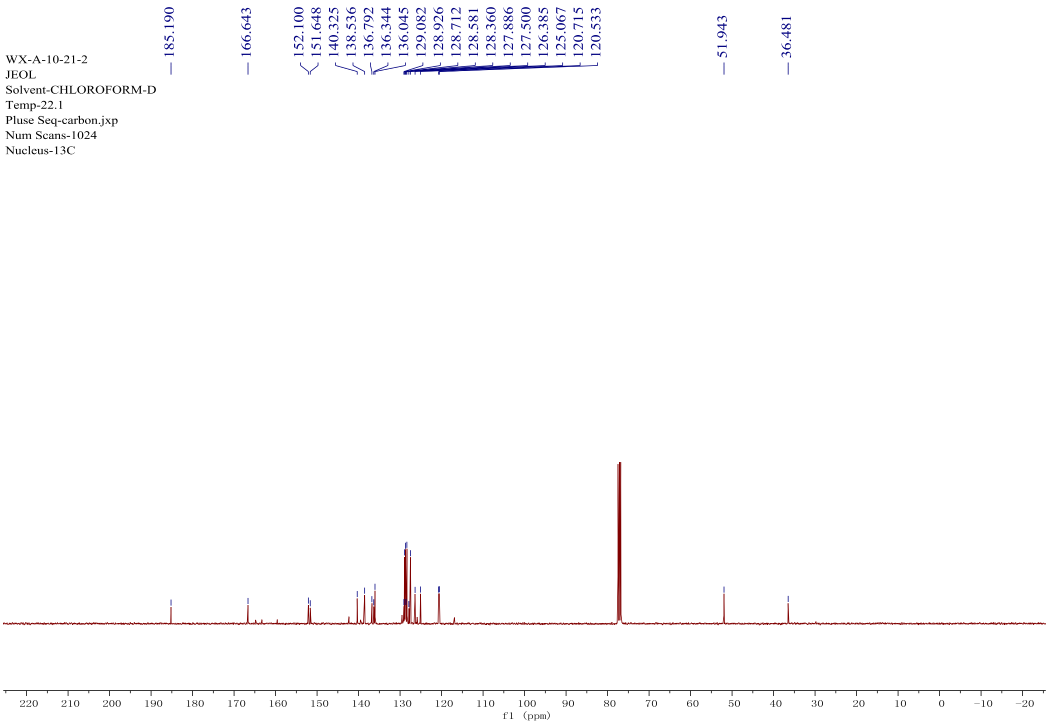

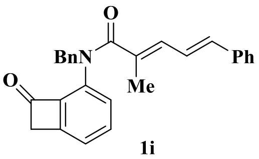

1i

^{1}H NMR of compound **1i**

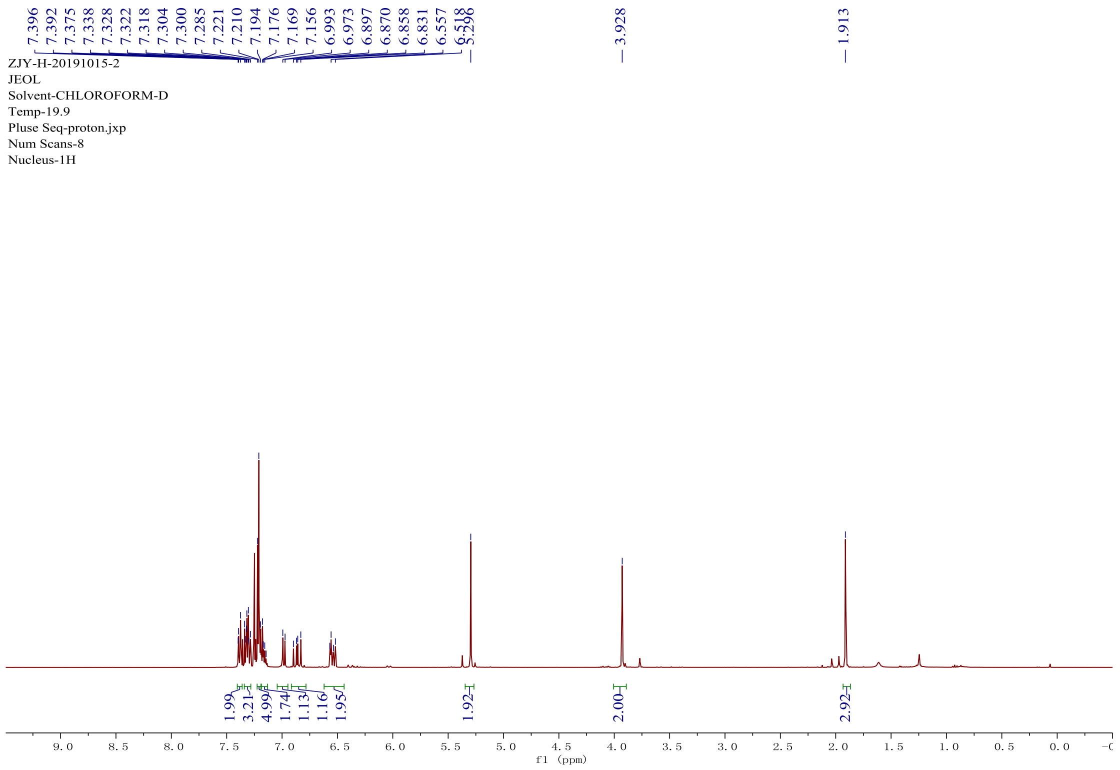

^{13}C NMR of compound **1i**

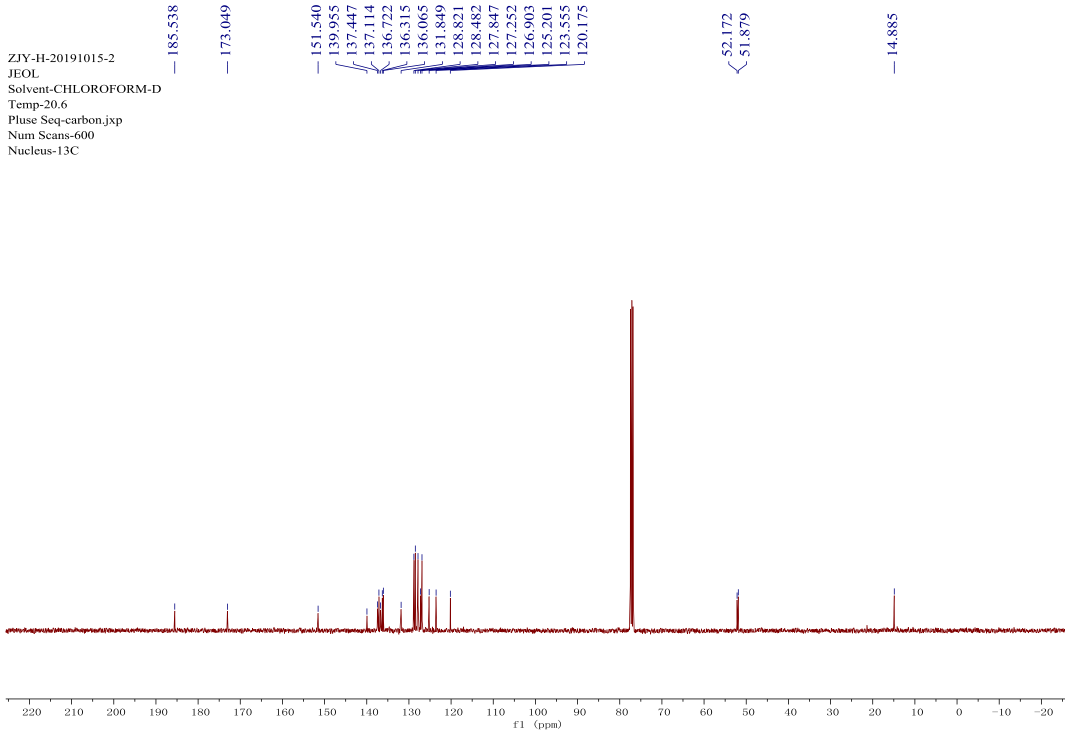

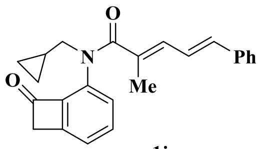

1j

^{1}H NMR of compound **1j**

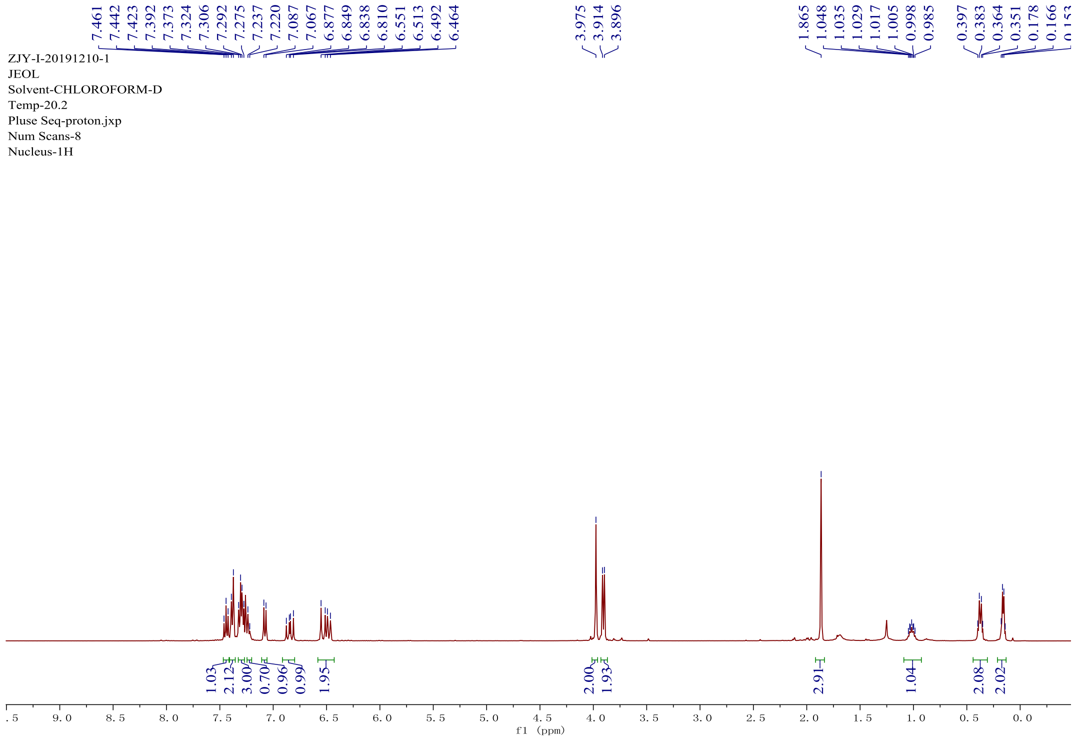

^{13}C NMR of compound **1j**

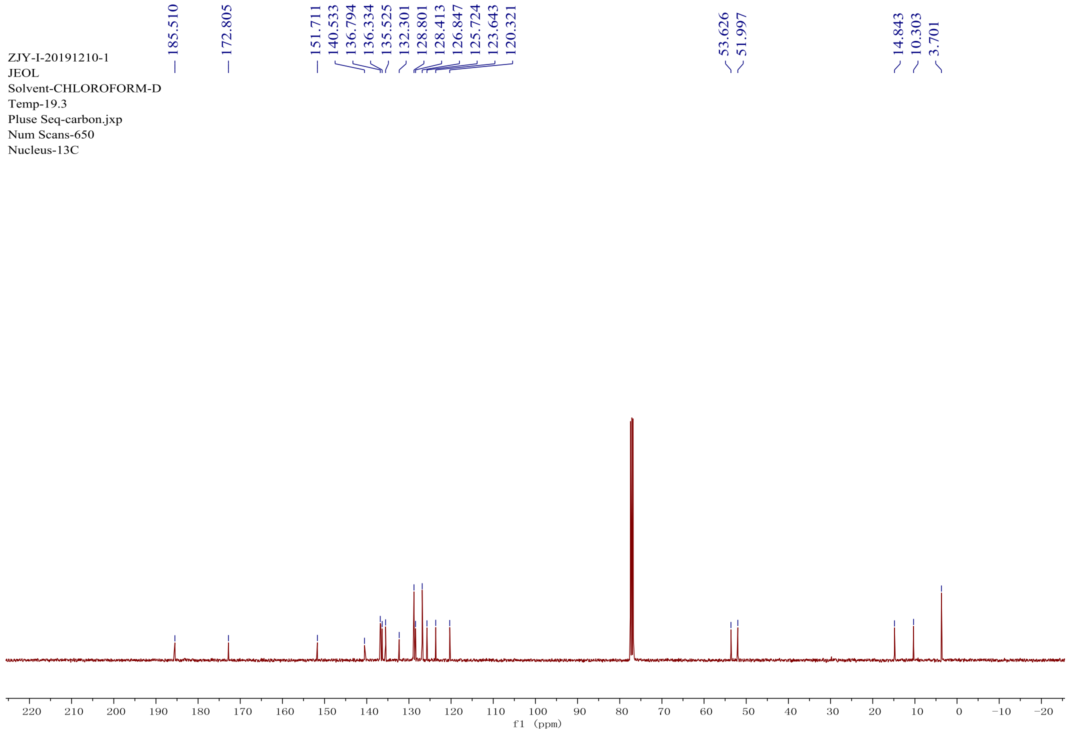

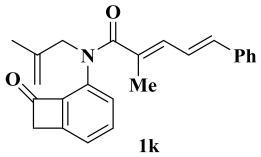

1k

^{1}H NMR of compound **1k**

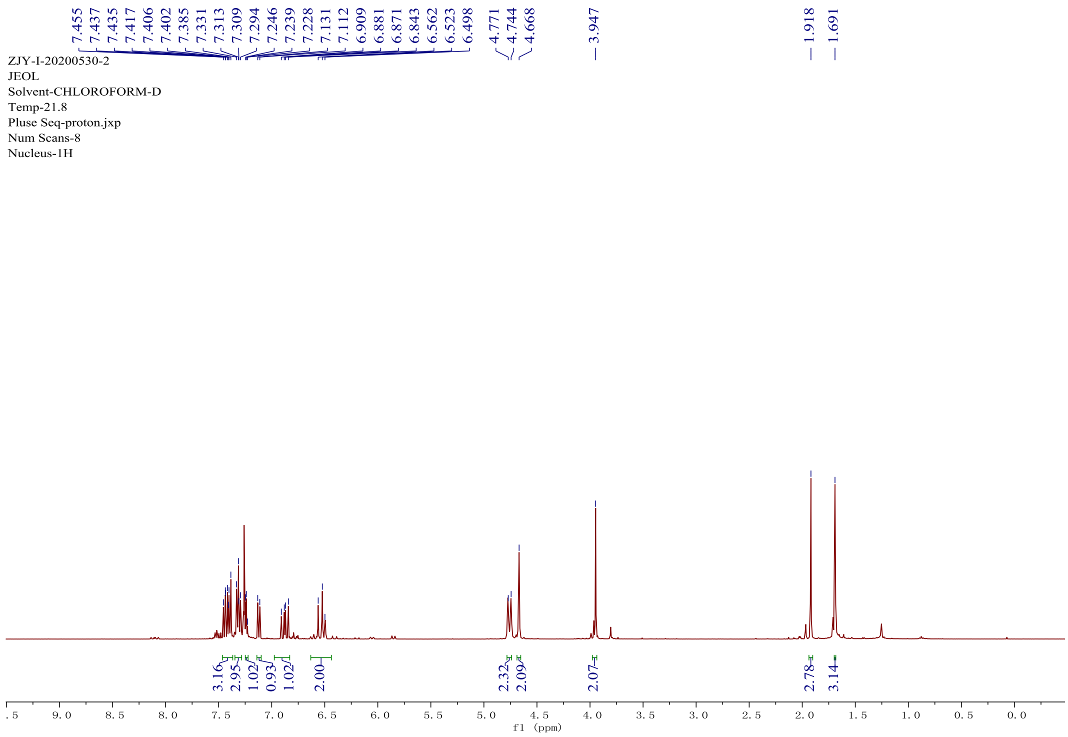

^{13}C NMR of compound **1k**

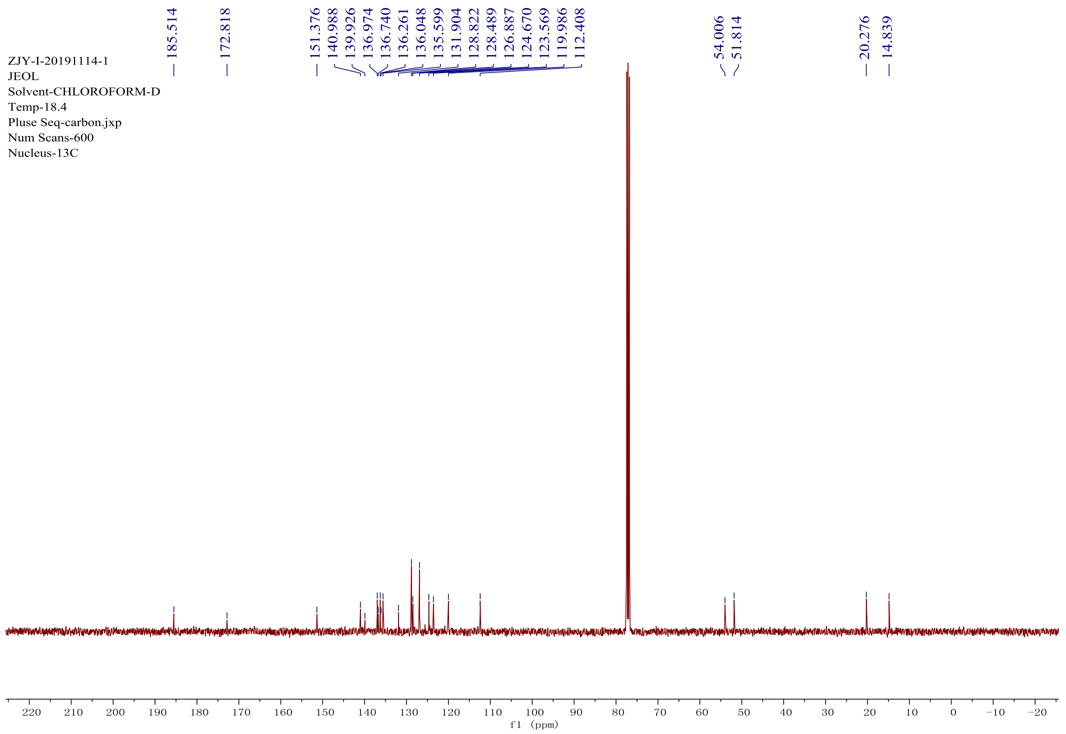

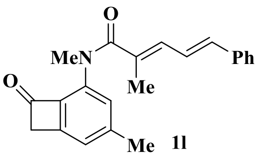

1l

^{1}H NMR of compound **1l**

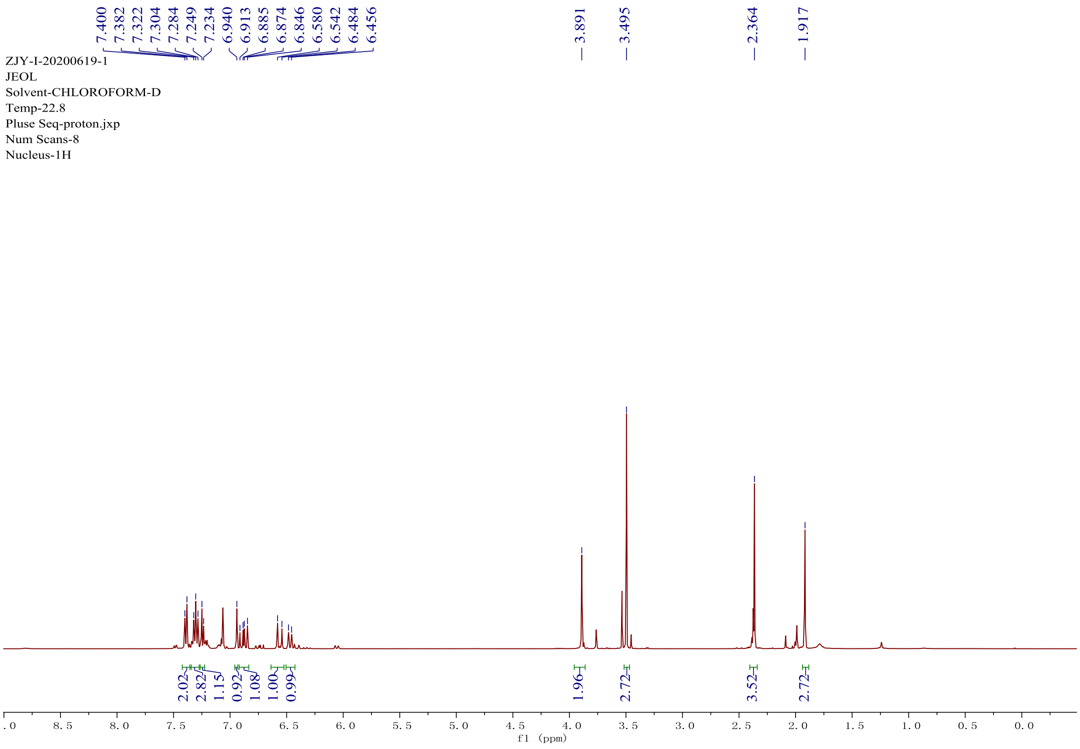

^{13}C NMR of compound **1l**

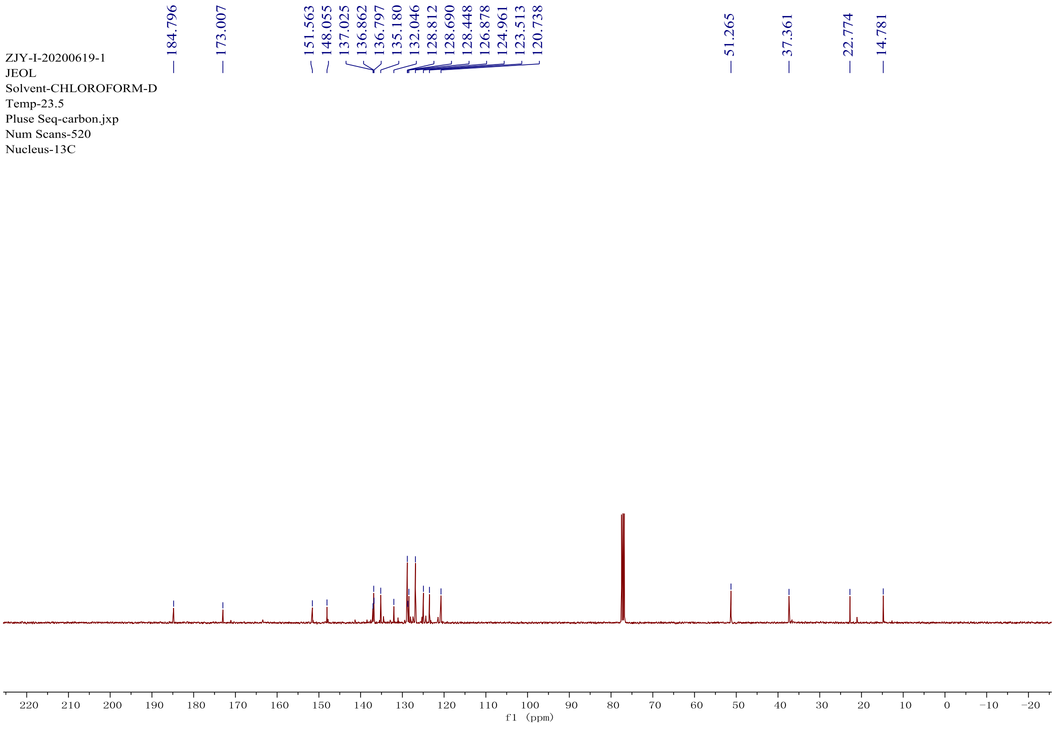

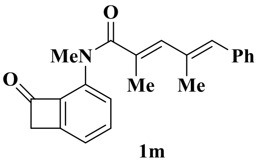

1m

^{1}H NMR of compound **1m**

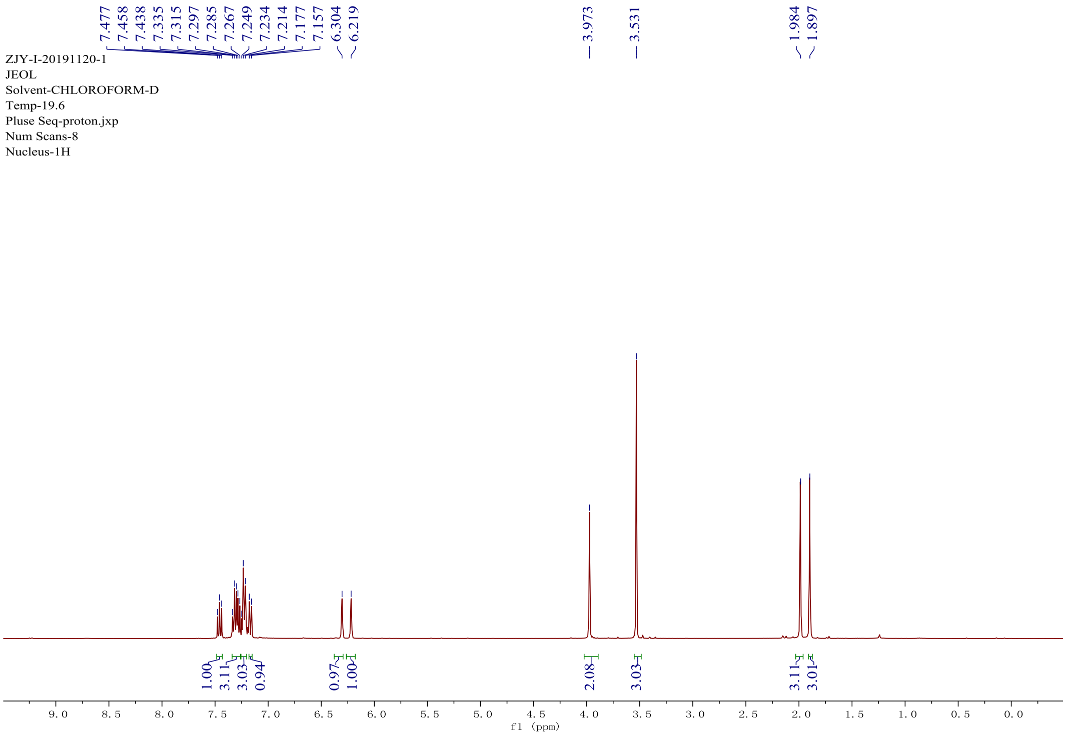

^{13}C NMR of compound **1m**

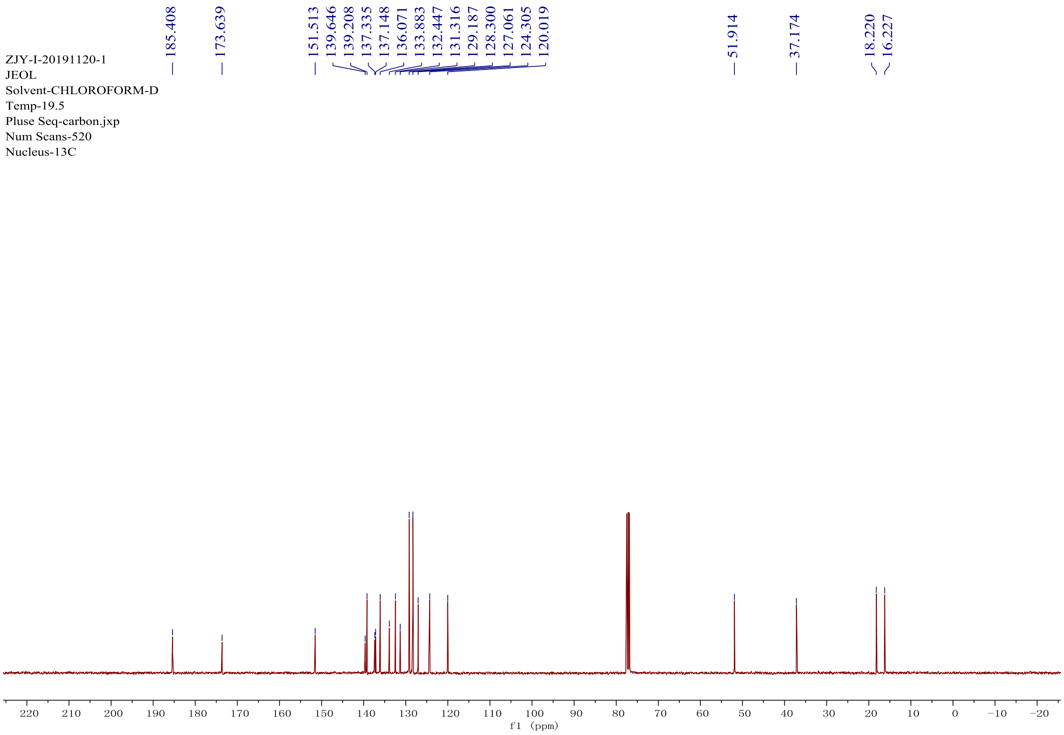

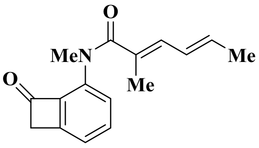

1n

^{1}H NMR of compound **1n**

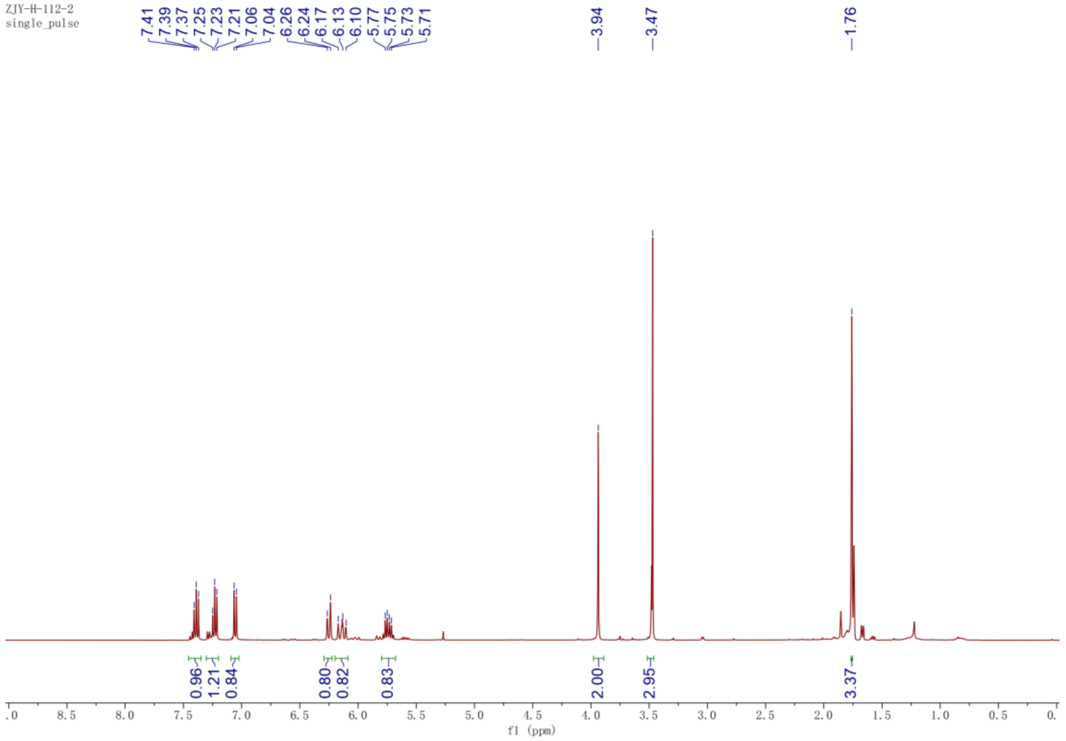

^{13}C NMR of compound **1n**

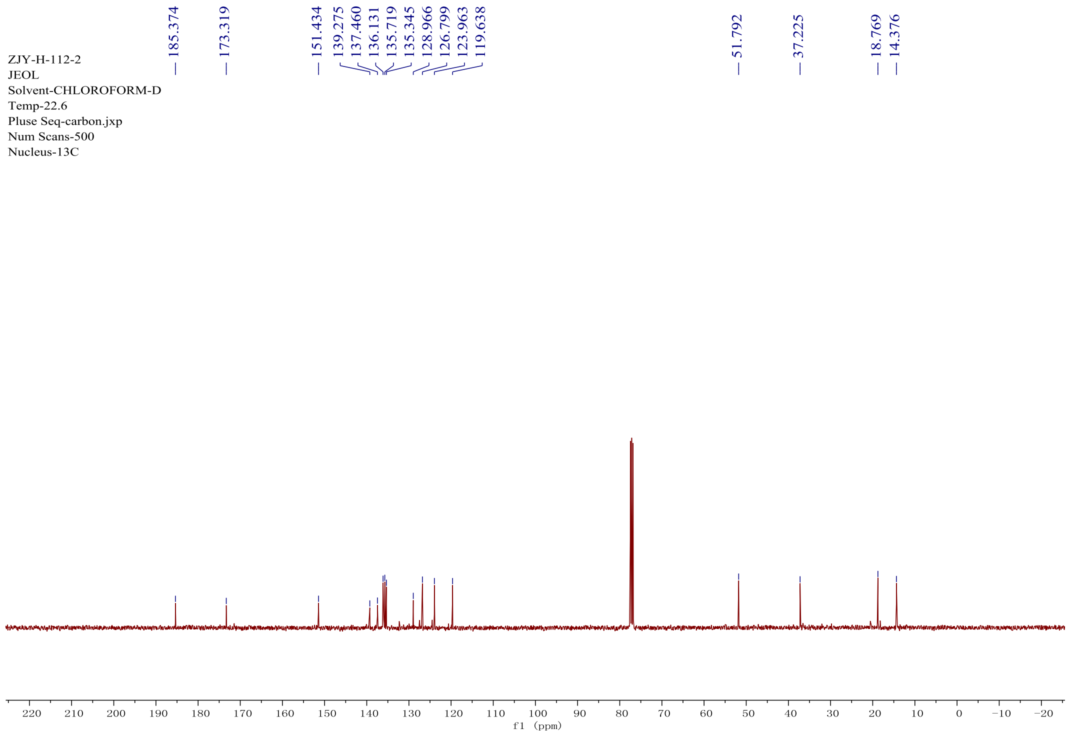

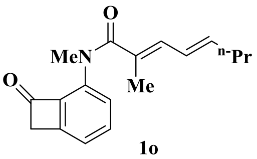

1o

^{1}H NMR of compound **1o**

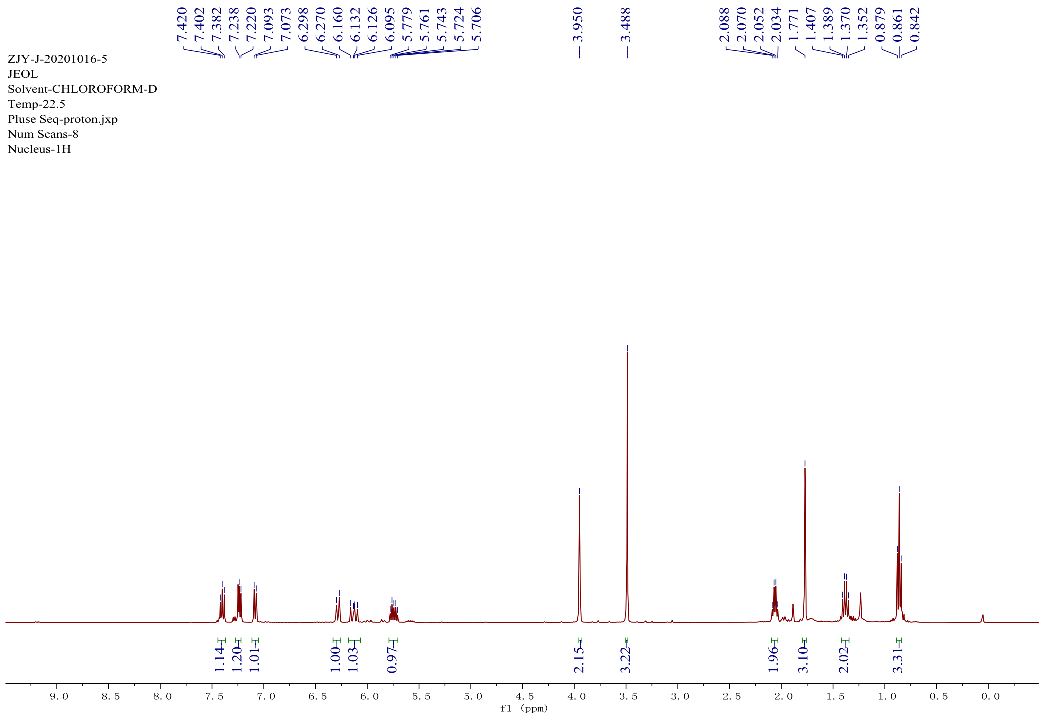

^{13}C NMR of compound **1o**

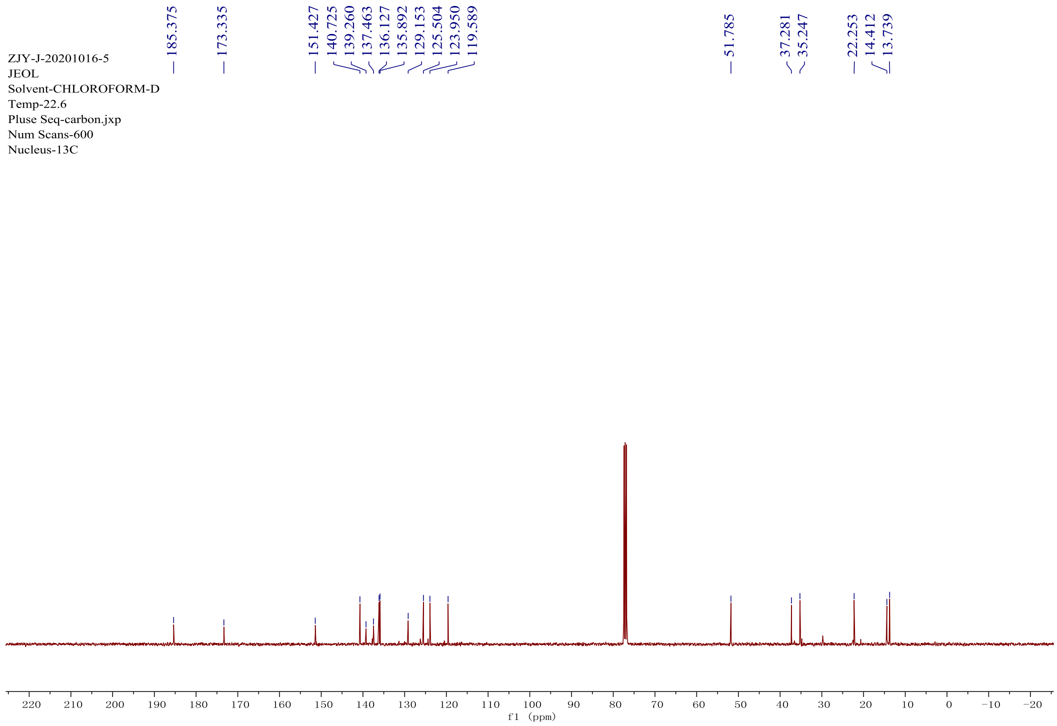

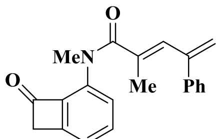

1p

^{1}H NMR of compound **1p**

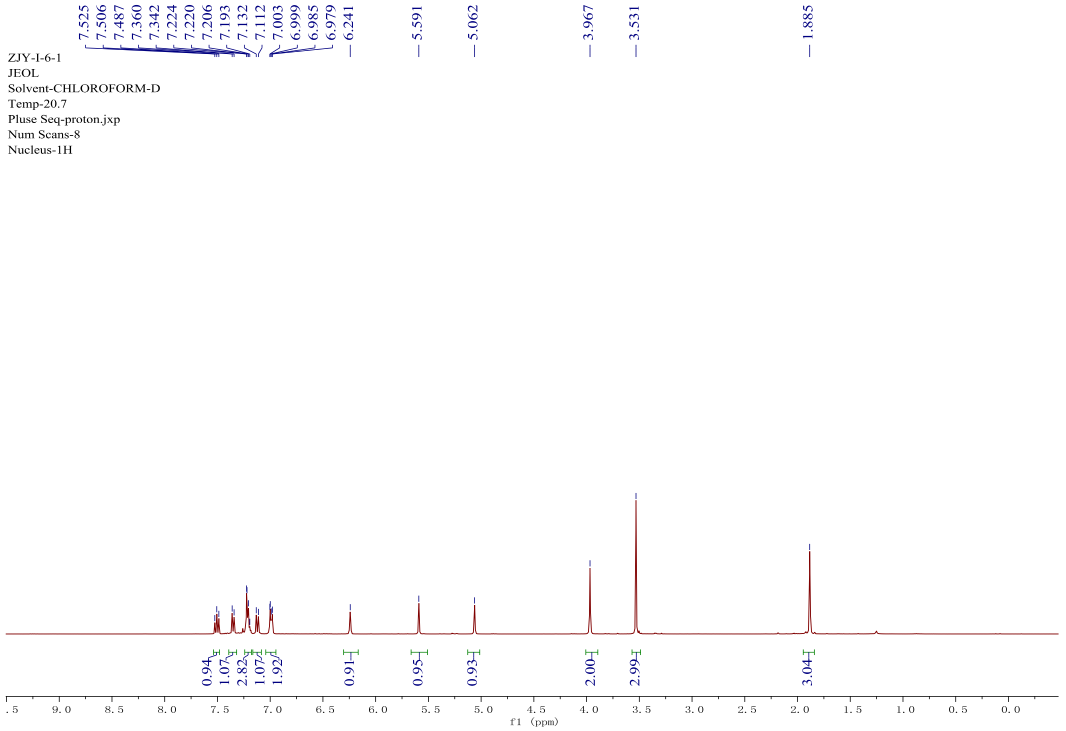

^{13}C NMR of compound **1p**

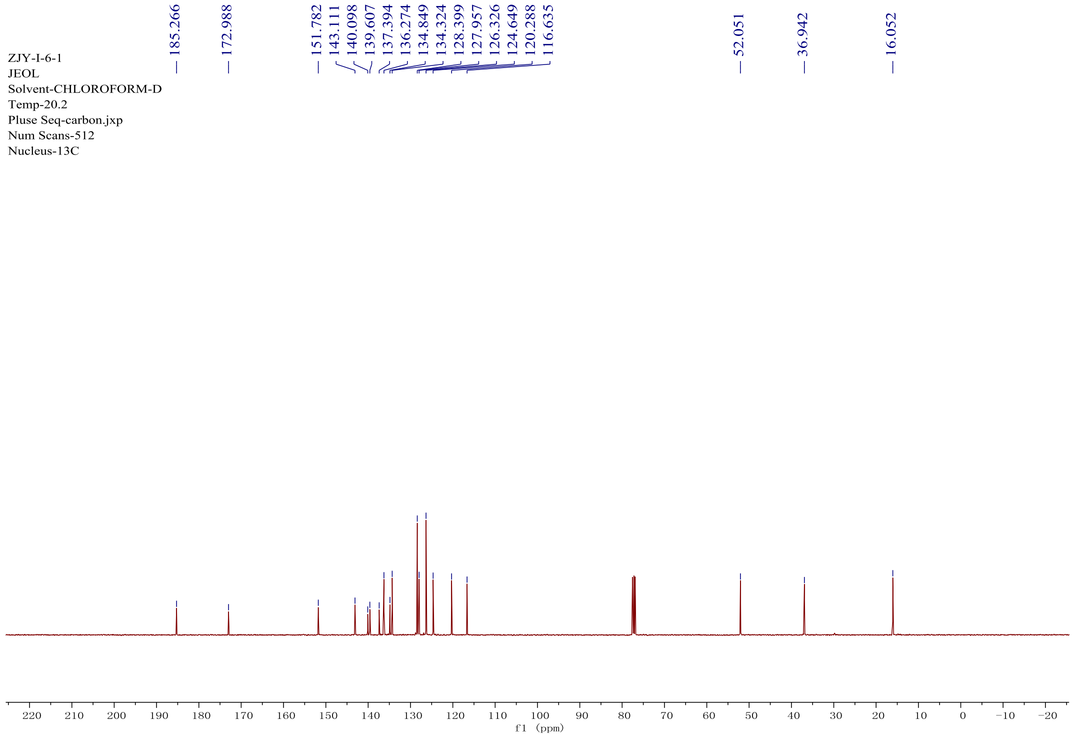

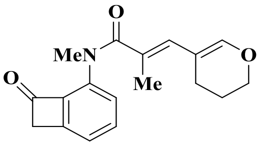

1q

^{1}H NMR of compound **1q**

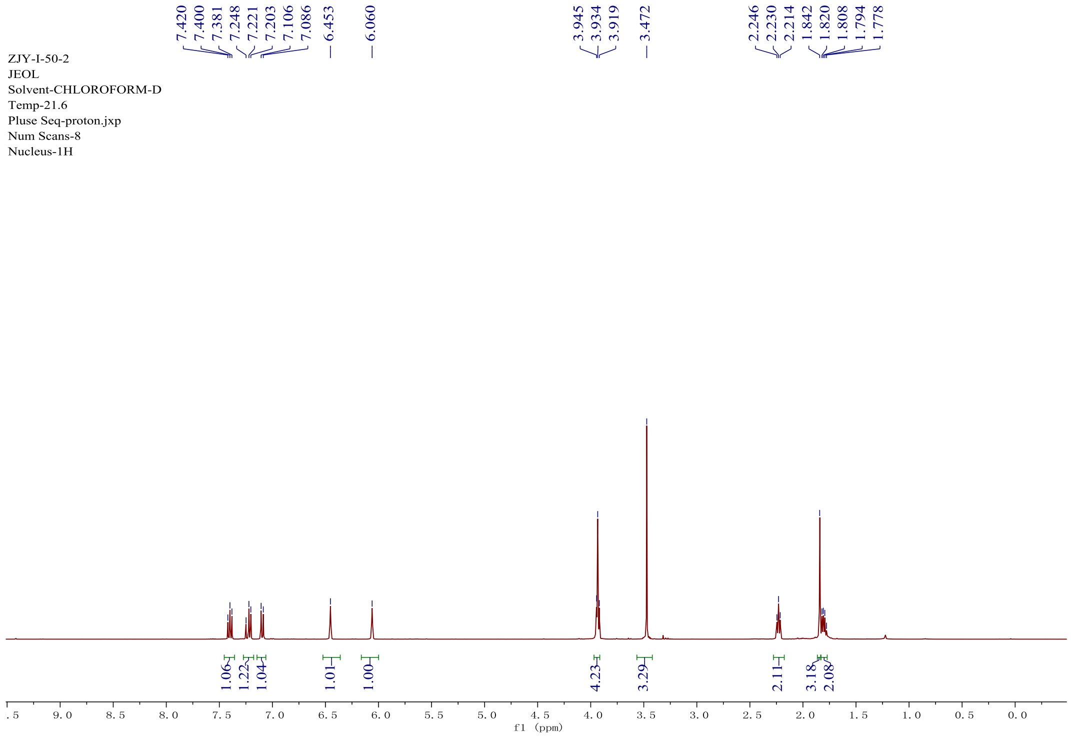

^{13}C NMR of compound **1q**

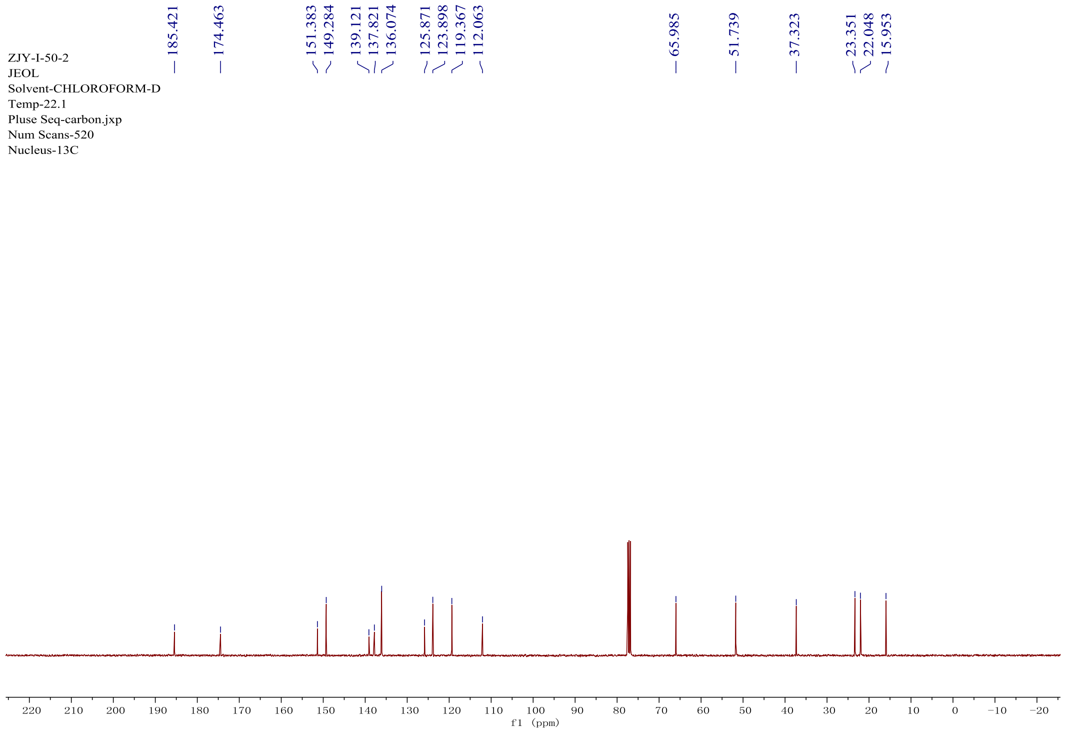

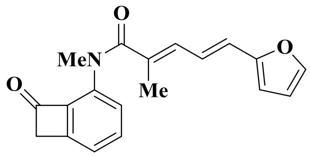

1r

^{1}H NMR of compound **1r**

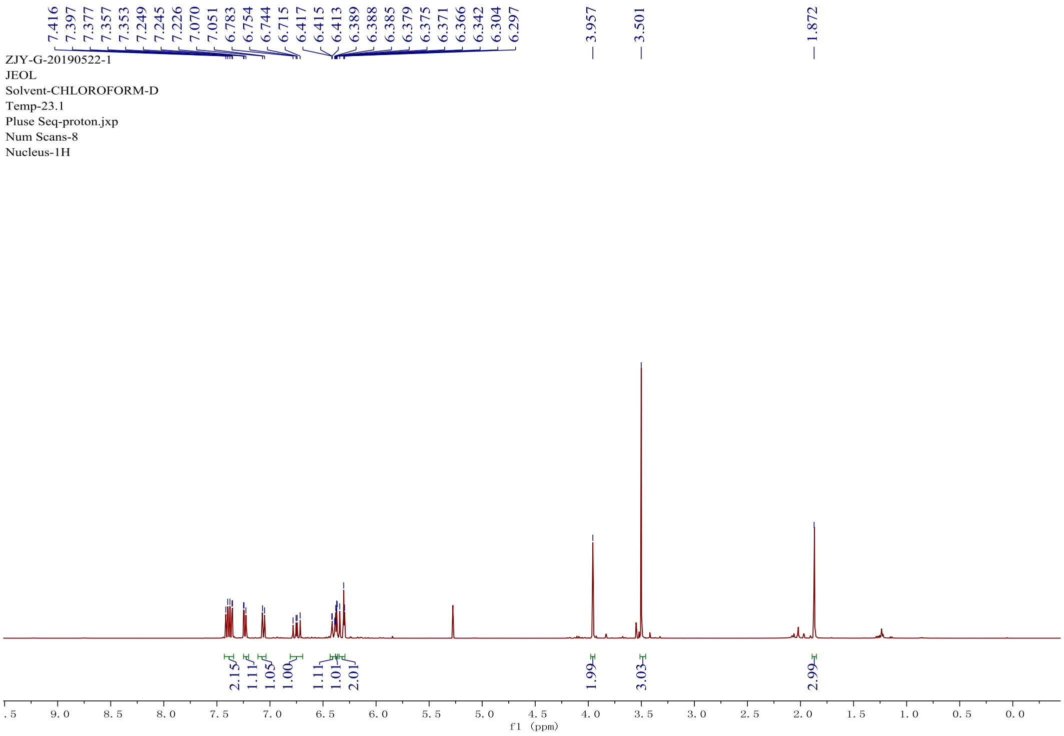

^{13}C NMR of compound **1r**

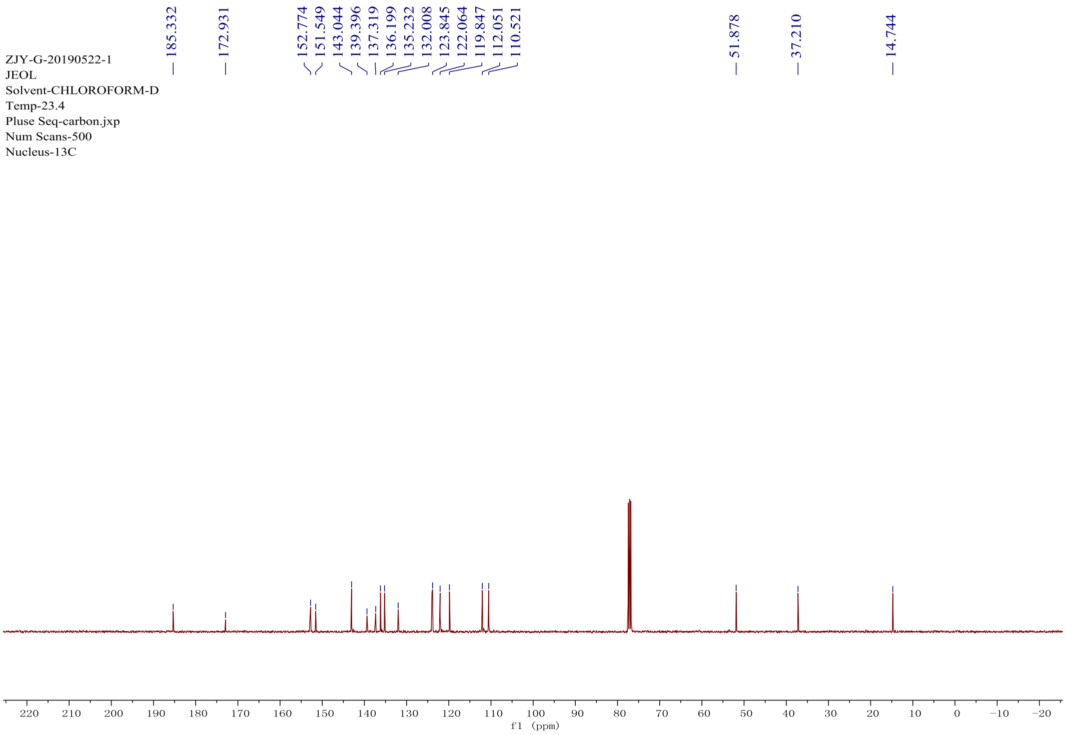

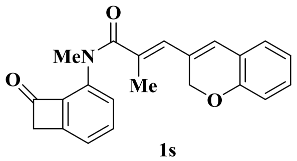

1s

^{1}H NMR of compound **1s**

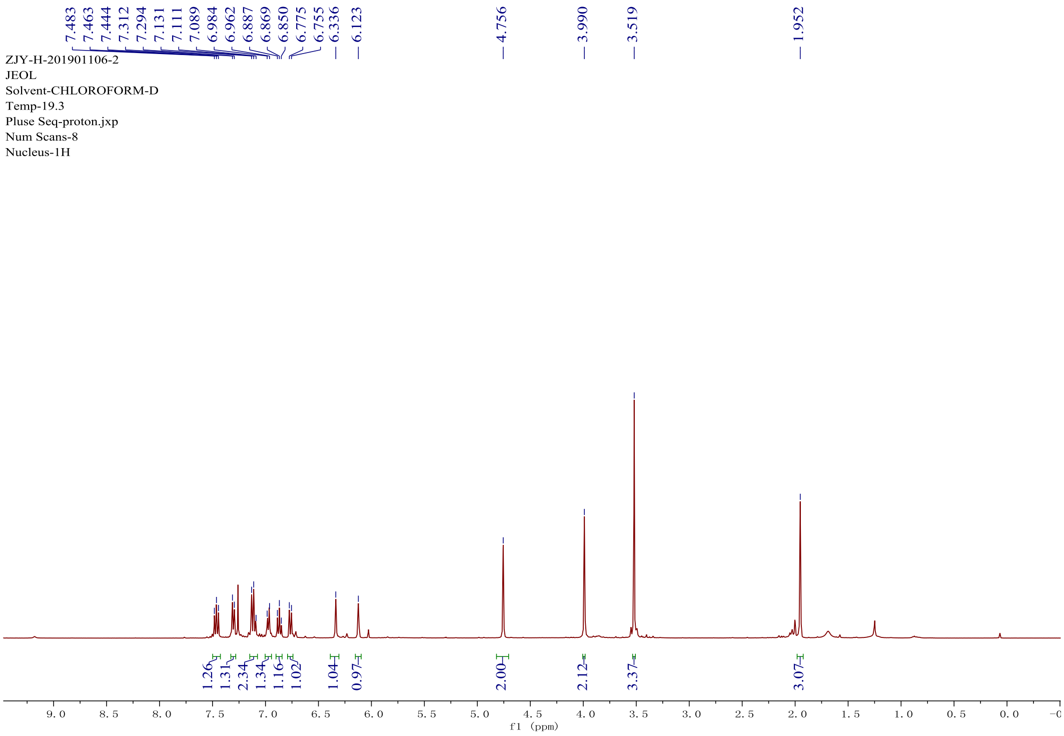

^{13}C NMR of compound **1s**

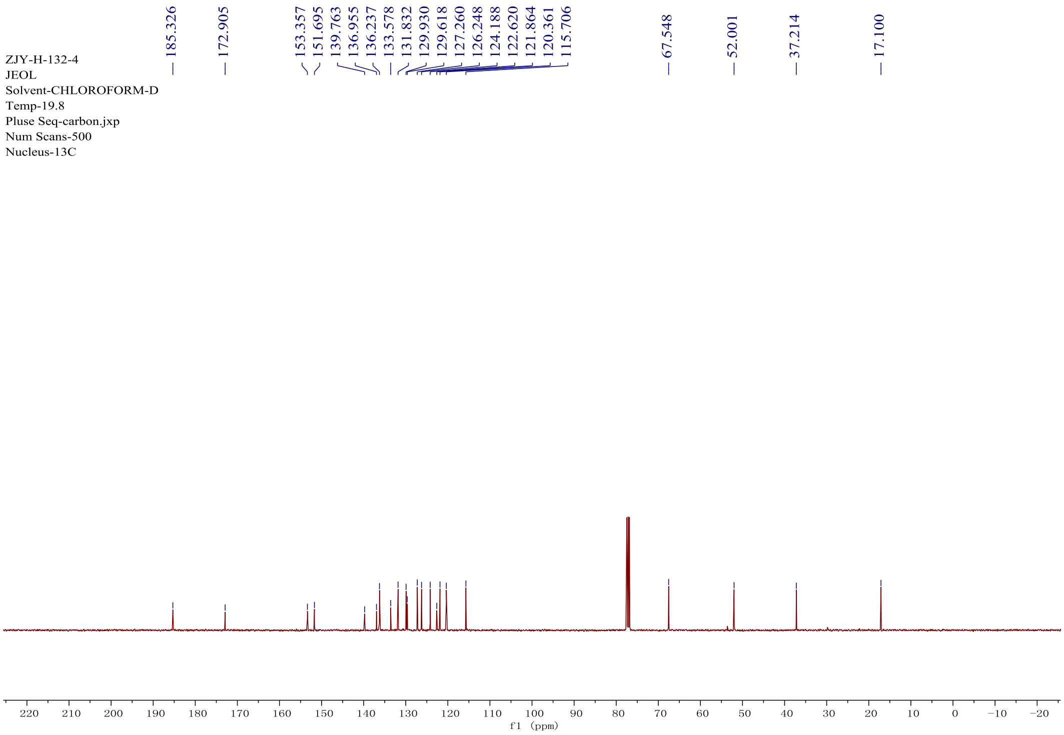

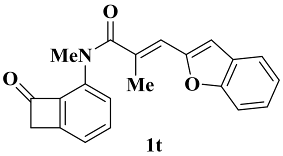

1t

^{1}H NMR of compound **1t**

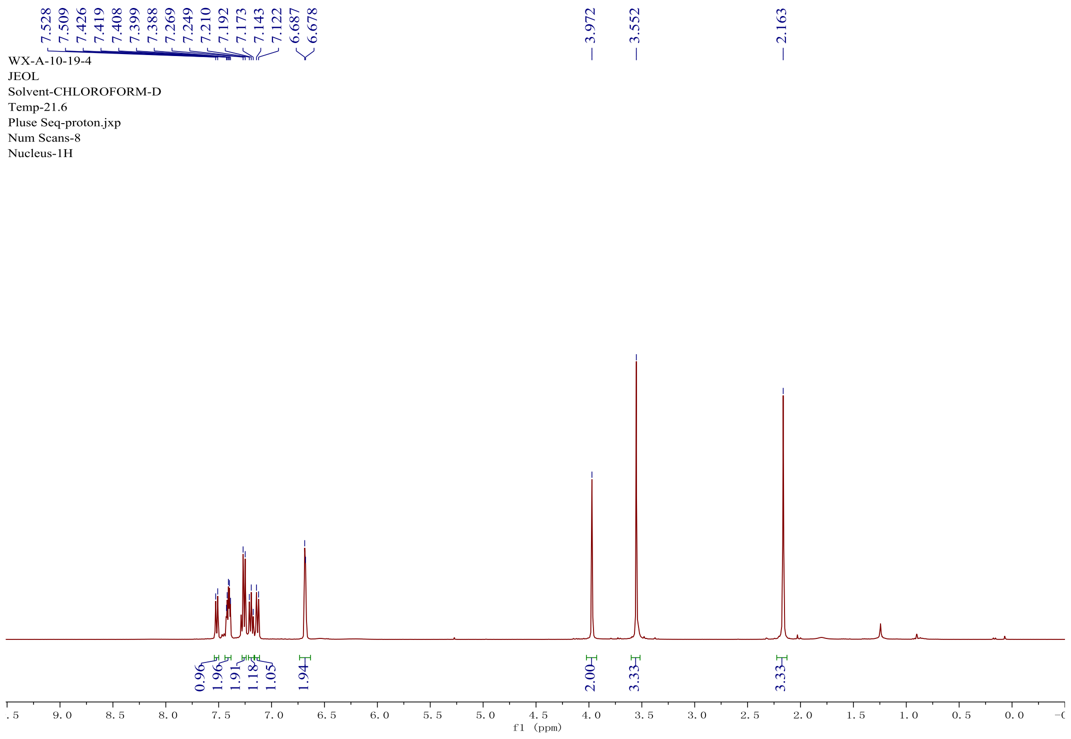

^{13}C NMR of compound **1t**

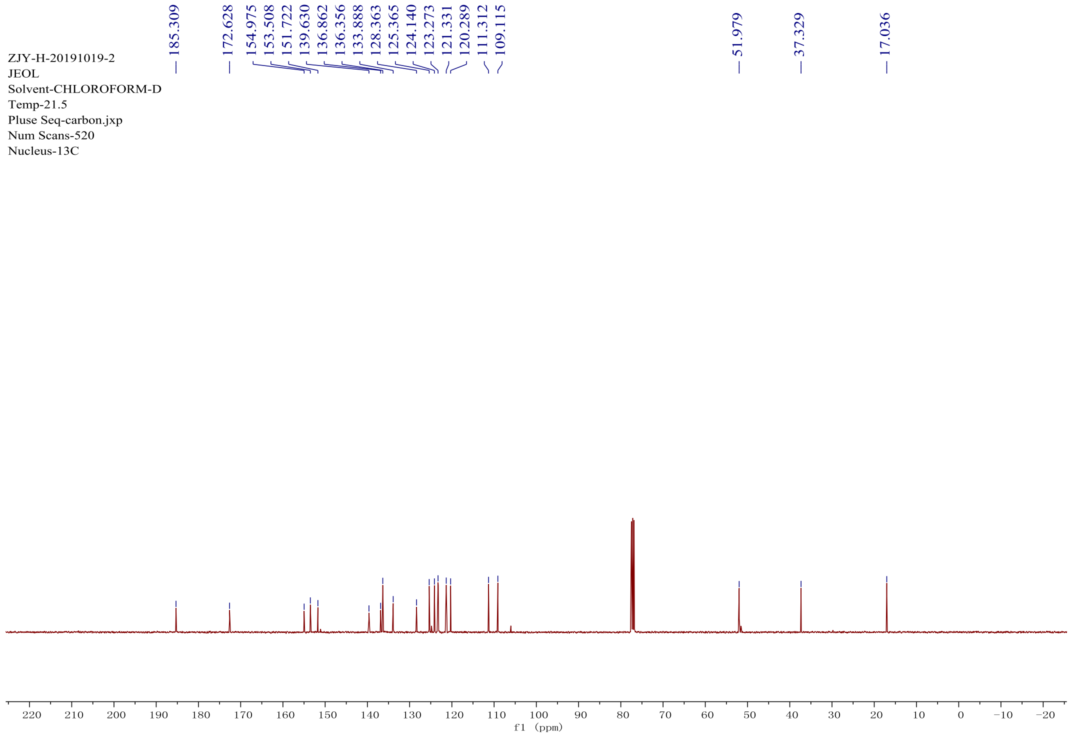

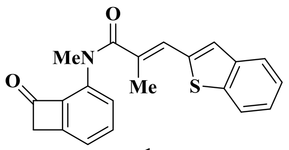

1u

^{1}H NMR of compound **1u**

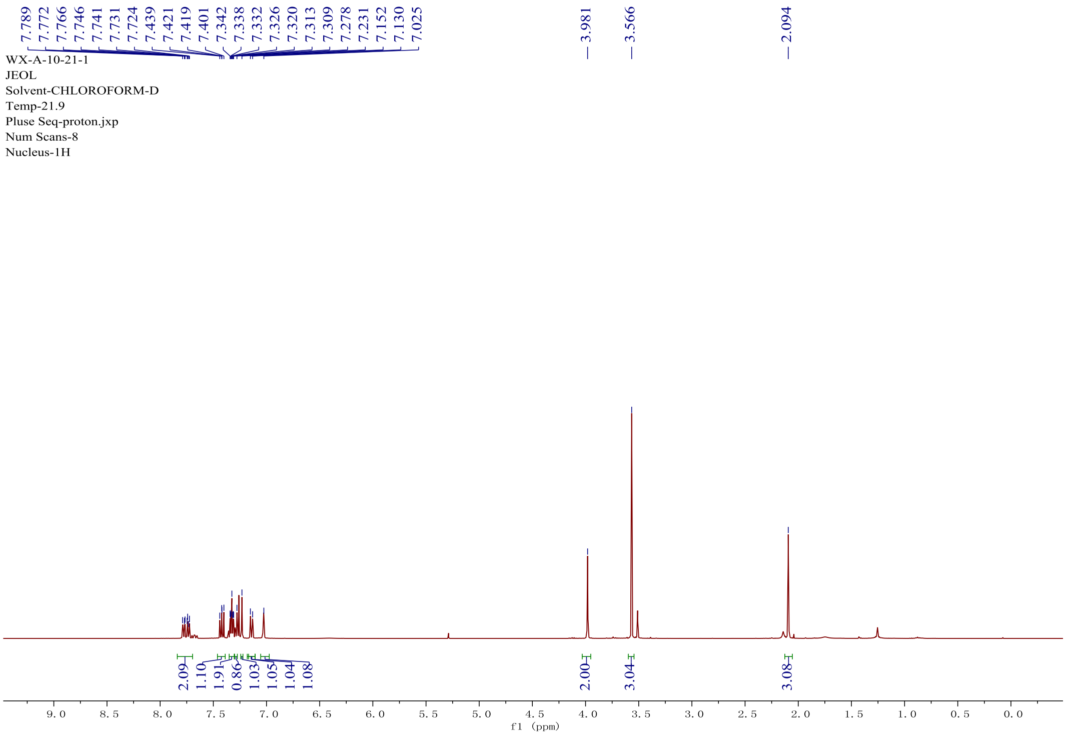

^{13}C NMR of compound **1u**

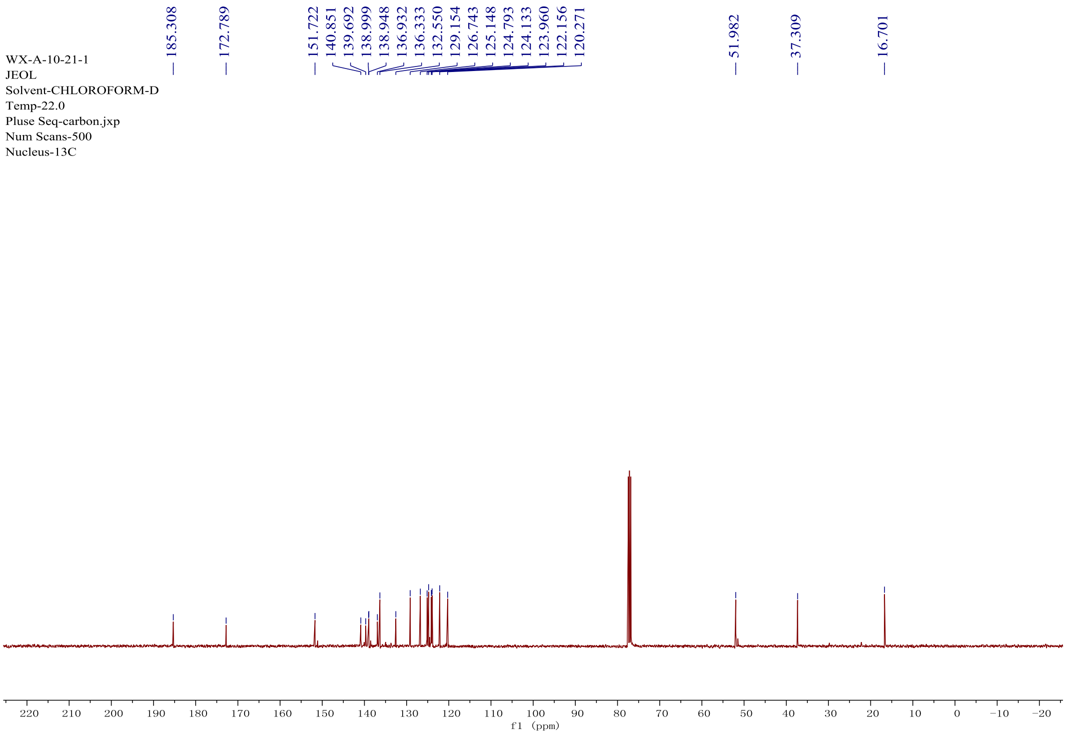

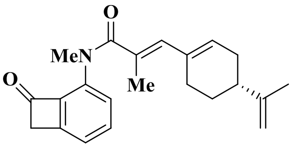

1v

^{1}H NMR of compound **1v**

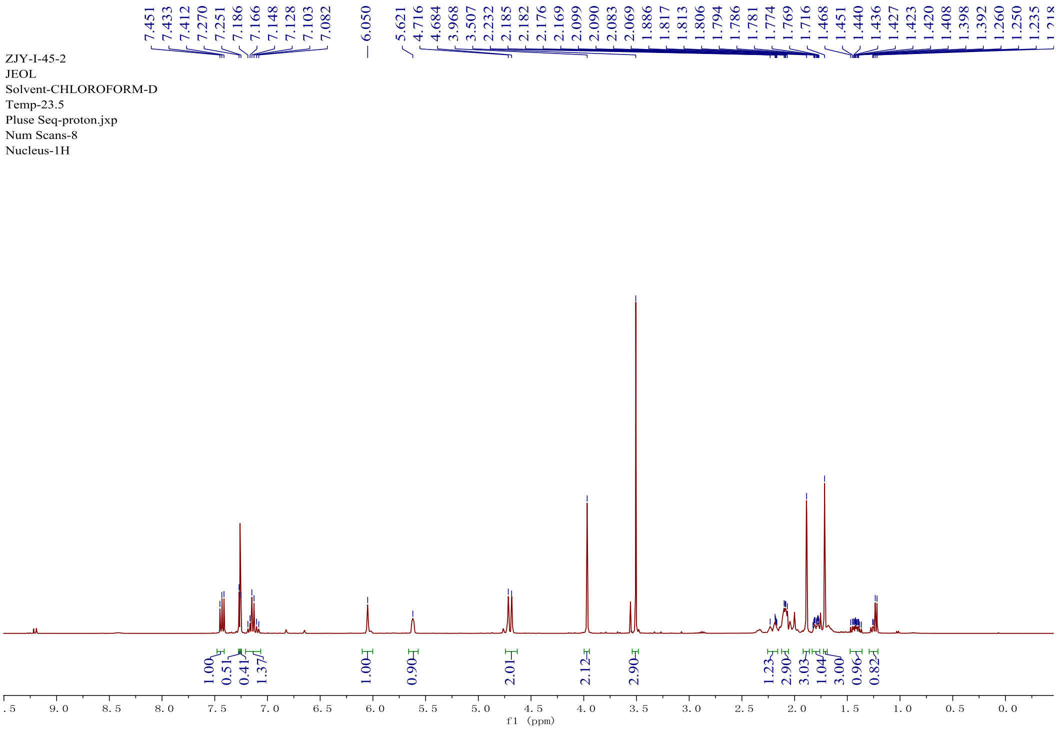

^{13}C NMR of compound **1v**

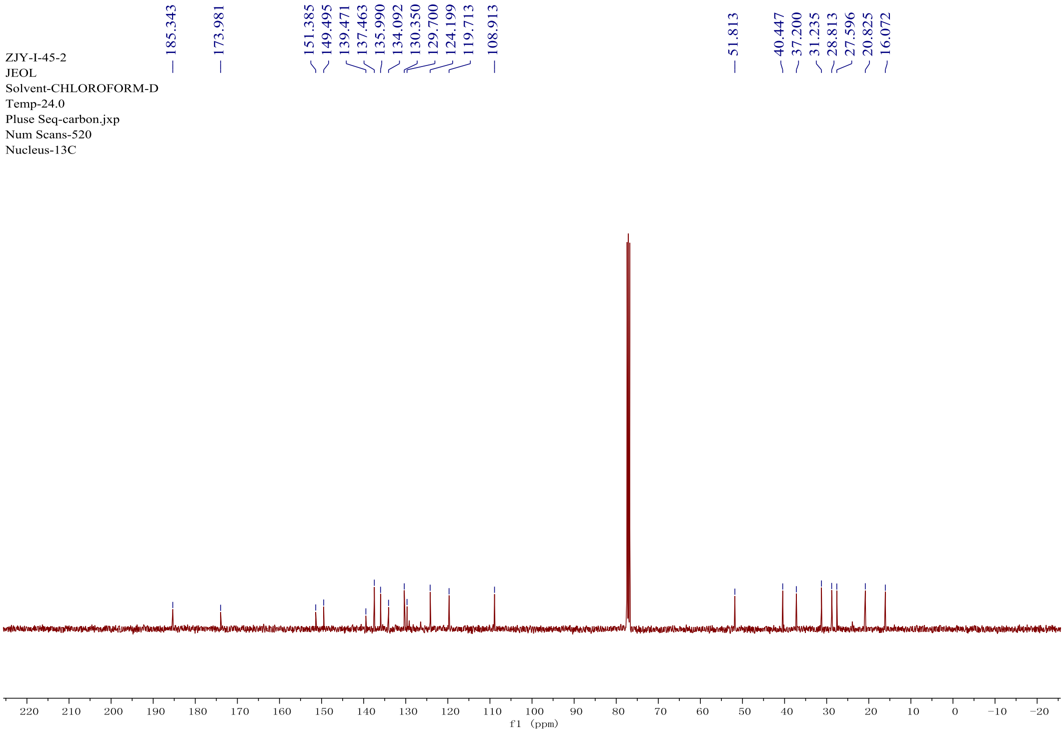

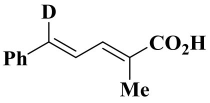

S44

^{1}H NMR of compound **S44**

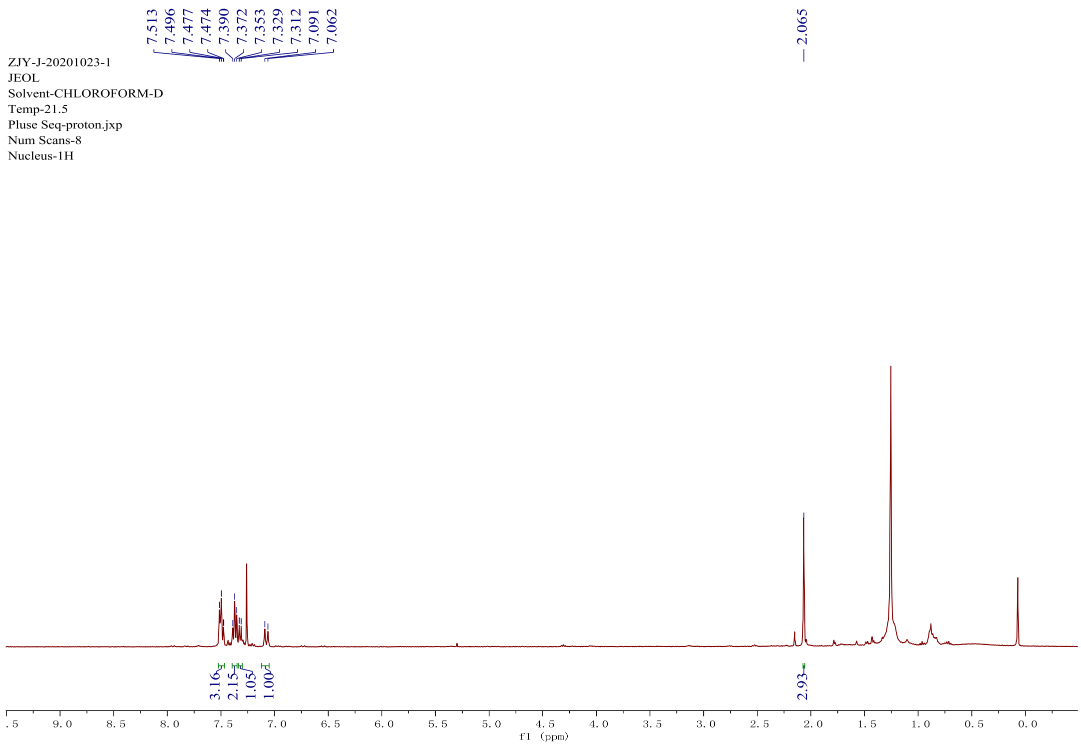

^{13}C NMR of compound **S44**

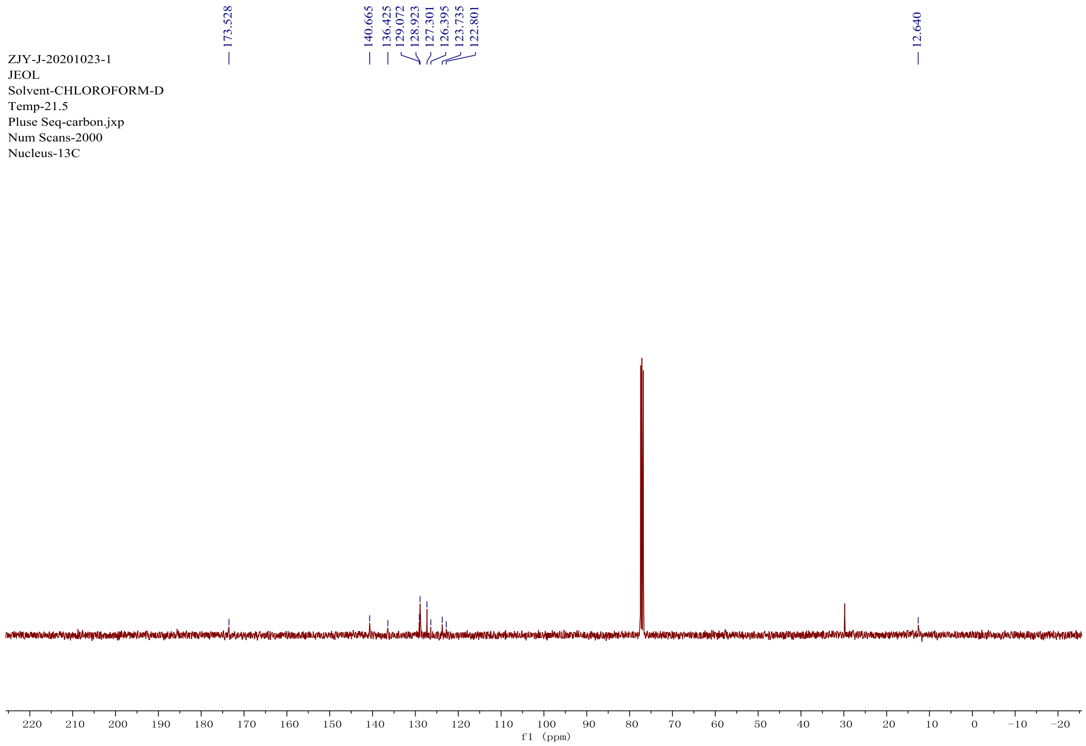

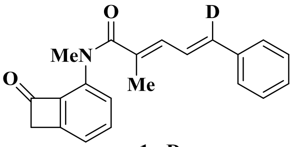

1a-D

^{1}H NMR of compound **1a-D**

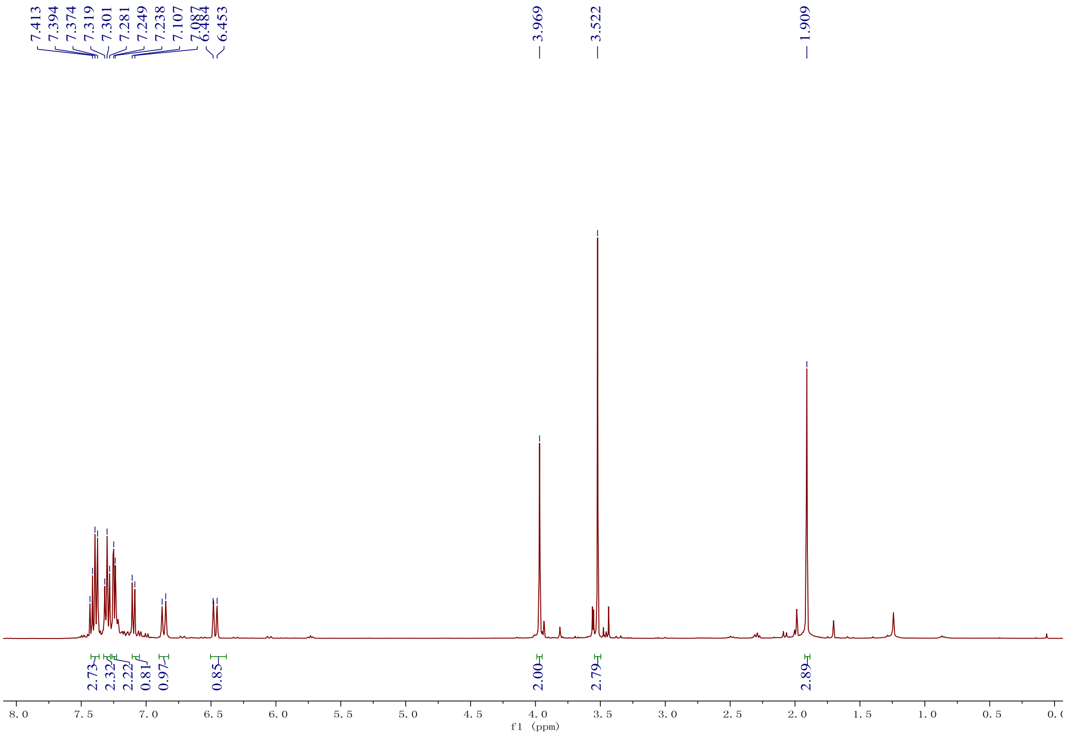

^{13}C NMR of compound **1a-D**

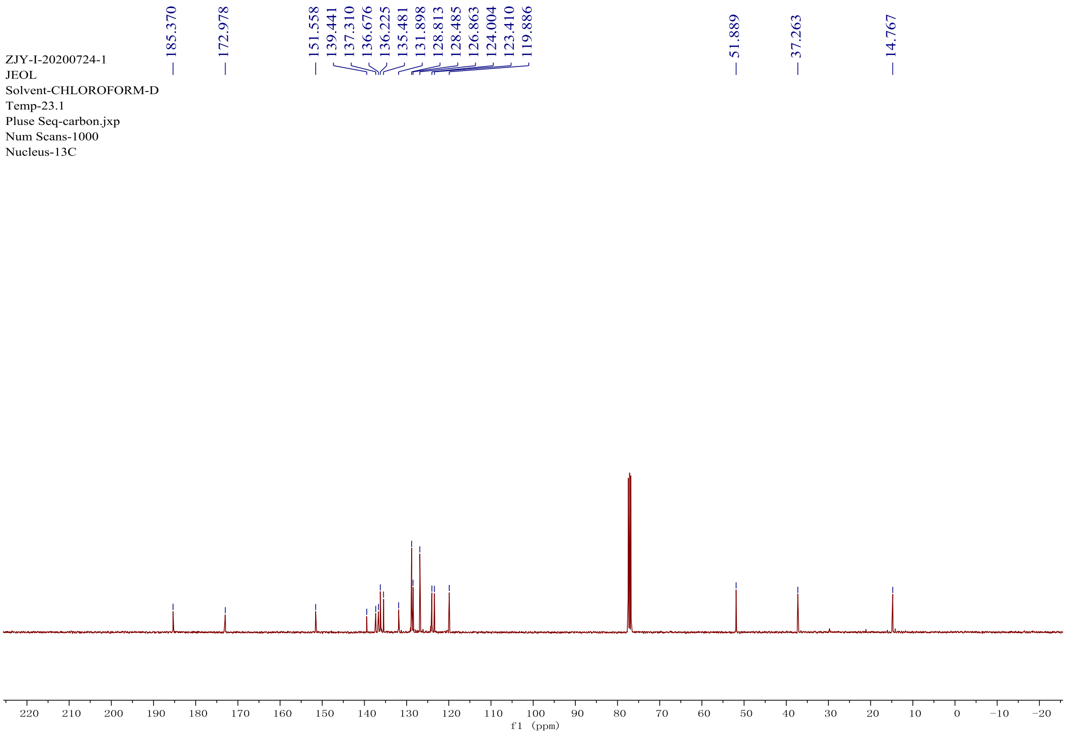

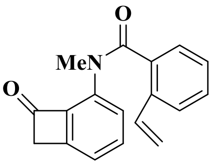

6

^{1}H NMR of compound **6**

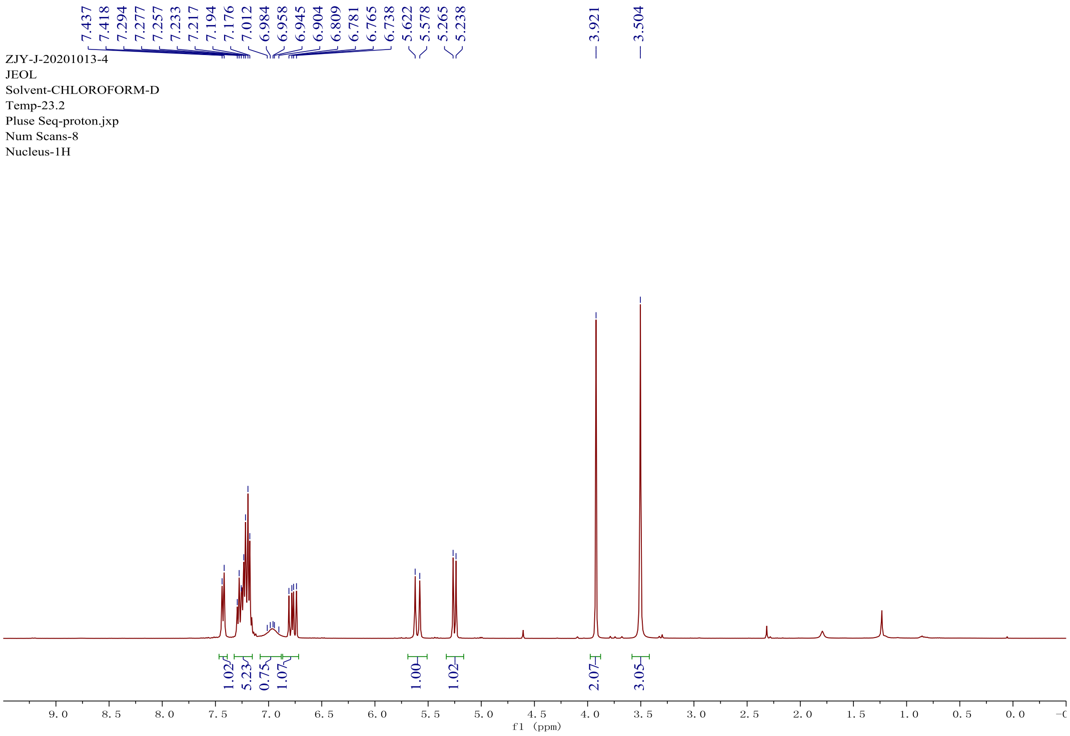

^{13}C NMR of compound **6**

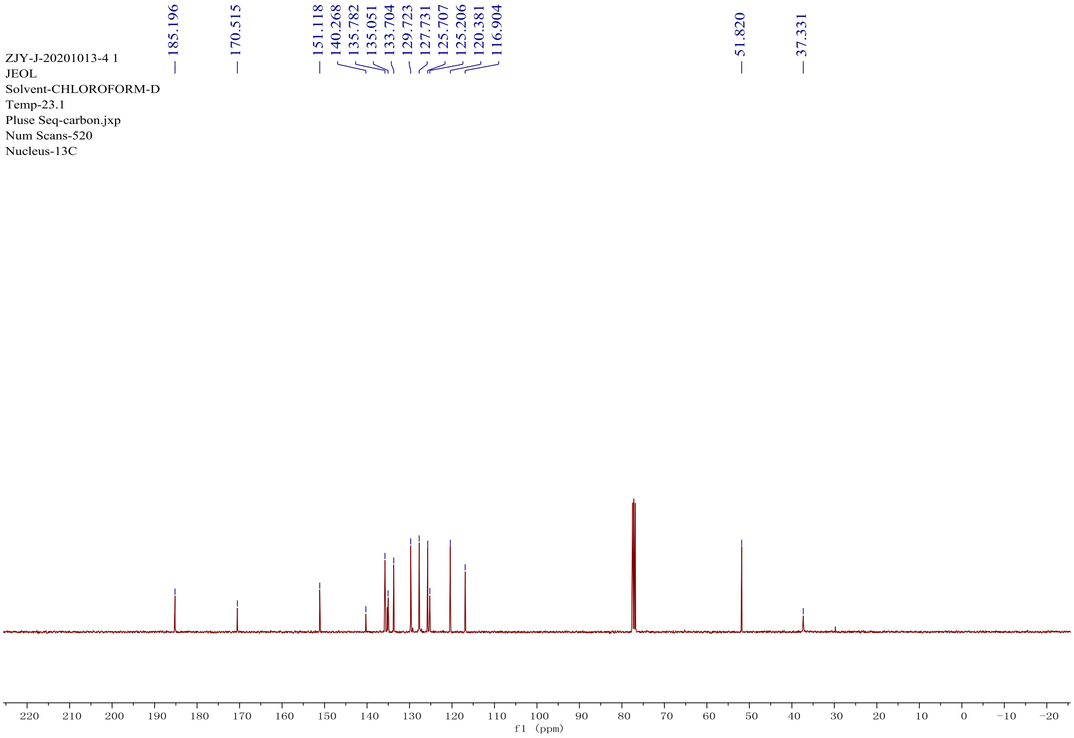

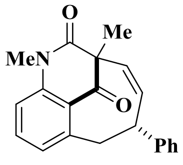

2a

^{1}H NMR of compound **2a**

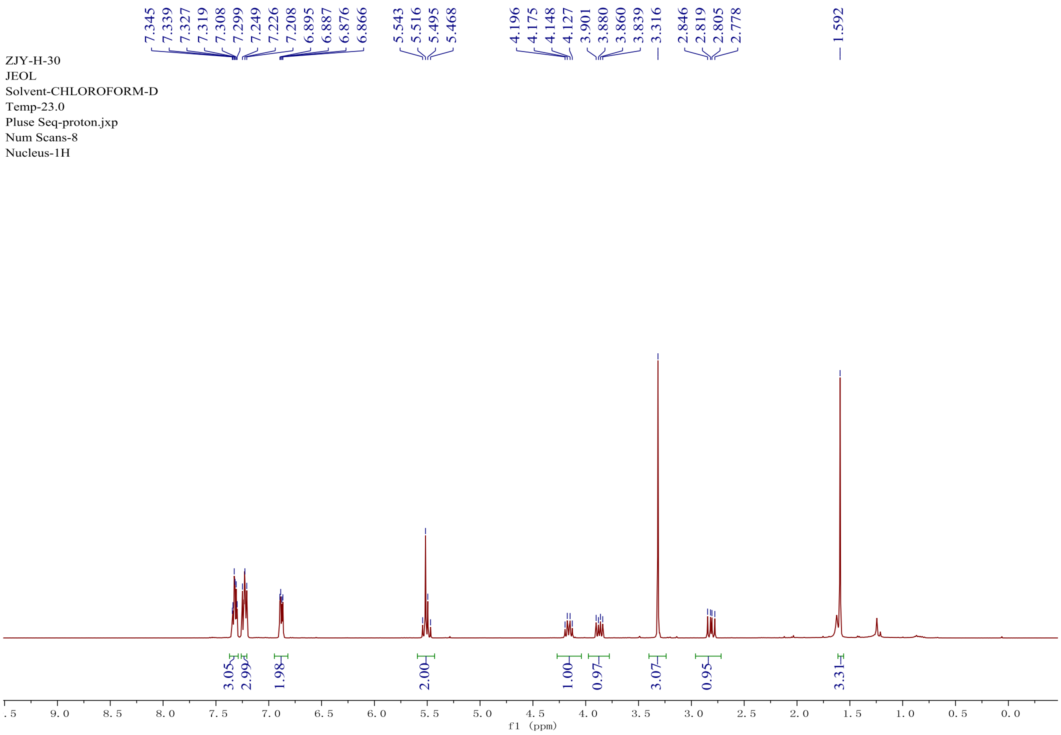

^{13}C NMR of compound **2a**

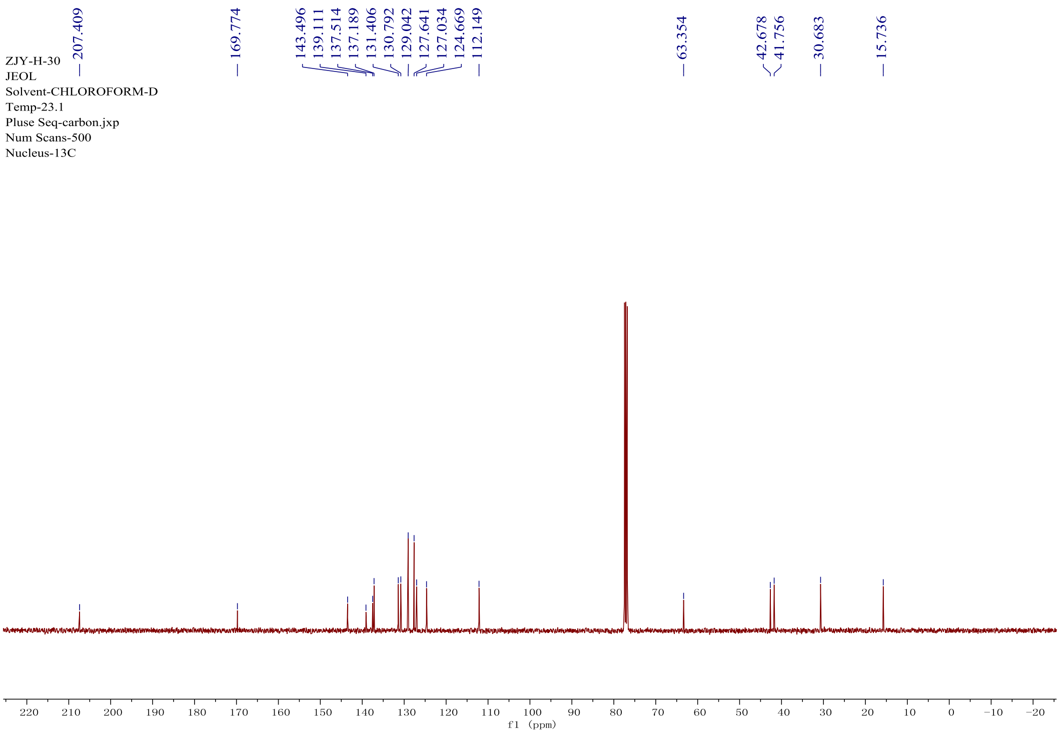

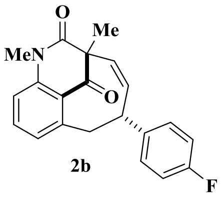

2b

^{1}H NMR of compound **2b**

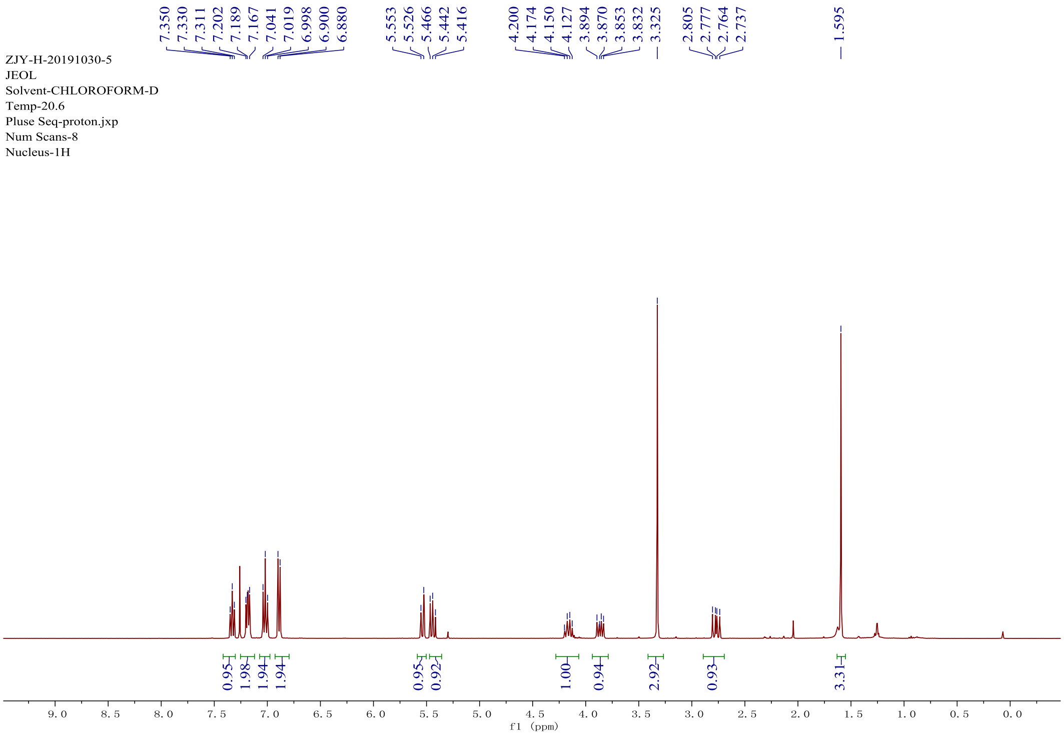

^{13}C NMR of compound **2b**

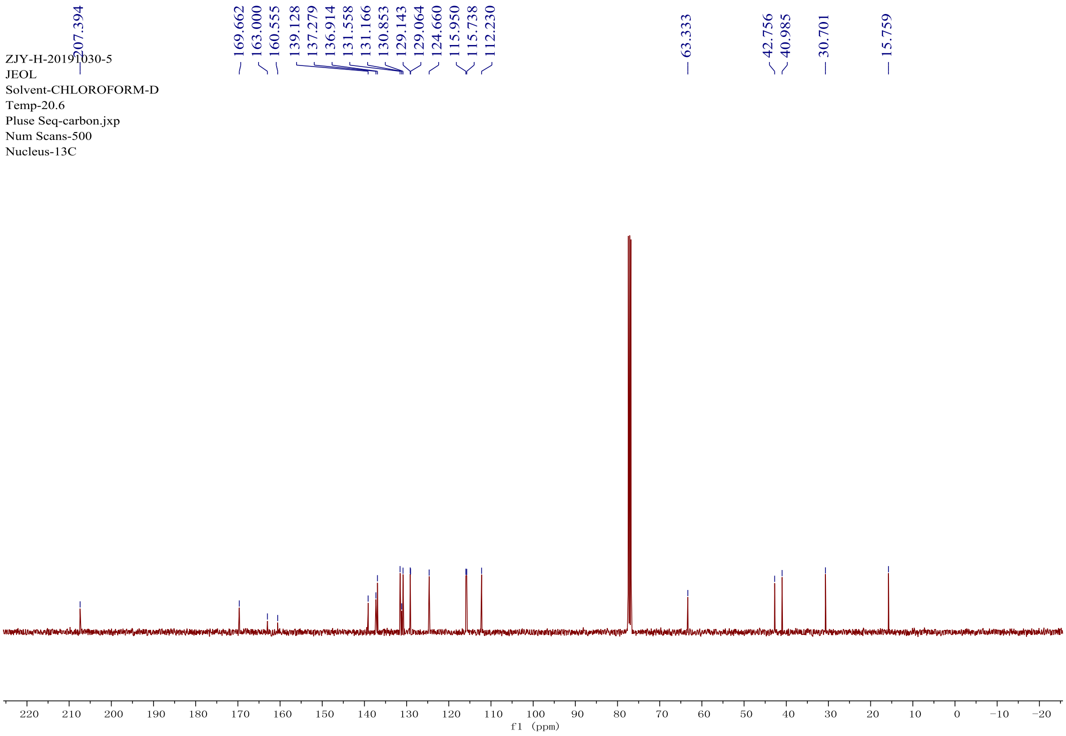

^{13}F NMR of compound **2b**

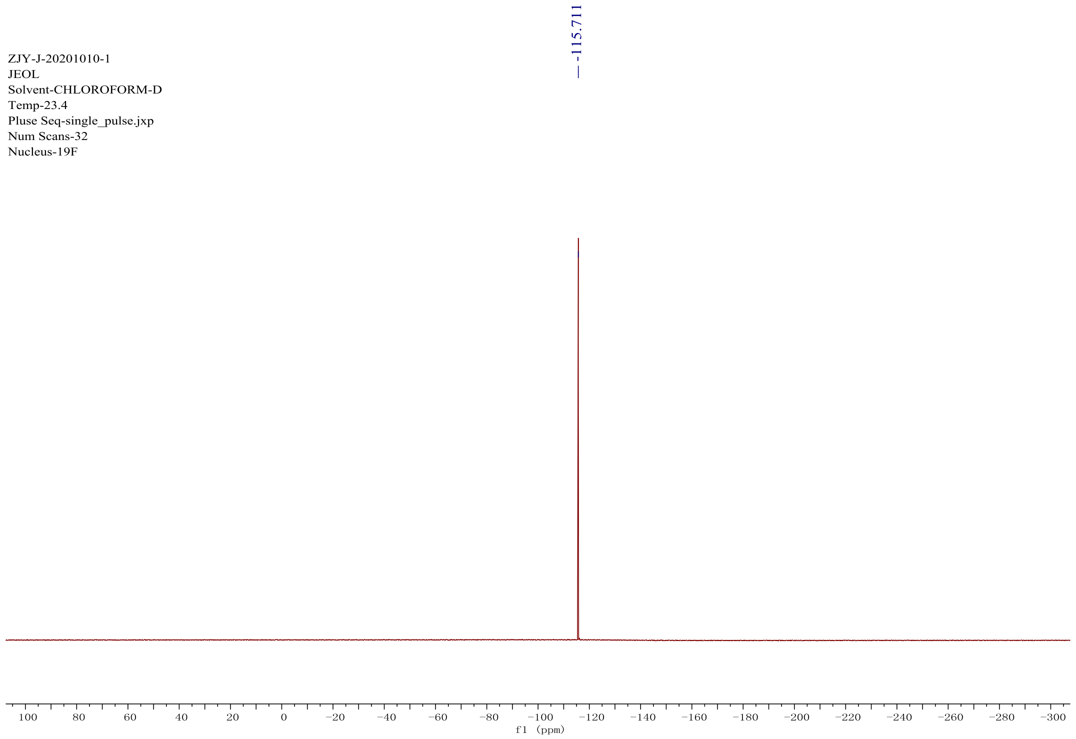

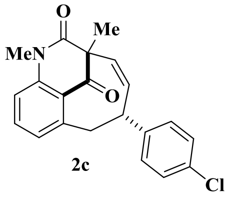

2c

^{1}H NMR of compound **2c**

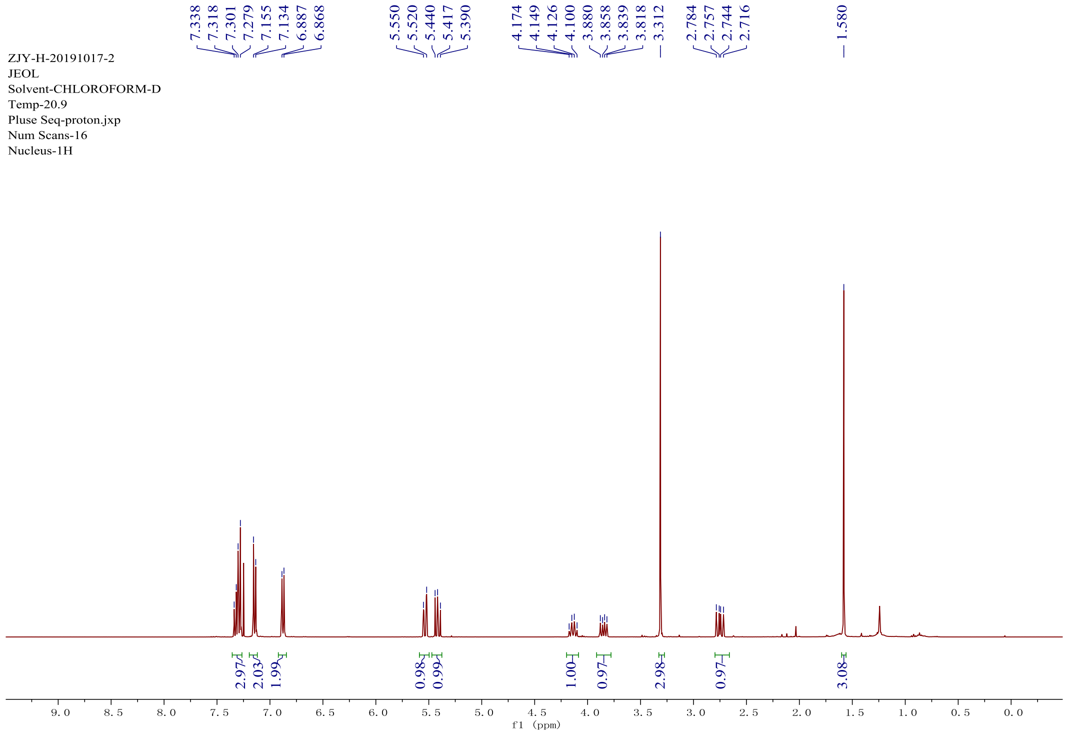

^{13}C NMR of compound **2c**

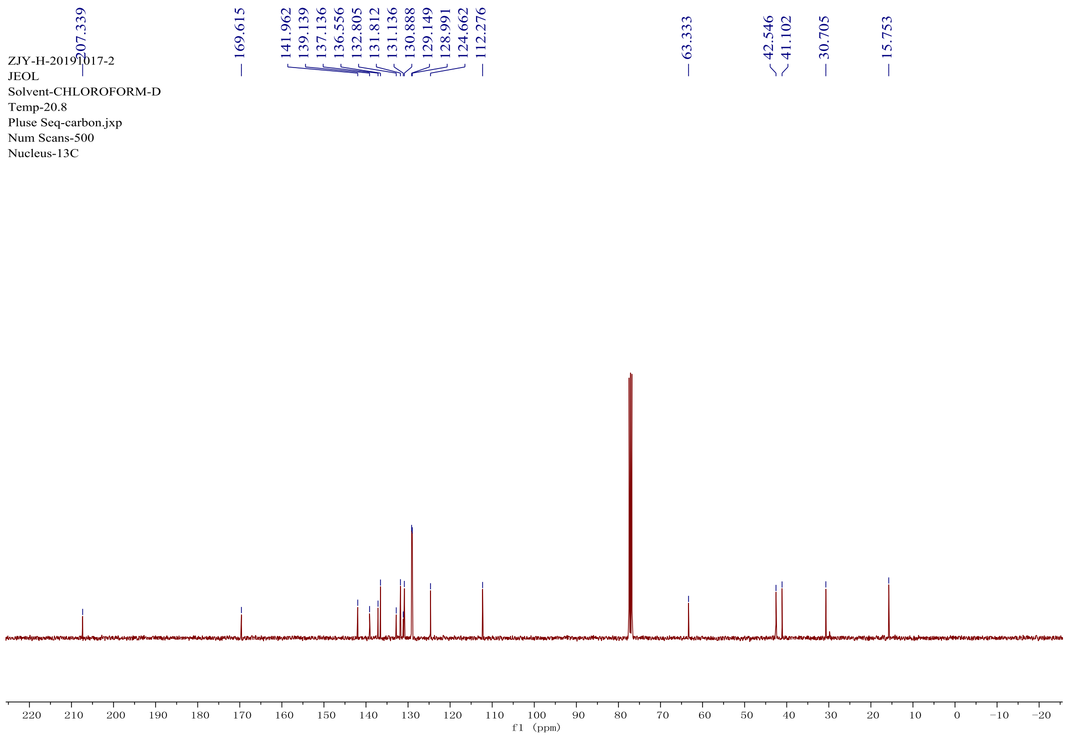

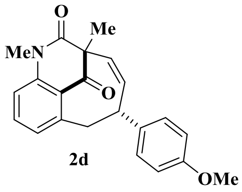

2d

^{1}H NMR of compound **2d**

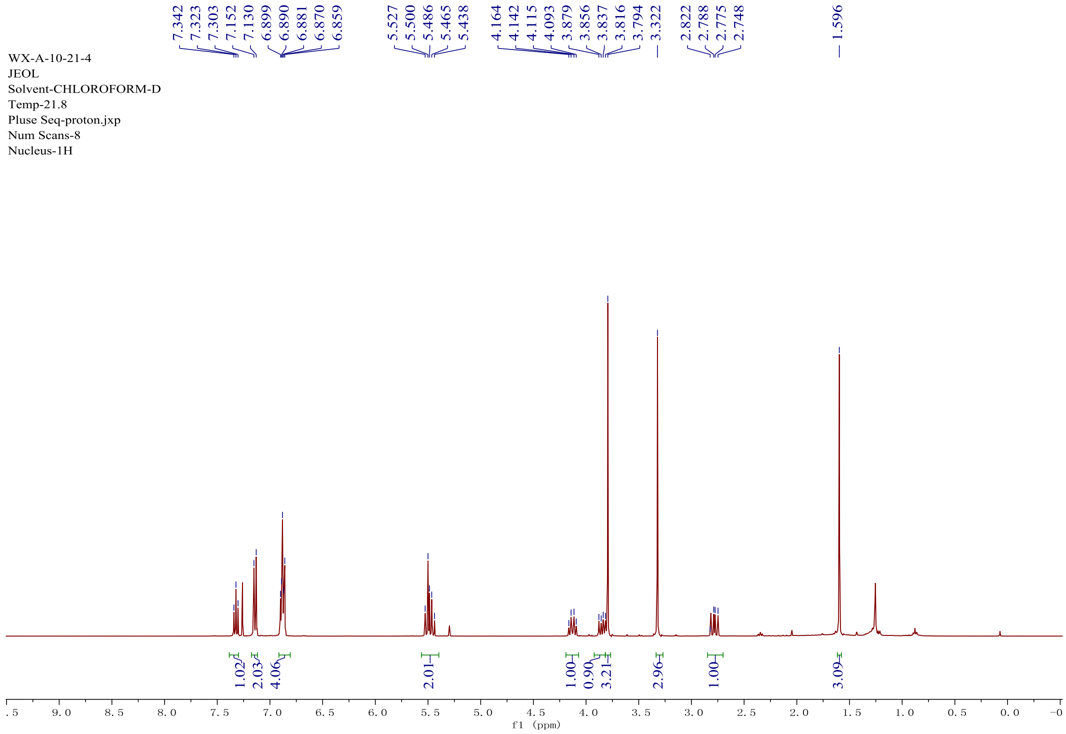

^{13}C NMR of compound **2d**

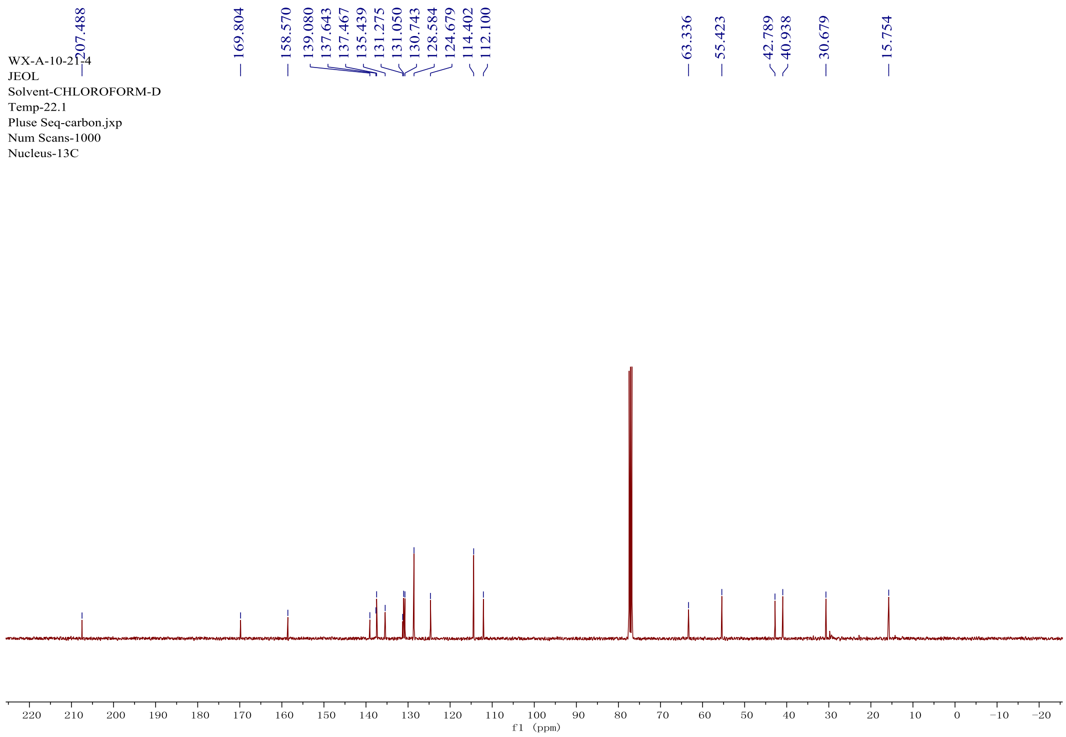

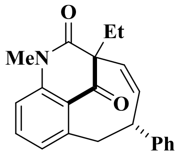

2e

^{1}H NMR of compound **2e**

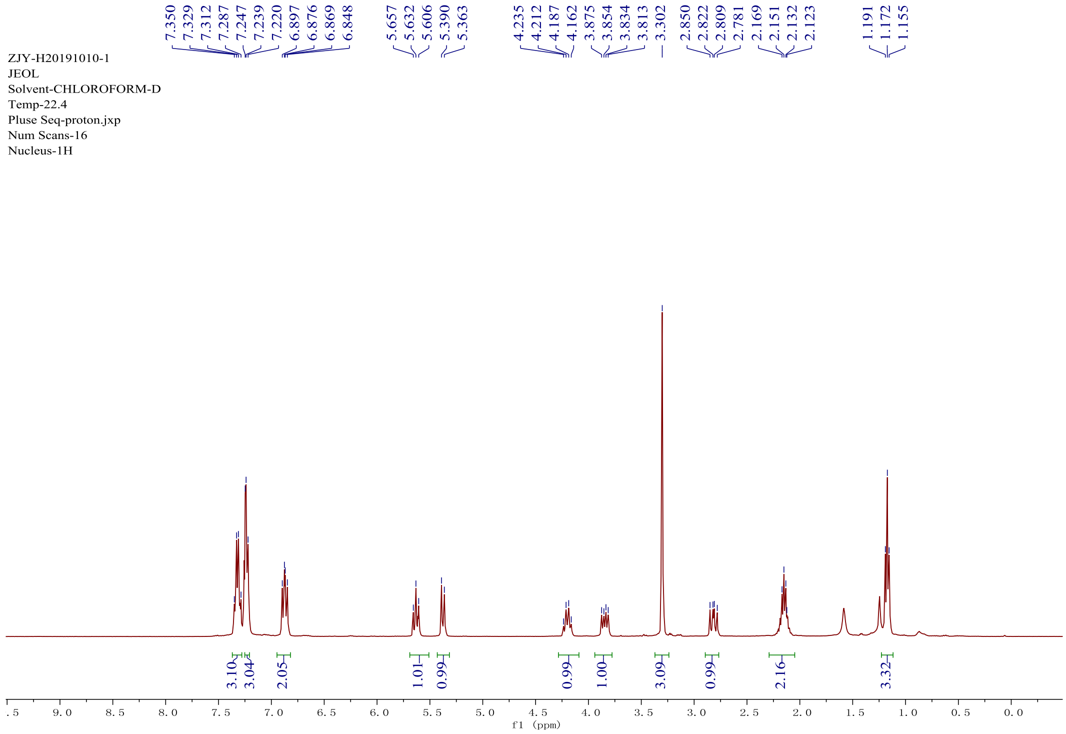

^{13}C NMR of compound **2e**

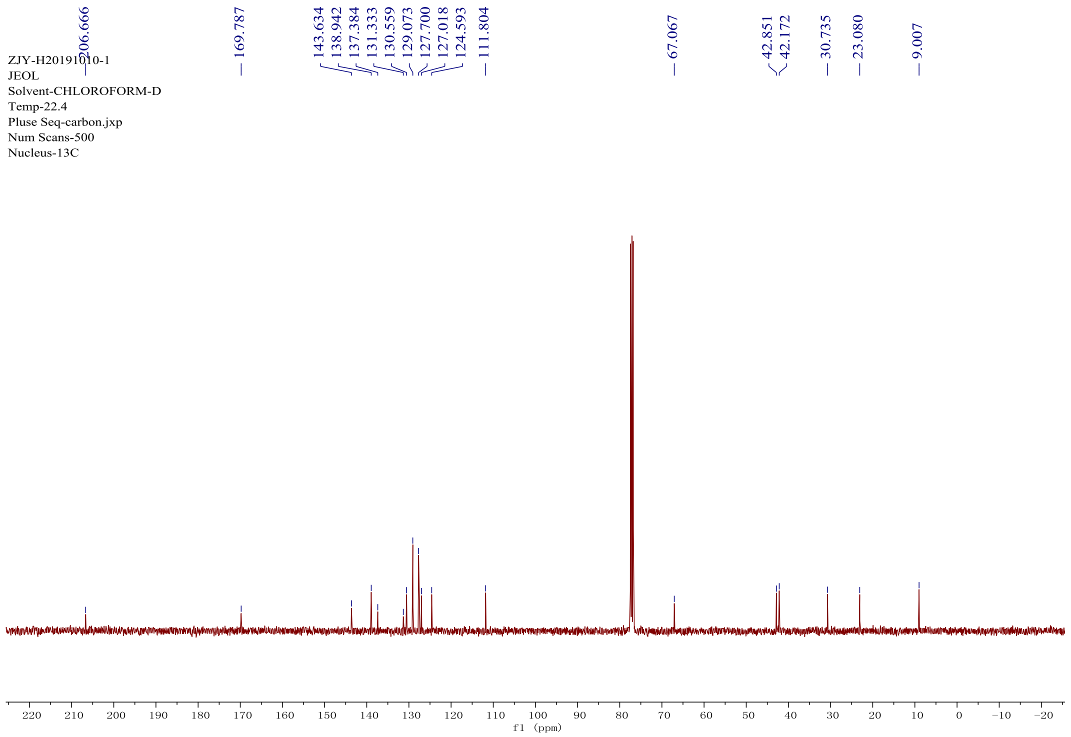

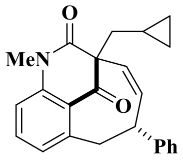

2f

^{1}H NMR of compound **2f**

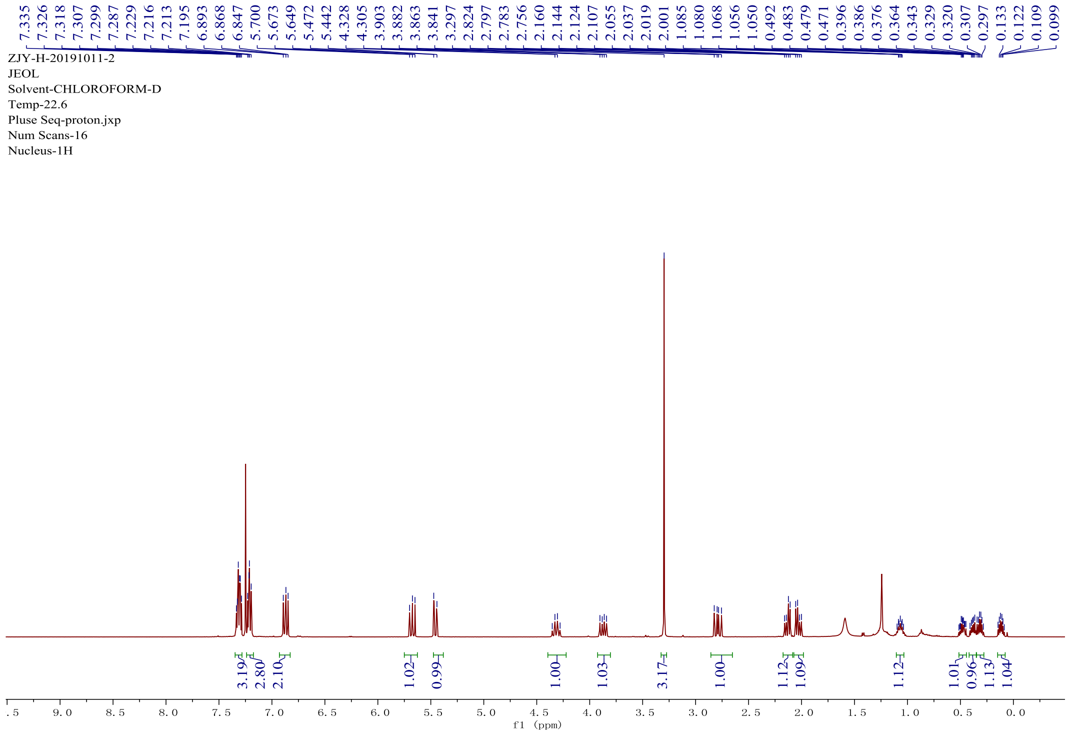

^{13}C NMR of compound **2f**

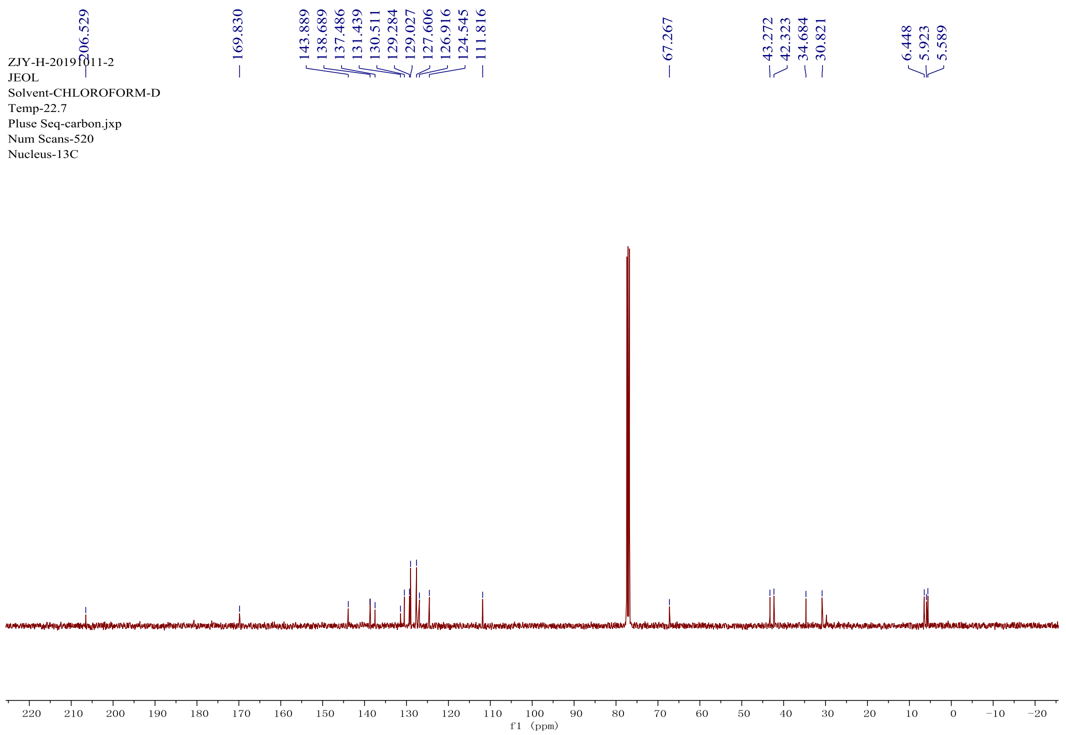

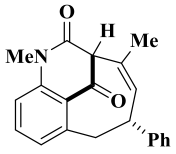

2g

^{1}H NMR of compound **2g**

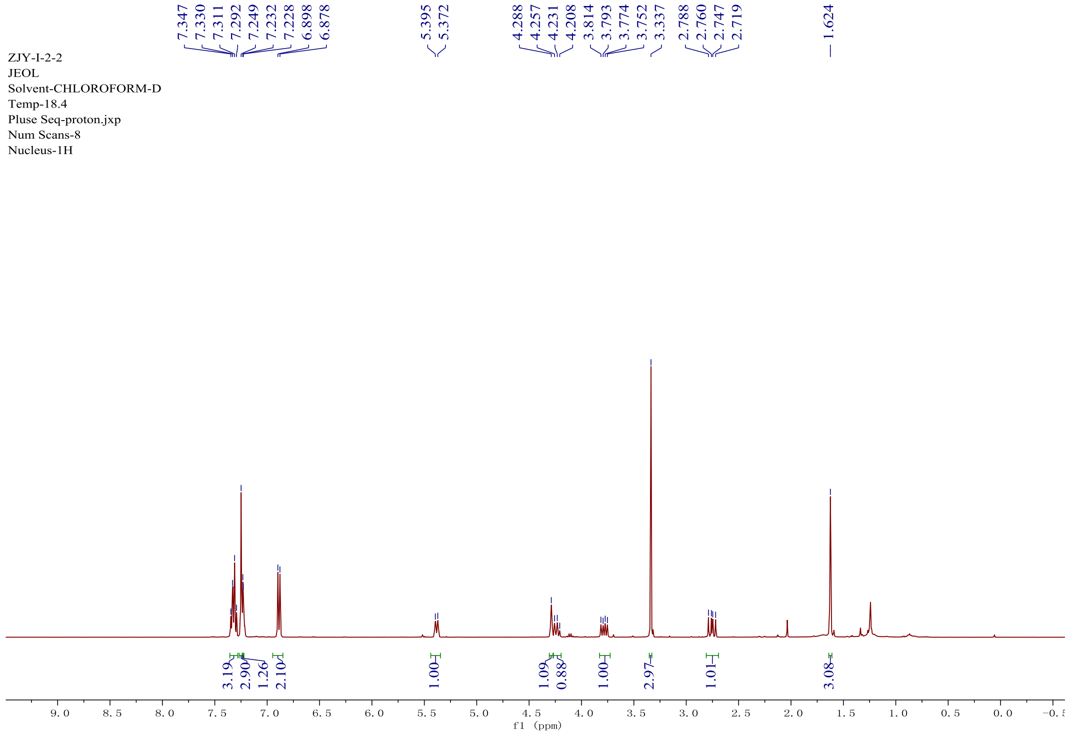

^{13}C NMR of compound **2g**

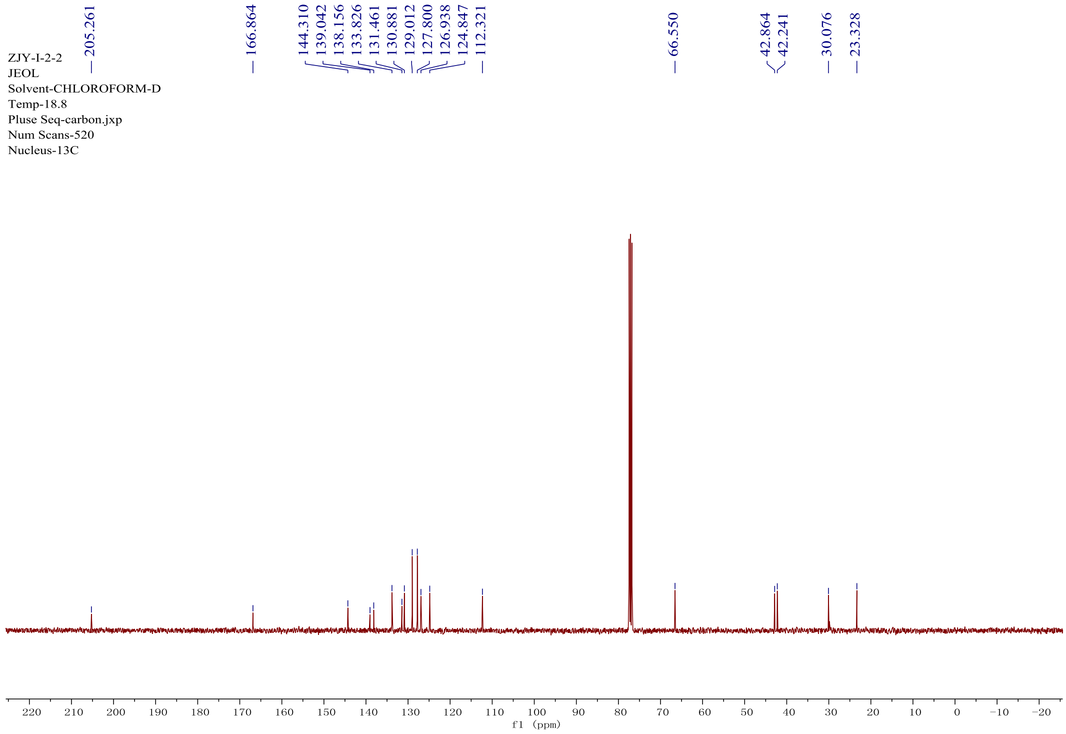

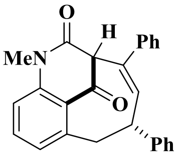

2h

^{1}H NMR of compound **2h**

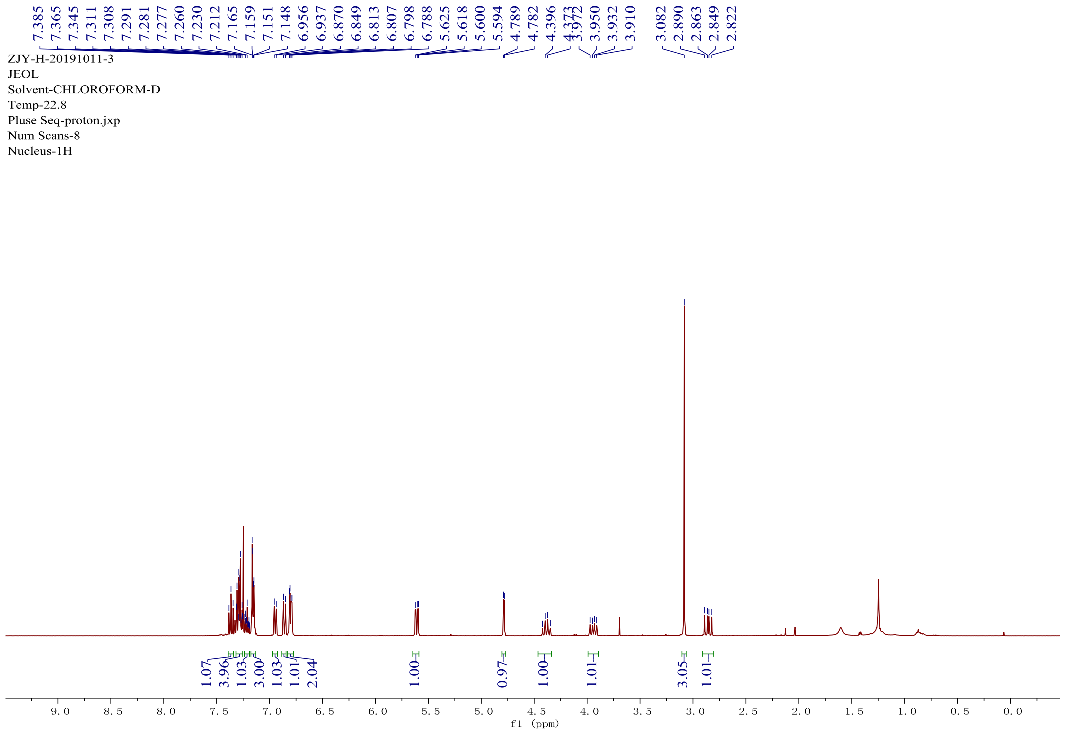

^{13}C NMR of compound **2h**

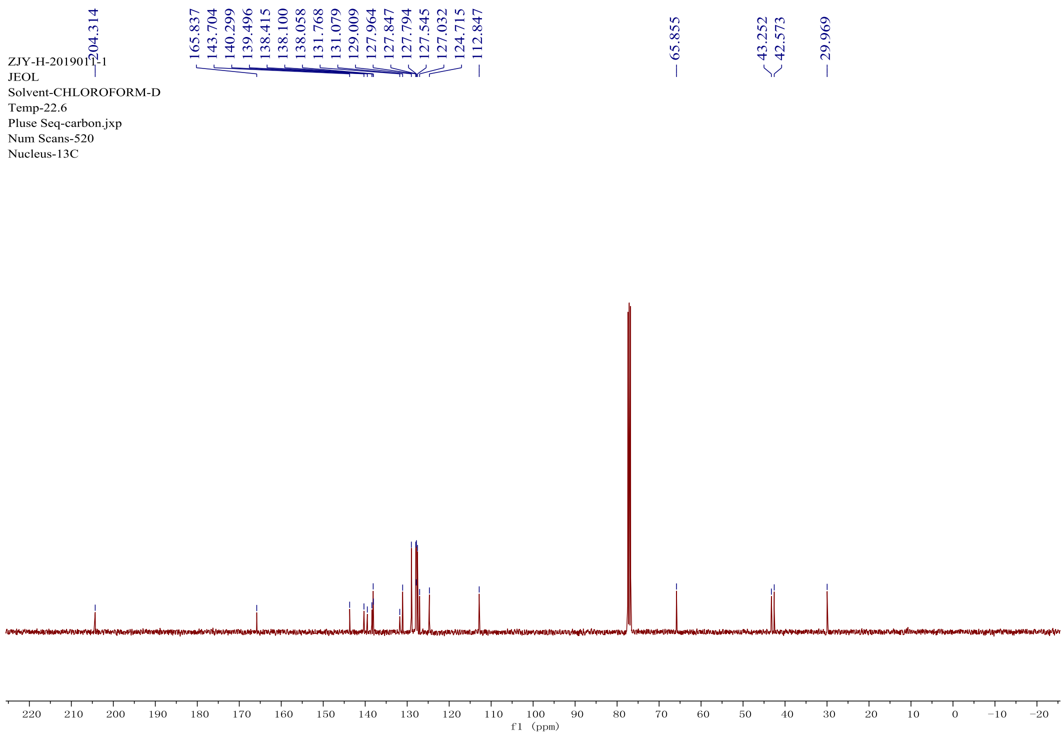

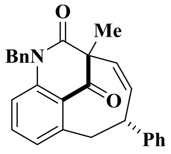

2i

^{1}H NMR of compound **2i**

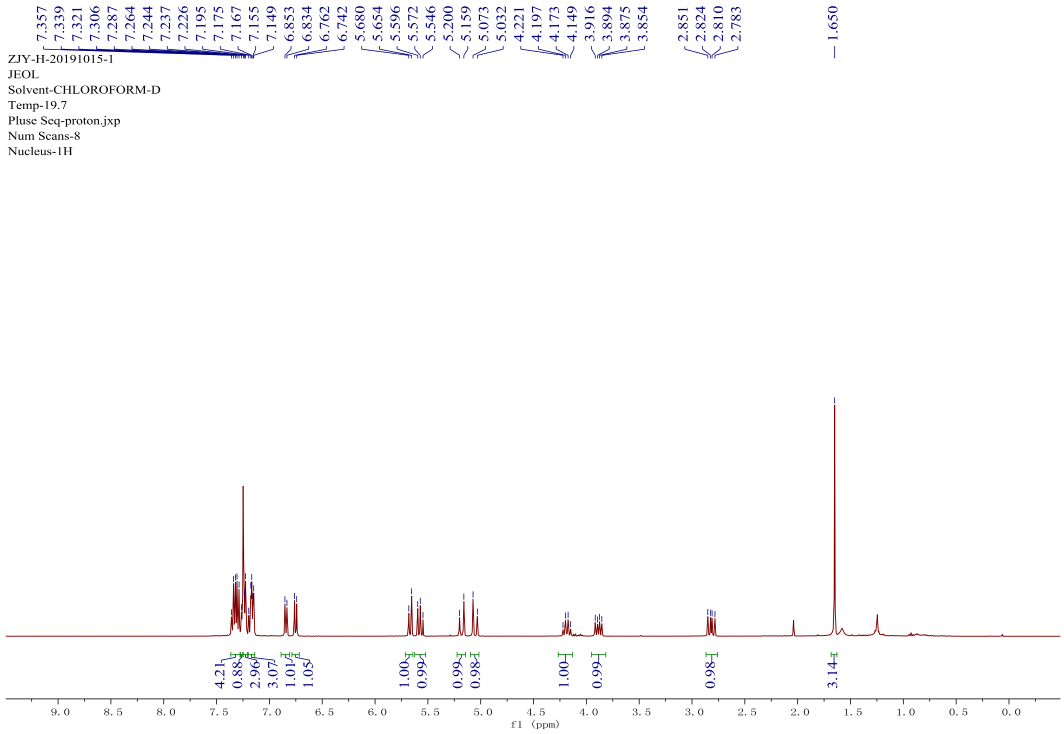

^{13}C NMR of compound **2i**

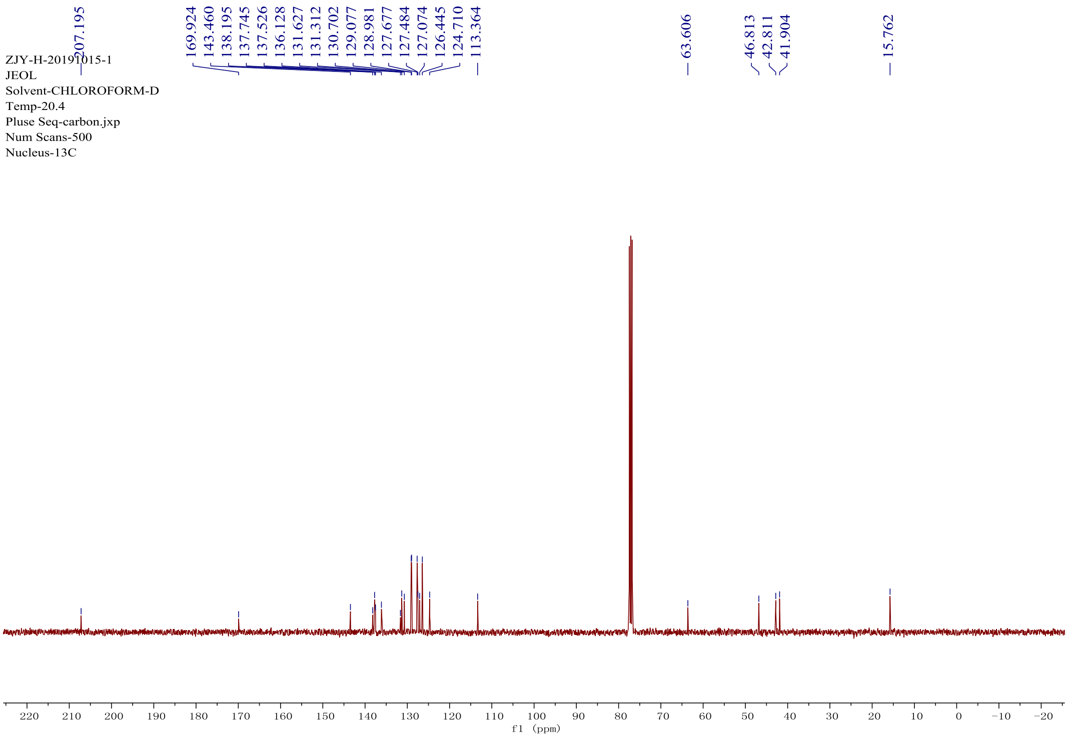

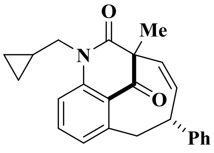

2j

^{1}H NMR of compound **2j**

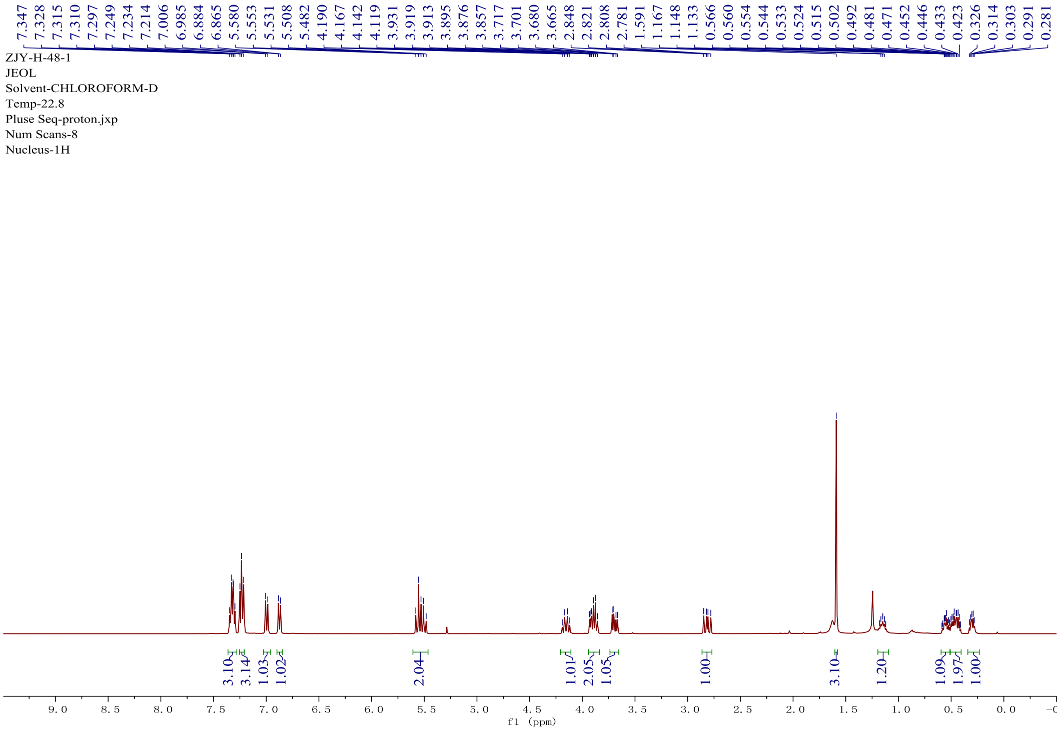

^{13}C NMR of compound **2j**

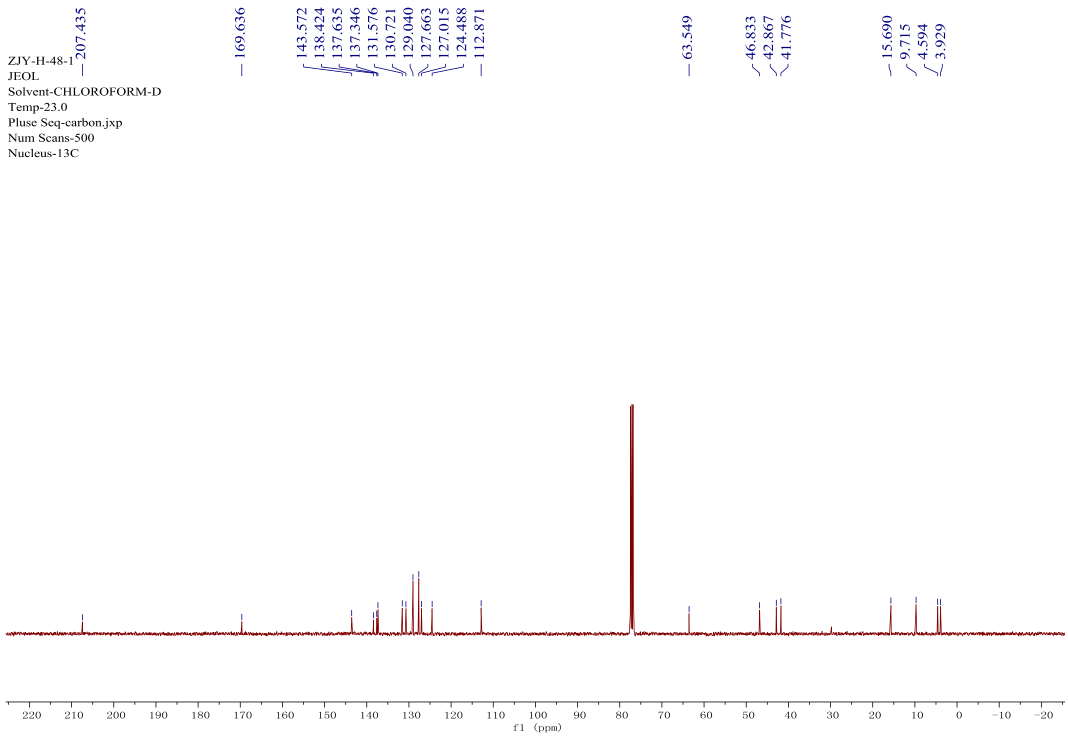

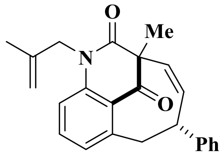

2k

^{1}H NMR of compound **2k**

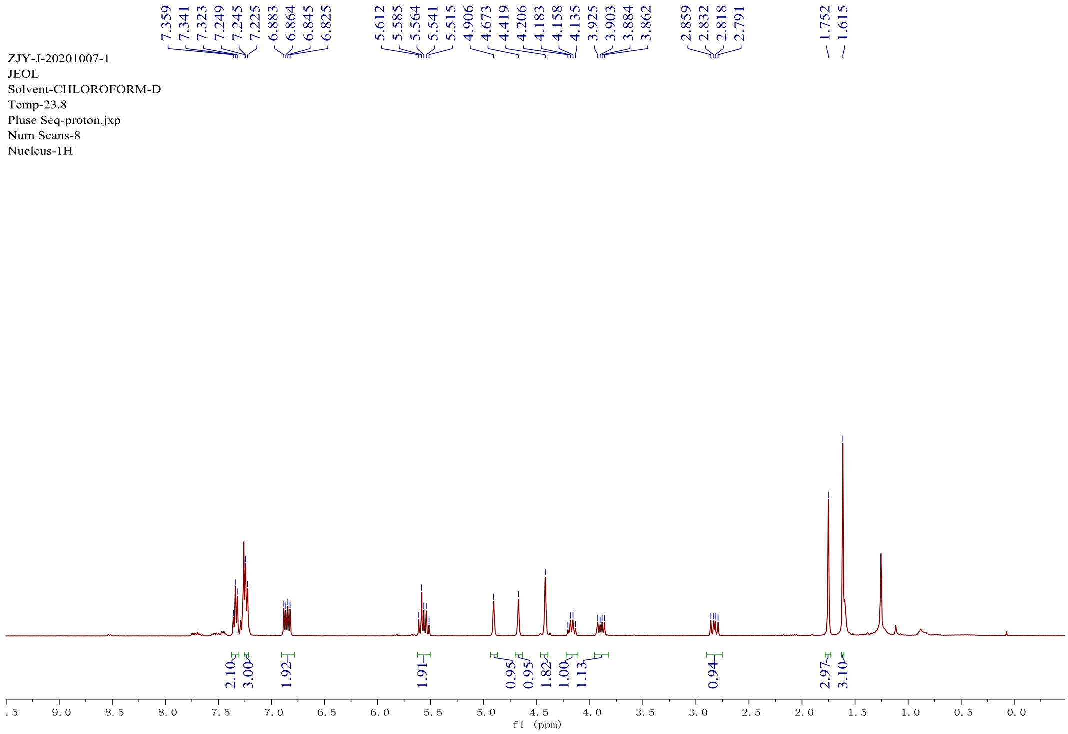

^{13}C NMR of compound **2k**

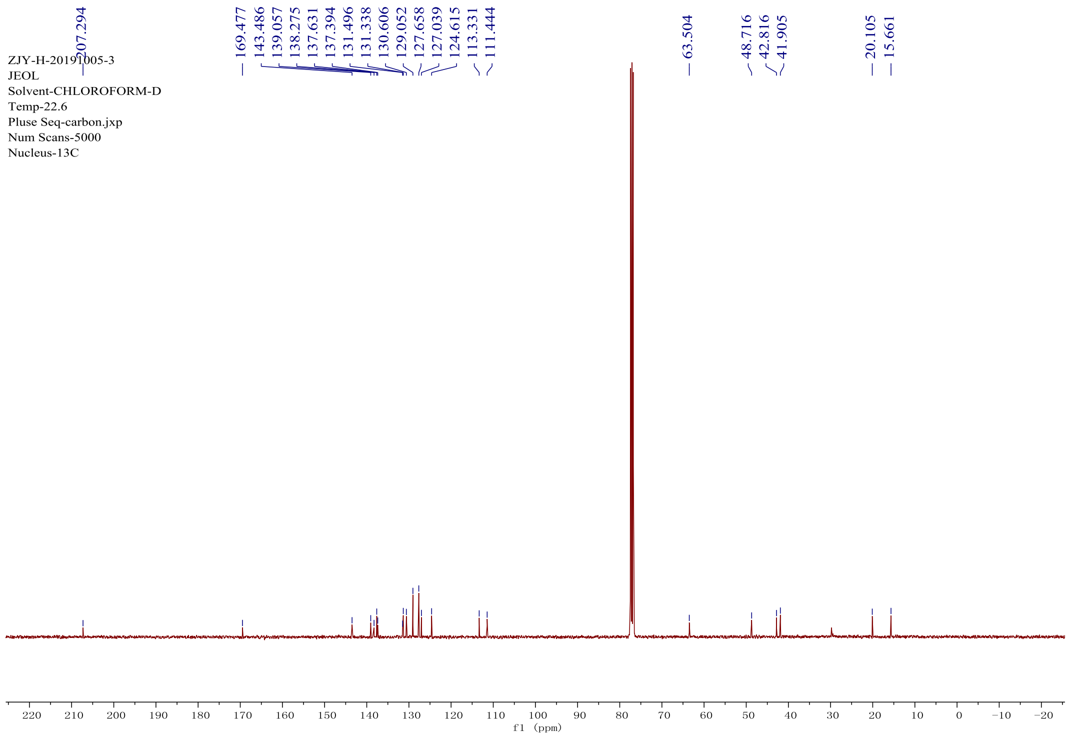

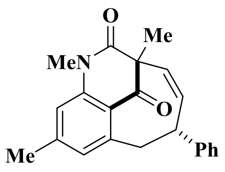

2l

^{1}H NMR of compound **2l**

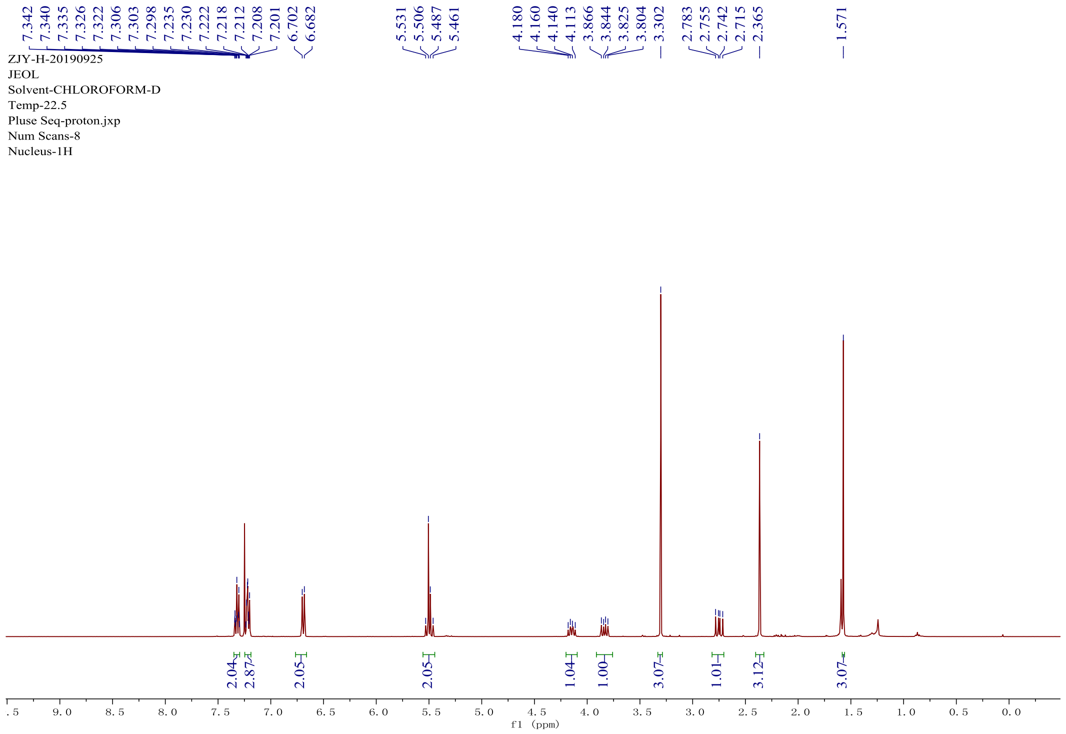

^{13}C NMR of compound **2l**

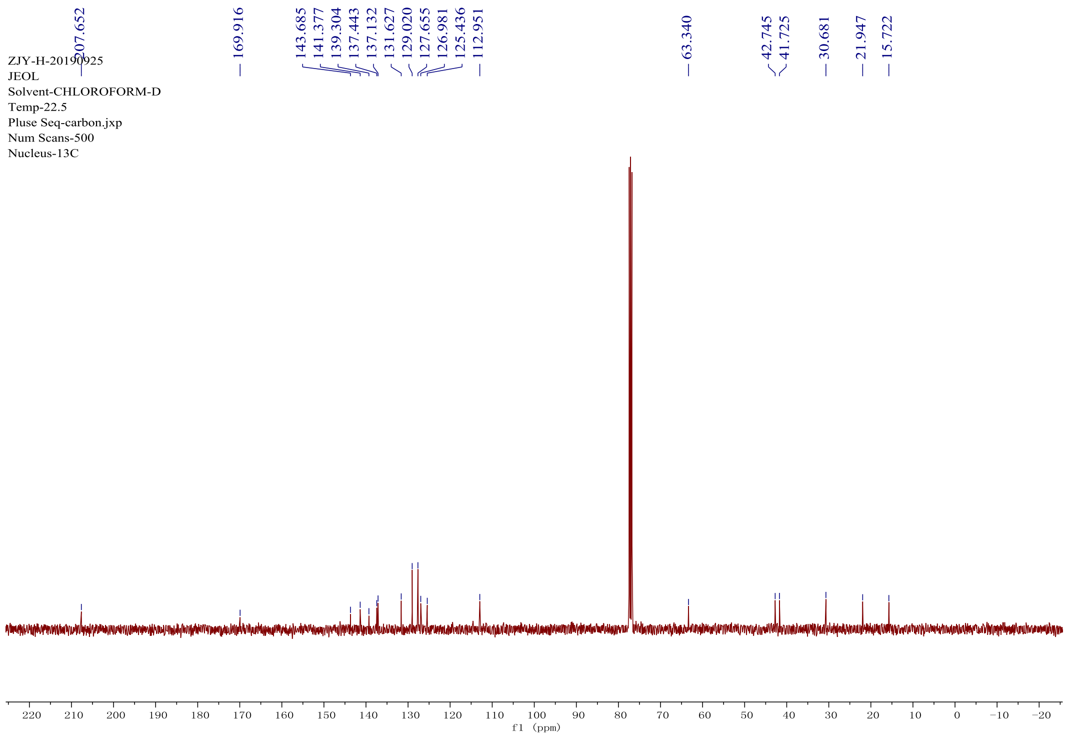

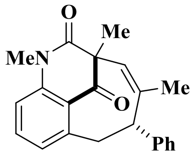

2m

^{1}H NMR of compound **2m**

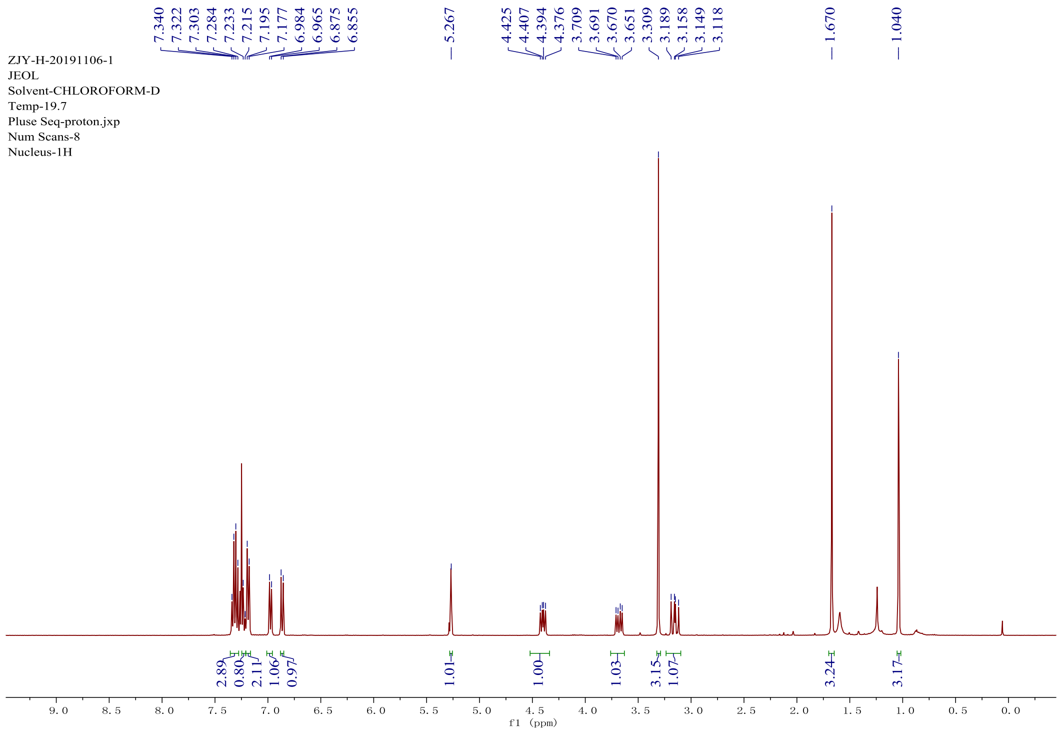

^{13}C NMR of compound **2m**

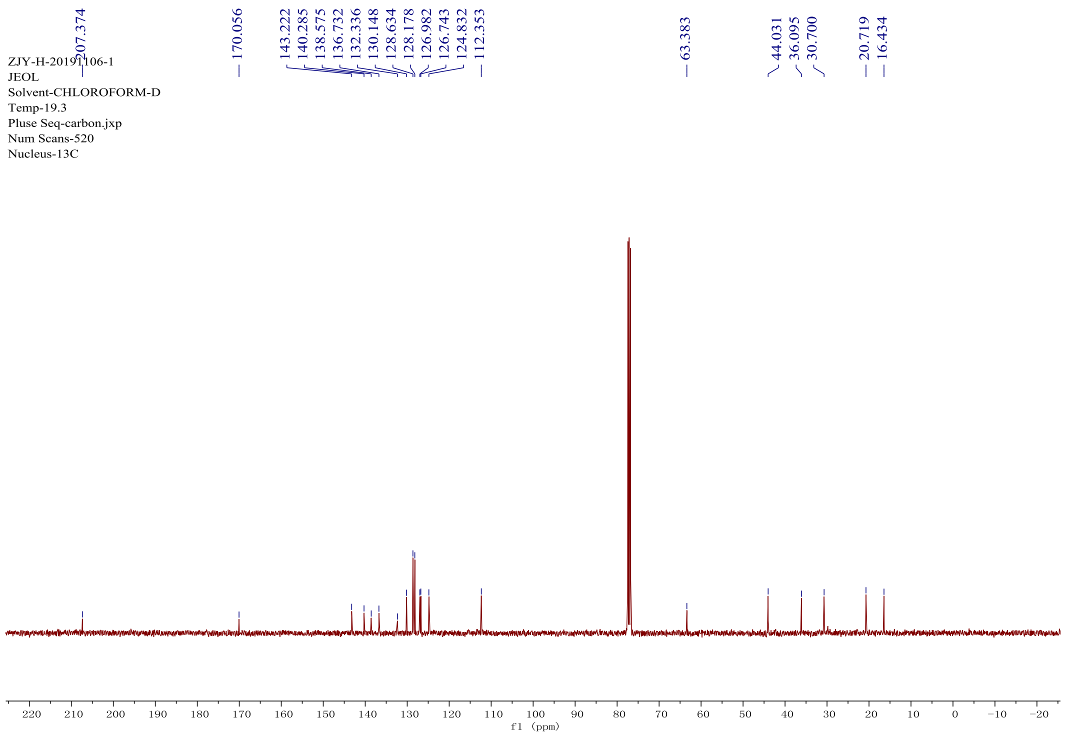

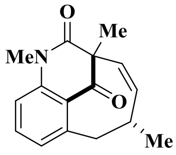

2n

^{1}H NMR of compound **2n**

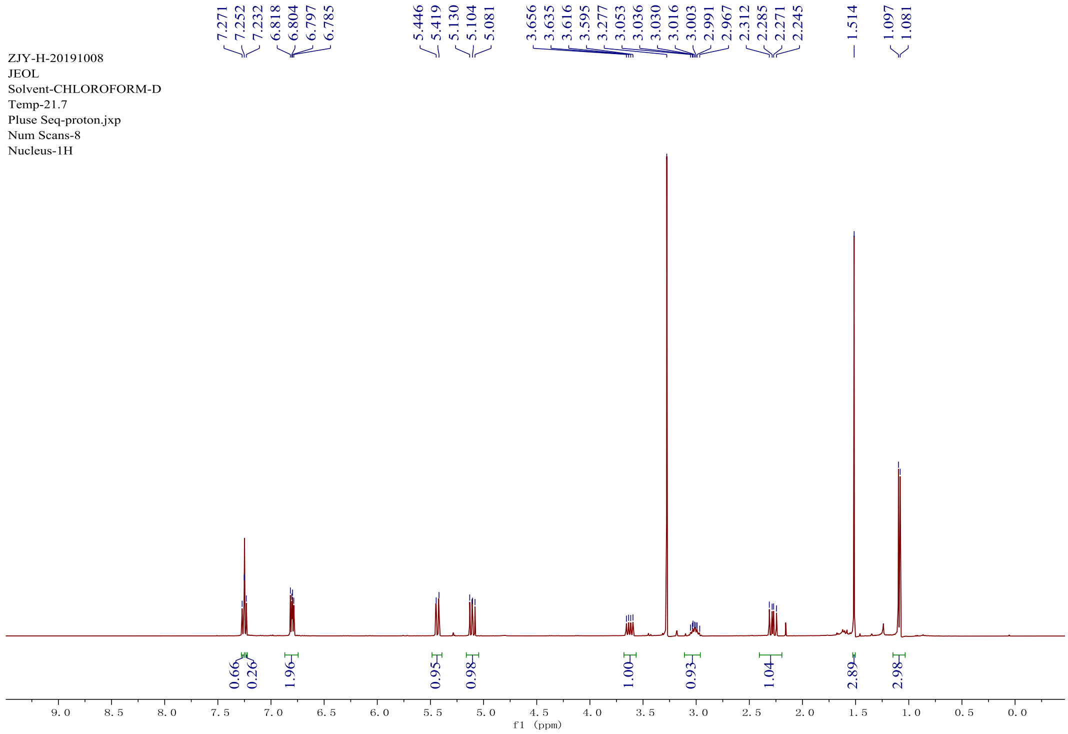

^{13}C NMR of compound **2n**

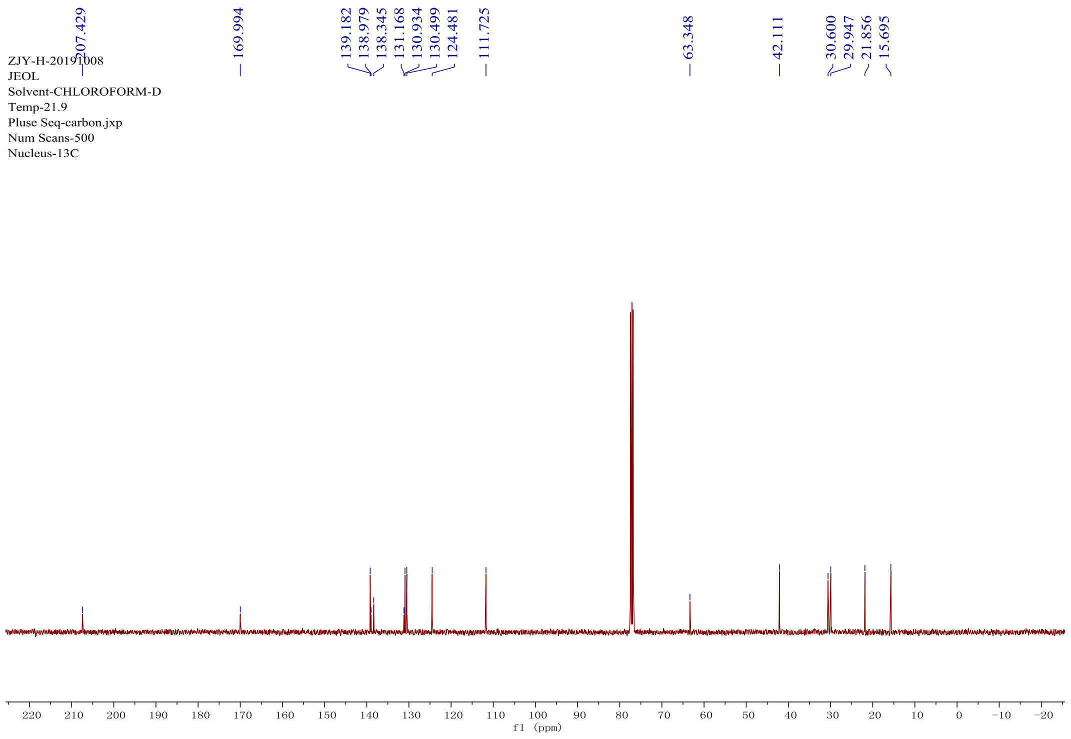

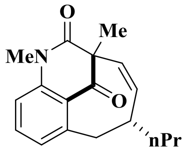

2o

^{1}H NMR of compound **2o**

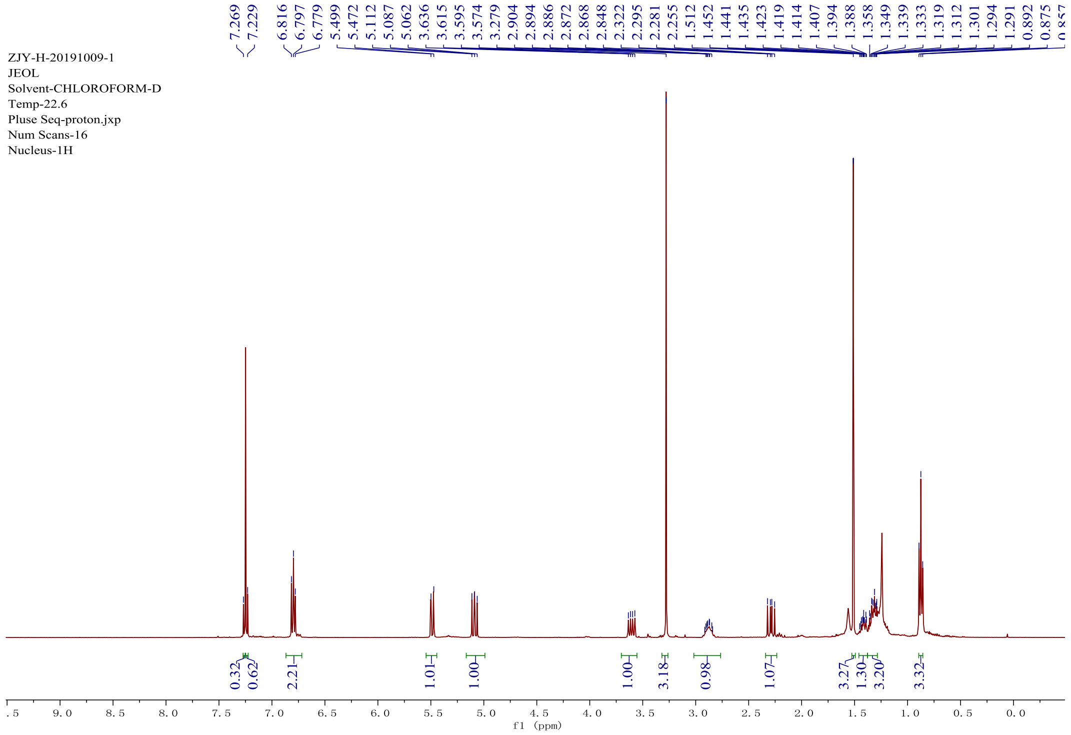

^{13}C NMR of compound **2o**

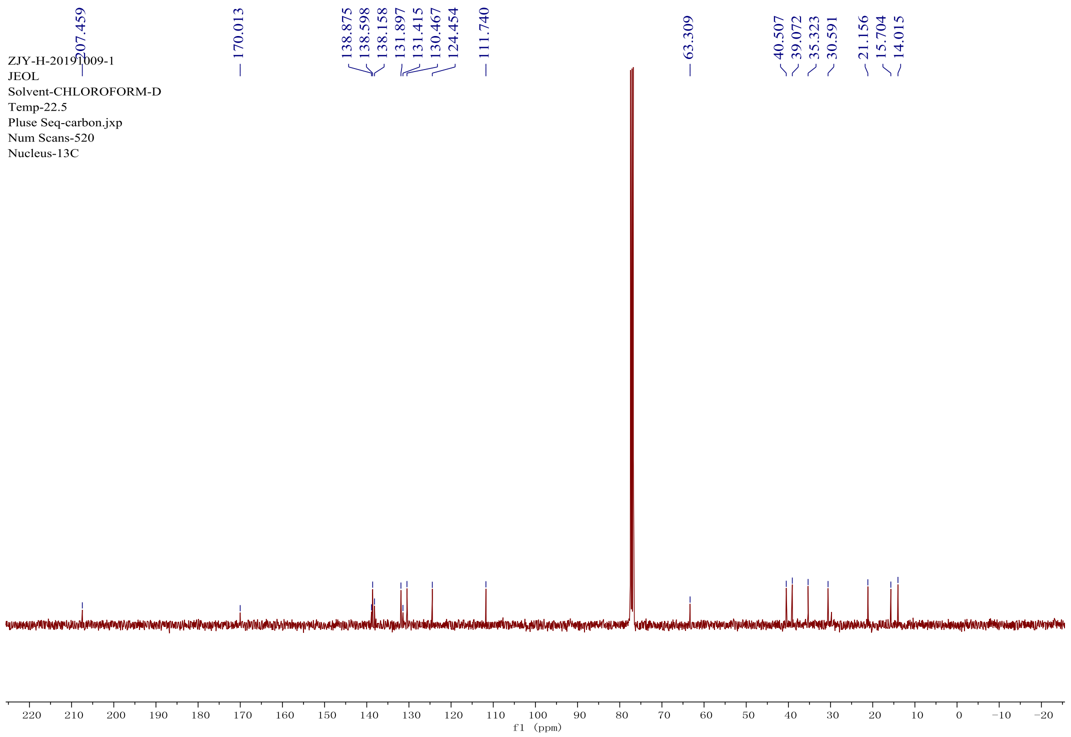

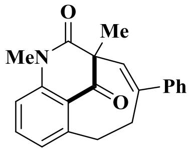

2p

^{1}H NMR of compound **2p**

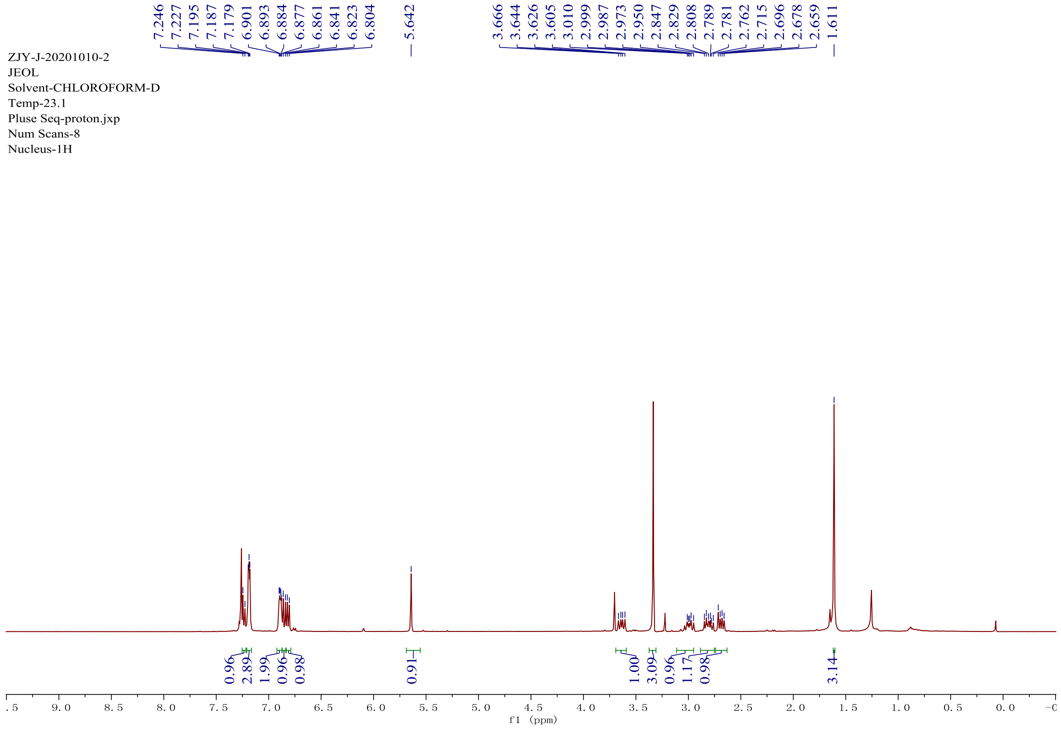

^{13}C NMR of compound **2p**

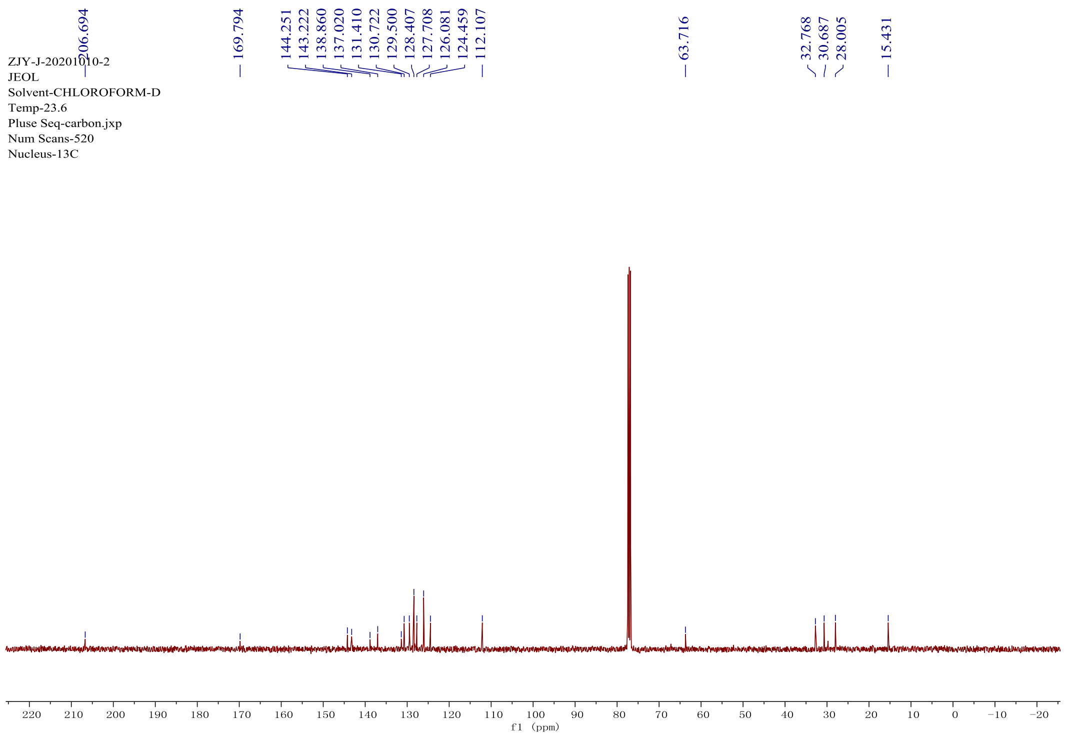

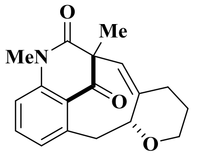

2q

^{1}H NMR of compound **2q**

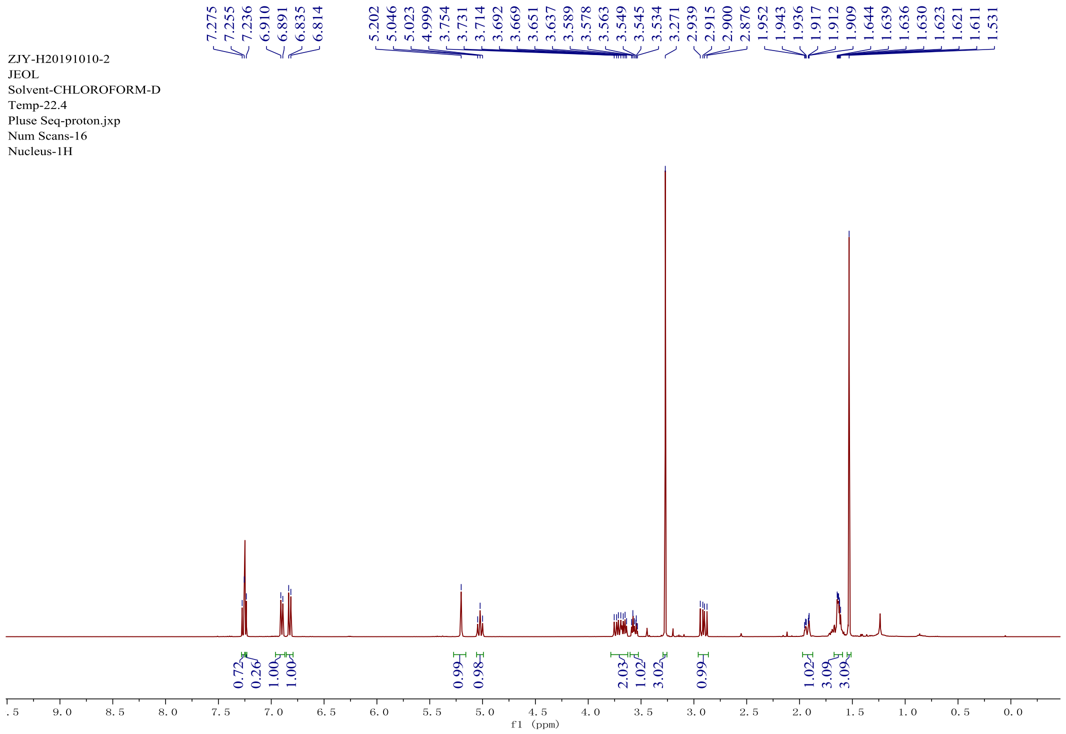

^{13}C NMR of compound **2q**

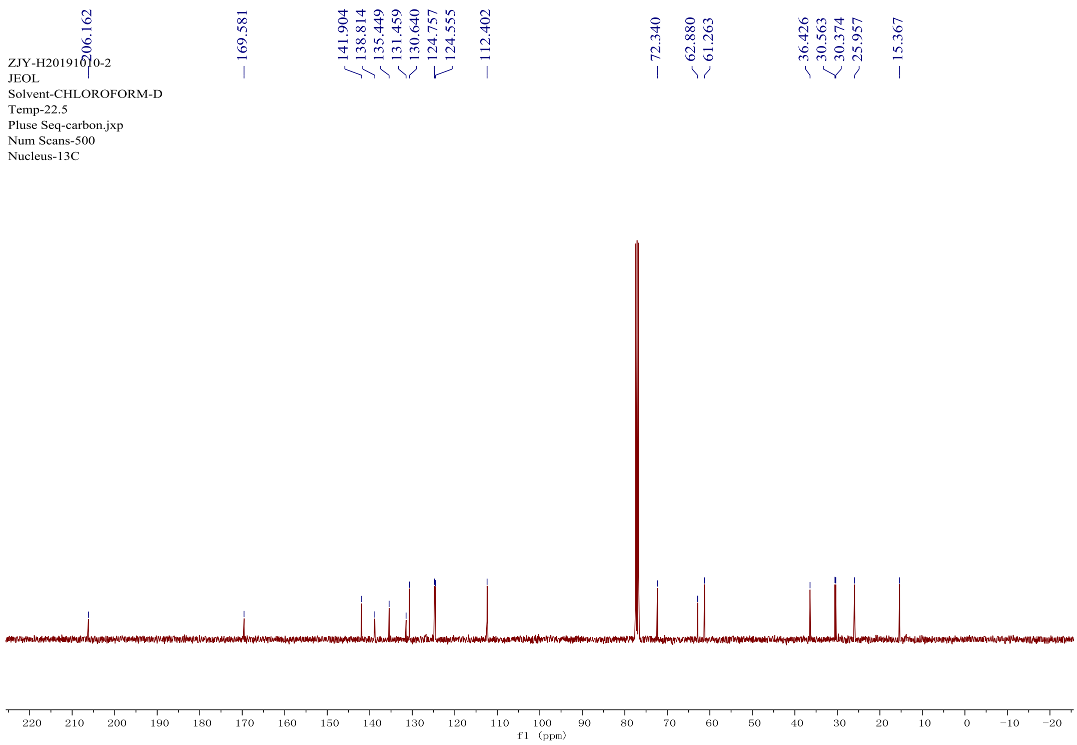

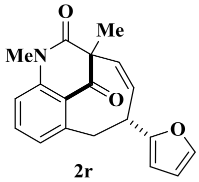

2r

^{1}H NMR of compound **2r**

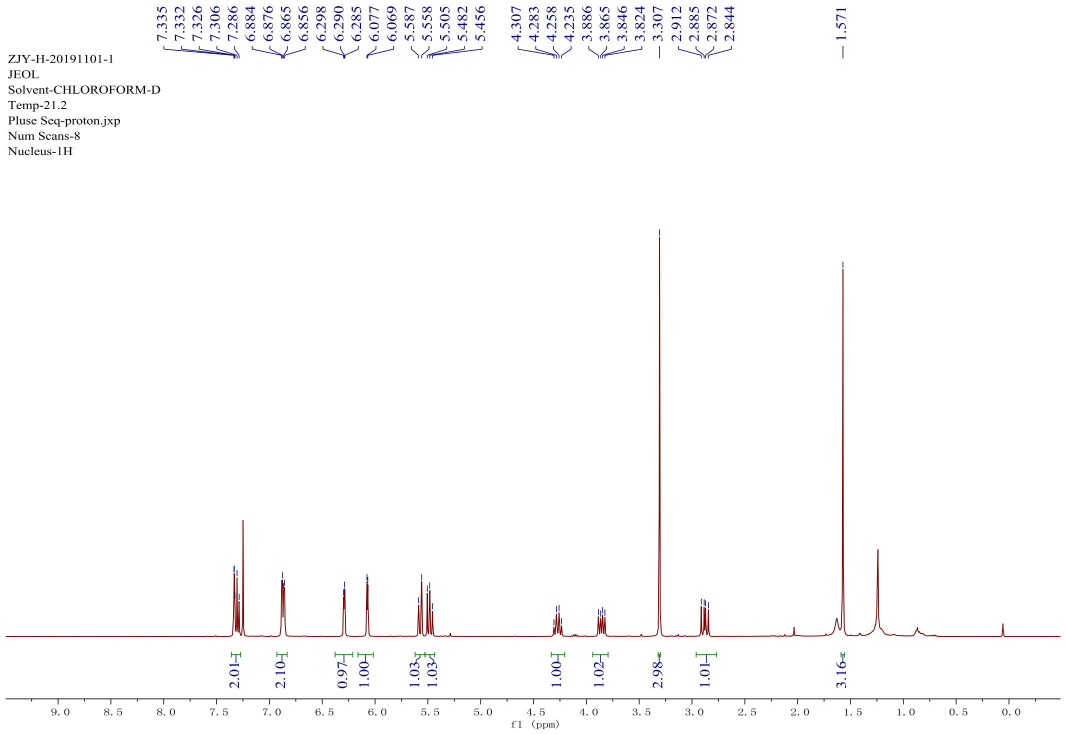

^{13}C NMR of compound **2r**

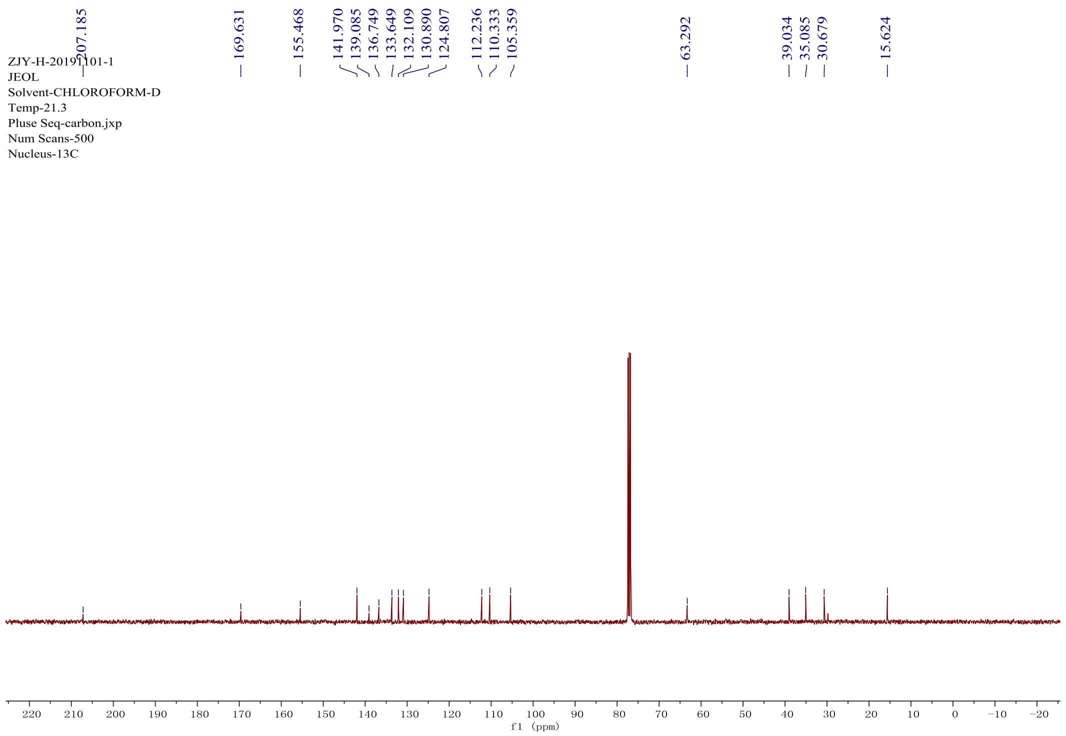

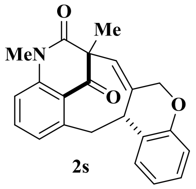

^{1}H NMR of compound **2s**

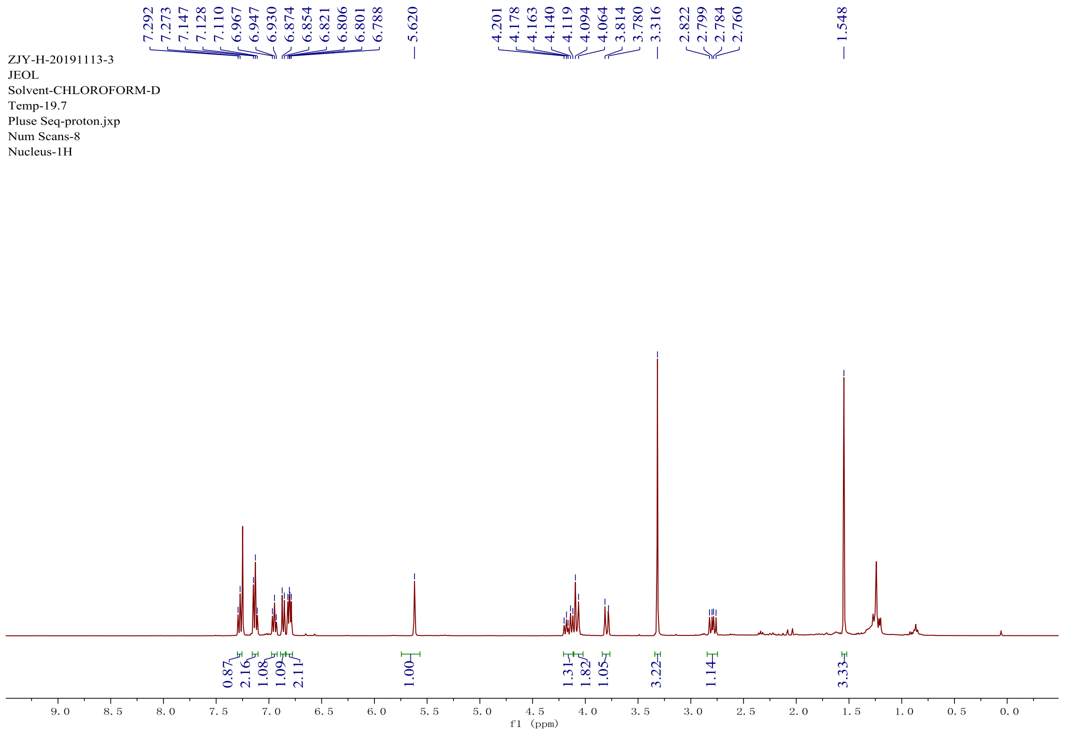

^{13}C NMR of compound **2s**

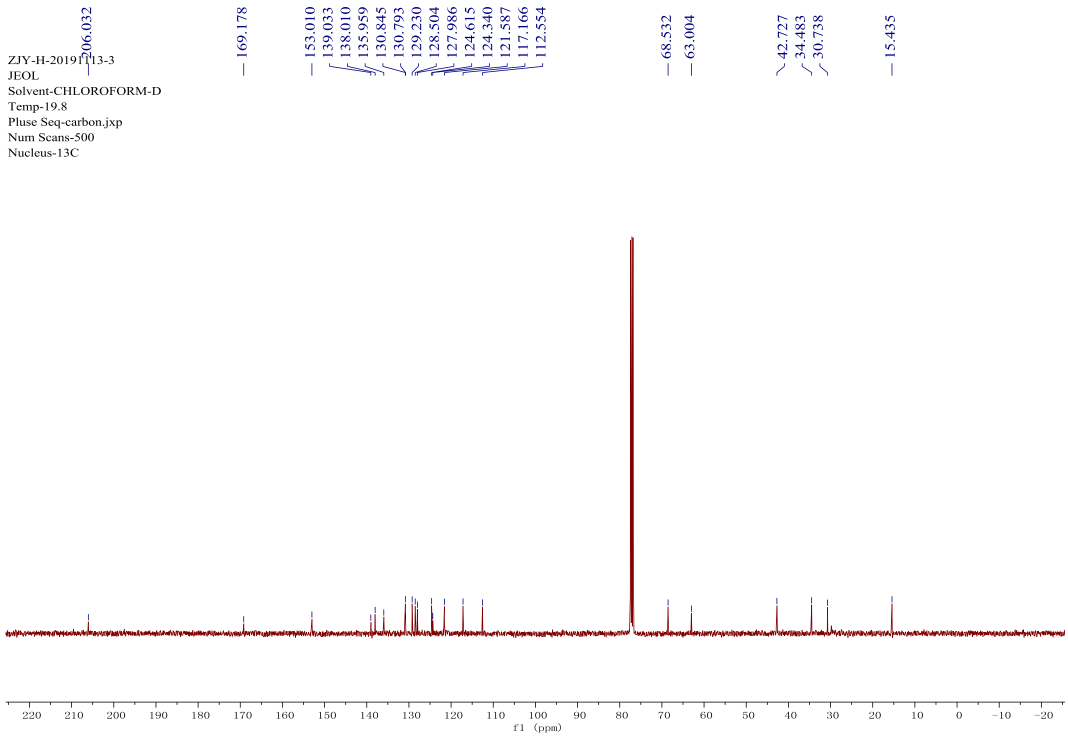

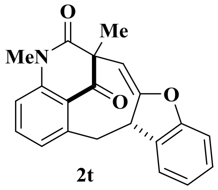

^{1}H NMR of compound **2t**

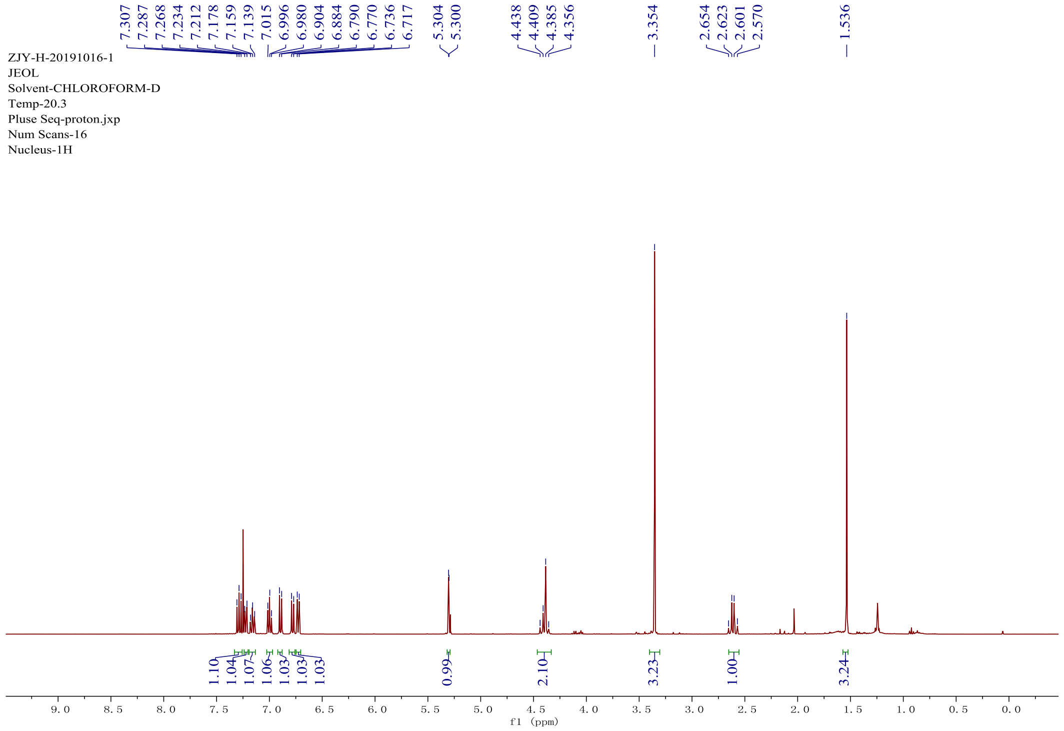

^{13}C NMR of compound **2t**

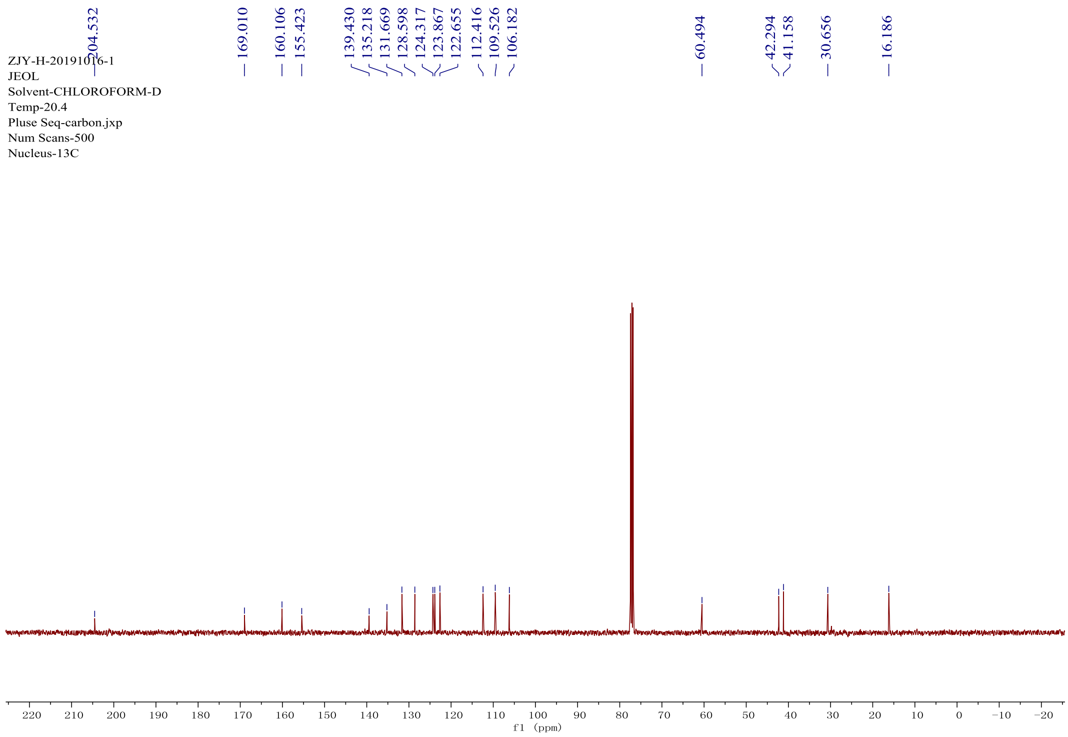

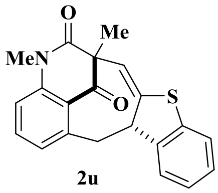

2u

^{1}H NMR of compound **2u**

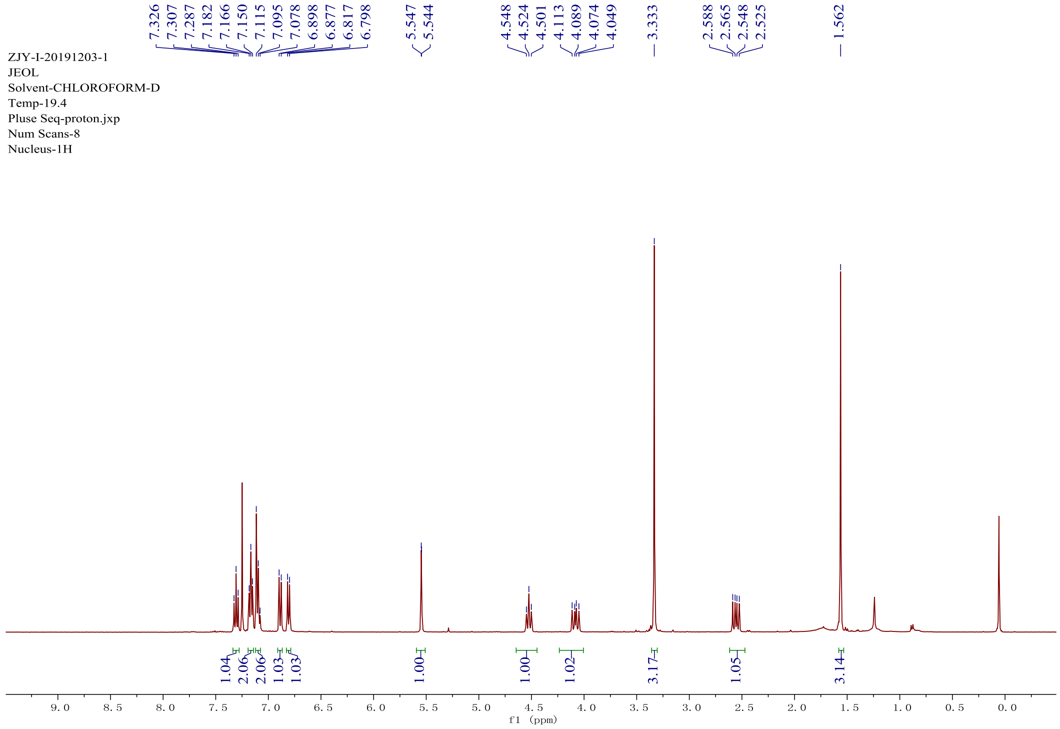

^{13}C NMR of compound **2u**

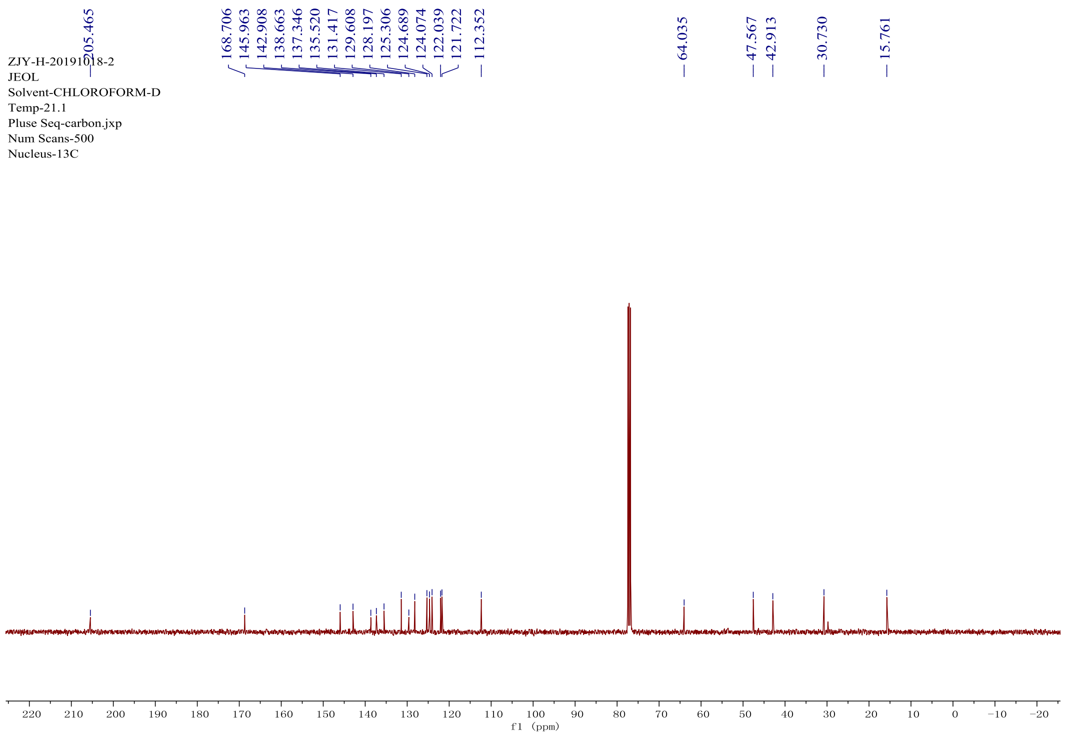

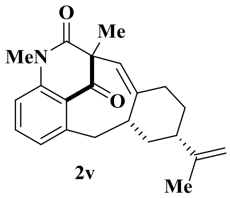

2v

^{1}H NMR of compound **2v**

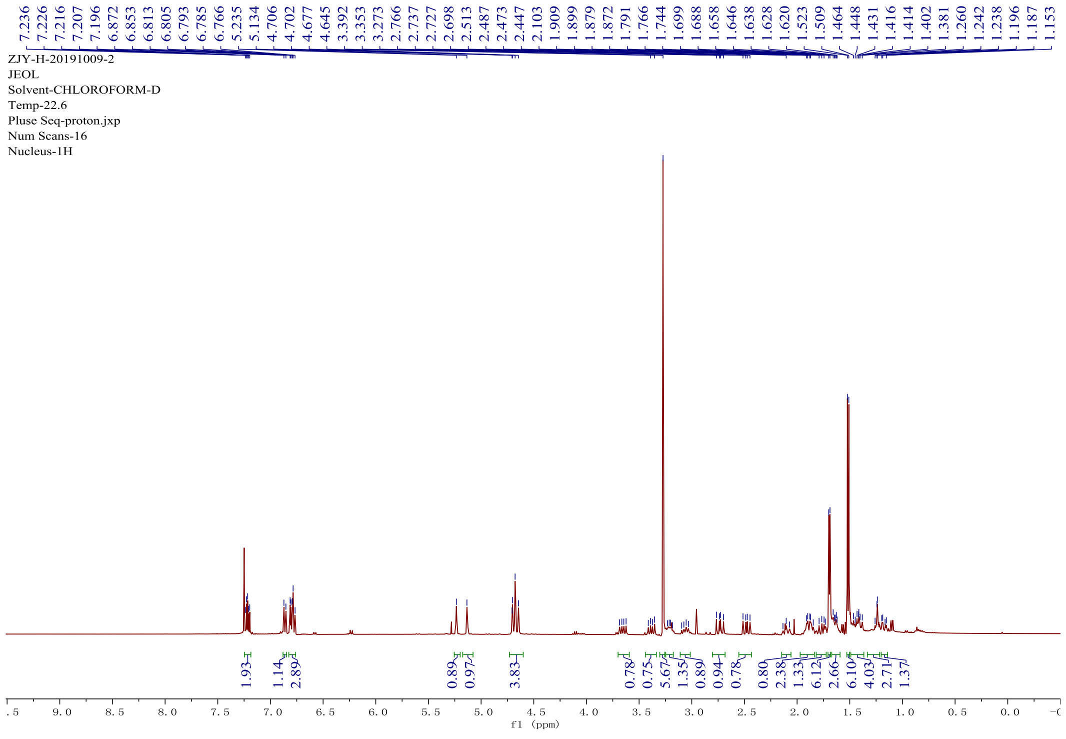

^{13}C NMR of compound **2v**

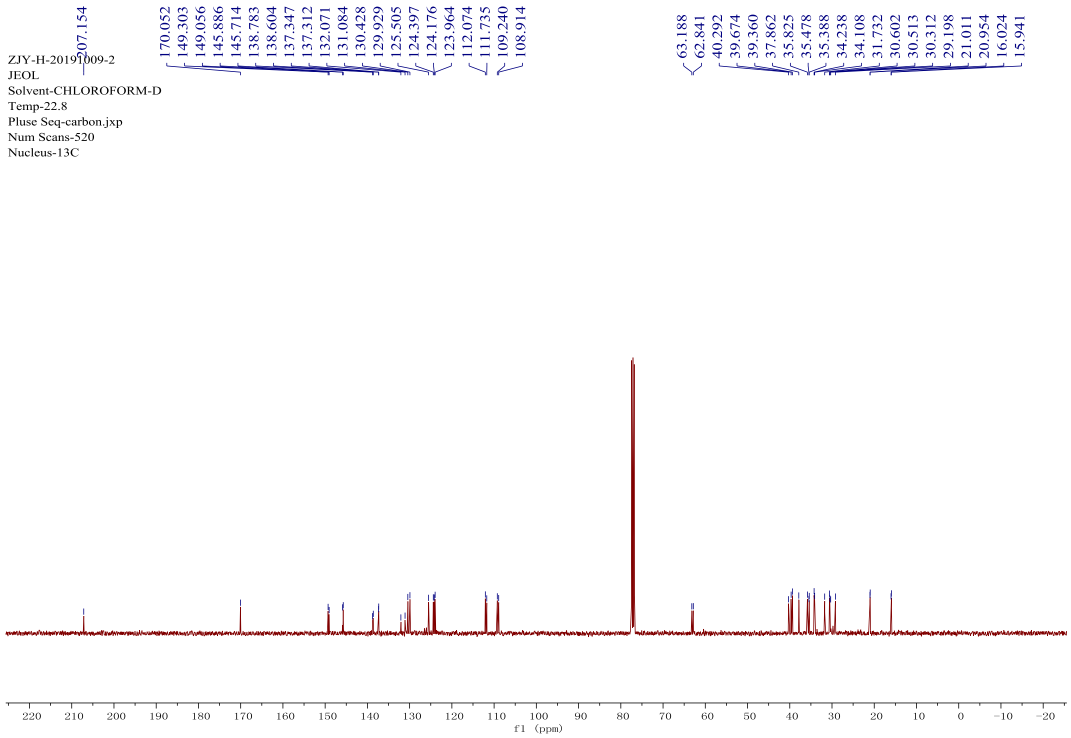

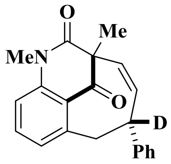

2a-D

^{1}H NMR of compound **2a-D**

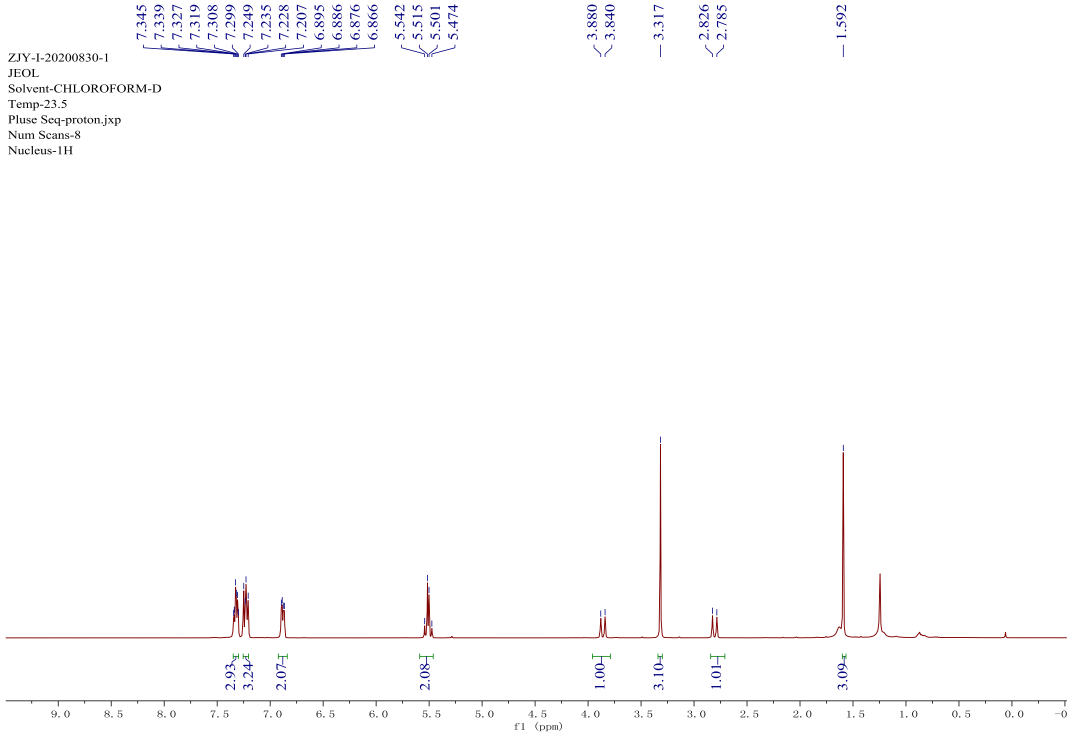

^{13}C NMR of compound **2a-D**

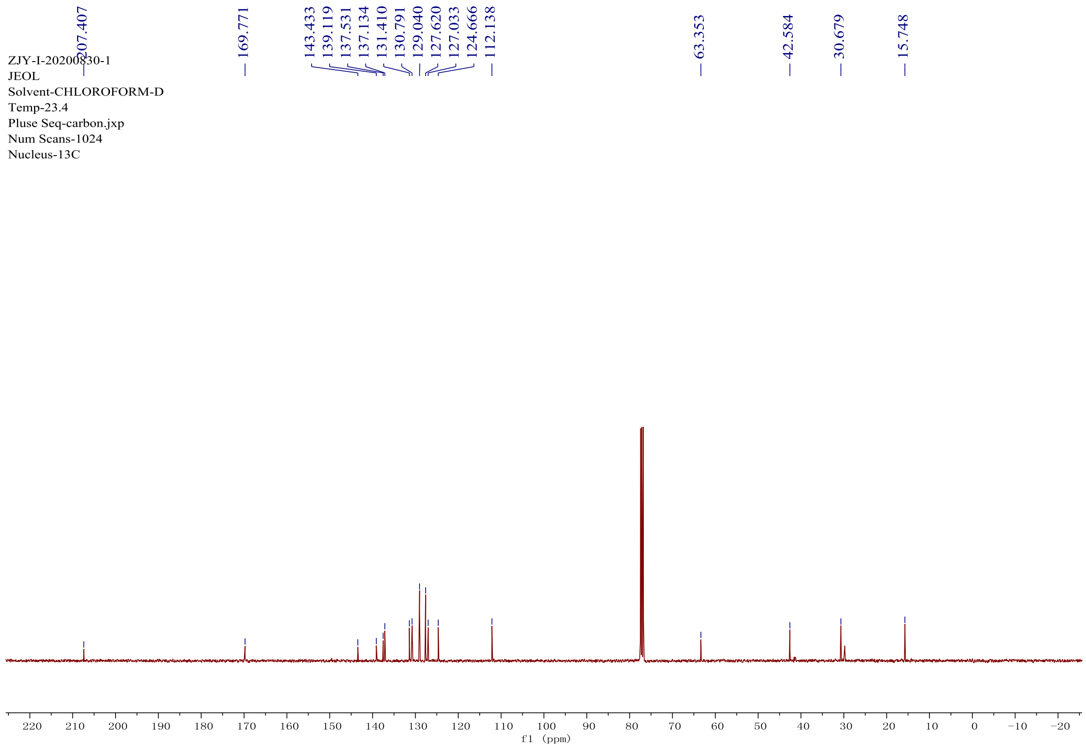

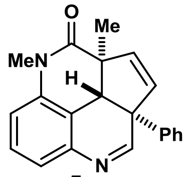

5a

^{1}H NMR of compound **5a**

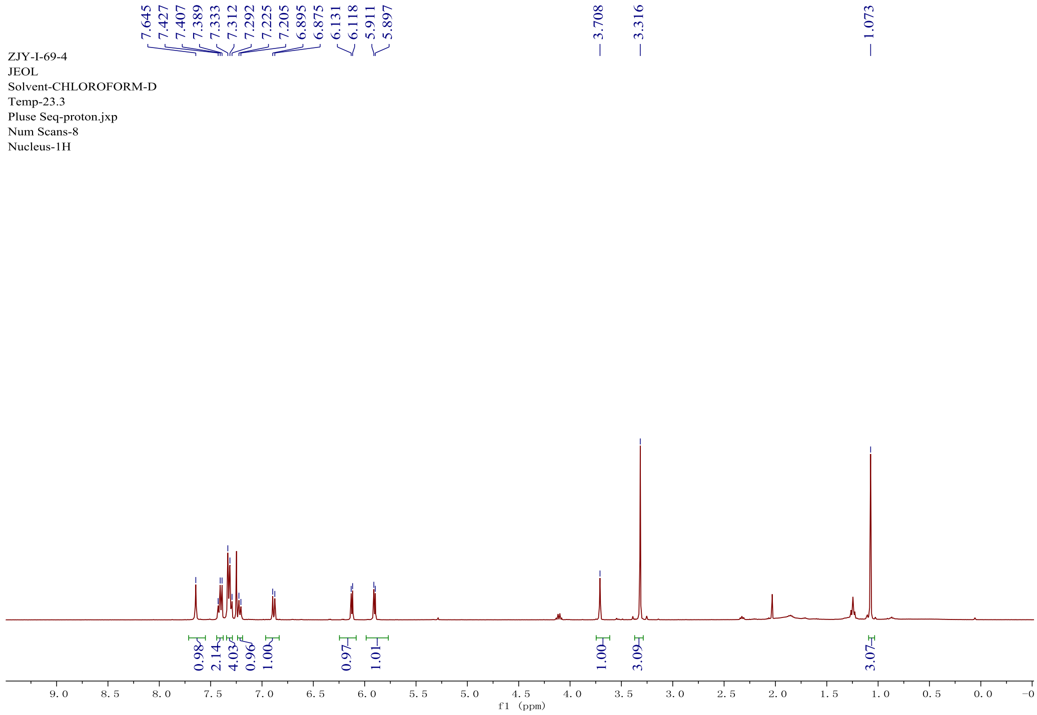

^{13}C NMR of compound **5a**

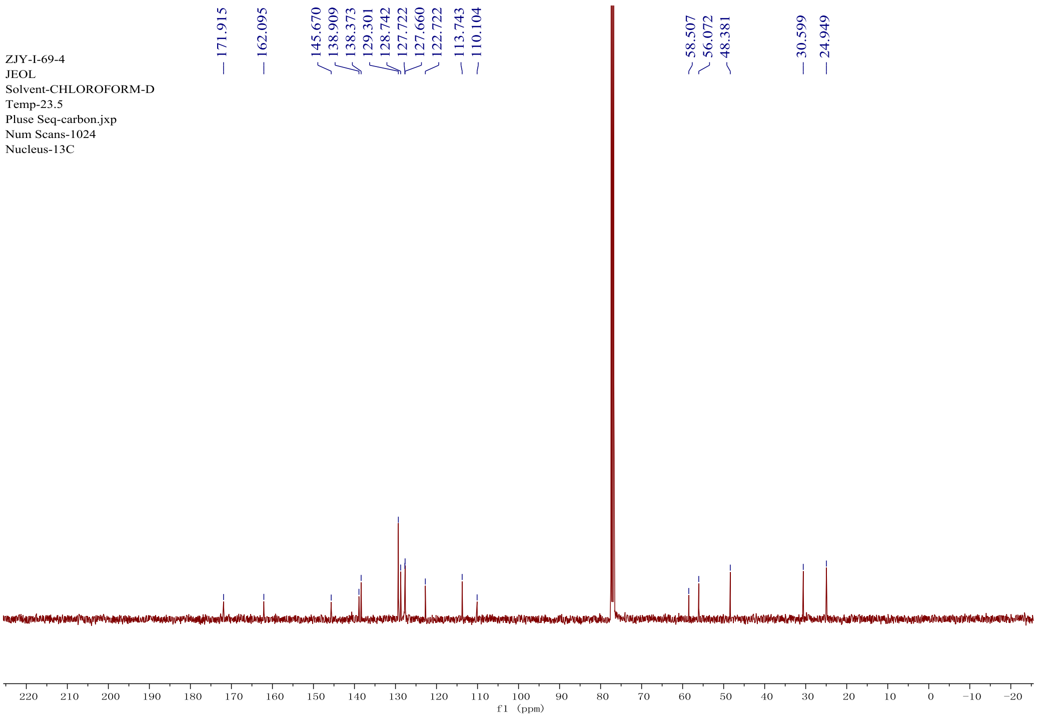

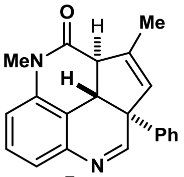

5g

^{1}H NMR of compound **5g**

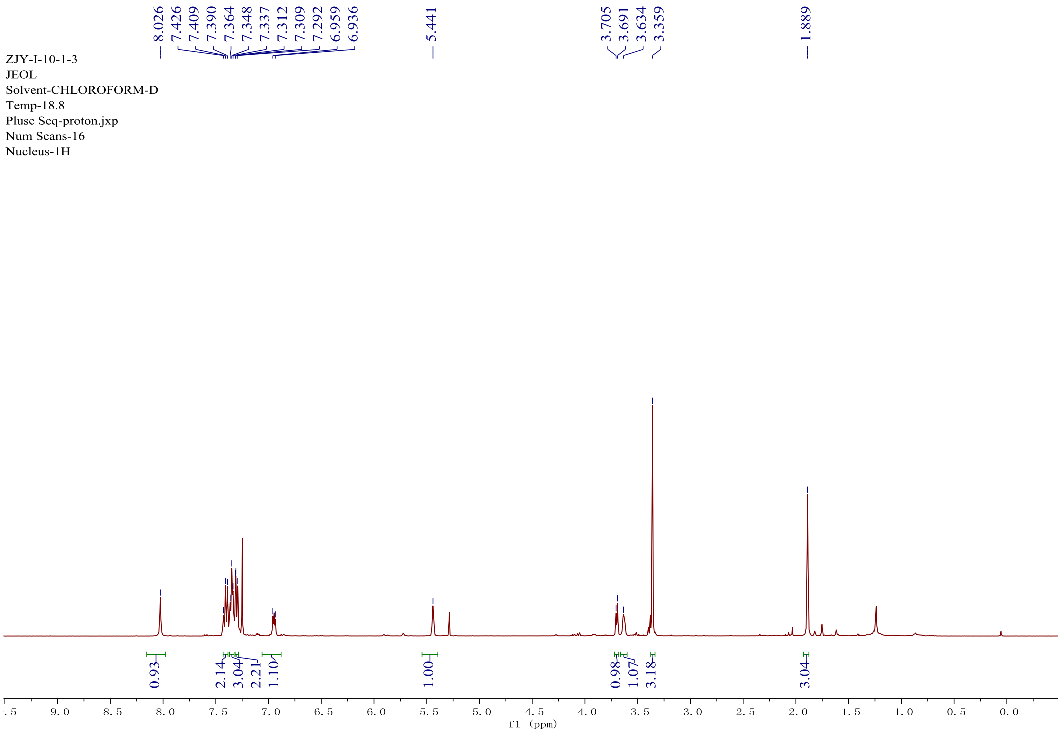

^{13}C NMR of compound **5g**

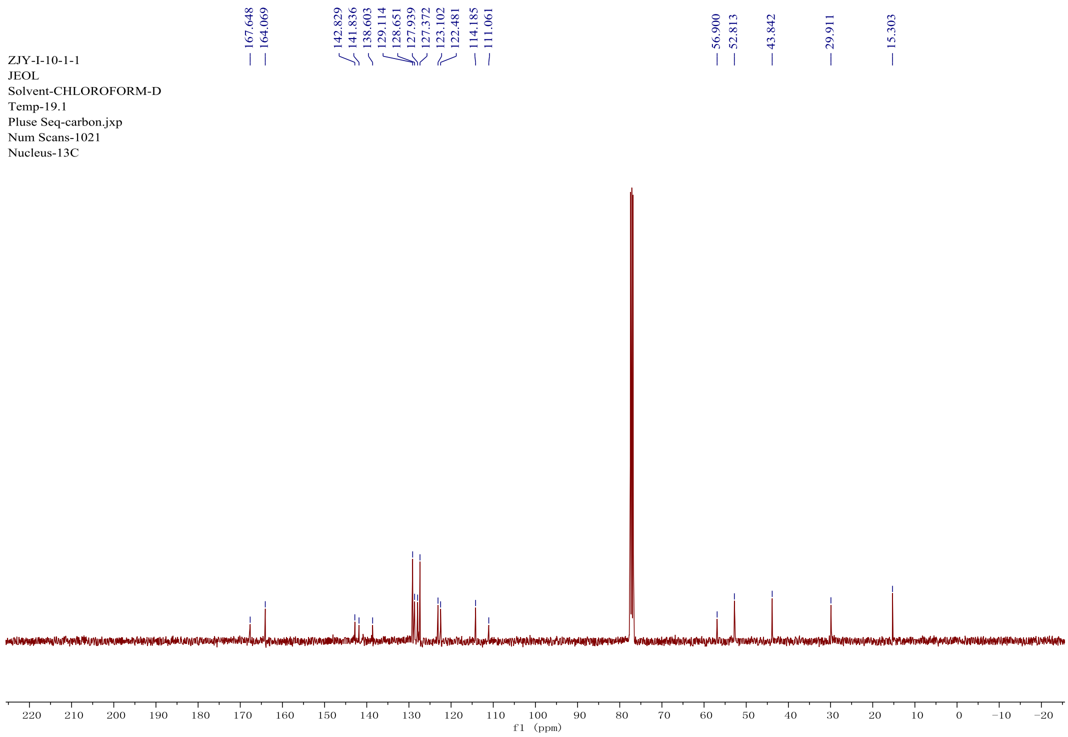

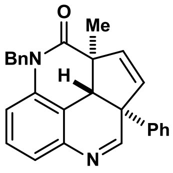

5i

^{1}H NMR of compound **5i**

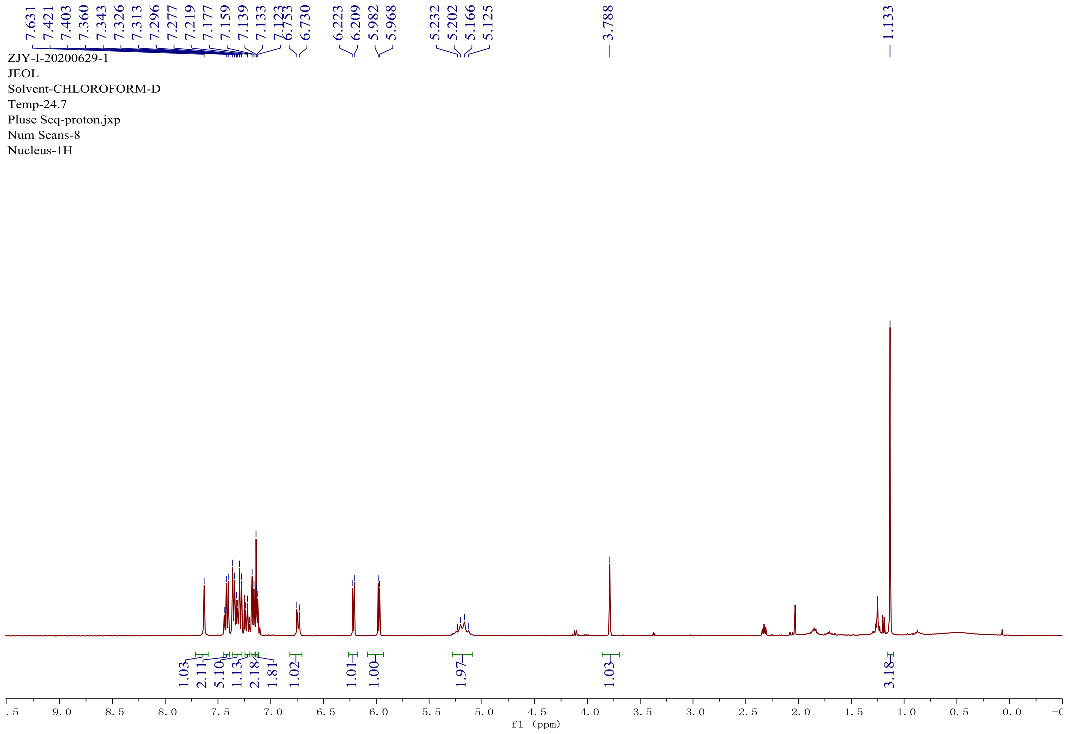

^{13}C NMR of compound **5i**

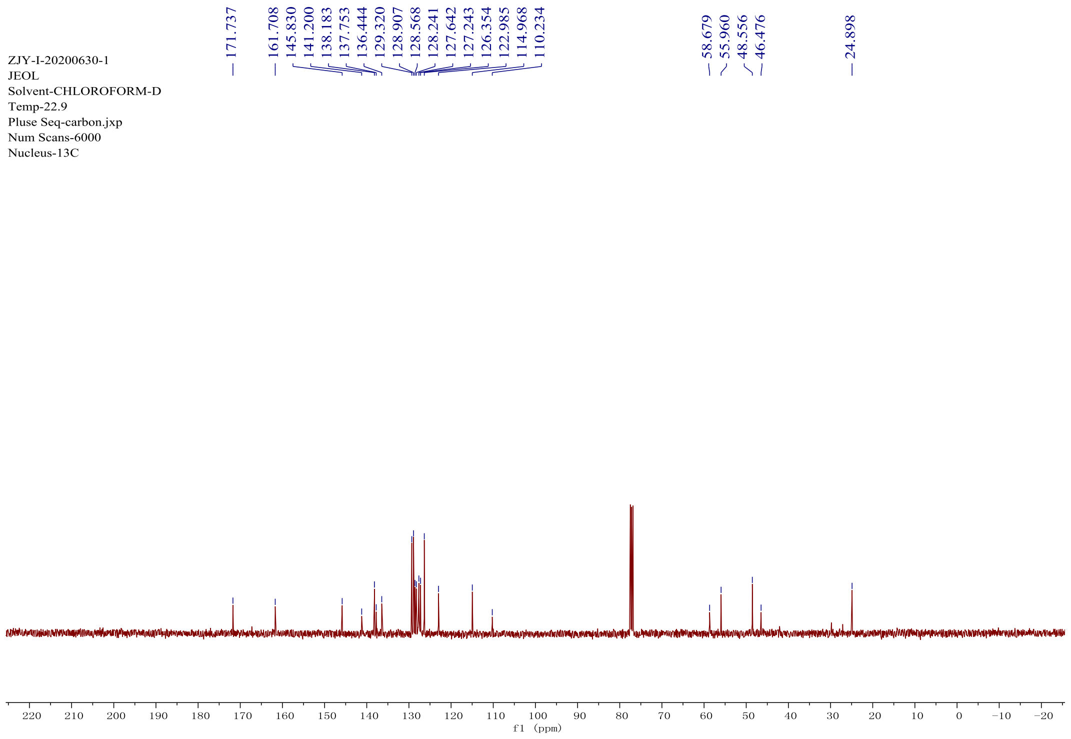

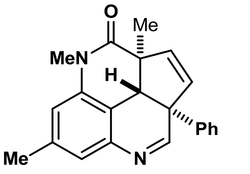

5l

^{1}H NMR of compound **5l**

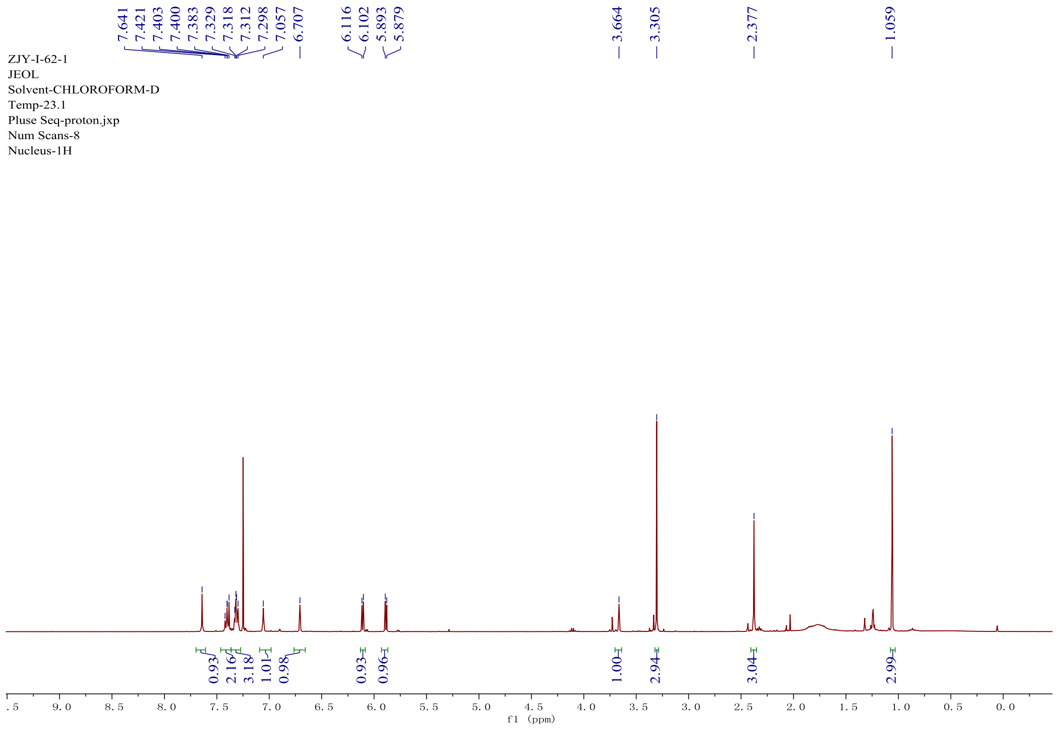

^{13}C NMR of compound **5l**

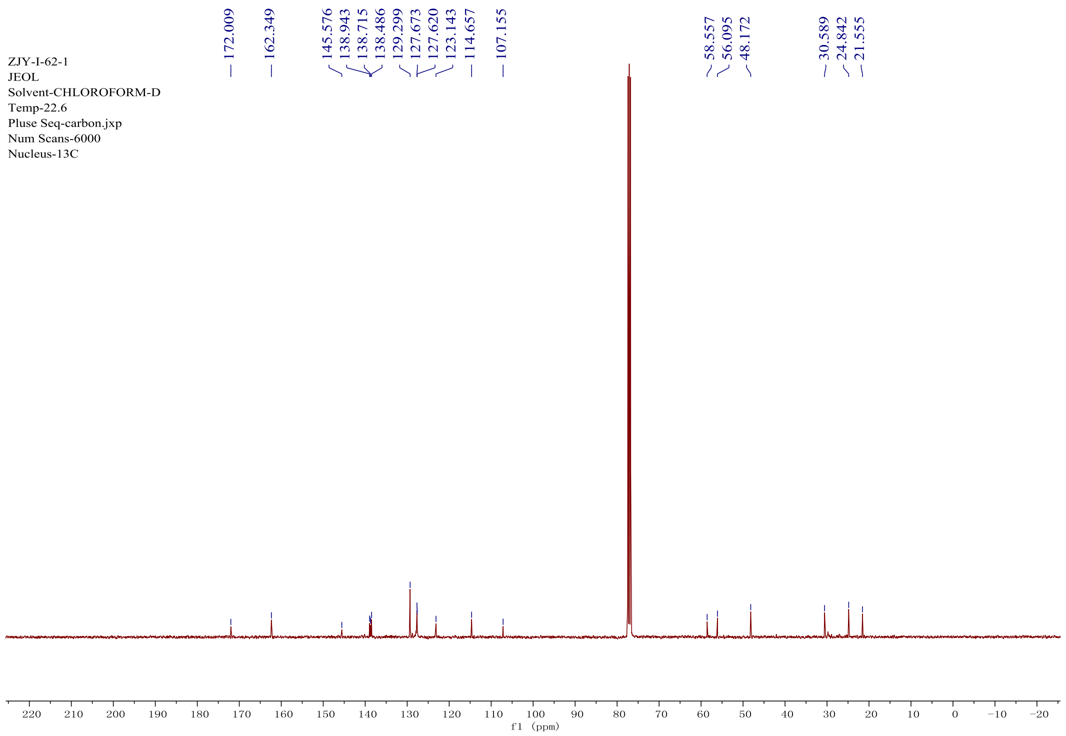

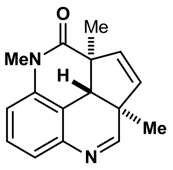

5n

^{1}H NMR of compound **5n**

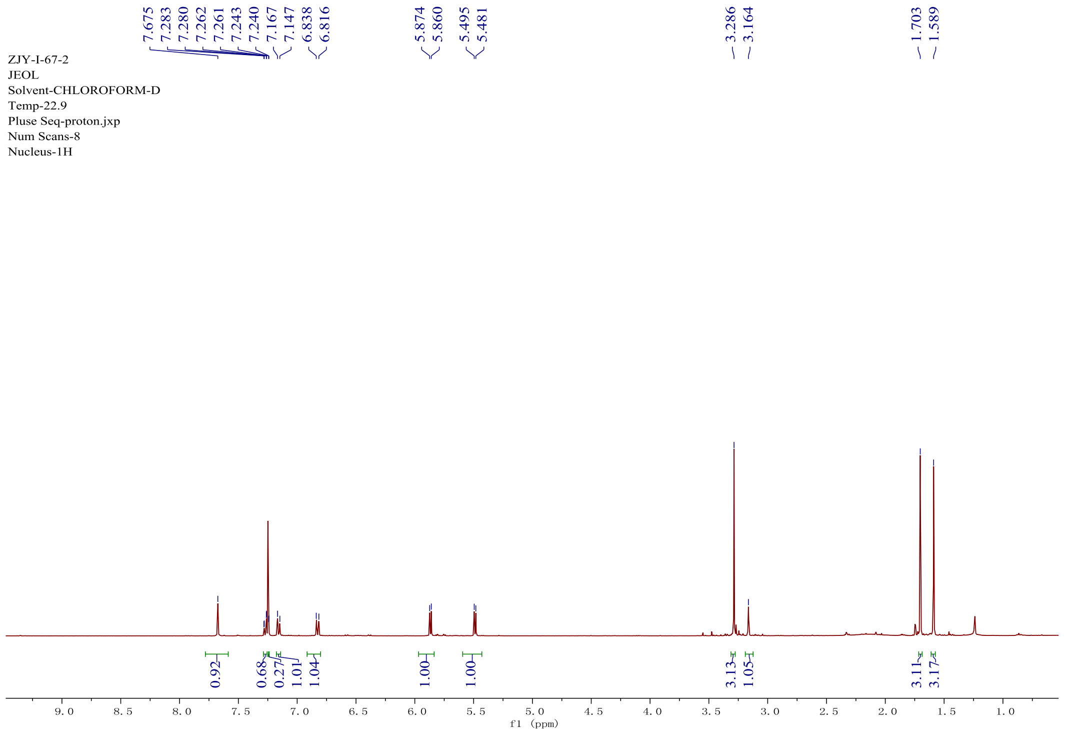

^{13}C NMR of compound **5n**

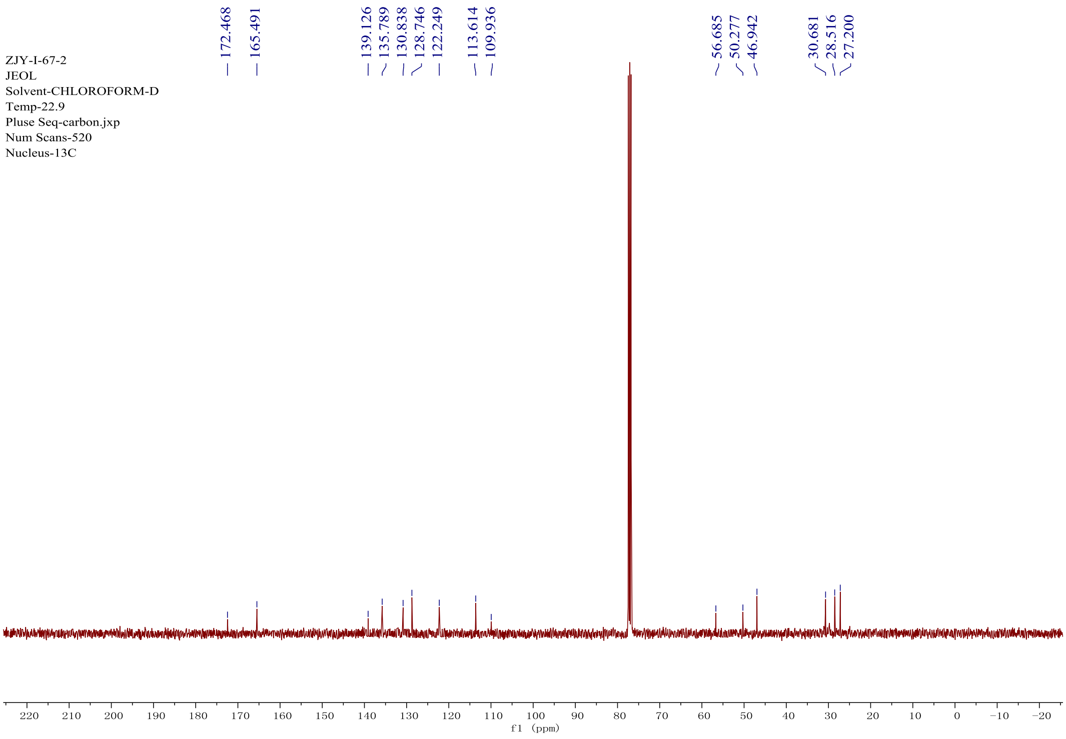

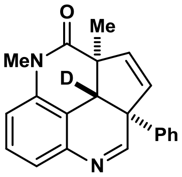

5a-D

^{1}H NMR of compound **5a-D**

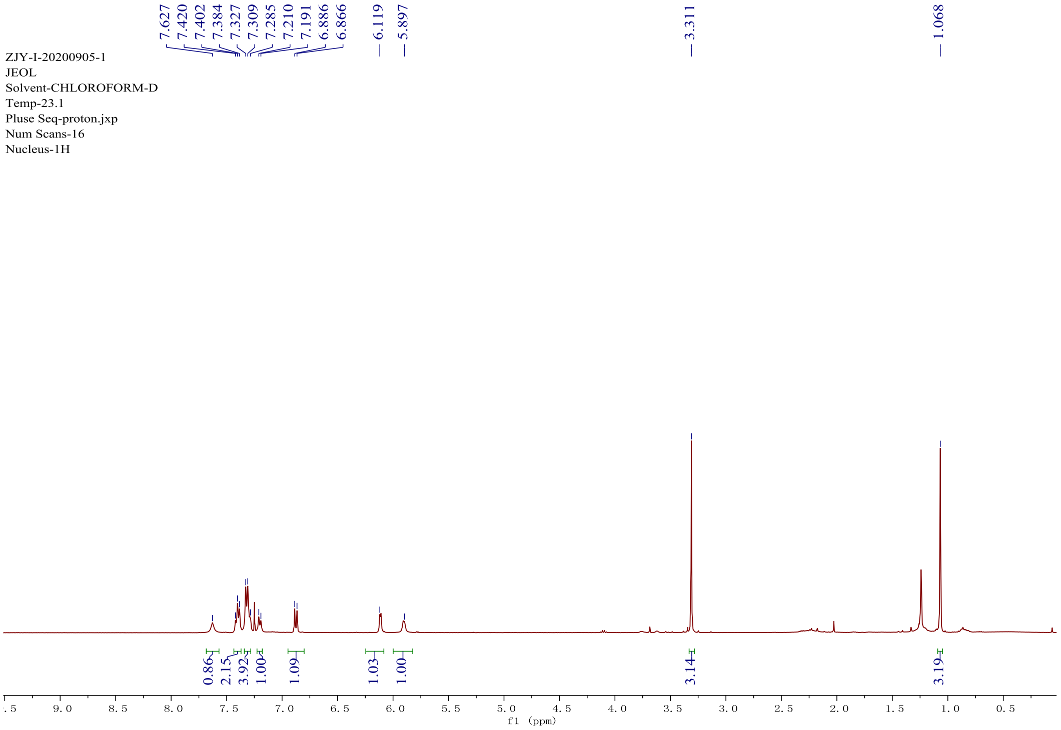

^{13}C NMR of compound **5a-D**

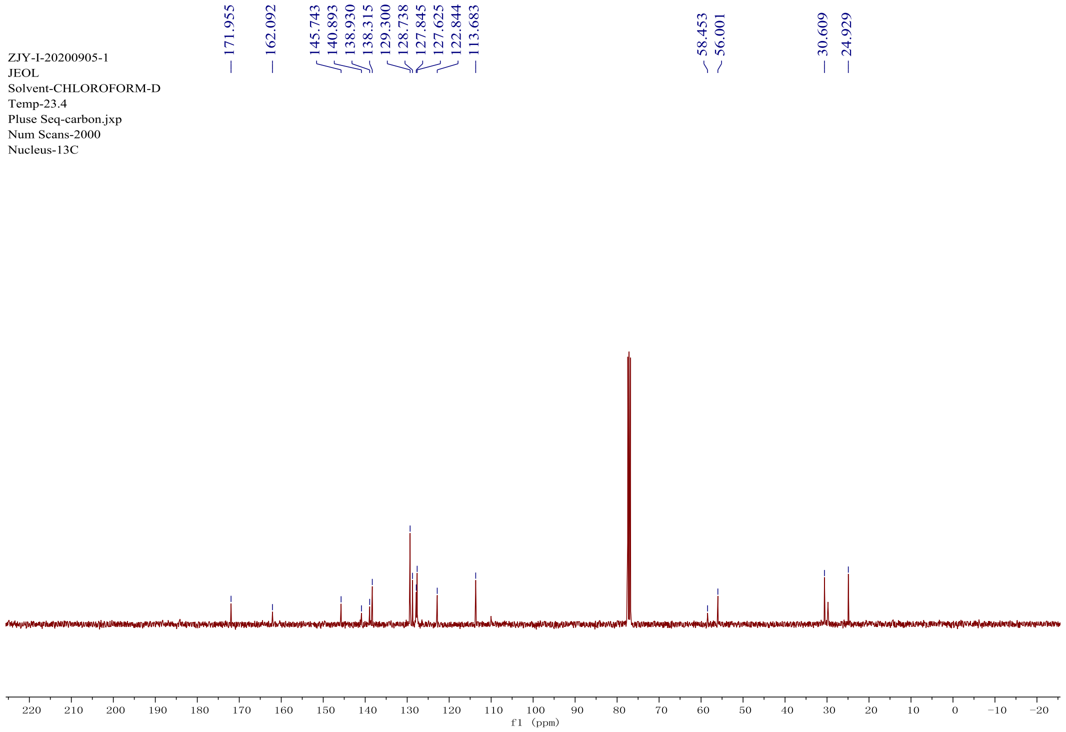

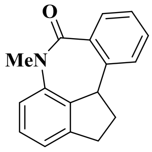

7

^{1}H NMR of compound **7**

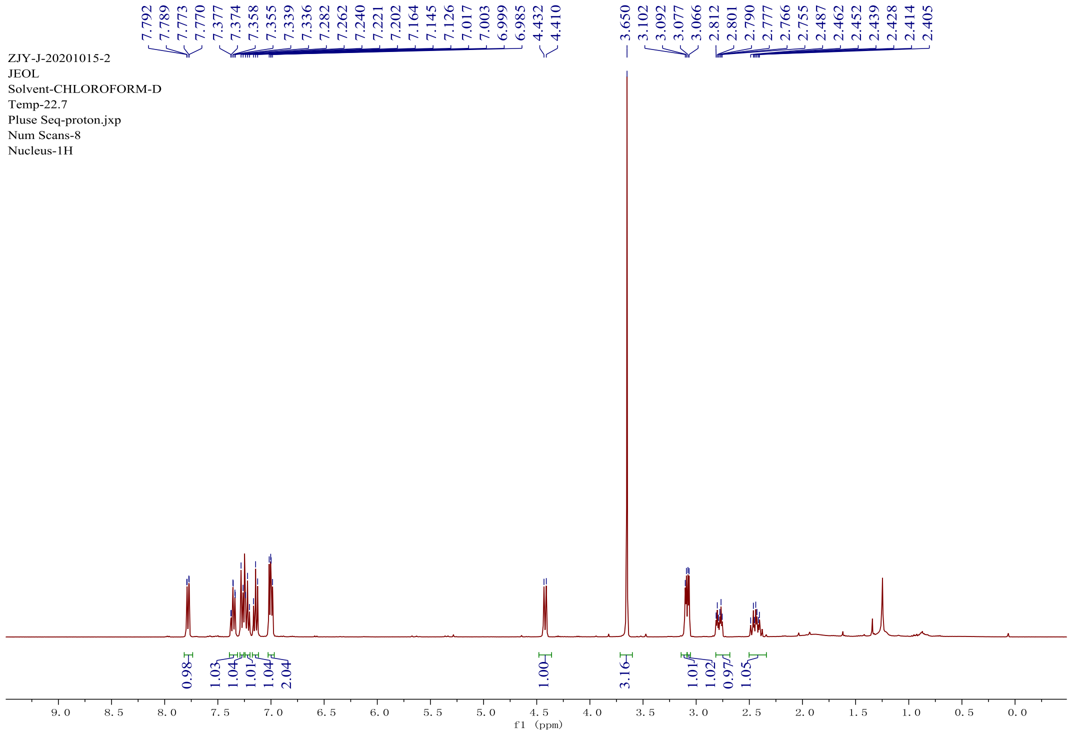

^{13}C NMR of compound **7**

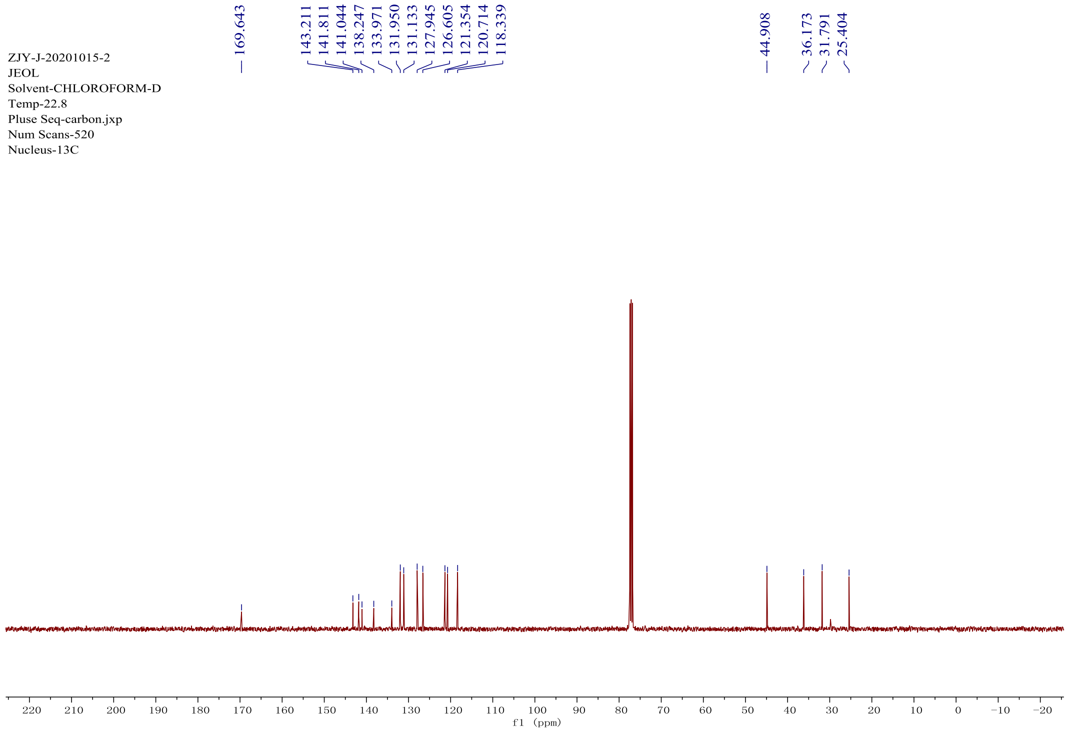

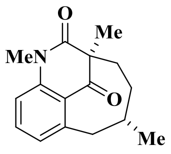

8

^{1}H NMR of compound **8**

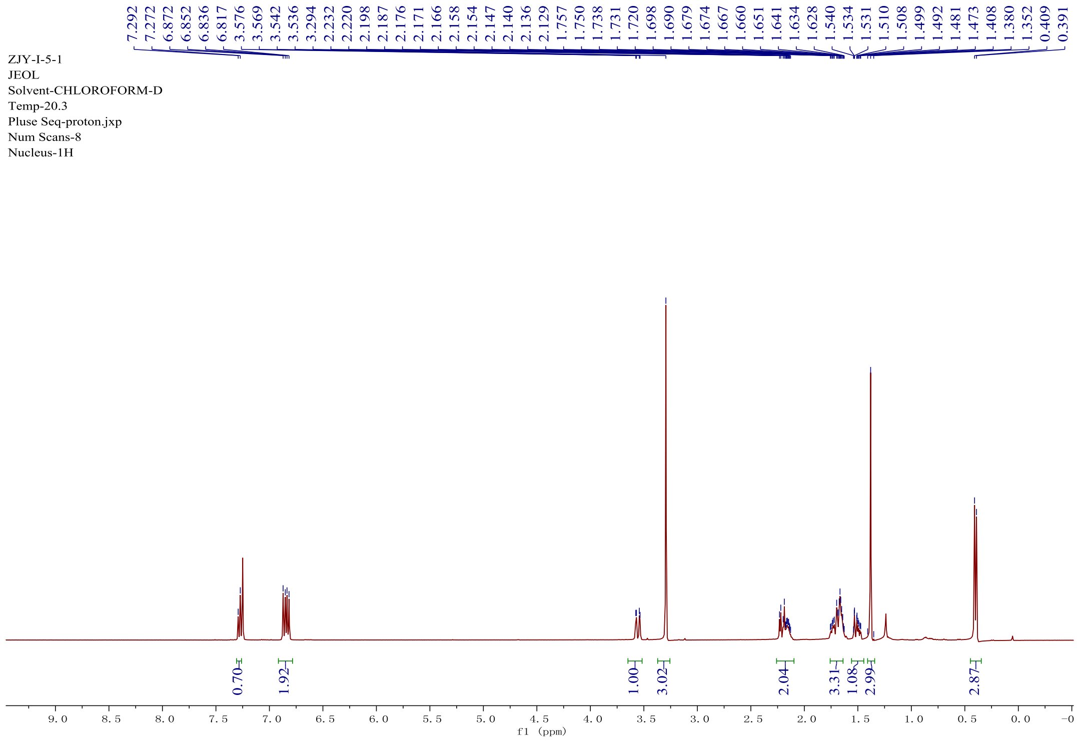

^{13}C NMR of compound **8**

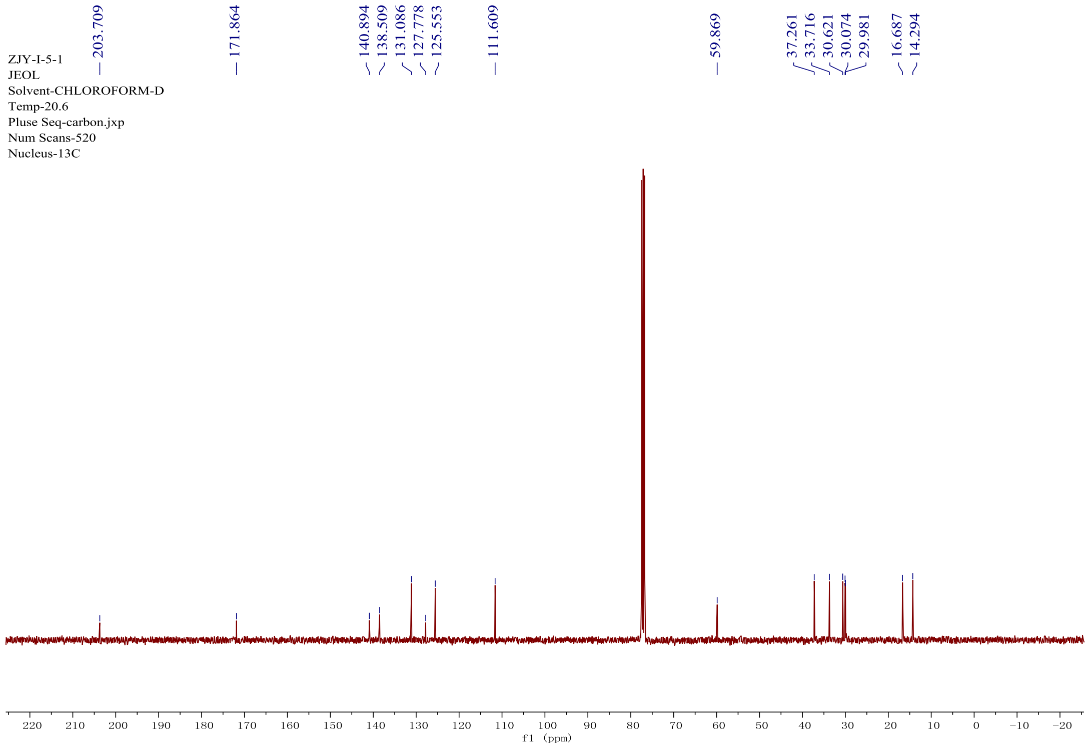

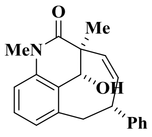

9

^{1}H NMR of compound **9**

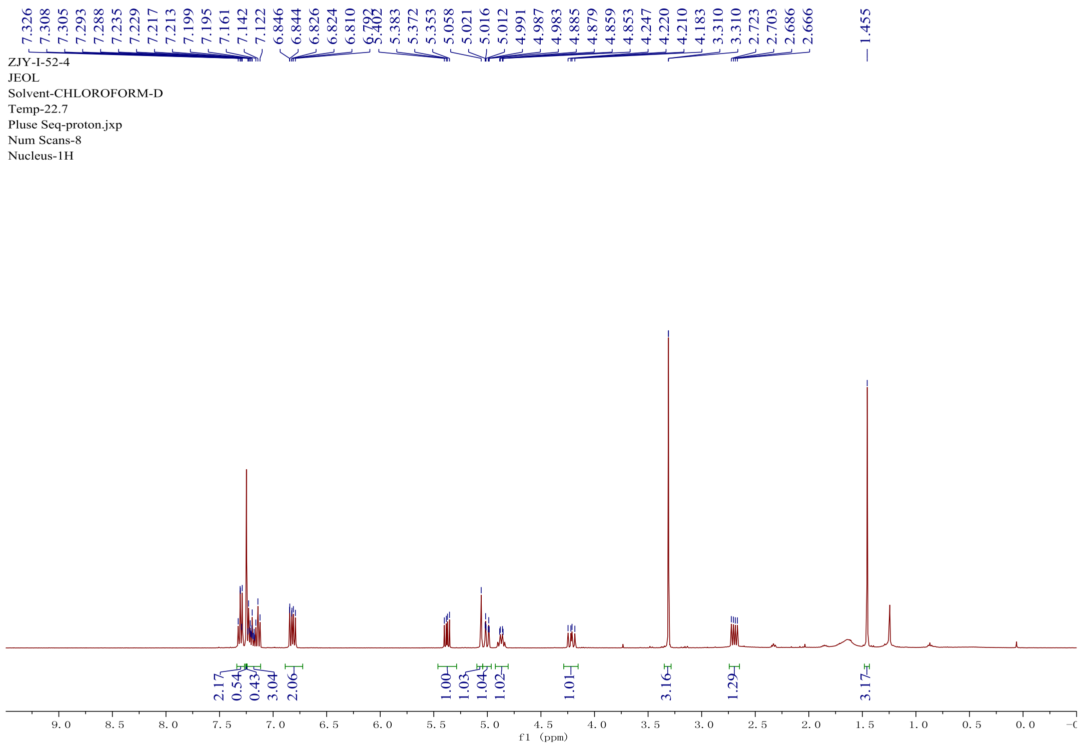

^{13}C NMR of compound **9**

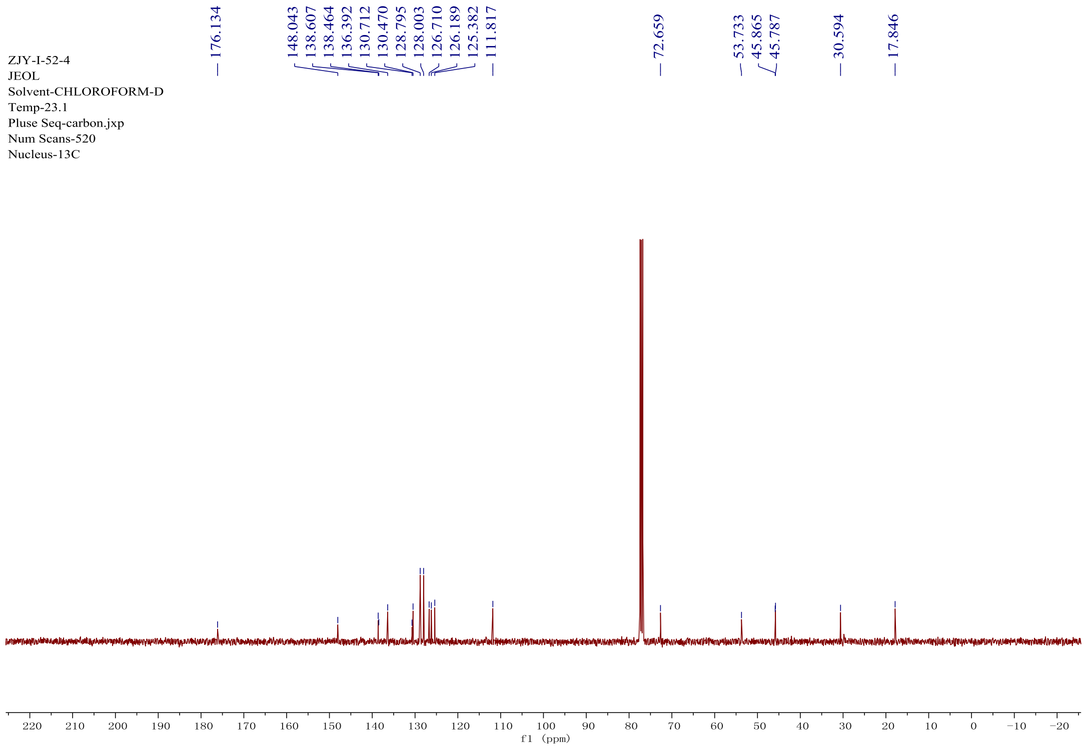

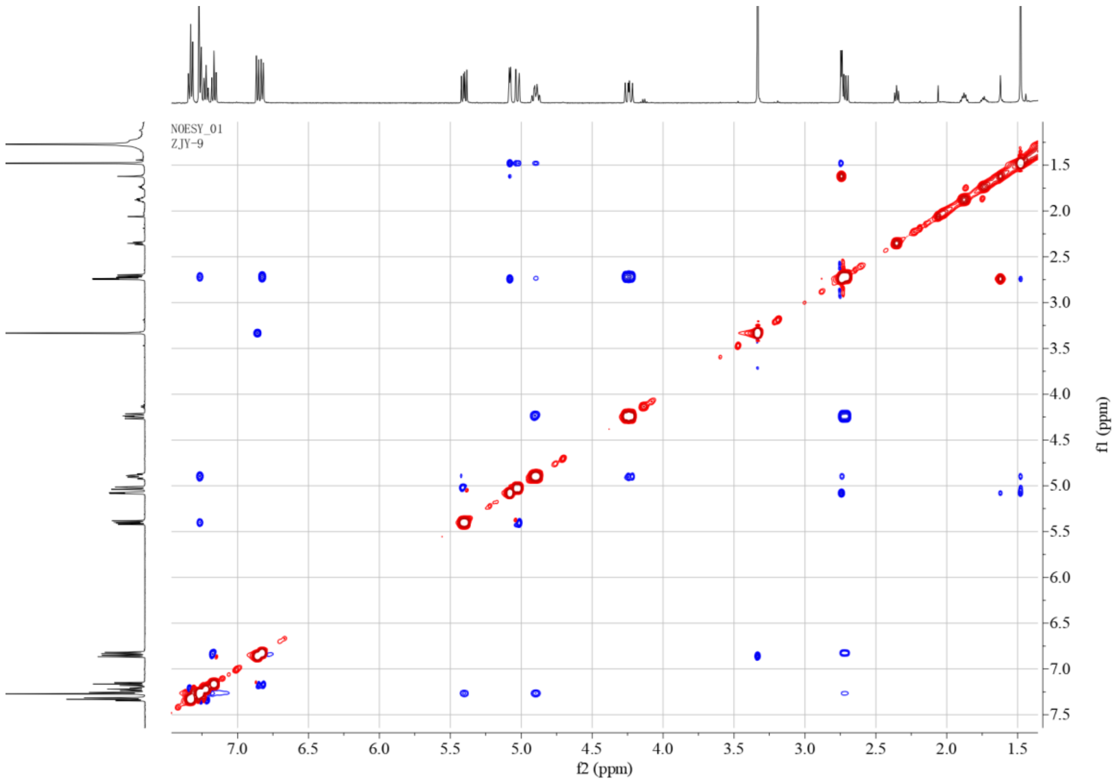
NOESY_01
ZJY-9
f1 (ppm)
f2 (ppm)

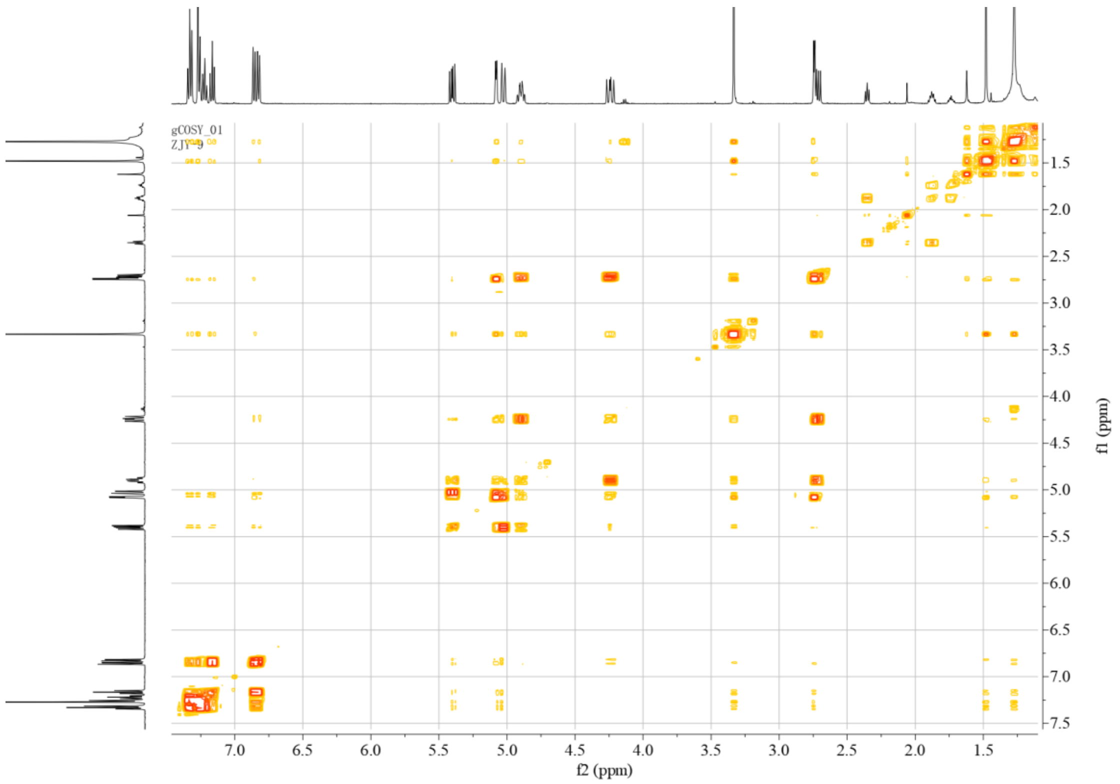
gCOSY_01
ZJY-9
f1 (ppm)
f2 (ppm)

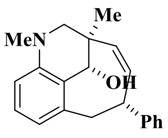

10

^{1}H NMR of compound **10**

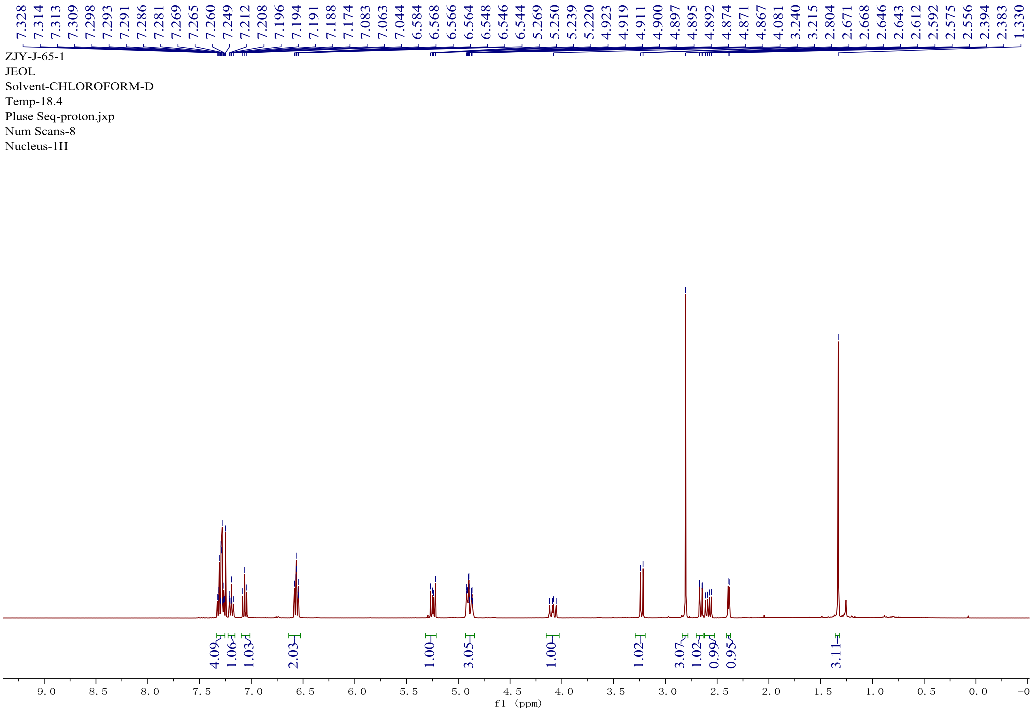

^{13}C NMR of compound **10**

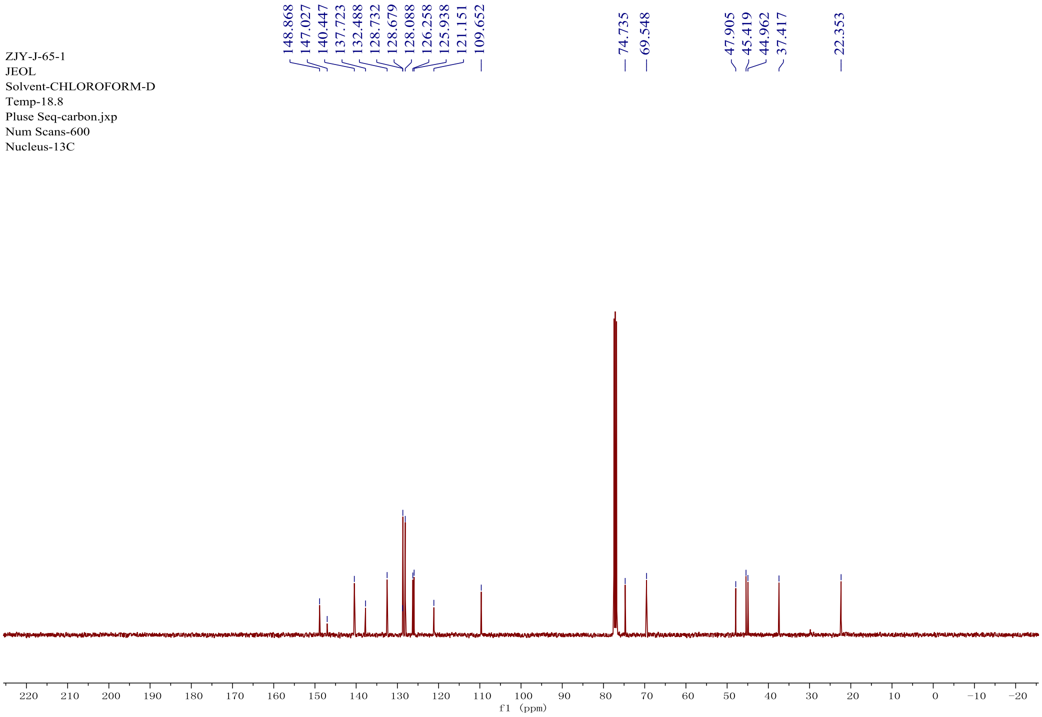

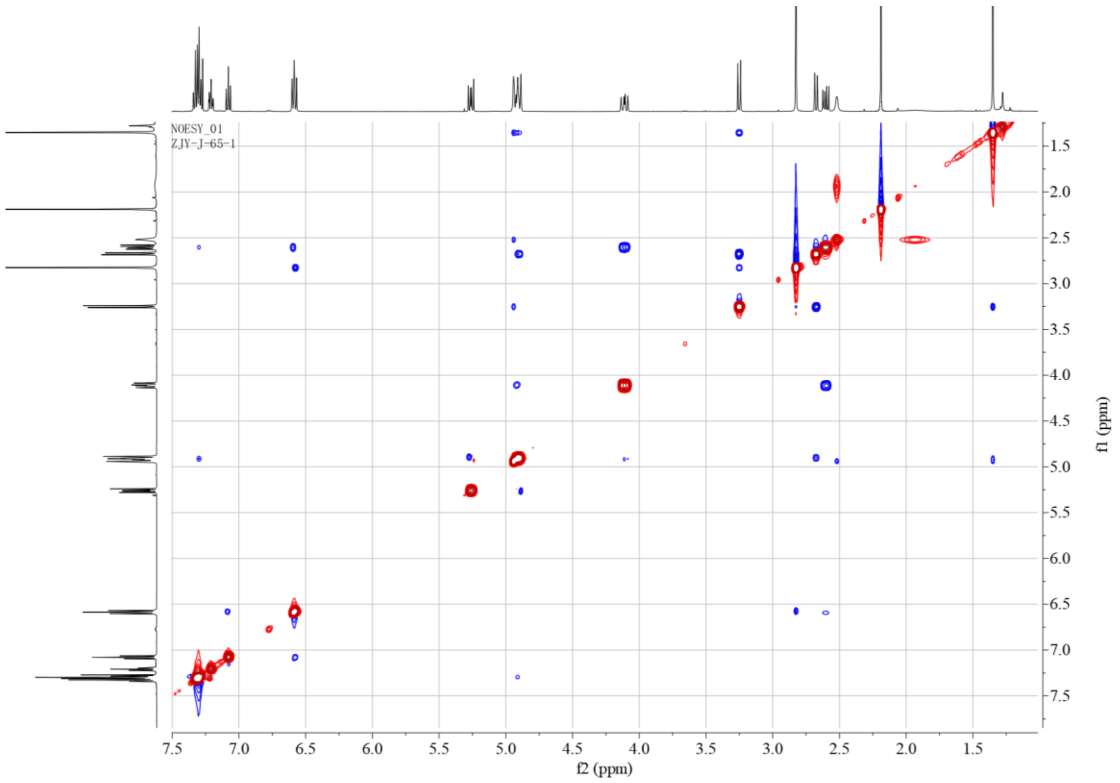
NOESY_01
ZJY-J-65-1
f2 (ppm)
f1 (ppm)

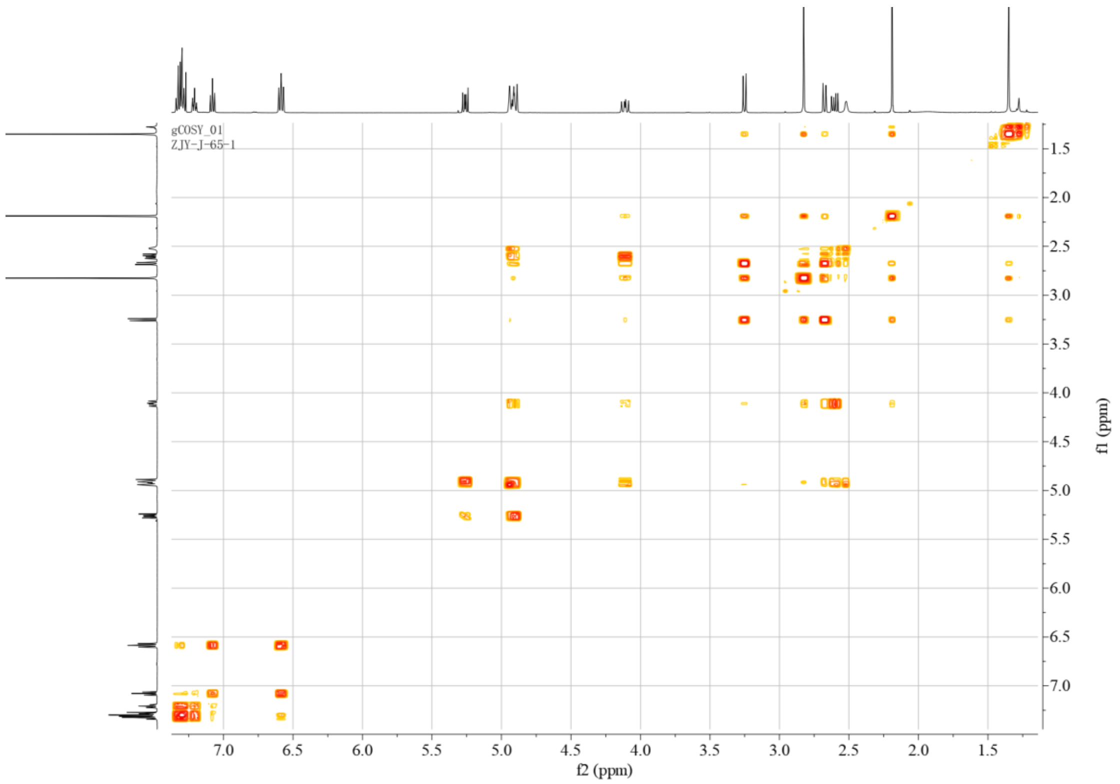
gCOSY_01
ZJY-J-65-1
f2 (ppm)
f1 (ppm)

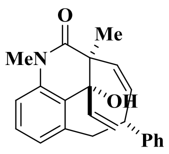

11

^{1}H NMR of compound **11**

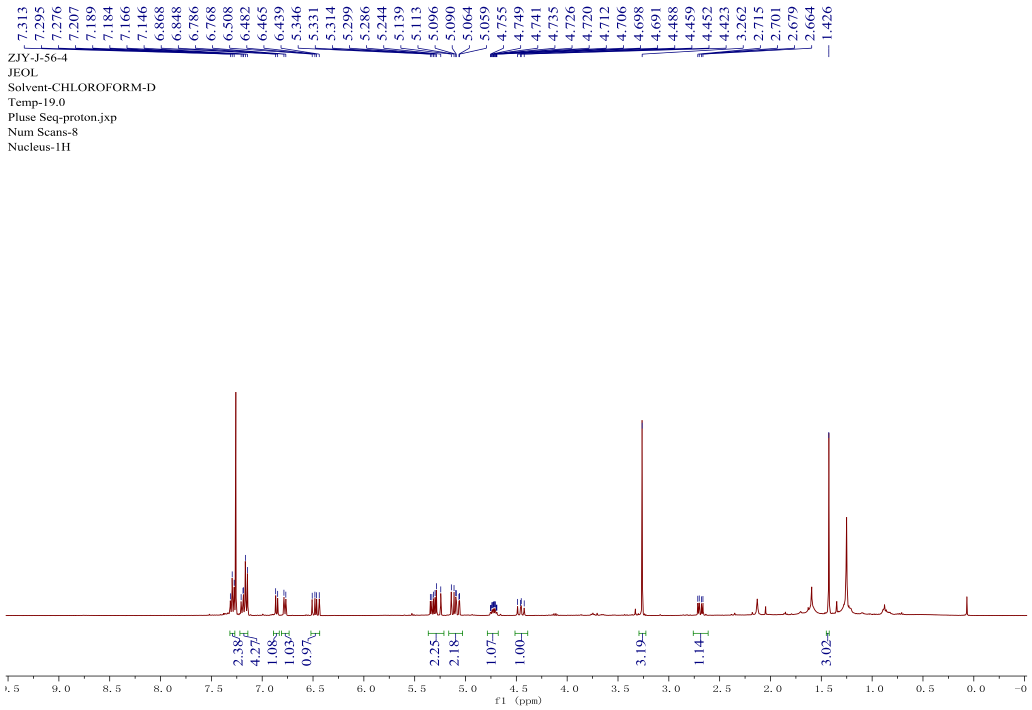

^{13}C NMR of compound **11**

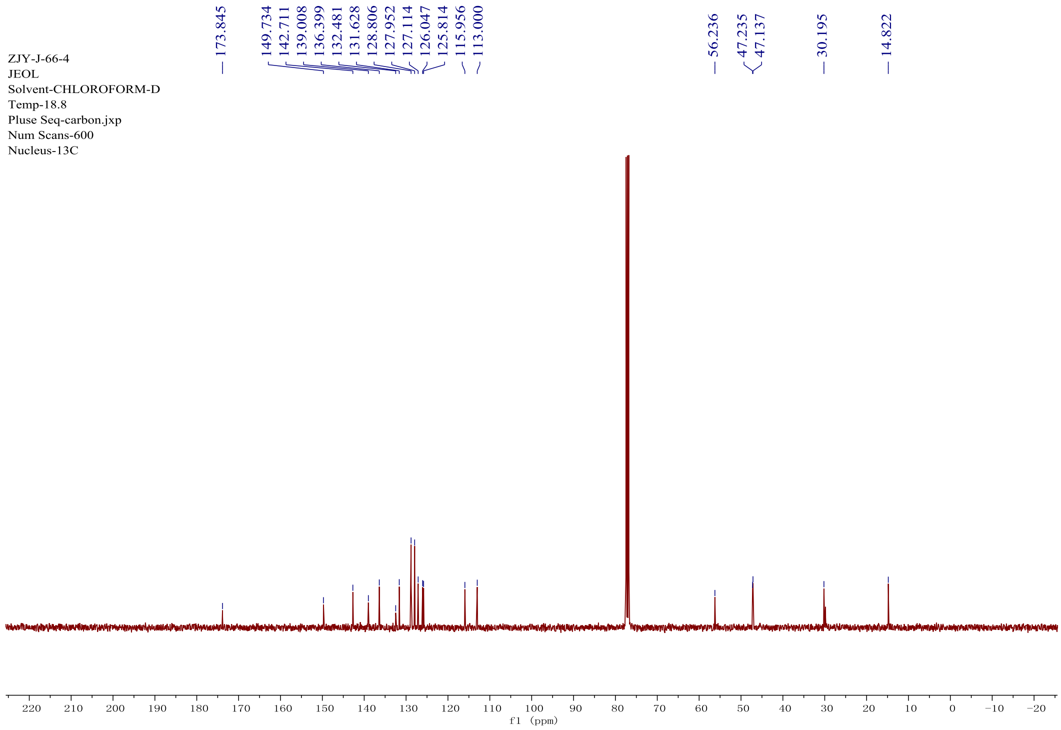

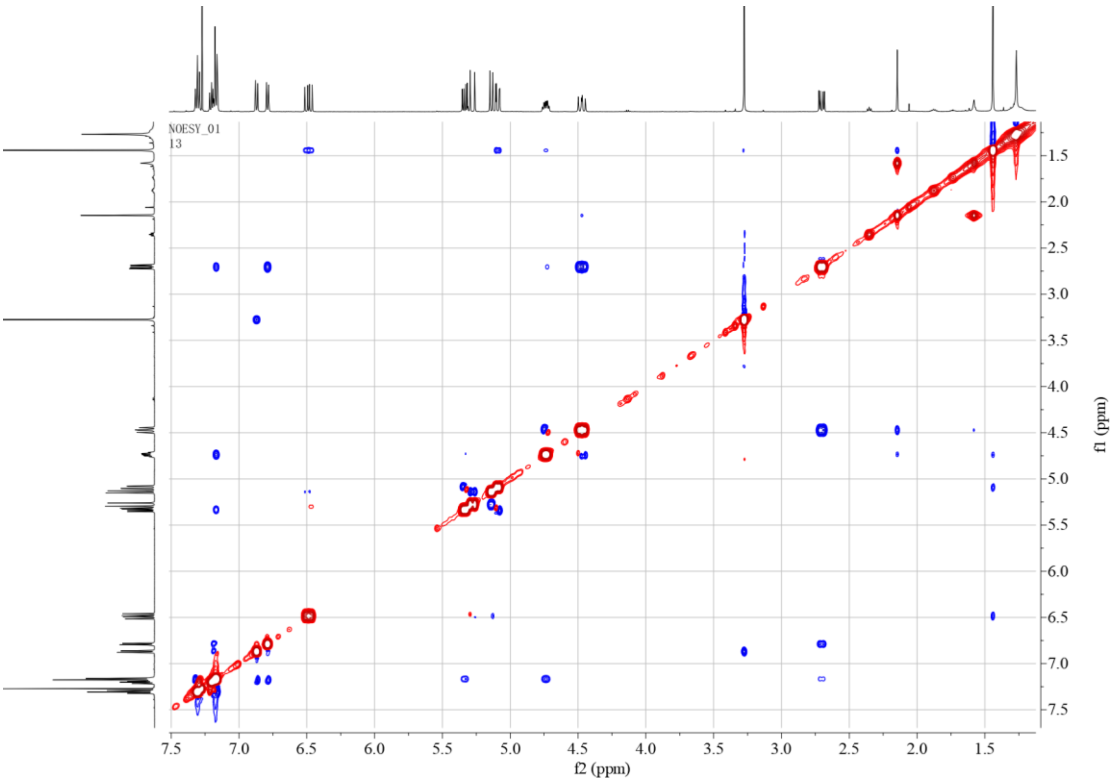

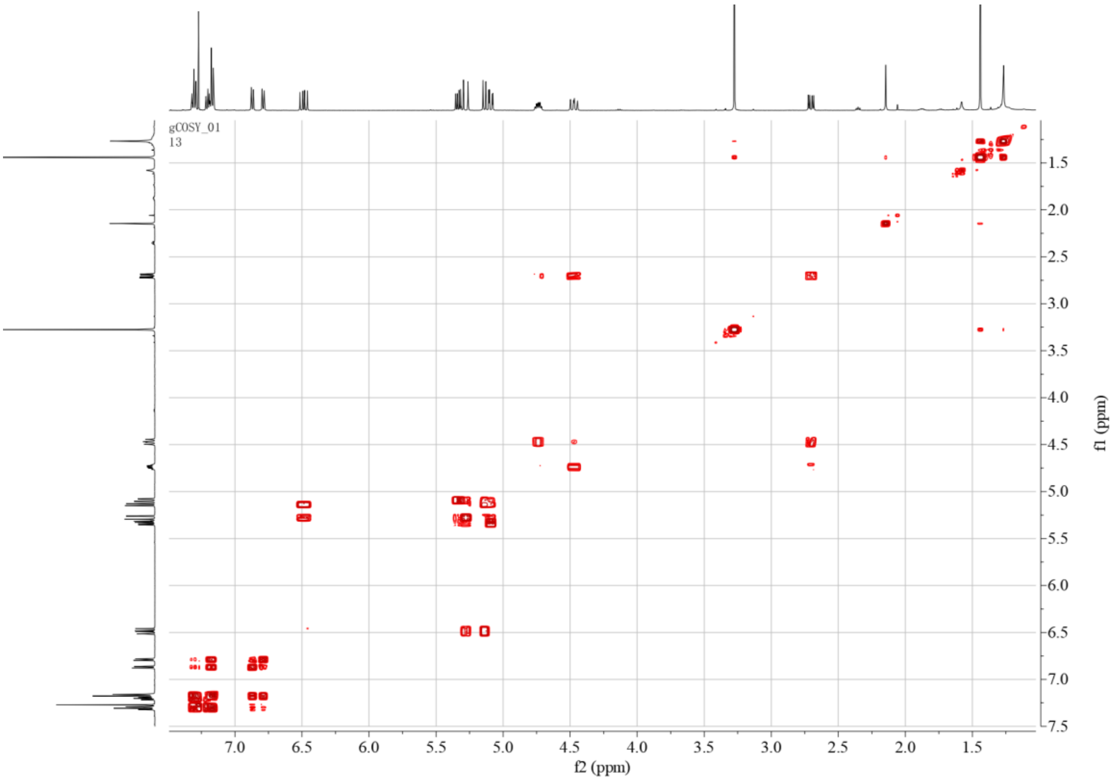

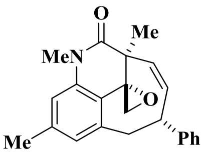

12

^{1}H NMR of compound **12**

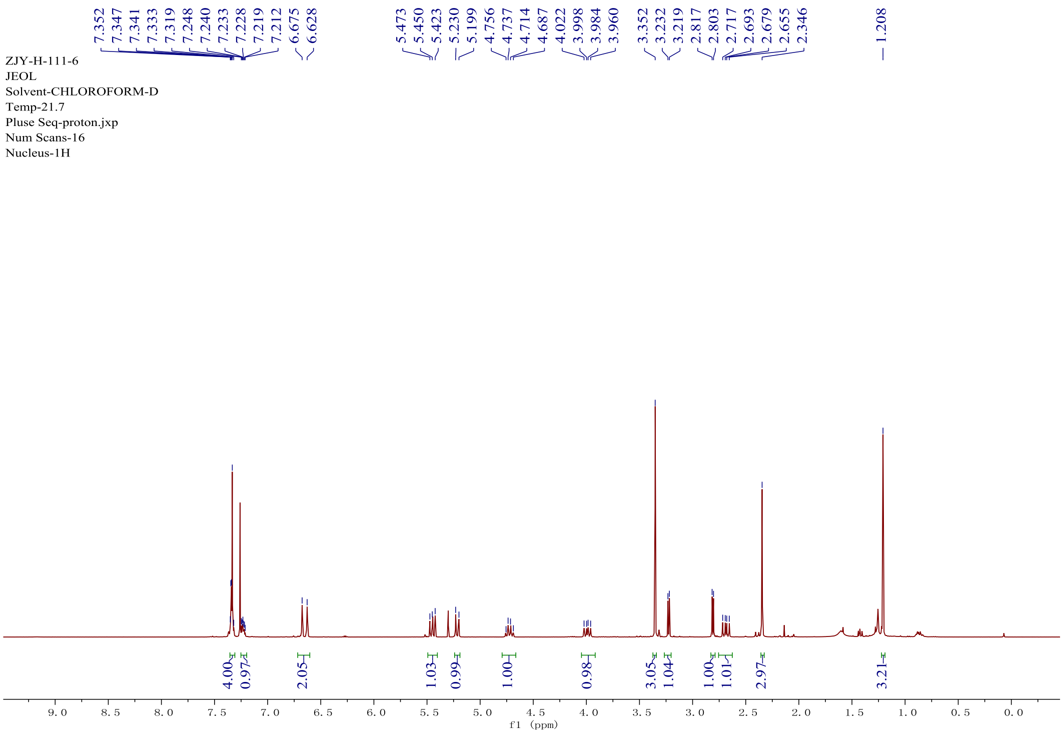

^{13}C NMR of compound **12**

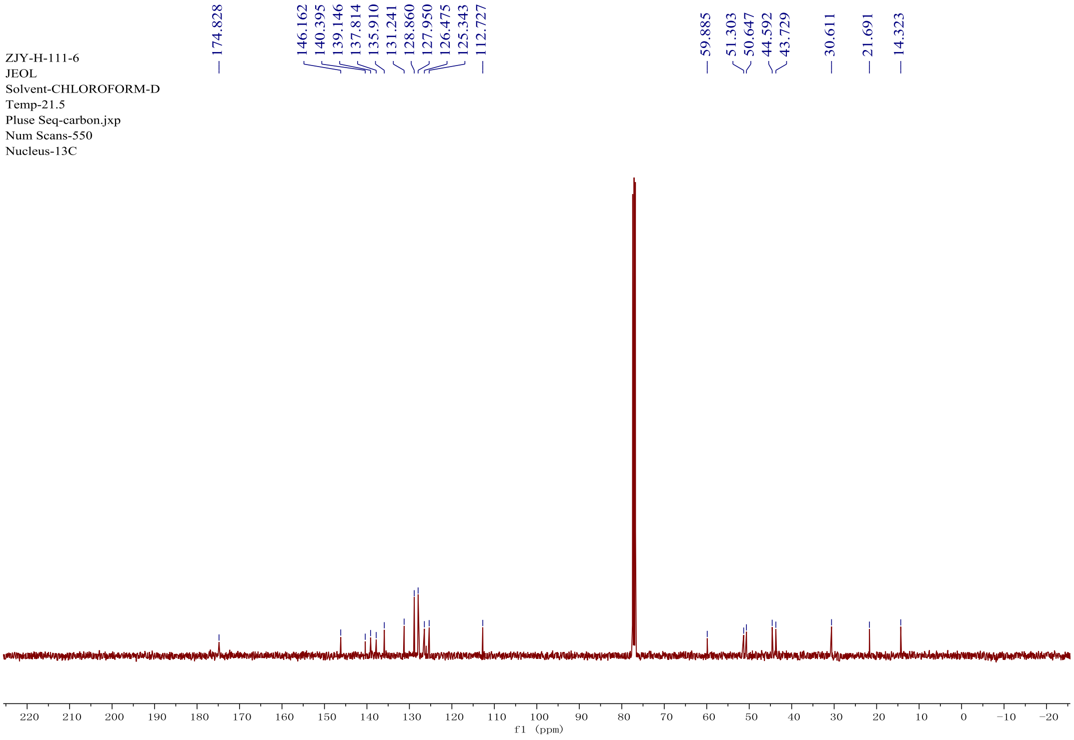

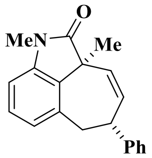

13

^{1}H NMR of compound **13**

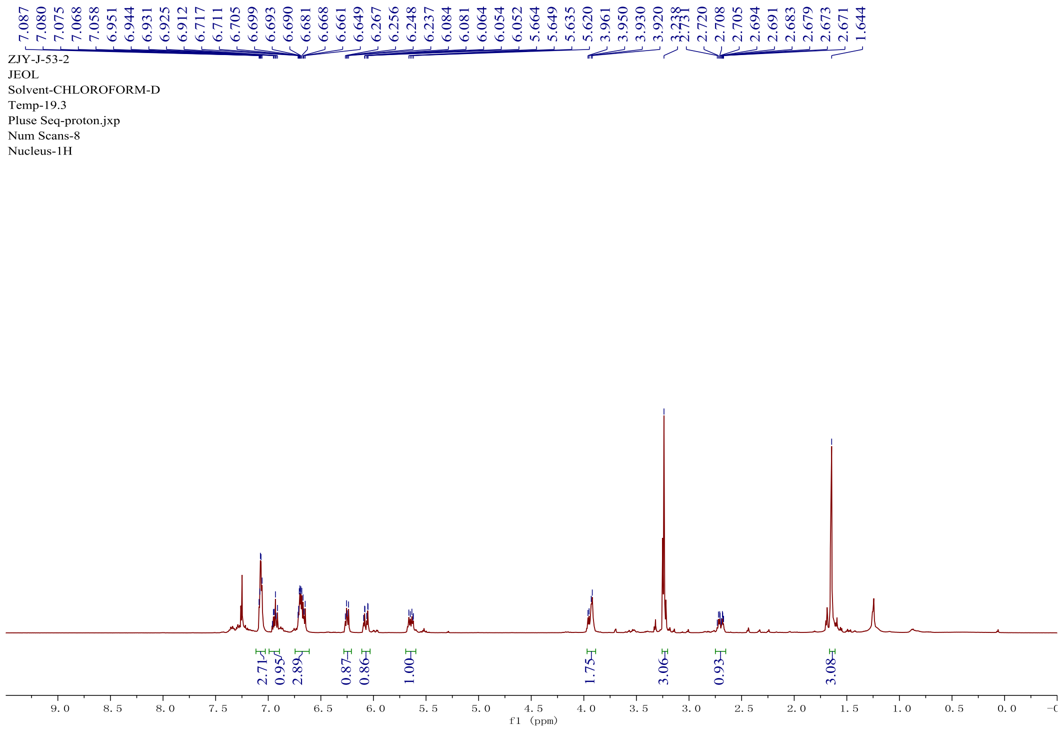

^{13}C NMR of compound **13**

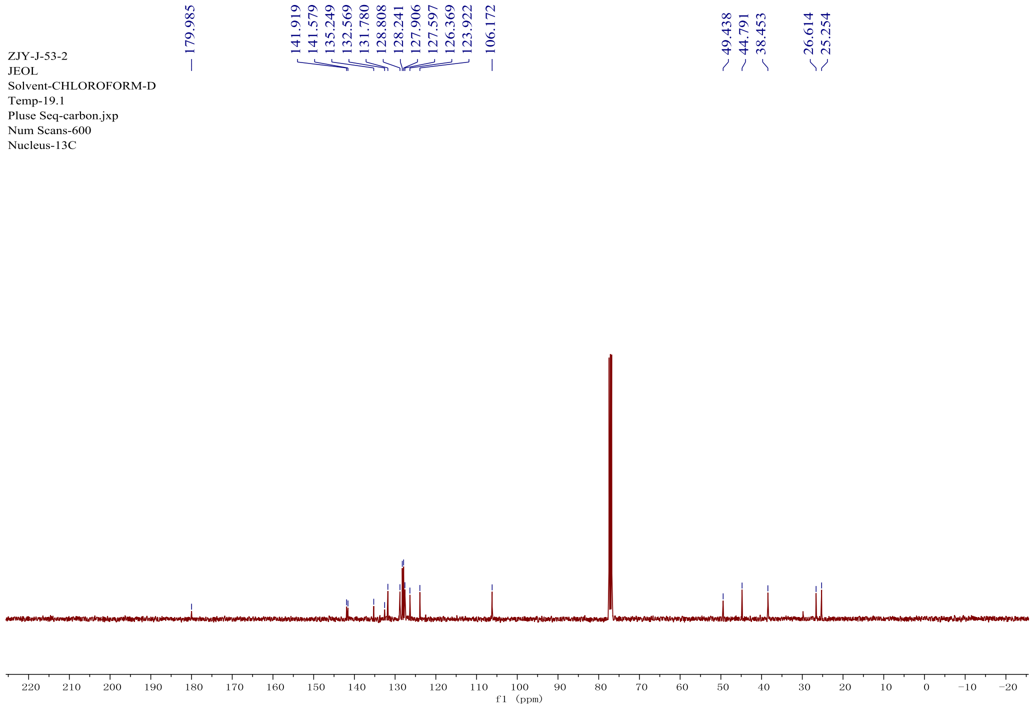

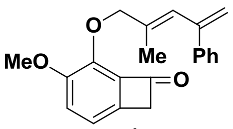

1w

^{1}H NMR of compound **1w**

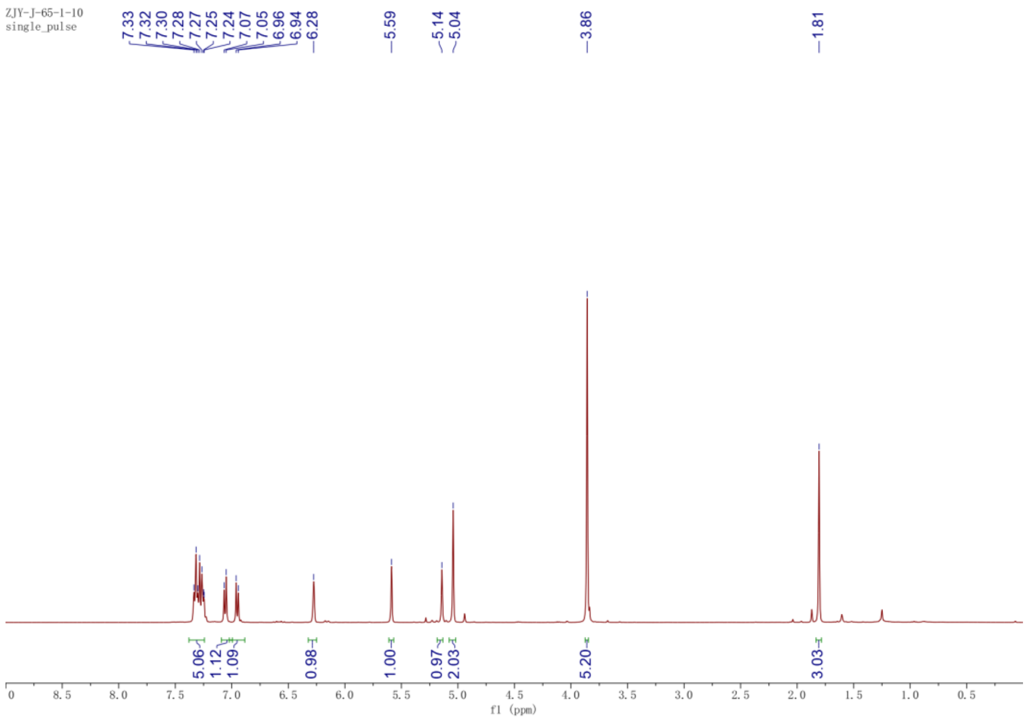

^{13}C NMR of compound **1w**

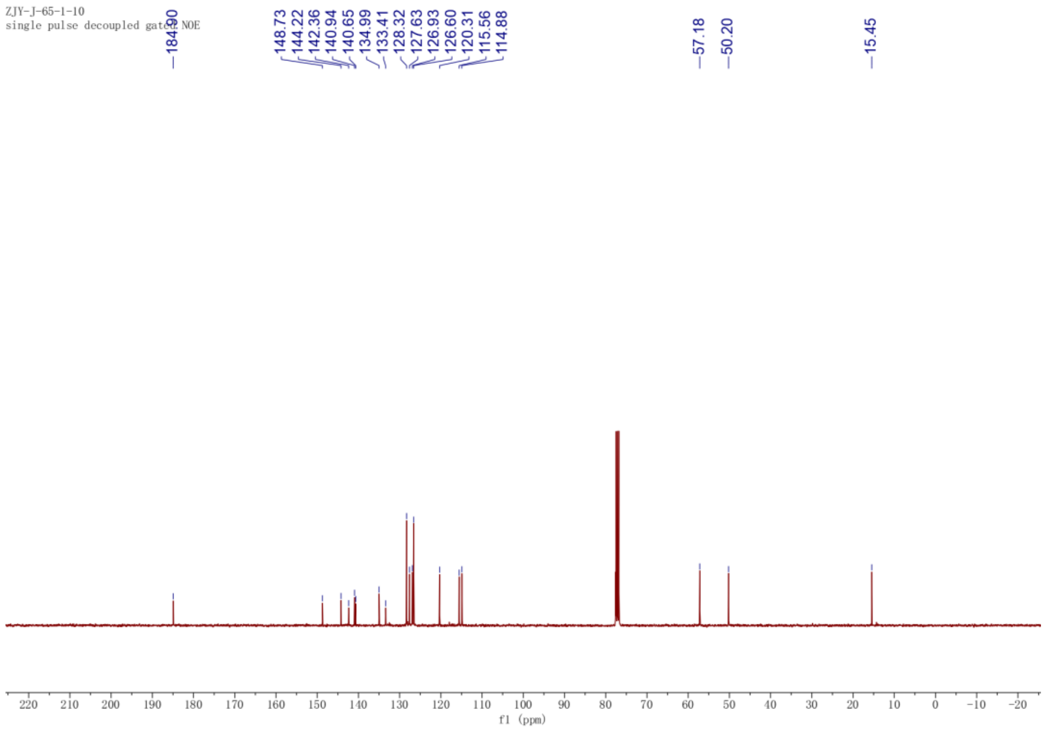

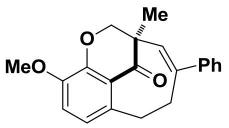

2w

^{1}H NMR of compound **2w**

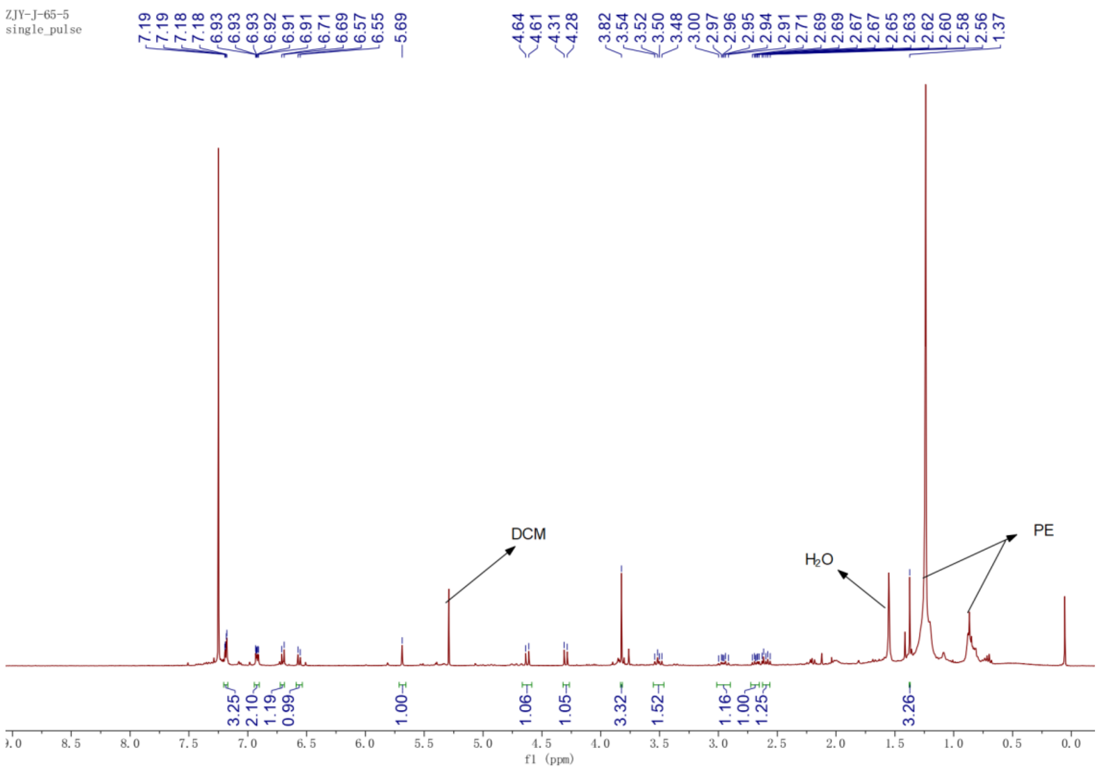

^{13}C NMR of compound **2w**

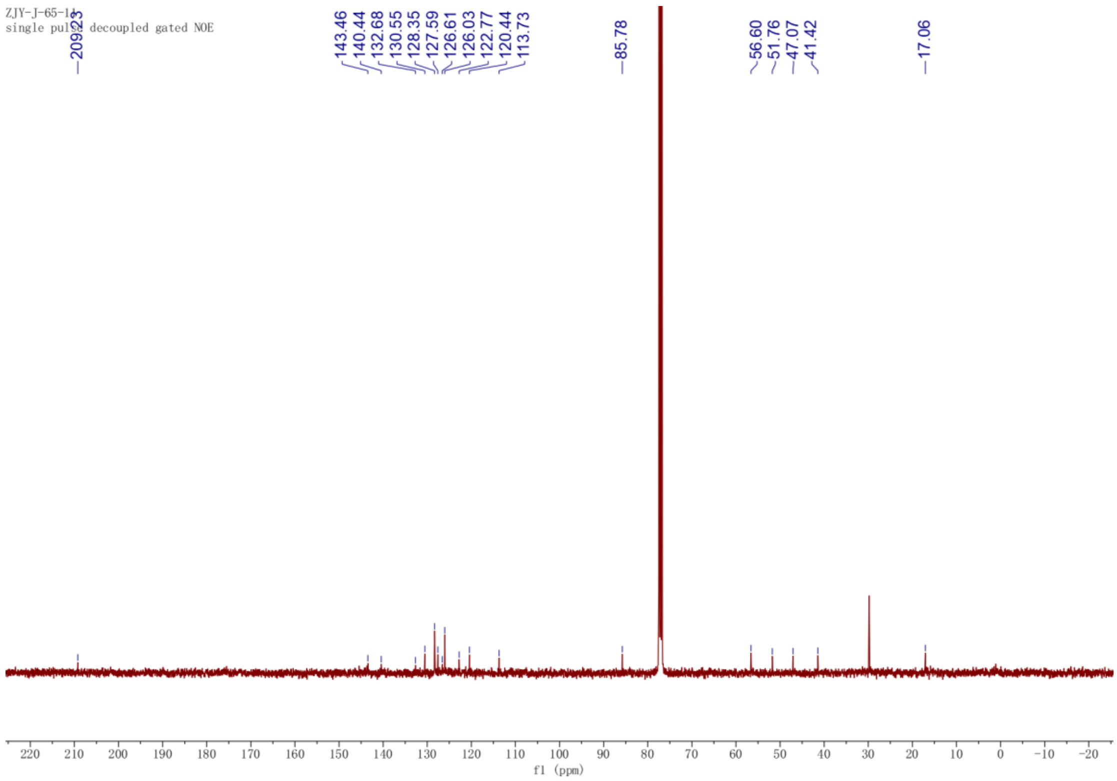

Supplement: Supplementary file 1 — Supplementary Information [file 41467_2021_23344_MOESM1_ESM.pdf]
